# Mendelian randomization reveals probucol’s preventive role in Behçet’s disease via circulating metabolites

**DOI:** 10.1038/s41598-025-93644-8

**Published:** 2025-03-21

**Authors:** Zhang Hongchen, Zhang Yijia, Xu Yubo, Zhong Jianguang, Sun Mouyuan, Li Jian, Li Jin

**Affiliations:** 1 The Department of Gastroenterology, Affiliated Hangzhou First People’s Hospital, School of Medicine, Westlake University, Hangzhou, 310003 People’s Republic of China; 2 The Fourth School of Clinical Medicine, Zhejiang Chinese Medical University Hangzhou First People’s Hospital, Hangzhou, 310003 People’s Republic of China; 3 The Department of Ophthalmology, Affiliated Hangzhou First People’s Hospital, School of Medicine, Westlake University, No. 261 HuanSha Road, Hangzhou, 310003 Zhejiang Province People’s Republic of China; 4 Stomatology Hospital, School of Stomatology, Zhejiang University School of Medicine, Zhejiang Provincial Clinical Research Center for Oral Diseases, Key Laboratory of Oral Biomedical Research of Zhejiang Province, Cancer Center of Zhejiang University, Engineering Research Center of Oral Biomaterials and Devices of Zhejiang Province, Hangzhou, 310000 People’s Republic of China

**Keywords:** Behçet’s disease, Probucol, Circulating metabolites, Mendelian randomization, Drug discovery, Genetics, Rheumatology, Medical research

## Abstract

Behçet’s disease (BD) is a chronic, recurrent condition for which effective preventive medications are currently lacking. This disease often disrupts lipid metabolism, adversely affecting vascular endothelial function. Exploring preventive strategies, such as lipid-lowering medications, is crucial. Probucol, known for its lipid-lowering properties, emerges as a promising candidate. By inhibiting the ATP-binding cassette transporter A1 (ABCA1), probucol is hypothesized to regulate circulating metabolites, potentially reducing the risk of BD. This study employs Mendelian randomization (MR) to evaluate probucol’s impact on BD and investigate its preventive potential through the modulation of circulating metabolites. For this MR study, we selected single nucleotide polymorphisms (SNPs) associated with probucol as instrumental variables and conducted a positive control analysis with SNPs linked to high-density lipoprotein (HDL) to validate our instrument selection. The study was structured in two steps: first, using probucol’s eQTLs to estimate its causal effect on circulating metabolites; second, using SNPs linked to these metabolites to assess their causal impact on Behcet’s disease risk. To ensure the robustness and validity of our findings, we employed several MR methods, including the Inverse Variance Weighted (IVW) approach, heterogeneity tests, and pleiotropy analysis. This study identified a total of 30 SNPs associated with BD, 7502 SNPs linked to circulating metabolites, and 1,049 SNPs associated with BD from circulating metabolites, all derived from ABCA1 expression quantitative trait loci (eQTL) data. Utilizing Mendelian randomization (MR) analysis, it was confirmed that probucol leads to a reduction in concentrations of cholesterol esters in HDL, consistent with findings from randomized drug trials (odds ratio [OR] = 0.932, 95% confidence interval [CI] 0.907–0.958, *P* < 0.001). Furthermore, the study demonstrated that probucol significantly decreased the risk of BD with an OR of 0.496 (95% CI 0.283–0.868, *P* = 0.014). Among 123 assessed circulating metabolites, thirty-six were found to be associated with probucol. Notably, probucol demonstrated a notable reduction in very large HDL particle concentrations (OR = 0.917, 95% CI 0.889–0.947, *P* < 0.001), contributing to approximately 10.407% of its overall impact on decreasing BD risk. This study establishes that probucol significantly lowers the risk of BD by reducing very large HDL particle concentrations. It provides a genetic basis for considering probucol as a potential therapeutic option for BD high risk individuals.

## Introduction

Behçet’s disease (BD) is a multisystem inflammatory disorder characterized by recurrent acute inflammation involving neutrophils and immune cells. Its pathogenesis involves genetic factors like HLA-B51 and environmental triggers. Inflammation in BD is driven by innate and adaptive immune responses, with hyperactive neutrophils releasing pro-inflammatory cytokines (IL-1β, TNF-α, IL-6)^1^. T helper cells (Th1 and Th17) further sustain inflammation by secreting cytokines (IFN-γ, IL-17)^2^. BD is more prevalent along the Silk Road than in Western countries, influenced by genetic and environmental factors^3,4^. Diagnosis is challenging due to its multisystemic nature and lack of definitive tests^4^. Clinically, BD presents with recurrent ulcers and eye and skin lesions, with possible severe complications^5^. Treatment involves symptom management with immunosuppressants and biologics, but standardized protocols are lacking, necessitating personalized approaches and emphasizing the need for more controlled trials ^6^.

Increasing evidence underscores the crucial role of metabolic disorders in the pathogenesis of BD, linking disturbances in metabolic homeostasis to the onset and exacerbation of this condition^7^. Recent studies reveal that specific circulating metabolites, including amino acids and phospholipids, disrupt metabolic processes and may serve as biomarkers for the disease^8,9^. Furthermore, observational studies suggest an inverse relationship between certain lipid components, such as total cholesterol and HDL-cholesterol, and BD risk, highlighting the significant impact of lipid metabolism. These insights into metabolic dysregulation offer potential avenues for preventing BD.

Probucol, an ATP-binding cassette transporter A1 (ABCA1) inhibitor, is known for its lipid-lowering effects by modulating cholesterol efflux and HDL formation. In the DrugBank database, probucol and ABCA1 are uniquely and explicitly associated as a drug and target with a defined pathological mechanism. Traditionally used for hyperlipidemia, probucol also shows potential in influencing inflammatory processes in BD^10^. By inhibiting ABCA1, probucol reduces cholesterol efflux from macrophages, potentially affecting inflammation pathways involved in BD^11,12^. Despite lowering HDL, probucol has antiatherogenic properties that may help manage BD’s vascular complications. These effects on lipid and inflammation pathways suggest probucol could be a novel therapeutic strategy for BD, warranting further investigation. Probucol, known for its lipid-lowering effects, is being explored for its potential impact on autoimmune diseases and metabolic profiles due to its antioxidant and anti-inflammatory properties. However, evidence linking probucol to improved autoimmune outcomes is limited, requiring rigorous clinical studies to confirm these effects and understand the mechanisms involved^13–16^. Additionally, while probucol reduces HDL cholesterol and alters lipid profiles, its broader impact on metabolites and cardiovascular disease progression is not well understood^17–19^. Therefore, extensive research is needed to fully explore the role of probucol in preventing BD in these areas.

Mendelian randomization (MR) uses genetic variants linked to an exposure to explore causal relationships with outcomes^20^. By employing these genetic variants as instruments, MR minimizes confounding and reverse causation, similar to randomized controlled trials. This is because genetic variants are randomly distributed at conception, akin to the randomization process in trials^21^.

In this study, we aim to explore the potential of probucol in preventing BD. We hypothesize that probucol may impact BD through changes in circulating metabolites. To test this hypothesis, we first employed a two-sample Mendelian randomization (MR) approach to investigate the association between probucol use and BD. Next, we conducted a two-step MR study to examine how probucol might influence BD via alterations in blood metabolites, particularly lipid profiles. This analytical approach allows us to evaluate the causal effects of probucol on these metabolites, providing valuable insights into the metabolic pathways that link probucol to BD prevention.

## Methods

### Study design

We accessed the expression quantitative trait loci (eQTLs) for ABCA1 related to probucol (https://go.drugbank.com/drugs/DB01599) under the identifier eqtl-a-ENSG00000165029. The eqtl-a-ENSG00000165029 dataset was obtained from the publicly available eQTLGen database (https://www.eqtlgen.org/phase1.html), which identified cis-eQTLs for 19,938 genes through a large-scale meta-analysis of blood samples from 31,684 Europeans across 37 cohorts. This dataset has been included in the IEU OpenGWAS project (https://gwas.mrcieu.ac.uk/) and was downloaded via this platform for our study. Additionally, GWAS data for 123 circulating metabolites and BD (ebi-a-GCST90018798) were retrieved from the same source. The data were accessed on February 20, 2024. The GCST90018798 dataset was obtained from the publicly available GWAS Catalog database (https://www.ebi.ac.uk/gwas/home), which includes data from 317,252 European (UK) and 172,044 East Asian (Japanese) participants. The dataset provides associations between participants’ SNPs and phenotypes obtained through whole-genome sequencing. Among these, the data from 317,252 European (UK) participants have been separately included in the IEU OpenGWAS project (https://gwas.mrcieu.ac.uk/). For this study, we downloaded the dataset through the IEU OpenGWAS platform, ensuring that all included participants were of European ancestry. Both the exposure eQTL data and the outcome data used in this study were derived from European populations, ensuring a relatively consistent genetic background. No ethical approval was required for the present study, for all data sources were based on publicly available summary-level data. All these studies were approved by the relevant institutional review committees.

**Fig. 1:**
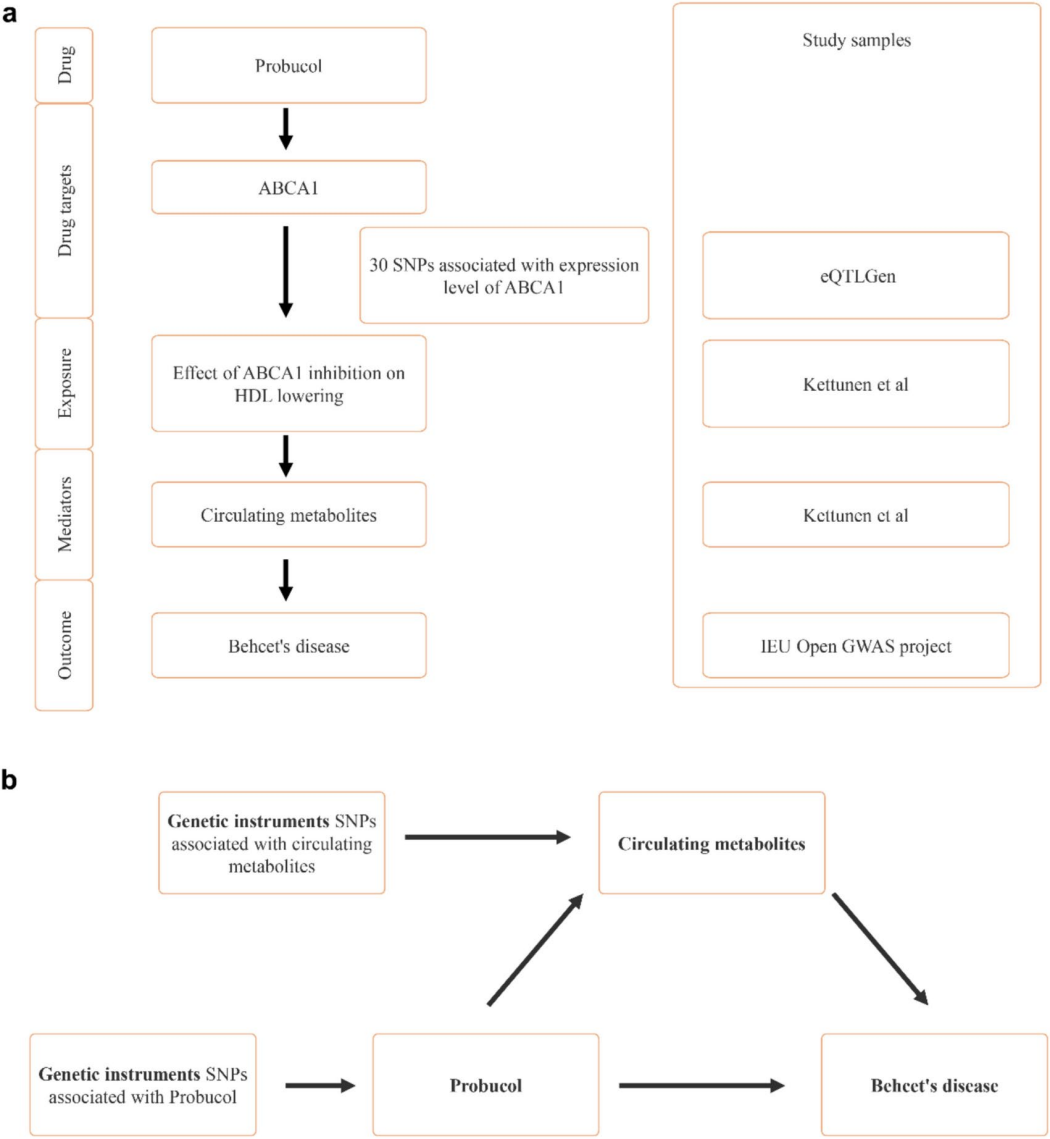
Study Design Overview. (**a**) A detailed flowchart illustrating the steps taken to assess the role of circulating metabolites as mediators in the effect of Probucol on Behçet’s disease. This includes the identification of genetic instruments and the evaluation of causal pathways. (**b**) A schematic representation of the two-step Mendelian randomization (MR) process, where the first step evaluates the association between Probucol (via ABCA1) and circulating metabolites, and the second step assesses the link between these metabolites and Behçet’s disease.

The present study employed a two-sample MR design (Fig. 1). In order to uphold the credibility of the inferred causal effects, the MR analyses were mandated to satisfy three fundamental assumptions^20^: (1) the genetic variants should exhibit a robust association with the exposure (relevance), (2) the genetic variants should be free from any potential confounding factors (exchangeability), and (3) the genetic variants should solely impact the outcome exclusively through the exposure (exclusion restriction). This study was reported in accordance with the Strengthening the Reporting of Observational Studies in Epidemiology Using Mendelian Randomization (STROBE-MR) guidelines^22^.

### Selection of Instrumental Variables for eQTLs of ABCA1

Relevant SNPs were extracted from the eQTL data of ABCA1, as well as GWAS summary data for BD, HDL, and circulating metabolites. SNPs strongly associated with eQTLs were selected based on the criteria of (*P* < 1 × 10^–5^), and weak instruments were excluded using an F-statistic threshold > 10. SNPs located within ± 100 kb of the cis-regulatory region of the target gene were retained. To ensure independence among SNPs and eliminate the influence of linkage disequilibrium (LD), an LD coefficient (r^2^ = 0.3), a region width of 100 kb, and a minor allele frequency (MAF) > 0.01 were applied. SNPs associated with confounders and outcomes (BD, HDL, and circulating metabolites) were excluded using PhenoScanner (*P* < 1 × 10^–5^). Additionally, palindromic SNPs were removed, and outliers were excluded using the MR-PRESSO method.

### Selection of instrumental variables for circulating metabolites

Relevant SNPs were extracted from the GWAS summary data for BD. To identify SNPs strongly associated with circulating metabolites, a selection threshold of (*P* < 1 × 10^–5^) was applied, and weak instruments were excluded using an F-statistic threshold > 10. To ensure independence among SNPs and eliminate the influence of linkage disequilibrium (LD), an LD coefficient (r^2^ = 0.3), an LD region width of 100 kb, and a minor allele frequency (MAF) > 0.01 were set. SNPs associated with confounders and outcomes (Behçet’s disease) were excluded using PhenoScanner (*P* < 1 × 10^–5^). Additionally, palindromic SNPs were removed, and outliers were excluded using the MR-PRESSO method.

### Statistical analyses

#### MR analysis to estimate the effects of probucol on Behçet’s disease

In this study, we applied two-sample univariable Mendelian randomization (UVMR) to estimate this effect, using inverse variance weighting (IVW) as the primary analytical method, with other methods used as supplementary analyses. When the number of SNPs was limited to one, the Wald ratio method was applied to evaluate the effect of the single SNP on the outcome; for other cases, the random-effects IVW method was employed. When horizontal pleiotropy among SNPs was present, MR-Egger results were referenced. MR-Egger regression, used as a supplementary analysis^23,24^, employs a weighted linear regression model that allows for bias in the relationship between instrumental variables and outcomes, effectively identifying and adjusting for pleiotropy.

#### Mediation MR analysis to investigate the potential link between probucol and Behçet’s disease through circulating metabolites

A two-step Mendelian randomization analysis was employed to assess the mediating role of circulating metabolites in the relationship between probucol and BD (Fig. 1b). The initial step involved utilizing UVMR to estimate the impact of ABCA1 inhibition on 123 circulating metabolites (β1). Subsequently, the metabolites that exhibited statistically significant associations with ABCA1 inhibition were evaluated for their effect on BD using UVMR in the second step. Following this, we conducted a screening for metabolites that exhibited a significant association with BD and subsequently utilized multivariable Mendelian randomization (MVMR) to adjust for potential confounders. The mediation proportion of each metabolite in the relationship between ABCA1 inhibition and BD was determined by multiplying β1 and β2 and dividing by the total effect of ABCA1 inhibition on BD. The 95% confidence intervals (CIs) for the mediation proportions were calculated utilizing the delta method^25^.

### Sensitivity analysis

Heterogeneity among SNPs was assessed using Cochran’s Q test, where a P < 0.05 indicated heterogeneity. I^2^ (I-squared) was also calculated as a measure of heterogeneity, representing the proportion of total variance attributable to heterogeneity, with values ranging from 0 to 100%. An I^2^ > 50% suggests a certain level of heterogeneity in the IVW results. The intercept term in MR-Egger and the MR-PRESSO method^26^ were used for pleiotropy analysis. MR-PRESSO identifies and excludes outlier genetic variants that may bias causal inference, reducing pleiotropy-induced bias and improving the accuracy of causal estimates. SNPs were considered to have no pleiotropy when the MR-Egger intercept was not significantly different from 0 (*P* > 0.05) and the MR-PRESSO *P* > 0.05. Sensitivity analysis was performed using the leave-one-out method, in which each SNP was sequentially removed, and the remaining SNPs were reanalyzed to observe the impact of each SNP on the results. All analyses were conducted using the TwoSampleMR package in R software (version 4.1.0), with a significance level of α = 0.05.

## Results

### Effect of ABCA1 inhibitor on HDL related metabolites and Behçet’s disease

After screening, in the ABCA1-BD MR analysis, 30 SNPs associated with BD were identified from the eQTL data of ABCA1 (Supplementary Table 1). In the two-step mediator MR analysis, the first step (ABCA1-circulating metabolites MR analysis) identified 7,502 SNPs associated with circulating metabolites as instrumental variables (Supplementary Table 2). In the second step (circulating metabolites- BD MR analysis), 1,049 SNPs associated with BD were identified from the GWAS data for circulating metabolites (Supplementary Table 3). For the positive control analysis of ABCA1-HDL MR analysis, 915 SNPs associated with HDL were identified (Supplementary Table 4). The results of the ABCA1 instrument validation analysis were largely consistent with those of the meta-analysis of drug randomized trials (i.e., the direction of effect)^27^. For example, the IVW results indicated that ABCA1 inhibitor’s drug action was associated with lower concentration of cholesterol esters in large HDL (OR = 0.932, 95% CI 0.907–0.958, *P* < 0.001, Table 1). The IVW results showed no heterogeneity between ABCA1 inhibitors and HDL related SNPs (I^2^ = 0%, Cochran’s Q = 50.430, *P* = 0.806, Table 1). The MR-Egger analysis revealed that the intercept was not significantly different from zero (*P* = 0.893), and MR-PRESSO did not detect significant horizontal pleiotropy (*P* = 0.832). Therefore, we concluded that the SNPs do not exhibit horizontal pleiotropy. The leave-one-out analysis confirmed the robustness of our findings, while symmetrical funnel plots indicated no publication bias. Scatter plots showed a clear linear association between ABCA1 genetic variants and lower concentration of HDL, validating our results (Supplementary Fig. 1).

**Table 1:** Mendelian Randomization estimates of Probucol’s effect on concentration of cholesterol esters in large HDL.

Exposure	Outcome	Nsnp	Method	MR		Heterogeneity			Horizontal pleiotropy			
				Drug_OR (95%CI)	*P* value	I^2^ (%)	Cochran’s Q	*P*-value	Egger intercept	SE	*P*-value	MR-PRESSO global test p
ABCA1 inhibitor	Cholesterol esters in large HDL	61	MR Egger	0.9274 (0.8574–1.0031)	0.0646	0	50.4119	0.7795	− 0.0007	0.0052	0.8926	0.8317
			Weighted median	0.9312 (0.8949–0.9691)	< 0.001	–	–	–	–	–	–	0.8317
			Inverse variance weighted	0.9321 (0.9069–0.958)	< 0.001	0	50.4303	0.8060	–	–	–	0.8317
			Simple mode	0.9192 (0.8533–0.9903)	0.0305	–	–	–	–	–	–	0.8317
			Weighted mode	0.9228 (0.868–0.981)	0.0125	–	–	–	–	–	–	0.8317

IVW, inverse–variance weighted; CI, confidence interval; HDL, high-density lipoprotein; VLDL, Very-Low-Density Lipoprotein; LDL, Low-Density Lipoprotein; OR, odds ratio; MR-PRESSO, Mendelian Randomization Pleiotropy RESidual Sum and Outlier.

Based on the dataset for BD (ebi-a-GCST90018798), we performed MR analysis using the eQTLs of the Probucol target gene ABCA1 as instrumental variables to examine the association between Probucol and BD. The IVW results indicated that ABCA1 inhibitor was associated with a reduced risk of BD (OR = 0.496, 95% CI 0.283–0.868,* P* = 0.014, Table 2). There was no heterogeneity between the eQTLs associated with ABCA1 inhibitor and BD (I^2^ = 0%, Cochran’s Q = 23.513,* P* = 0.753, Table 2). The MR-Egger analysis showed that the intercept was not significantly different from zero (*P* = 0.875), and MR-PRESSO did not detect significant horizontal pleiotropy (*P* = 0.761). Thus, we concluded that the SNPs do not exhibit horizontal pleiotropy. The leave-one-out analysis confirmed the robustness of our findings, the funnel plot showed no significant publication bias, and the scatter plot indicated a clear linear relationship, reinforcing the association between ABCA1 variants and reduced BD risk (Supplementary Fig. 2).

**Table 2:** Mendelian Randomization estimates of Probucol’s effect on Behçet’s disease.

Exposure	Outcome	Nsnp	Method	MR		Heterogeneity			Horizontal pleiotropy			
				Drug_OR (95%CI)	*P* value	I^2^ (%)	Cochran’s Q	*P*-value	Egger intercept	SE	*P*-value	MR-PRESSO global test p
ABCA1 inhibitor	Behcet’s disease	30	MR Egger	0.5648 (0.1006–3.1702)	0.5216	0	23.4880	0.7083	0.0172	0.1097	0.8764	0.7610
			Weighted median	0.4233 (0.1867–0.96)	0.0396	–	–	–	–	–	–	0.7610
			Inverse variance weighted	0.4957 (0.283–0.8681)	0.0141	0	23.5126	0.7526	–	–	–	0.7610
			Simple mode	0.4539 (0.1285–1.6032)	0.2297	–	–	–	–	–	–	0.7610
			Weighted mode	0.4207 (0.1707–1.0369)	0.0700	–	–	–	–	–	–	0.7610

CI, confidence interval; OR, odds ratio; MR-PRESSO, Mendelian Randomization Pleiotropy RESidual Sum and Outlier.

### Mediation MR of ABCA1 inhibitor, circulating metabolites, and Behçet’s disease

Circulating metabolites might mediate the effect of Probucol in reducing the incidence of BD. To investigate this mediation pathway, we performed multivariable adjustments. In the first step, we used the eQTLs of ABCA1 inhibitor to estimate its causal effect on circulating metabolites. The IVW method showed statistically significant causal relationships between ABCA1 inhibitor and 36 circulating metabolites. ABCA1 inhibitor elevated six metabolites and reduced twenty-nine. Notably, ABCA1 inhibitor decreased the concentration of the circulating metabolite “Concentration of very large HDL particles” (OR = 0.917, 95% CI: 0.889–0.947,* P* < 0.001, Supplementary Table 5). The IVW results indicated no heterogeneity between the SNPs associated with ABCA1 inhibitor and the “Concentration of very large HDL particles” (I^2^ = 24%, Cochran’s Q = 79.013, *P* = 0.051, Supplementary Table 5). The MR-Egger analysis showed that the intercept was not significantly different from zero (*P* = 0.642), and MR-PRESSO did not detect significant horizontal pleiotropy (*P* = 0.066). Therefore, we concluded that the SNPs do not exhibit horizontal pleiotropy. The leave-one-out analysis confirmed the robustness of our findings, and symmetrical funnel plots indicated no publication bias. Additionally, scatter plots demonstrated a clear linear association between ABCA1 genetic variants and these metabolites, validating our results (Supplementary Fig. 3).

**Fig. 2:**
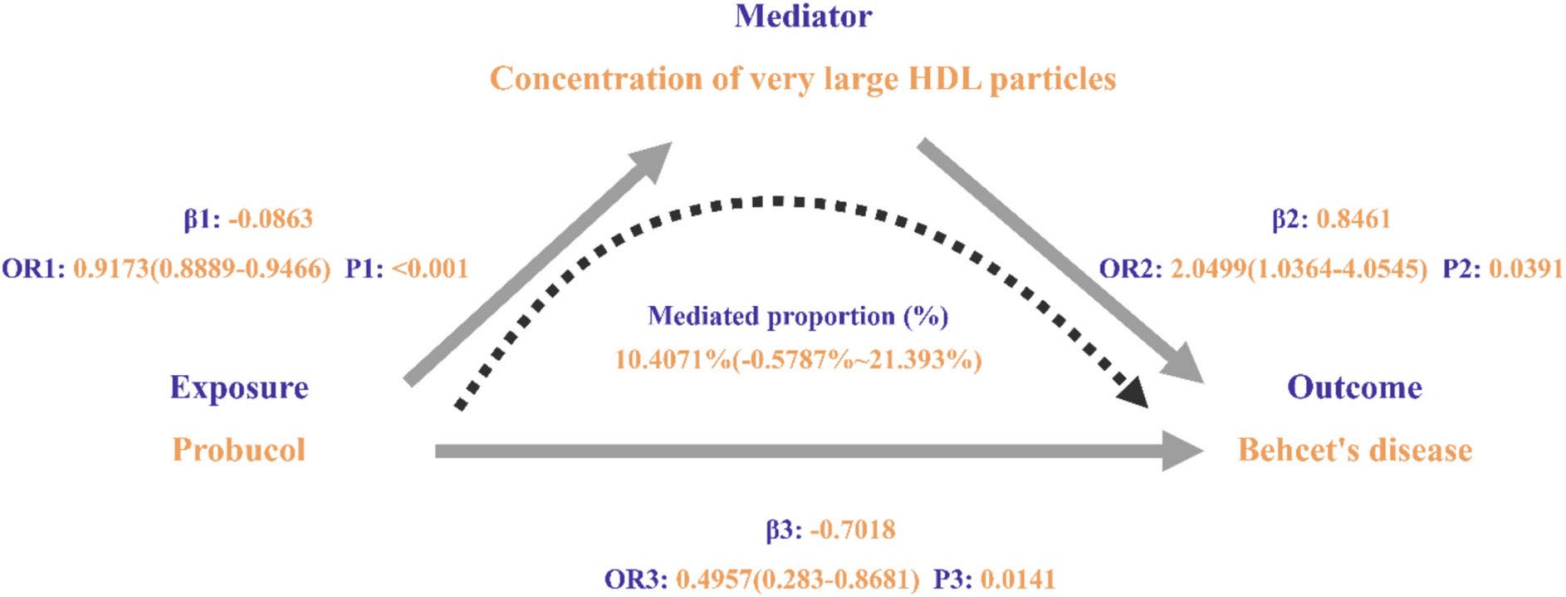
Mediation Analysis of Probucol’s Impact on Behçet’s Disease. This figure demonstrates the results of the two-step Mendelian randomization analysis, which evaluates the potential mediating effects of circulating metabolites on the causal pathway between Probucol and Behçet’s disease. The analysis highlights the contribution of each intermediary to the overall effect.

In the second step, we evaluated the causal effect of the 36 circulating metabolites, which showed statistically significant causal relationships with ABCA1 inhibitor in the first step, on the risk of BD using their associated SNPs. The IVW method revealed a statistically significant causal relationship between the “Concentration of very large HDL particles” and BD. The “Concentration of very large HDL particles” was identified as a risk factor for BD (OR = 2.050, 95% CI 1.036–4.055, *P* = 0.039, Table 3, Fig. 2). The IVW results indicated no heterogeneity between the SNPs associated with the “Concentration of very large HDL particles” and BD (I^2^ = 0%, Cochran’s Q = 27.975, *P* = 0.519, Table 3, Fig. 2). The MR-Egger analysis showed that the intercept was not significantly different from zero (*P* = 0.270), and MR-PRESSO did not detect significant horizontal pleiotropy (*P* = 0.519). Therefore, we concluded that the SNPs do not exhibit horizontal pleiotropy. The leave-one-out analysis confirmed the robustness of our findings, and symmetrical funnel plots indicated no publication bias. Additionally, scatter plots demonstrated a clear linear association (Supplementary Fig. 4).

**Table 3:** Mendelian randomization analysis results of the causal relationship between circulating metabolites and Behçet’s disease.

Exposure	Outcome	Nsnp	Method	MR			Heterogeneity			Horizontal pleiotropy		
				OR (95%CI)	*P* value	I^2^ (%)	Cochran’s Q	*P*-value	Egger intercept	SE	*P*-value	MR-PRESSO global test p
Concentration of very large HDL particles	Behcet’s disease	30	MR Egger	0.918 (0.1938–4.3491)	0.9149	0	26.7061	0.5343	0.0934	0.0830	0.2696	0.5190
			Weighted median	1.853 (0.6234–5.5078)	0.2671	–	–	–	–	–	–	0.5190
			Inverse variance weighted	2.0499 (1.0364–4.0545)	0.0391	0	27.9745	0.5193	–	–	–	0.5190
			Simple mode	2.2696 (0.4014–12.8322)	0.3614	–	–	–	–	–	–	0.5190
			Weighted mode	1.8064 (0.6351–5.1379)	0.2767	–	–	–	–	–	–	0.5190

CI, confidence interval; HDL, high-density lipoprotein; OR, odds ratio; MR-PRESSO, Mendelian Randomization Pleiotropy RESidual Sum and Outlier.

**Fig. 3:**
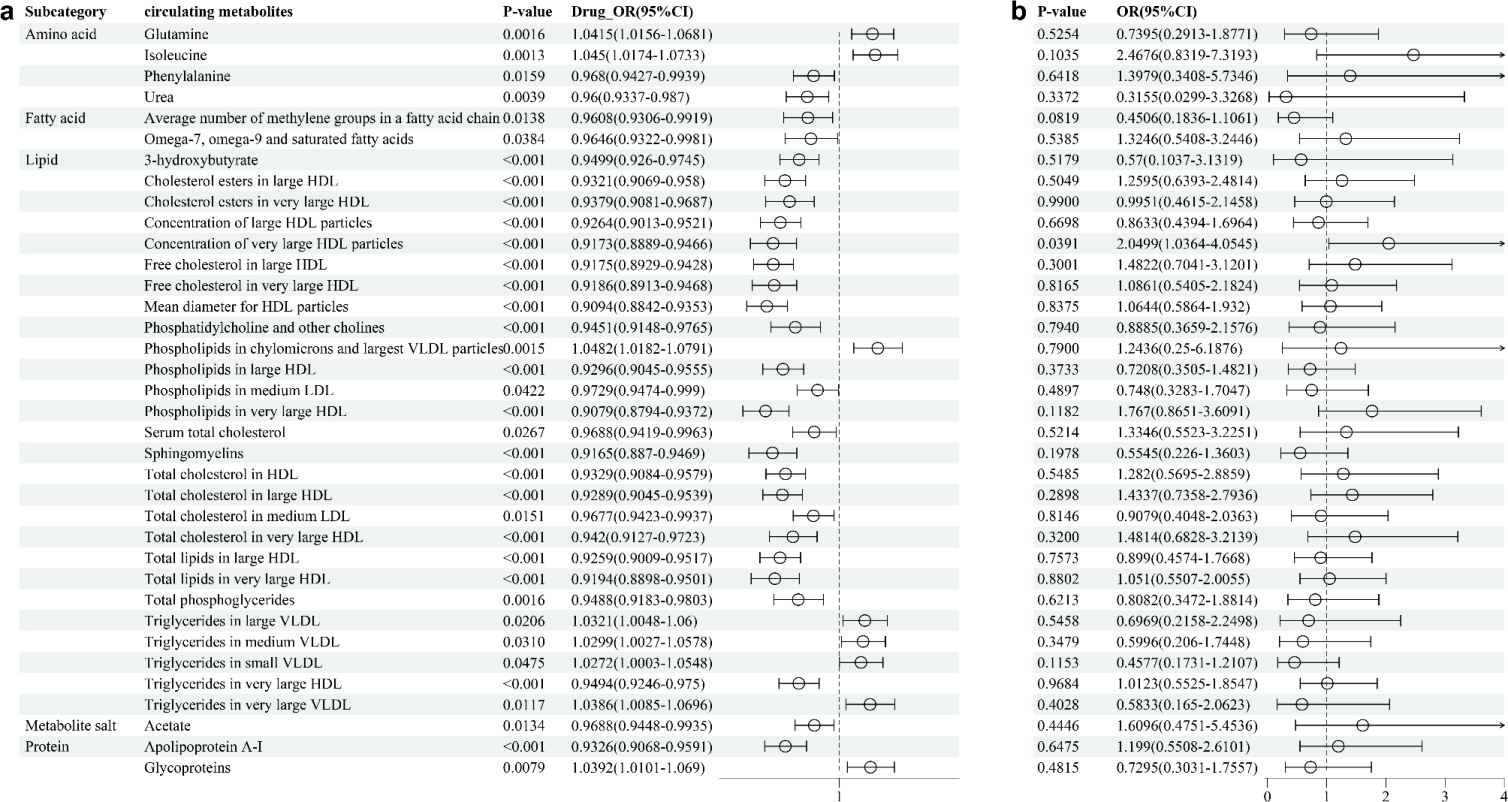
Forest Plot of Probucol’s Effects on Circulating Metabolites and Behçet’s Disease. The forest plot displays the estimated causal effects of Probucol on individual circulating metabolites (step one) and the subsequent effects of these metabolites on Behçet’s disease (step two). Each point estimate is accompanied by its confidence interval, providing a visual summary of the mediation effects.

Combining the results from the first two steps, we found that the circulating metabolite “Concentration of very large HDL particles” has a significant causal relationship with both ABCA1 inhibitor and BD (Fig. 3). Under the mediation condition of circulating metabolites, we calculated the mediating effect of the “Concentration of very large HDL particles.” The total effect of ABCA1 inhibitor on BD was estimated to be − 0.702. The mediating effect of the “Concentration of very large HDL particles” on the association between ABCA1 inhibitor and BD was − 0.073, accounting for 10.407% of the total effect (95% CI − 0.579–21.393%) (Table 4, Fig. 3). After adjusting for potential confounders (circulating metabolites) using MVMR analysis, the causal effect of ABCA1 inhibitor on BD was no longer significant (*P* > 0.05). This indicates that the previously observed causal relationship is related to the circulating metabolite “Concentration of very large HDL particles” (Table 5).

**Table 4:** The mediating effect of circulating metabolites on the relationship between Probucol and Behçet’s disease.

Mediator	Mediating_effect	Direct_effect	Total_effect	Proportion mediated (%)
Concentration of very large HDL particles	− 0.0730	− 0.6288	− 0.7018	10.4071 (− 0.5787 ~ 21.393)

**Table 5:** MVMR analysis of Probucol’s effect on Behçet’s disease after multivariable adjustment.

Exposure	Adjustment	Outcome	β	SE	*P*
ABCA1 inhibitor	Concentration of very large HDL particles	Behcet’s disease	− 0.0477	0.5269	0.9279

## Discussion

In this study, we explored for the first time the potential of probucol in preventing BD, specifically examining how circulating metabolites might mediate this association. We identified several key findings. First, probucol use was linked to a reduced risk of BD. Second, through a two-step MR analysis, we discovered that probucol has a statistically significant causal relationship with 36 circulating metabolites, notably decreasing the concentration of very large HDL particles. Very large HDL particles were found to have a significant causal relationship with both probucol use and BD risk, suggesting they may mediate the protective effects of probucol against BD.

In this study, we identified for the first time that probucol significantly reduces the risk of BD. This finding has important clinical implications, suggesting a new therapeutic option for reducing the risk of BD in high-risk populations. Previously, probucol had been studied for various health conditions, including its potential to modulate autoimmune responses by affecting circulating metabolites^28,29^. However, direct evidence linking probucol specifically to autoimmune diseases, particularly BD, was limited. Our use of robust genetic instruments and extensive GWAS data to demonstrate probucol’s protective effects on BD not only aligns with existing literature on its immunomodulatory properties but also fills a significant research gap, providing novel insights into its potential role in autoimmune disease management.

Using Mendelian analysis for the first time, we discovered that probucol influences circulating metabolites, highlighting its significant impact on metabolic health and potential for managing vascular and metabolic diseases. Our study utilized genetic variants of probucol and the largest GWAS on circulating metabolites, focusing on blood lipids and lipoprotein particle subfractions. The results showed significant changes in amino acids, fatty acids, and specific lipoprotein particles. Notably, probucol reduced HDL particle size and altered cholesterol ester concentrations in large and very large HDL particles. It also affected omega-7, omega-9, and saturated fatty acids, and phospholipids in chylomicrons and the largest VLDL particles. These findings are consistent with previous studies showing probucol’s effects on blood lipids and lipoprotein particle sizes, particularly HDL and LDL particles, highlighting its distinctive impact on lipid metabolism and vascular health^30–32^.

This study highlights a novel discovery that probucol can significantly reduce concentration of very large HDL particles. This finding is significant as it provides initial genetic evidence linking these HDL particles with BD and suggests a potential protective effect of probucol on BD. Previous research indicates that while smaller HDL particles protect against cardiovascular diseases, larger HDL particles may increase the risk of vascular and autoimmune diseases due to their different roles in cholesterol metabolism and inflammatory processes^33–35^. These larger HDL particles can exacerbate inflammatory and thrombotic activities, crucial in the progression of conditions like Systemic Lupus Erythematosus and Rheumatoid Arthritis, by interacting with immune responses and pathways involved in endothelial function and coagulation^36^. Our findings imply that probucol’s ability to reduce very large HDL particles could mediate its protective effect on BD. This mechanism, suggested by our genetic data, aligns with existing literature on HDL particles’ role in inflammation and autoimmunity, though further experimental studies are necessary to confirm causality.

This study’s finding that probucol significantly reduces the risk of BD offers promising clinical applications for both prevention and treatment. Given the limited effective treatments available for BD, this discovery provides a valuable new option^37^. Probucol’s ability to lower the risk of developing BD suggests its potential as a preventive therapy, particularly for high-risk populations. Early intervention with probucol could delay or prevent BD onset, reducing disease incidence and complications. For patients with BD, probucol’s effects present a novel therapeutic approach, which can help manage symptoms and decrease the frequency and severity of flares, enhancing patient outcomes and quality of life. Further studies are needed to confirm these findings and understand probucol’s protective mechanisms in BD. Clinical trials focused on its efficacy and safety are essential for translating these results into practice. Clinicians might evaluate its use alongside existing therapies to assess combined effects on symptom control and disease progression.

This study represents the first application of MR to examine the links between probucol, circulating metabolites, and BD within the general population. However, it faces several limitations. Firstly, the genetic variants used mimic the lifelong effects of ABCA1 inhibition by probucol, which may not accurately represent the drug’s short-term effects. To address this, future research could incorporate short-term clinical trials to complement MR findings. Secondly, the lack of stratification by subtypes in the original GWAS data precluded stratified analyses. Future studies should stratify analyses when specific datasets become available to capture subtype-specific effects. Thirdly, environmental modifiers of genetic risk for metabolic diseases might bias the estimated effects. Incorporating environmental data and performing multi-environment analyses could help mitigate this issue. Fourthly, the study focuses solely on the predicted effects related to specific drug targets, not accounting for possible off-target effects. Future research should explore potential off-target effects using comprehensive drug profiling methods. Additionally, while various sensitivity analyses aimed to mitigate horizontal pleiotropy, it cannot be entirely ruled out. Employing advanced statistical techniques and cross-validating with independent datasets could help address this limitation. Finally, as our sample was restricted to individuals of European ancestry, our results may not be generalizable to other ethnic groups. Differences in genetic architecture and allele frequencies across populations may lead to variations in associations. Expanding studies to include diverse populations is essential to validate these findings and explore potential ethnic-specific effects.

## Conclusions

In summary, the findings of this study support the link between probucol and circulating metabolites in Behçet’s disease, indicating that the protective effect of probucol is mediated by large HDL particles. These findings imply that probucol may have potential as a prophylactic treatment for individuals at high risk for Behçet’s disease, thereby informing future research efforts aimed at elucidating its mechanisms of action and evaluating its clinical efficacy.

## Supplementary Information

Supplementary Information 1.

Supplementary Information 2.

Supplementary Information 3.

Supplementary Information 4.

Supplementary Information 5.

Supplementary Information 6.

Supplementary Information 7.

Supplementary Information 8.

Supplementary Information 9.

## Data Availability

The expression quantitative trait loci (eQTLs) for ABCA1, associated with Probucol (https://go.drugbank.com/drugs/DB01599), were retrieved from the publicly available IEU OpenGWAS project database (https://gwas.mrcieu.ac.uk/). The eQTLs dataset (identifier eqtl-a-ENSG00000165029) was downloaded from the platform, which includes results from a large-scale meta-analysis of blood samples from 31,684 European participants across 37 cohorts. Additionally, GWAS data for 123 circulating metabolites and Behçet’s disease (identifier ebi-a-GCST90018798) were also accessed via the IEU OpenGWAS project. The GWAS Catalog (https://www.ebi.ac.uk/gwas/home) provided the dataset, which includes data from 317,252 European (UK) and 172,044 East Asian (Japanese) participants, and was downloaded for use in this study.

## Abbreviations

BD: Behçet’s disease
GWAS: Genome-wide association stud
HDL: High-density lipoprotein
IVW: Inverse variant weight
LDL: Low-density lipoprotein
MR: Mendelian randomization
MVMR: Multivariable Mendelian randomization
ABCA1: ATP-binding cassette transporter A1
UVMR: Univariable Mendelian randomization
MR-PRESSO: Mendelian Randomization Pleiotropy RESidual Sum and Outlier

## References

[1] Yüksel, Ş et al. Novel NLRP3/cryopyrin mutations and pro-inflammatory cytokine profiles in Behçet’s syndrome patients. *Int. Immunol.* **26**(2), 71–81. 10.1093/intimm/dxt046 (2014). 24135410 10.1093/intimm/dxt046

[2] Hamzaoui, K. et al. Expression of Th-17 and RORγt mRNA in Behçet’s disease. *Med. Sci. Monit. Int. Med. J. Exp. Clin. Res.* **17**(4), 227–234 (2011). 10.12659/MSM.881720 PMC3539514 21455110

[3] Mohammad, A. J., Mandl, T., Sturfelt, G. K. & Segelmark, M. Incidence, prevalence and clinical characteristics of Behcet’s disease in southern Sweden. *Rheumatology* **52**(2), 304–310 (2013). 23012468 10.1093/rheumatology/kes249

[4] Salvarani, C. et al. Epidemiology and clinical course of Behçet’s disease in the Reggio Emilia area of Northern Italy: A seventeen-year population-based study. *Arthritis Rheumat.* **57**(1), 171–178. 10.1002/art.22500 (2007). 17266063 10.1002/art.22500

[5] Chen, J.-J. & Yao, X. A contemporary review of Behcet’s syndrome. *Clin. Rev. Allerg. Immunol.* **61**, 363–376 (2021). 10.1007/s12016-021-08864-3 34076835

[6] Rokutanda, R., Kishimoto, M. & Okada, M. Update on the diagnosis and management of Behçet’s disease. *Open Access Rheumatol. Res. Rev.* **7**, 1–8. 10.2147/oarrr.s46644 (2015). 10.2147/OARRR.S46644 PMC5045120 27790039

[7] Yazici, Y. et al. Behçet syndrome. *Nat. Rev. Dis. Prim.* **7**(1), 67. 10.1038/s41572-021-00301-1 (2021). 34531393 10.1038/s41572-021-00301-1

[8] Kim, Y. H. et al. Risk for Behçet’s disease gauged via high-density lipoprotein cholesterol: A nationwide population-based study in Korea. *Sci. Rep.* **12**(1), 12735. 10.1038/s41598-022-17096-0 (2022). 35882901 10.1038/s41598-022-17096-0 PMC9325767

[9] Yücel, Ç. et al. Comparative metabolomic profiles of vascular involvement in Behçet’s disease. *Eur. J. Rheumatol.* **10**(4), 130–135. 10.5152/eurjrheum.2023.23062 (2023). 37850605 10.5152/eurjrheum.2023.23062 PMC10765176

[10] Favari, E. et al. Probucol inhibits ABCA1-mediated cellular lipid efflux. *Arterioscler. Thromb. Vasc. Biol.* **24**, 2345–2350 (2004). 15514211 10.1161/01.ATV.0000148706.15947.8a

[11] Wu, C.-a, Tsujita, M., Hayashi, M. & Yokoyama, S. Probucol inactivates ABCA1 in the plasma membrane with respect to its mediation of apolipoprotein binding and high density lipoprotein assembly and to its proteolytic degradation. *J. Biol. Chem.* **279**, 30168–30174 (2004). 15140889 10.1074/jbc.M403765200

[12] Arakawa, R. et al. Pharmacological inhibition of ABCA1 degradation increases HDL biogenesis and exhibits antiatherogenesis. *J. Lipid Res.* **50**, 2299–2305 (2009). 19458386 10.1194/jlr.M900122-JLR200 PMC2759836

[13] Al-Majed, A. A. Probucol attenuates oxidative stress, energy starvation, and nitric acid production following transient forebrain ischemia in the rat hippocampus. *Oxid. Med. Cell. Longev.* **2011**, 471590. 10.1155/2011/471590 (2011). 21904644 10.1155/2011/471590 PMC3166564

[14] Sharif, A. et al. The therapeutic potential of probucol and probucol analogues in neurodegenerative diseases. *Transl. Neurodegen.* **13**(1), 6. 10.1186/s40035-024-00398-w (2024). 10.1186/s40035-024-00398-w PMC10802046 38247000

[15] Han, G., Zhang, Y. & Li, H. The combination treatment of curcumin and probucol protects chondrocytes from TNF-α induced inflammation by enhancing autophagy and reducing apoptosis via the PI3K-Akt-mTOR pathway. *Oxid. Med. Cell. Longev.* **2021**, 5558066. 10.1155/2021/5558066 (2021). 34257809 10.1155/2021/5558066 PMC8249126

[16] Zucoloto, A. Z. et al. Probucol attenuates lipopolysaccharide-induced leukocyte recruitment and inflammatory hyperalgesia: Effect on NF-кB activation and cytokine production. *Eur. J. Pharmacol.* **809**, 52–63. 10.1016/j.ejphar.2017.05.016 (2017). 10.1016/j.ejphar.2017.05.016 28501577

[17] Nomura, S. et al. Probucol and ticlopidine: Effect on platelet and monocyte activation markers in hyperlipidemic patients with and without type 2 diabetes. *Atherosclerosis* **174**(2), 329–335 (2004). 15136063 10.1016/j.atherosclerosis.2004.01.027

[18] Bird, D. A. et al. Effect of probucol on LDL oxidation and atherosclerosis in LDL receptor-deficient mice. *J. Lipid Res.* **39**(5), 1079–1090 (1998). 9610776

[19] Chen, Y. et al. Probucol protects circulating endothelial progenitor cells from ambient PM2.5 damage via inhibition of reactive oxygen species and inflammatory cytokine production in vivo. *Exp. Therap. Med.* **16**, 4322–4328 (2018). 30542381 10.3892/etm.2018.6791 PMC6257429

[20] Emdin, C. A., Khera, A. V. & Kathiresan, S. Mendelian randomization. *JAMA* **318**(19), 1925–1926. 10.1001/jama.2017.17219 (2017). 29164242 10.1001/jama.2017.17219

[21] Burgess, S., Timpson, N. J., Ebrahim, S. & Davey, S. G. Mendelian randomization: Where are we now and where are we going?. *Int. J. Epidemiol.* **44**(2), 379–388. 10.1093/ije/dyv108 (2015). 26085674 10.1093/ije/dyv108

[22] Skrivankova, V. W. et al. Strengthening the reporting of observational studies in epidemiology using Mendelian randomization: The STROBE-MR statement. *Jama* **326**(16), 1614–1621. 10.1001/jama.2021.18236 (2021). 34698778 10.1001/jama.2021.18236

[23] Lin, Z., Deng, Y. & Pan, W. Combining the strengths of inverse-variance weighting and Egger regression in Mendelian randomization using a mixture of regressions model. *PLoS Genet.* **17**(11), e1009922. 10.1371/journal.pgen.1009922 (2021). 34793444 10.1371/journal.pgen.1009922 PMC8639093

[24] Burgess, S. et al. Guidelines for performing Mendelian randomization investigations: Update for summer. *Wellcome Open Res.* **4**, 186. 10.12688/wellcomeopenres.15555.3 (2019). 32760811 10.12688/wellcomeopenres.15555.1 PMC7384151

[25] MacKinnon, D. P., Lockwood, C. M., Hoffman, J. M., West, S. G. & Sheets, V. A comparison of methods to test mediation and other intervening variable effects. *Psychol. Methods* **7**(1), 83–104. 10.1037/1082-989x.7.1.83 (2002). 11928892 10.1037/1082-989x.7.1.83 PMC2819363

[26] Verbanck, M., Chen, C. Y., Neale, B. & Do, R. Detection of widespread horizontal pleiotropy in causal relationships inferred from Mendelian randomization between complex traits and diseases. *Nat. Genet.* **50**(5), 693–698. 10.1038/s41588-018-0099-7 (2018). 29686387 10.1038/s41588-018-0099-7 PMC6083837

[27] Liu, J. et al. Effects of probucol on restenosis after percutaneous coronary intervention: A systematic review and meta-analysis. *PloS One* **10**(4), e0124021. 10.1371/journal.pone.0124021 (2015). 25898372 10.1371/journal.pone.0124021 PMC4405356

[28] Drash, A. L., Rudert, W. A., Borquaye, S., Wang, R. & Lieberman, I. Effect of probucol on development of diabetes mellitus in BB rats. *Am. J. Cardiol.* **62**(3), 27B-30B (1988). 3394650 10.1016/s0002-9149(88)80047-x

[29] Denton, C. P. et al. Probucol improves symptoms and reduces lipoprotein oxidation susceptibility in patients with Raynaud’s phenomenon. *Rheumatology (Oxford, England)* **38**(4), 309–315. 10.1093/rheumatology/38.4.309 (1999). 10378706 10.1093/rheumatology/38.4.309

[30] Brown, H. B. & deWolfe, V. G. The additive effect of probucol on diet in hyperlipidemia. *Clin. Pharmacol. Therap.* **16**, 44 (1974). 4602055 10.1002/cpt1974161part144

[31] Inagaki, M. et al. Effect of probucol on antioxidant properties of HDL in patients with heterozygous familial hypercholesterolemia. *J. Atheroscler. Thromb.* **19**(7), 643–656 (2012). 22785024 10.5551/jat.12807

[32] Elinder, L. S. et al. Probucol treatment decreases serum concentrations of diet-derived antioxidants. *Arterioscler. Thromb. Vasc. Biol.* **15**(8), 1057–1063 (1995). 7627696 10.1161/01.atv.15.8.1057

[33] Kontush, A. HDL particle number and size as predictors of cardiovascular disease. *Front. Pharmacol.*10.3389/fphar.2015.00218 (2015). 26500551 10.3389/fphar.2015.00218 PMC4593254

[34] Chang, C.-K. et al. The sizes and composition of HDL-cholesterol are significantly associated with inflammation in rheumatoid arthritis patients. *Int. J. Mol. Sci.* **24**, 10645 (2023). 37445823 10.3390/ijms241310645 PMC10341560

[35] Camont, L. et al. Small, dense high-density lipoprotein-3 particles are enriched in negatively charged phospholipids: Relevance to cellular cholesterol efflux, antioxidative, antithrombotic, anti-inflammatory, and antiapoptotic functionalities. *Arterioscler. Thrombosis Vasc. Biol.* **33**, 2715–2723 (2013). 10.1161/ATVBAHA.113.301468 24092747

[36] Hahn, B. H., Grossman, J. M., Ansell, B. J., Skaggs, B. J. & McMahon, M. Altered lipoprotein metabolism in chronic inflammatory states: Proinflammatory high-density lipoprotein and accelerated atherosclerosis in systemic lupus erythematosus and rheumatoid arthritis. *Arthritis Res. Ther.* **10**, 213 (2008). 18828865 10.1186/ar2471 PMC2575639

[37] Moriano Morales, C. et al. SER recommendations on treatment of refractory Behçet’s syndrome. *Reumatol. Clin.* **20**(4), 204–217. 10.1016/j.reumae.2023.12.006 (2024). 38614885 10.1016/j.reumae.2023.12.006

