# Supplementary figures and images for: Mendelian randomization reveals probucol’s preventive role in Behçet’s disease via circulating metabolites (part 1 of 2)
Source: Sci Rep. 2025 Mar 21;15:9722. doi: 10.1038/s41598-025-93644-8 (PMC11928609; doi:10.1038/s41598-025-93644-8)

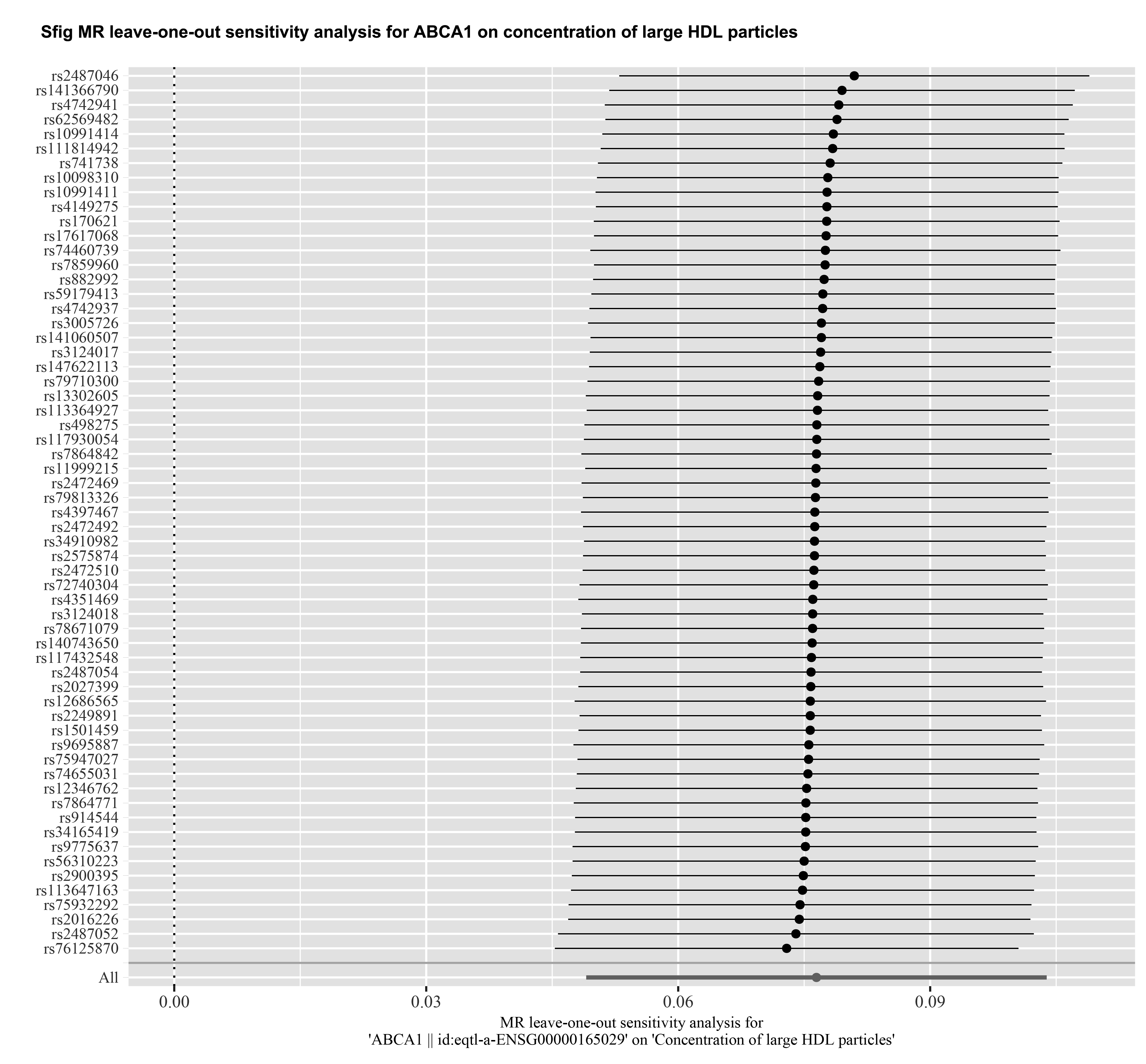

Supplement: Supplementary file 1 — Supplementary Information 1. [file 41598_2025_93644_MOESM1_ESM.zip › leave-one-out analysis/Sfig MR leave-one-out sensitivity analysis for ABCA1 on concentration of large HDL particles.tif]

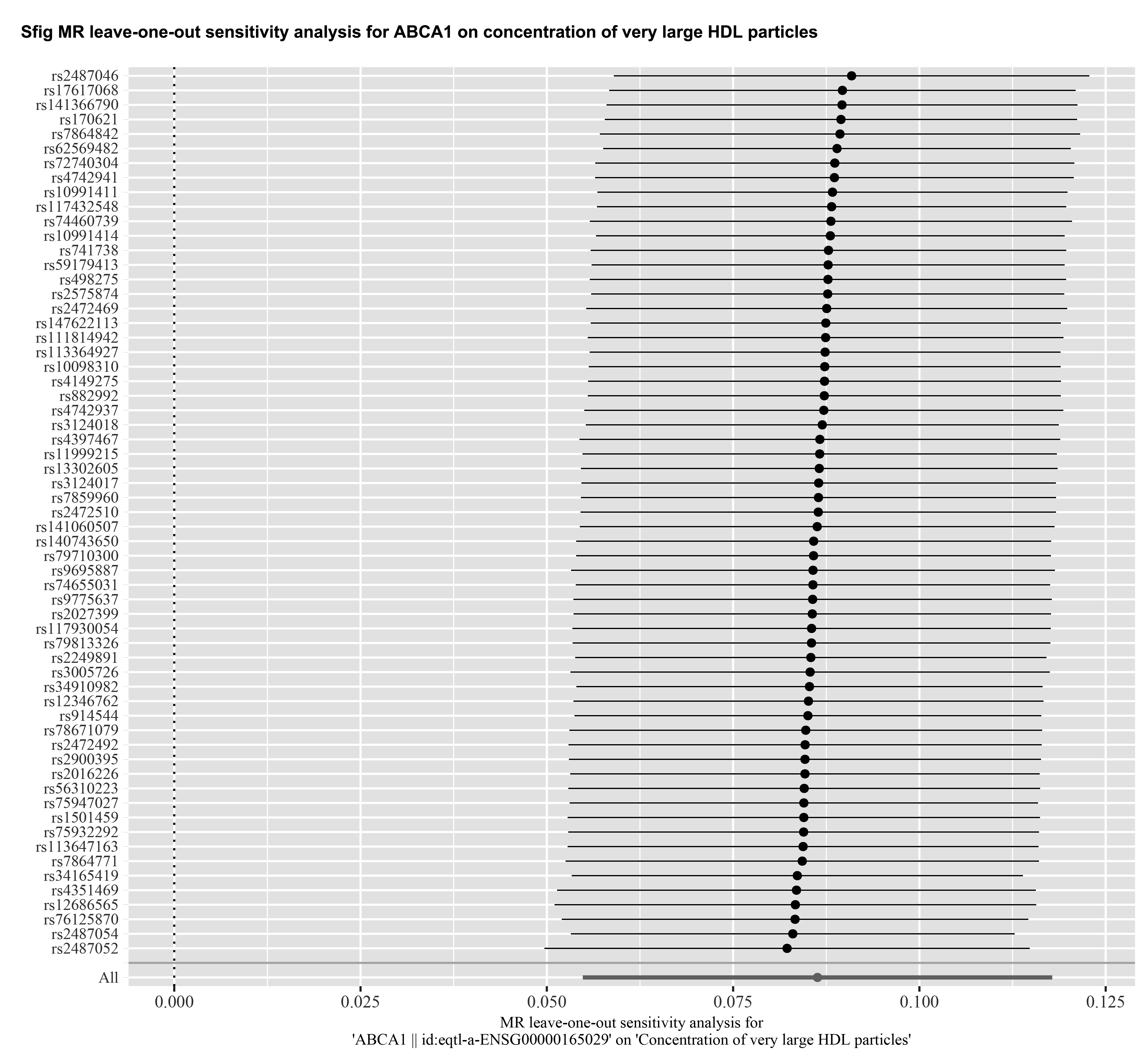

Supplement: Supplementary file 1 — Supplementary Information 1. [file 41598_2025_93644_MOESM1_ESM.zip › leave-one-out analysis/Sfig MR leave-one-out sensitivity analysis for ABCA1 on concentration of very large HDL particles.tif]

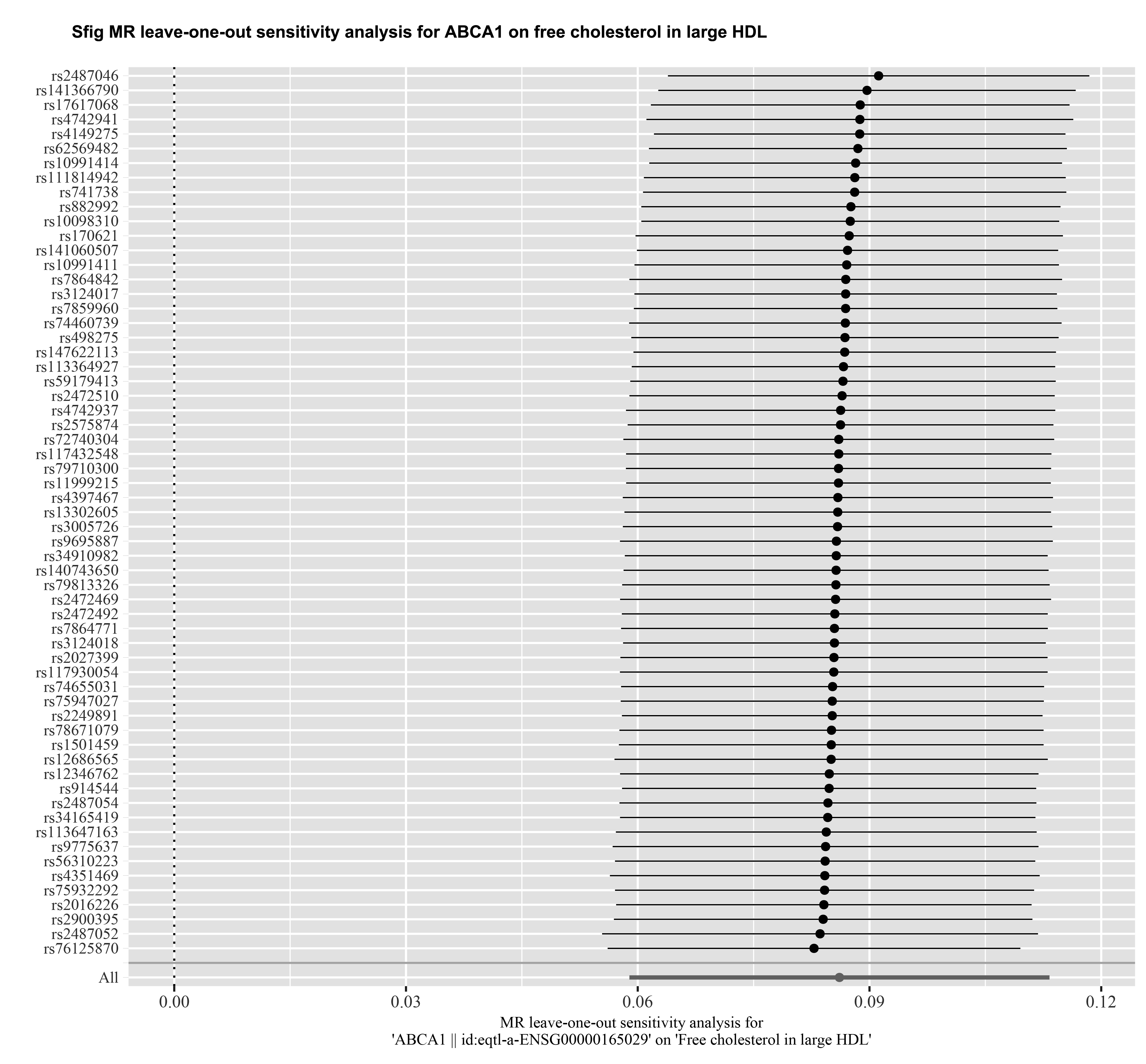

Supplement: Supplementary file 1 — Supplementary Information 1. [file 41598_2025_93644_MOESM1_ESM.zip › leave-one-out analysis/Sfig MR leave-one-out sensitivity analysis for ABCA1 on free cholesterol in large HDL.tif]

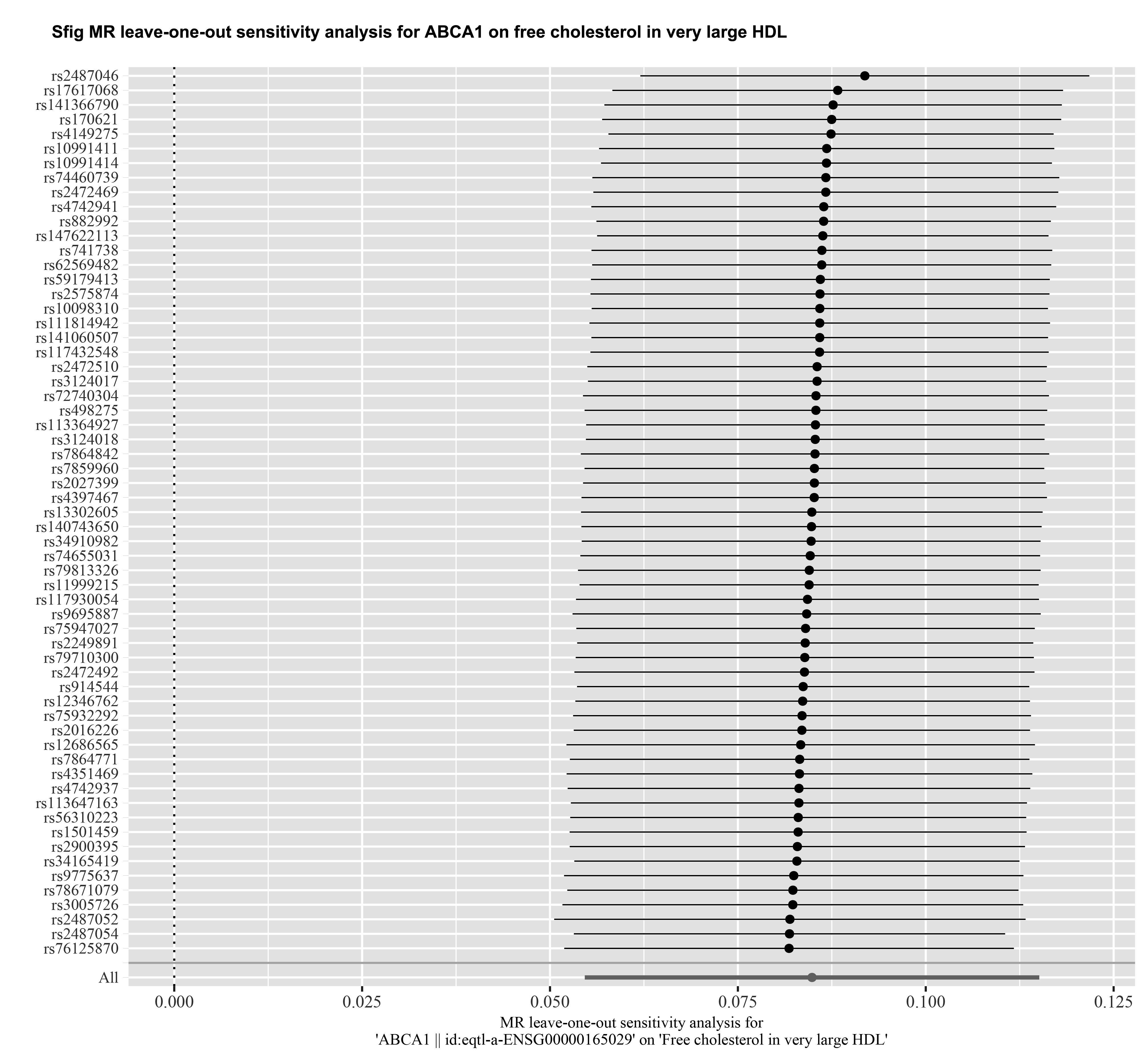

Supplement: Supplementary file 1 — Supplementary Information 1. [file 41598_2025_93644_MOESM1_ESM.zip › leave-one-out analysis/Sfig MR leave-one-out sensitivity analysis for ABCA1 on free cholesterol in very large HDL.tif]

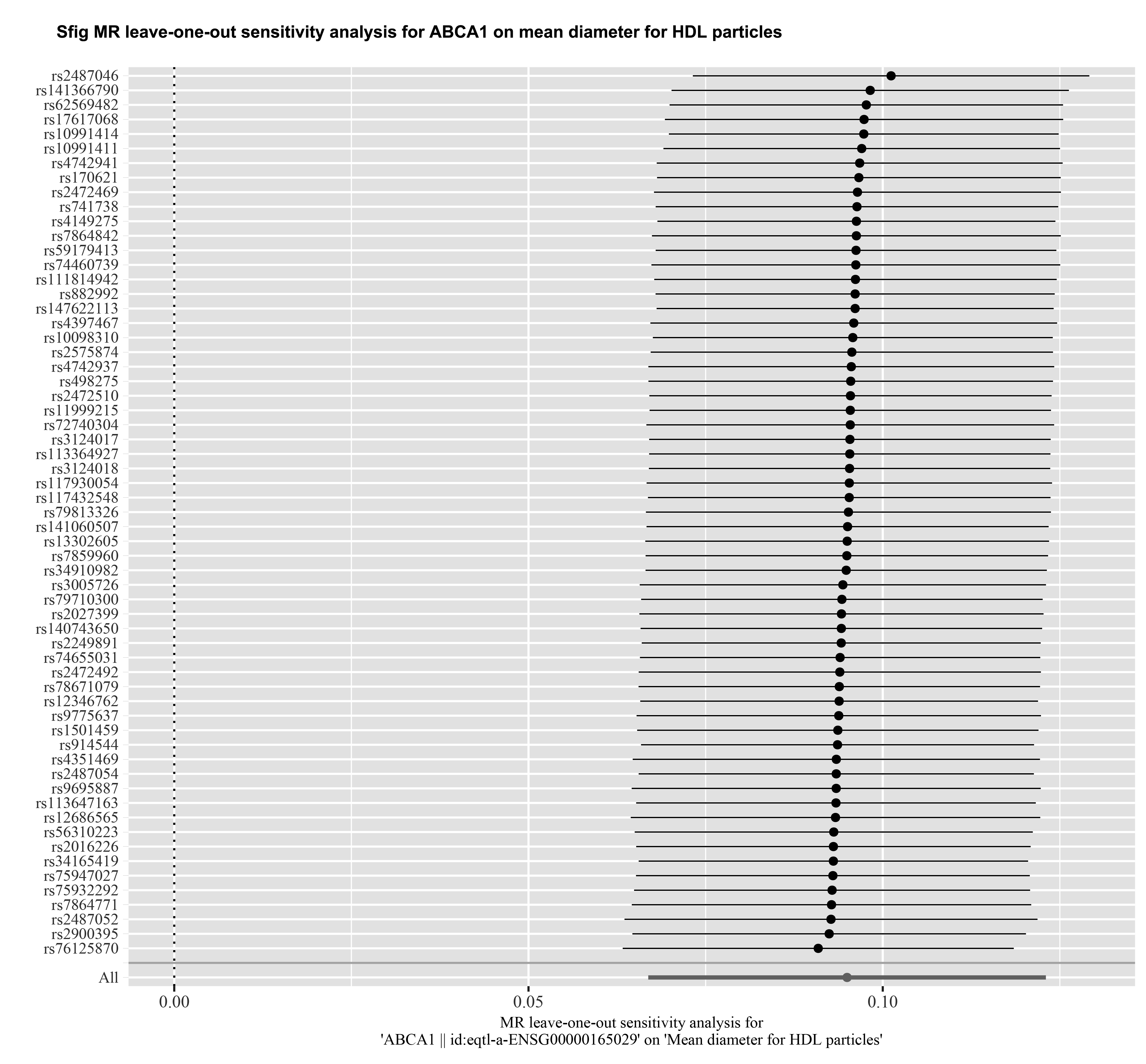

Supplement: Supplementary file 1 — Supplementary Information 1. [file 41598_2025_93644_MOESM1_ESM.zip › leave-one-out analysis/Sfig MR leave-one-out sensitivity analysis for ABCA1 on mean diameter for HDL particles.tif]

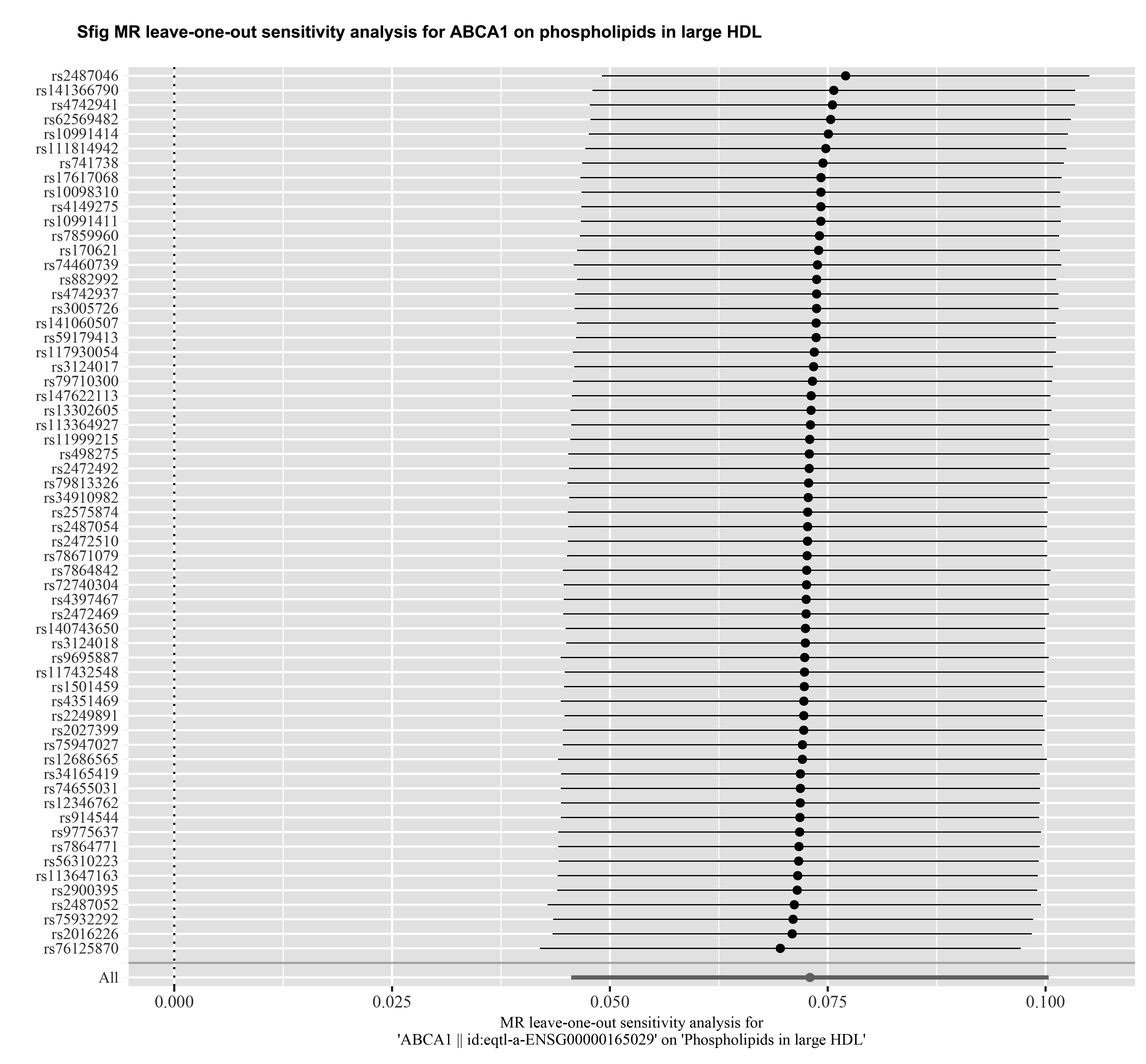

Supplement: Supplementary file 1 — Supplementary Information 1. [file 41598_2025_93644_MOESM1_ESM.zip › leave-one-out analysis/Sfig MR leave-one-out sensitivity analysis for ABCA1 on phospholipids in large HDL.tif]

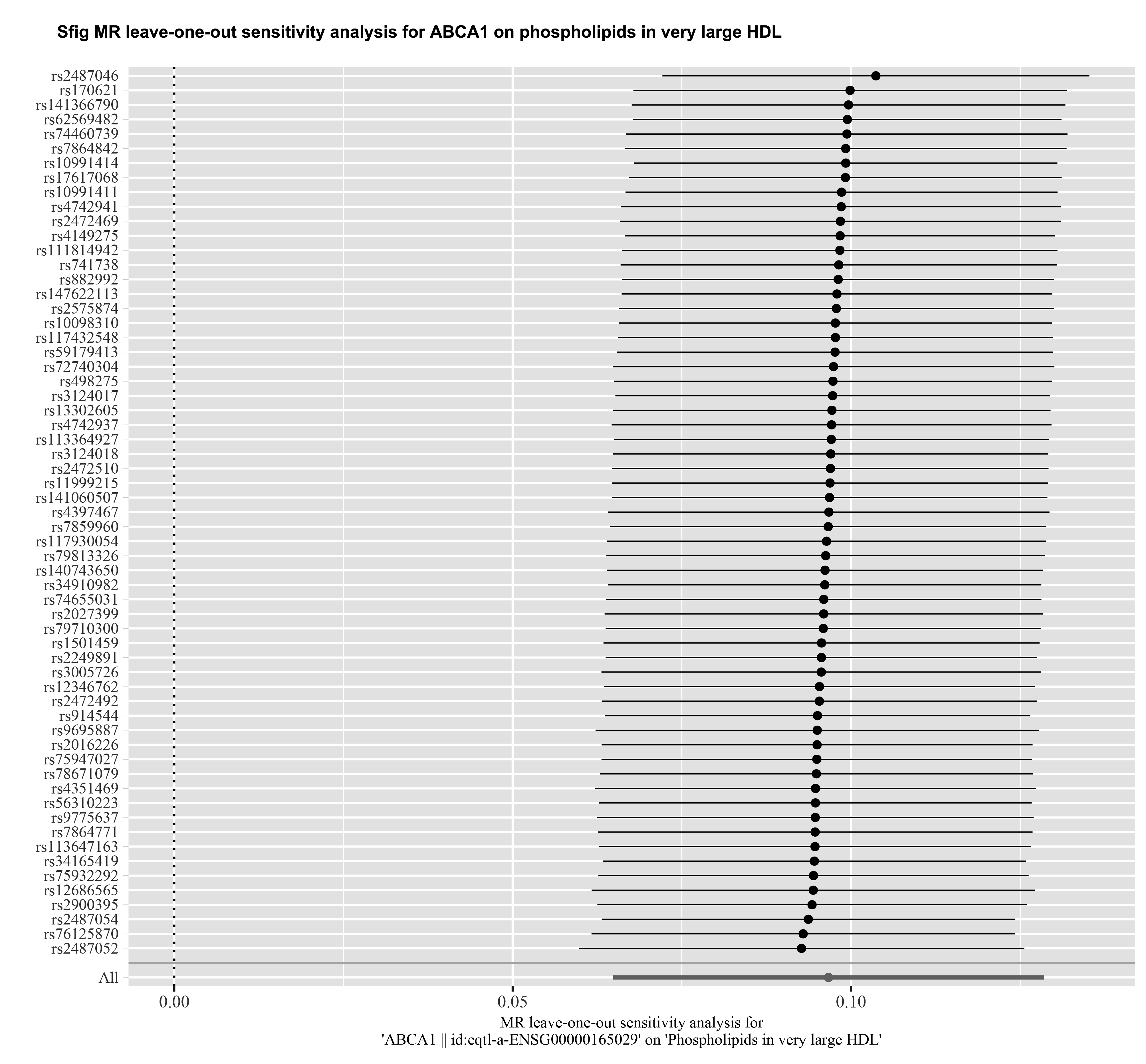

Supplement: Supplementary file 1 — Supplementary Information 1. [file 41598_2025_93644_MOESM1_ESM.zip › leave-one-out analysis/Sfig MR leave-one-out sensitivity analysis for ABCA1 on phospholipids in very large HDL.tif]

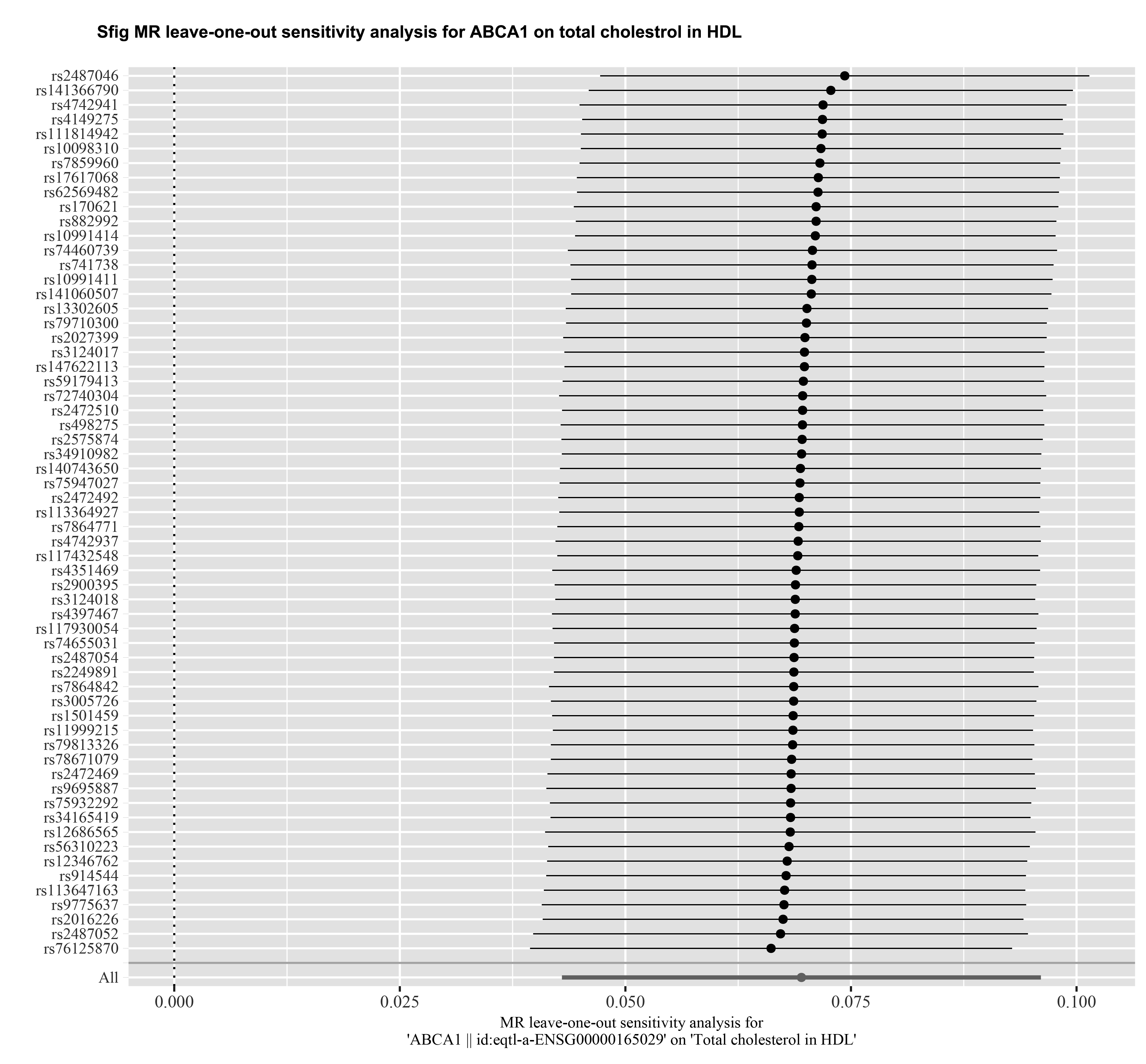

Supplement: Supplementary file 1 — Supplementary Information 1. [file 41598_2025_93644_MOESM1_ESM.zip › leave-one-out analysis/Sfig MR leave-one-out sensitivity analysis for ABCA1 on total cholestrol in HDL.tif]

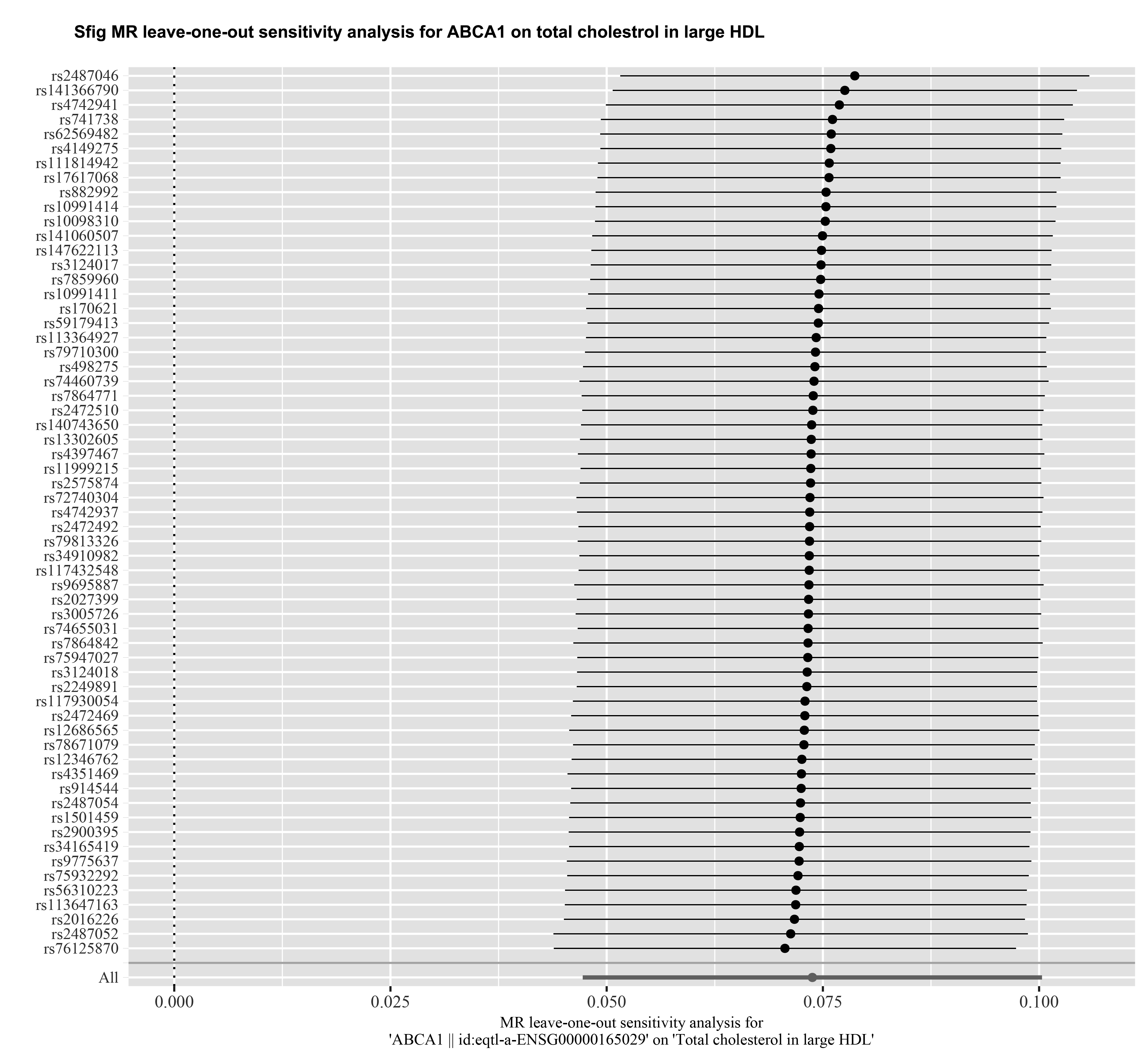

Supplement: Supplementary file 1 — Supplementary Information 1. [file 41598_2025_93644_MOESM1_ESM.zip › leave-one-out analysis/Sfig MR leave-one-out sensitivity analysis for ABCA1 on total cholestrol in large HDL.tif]

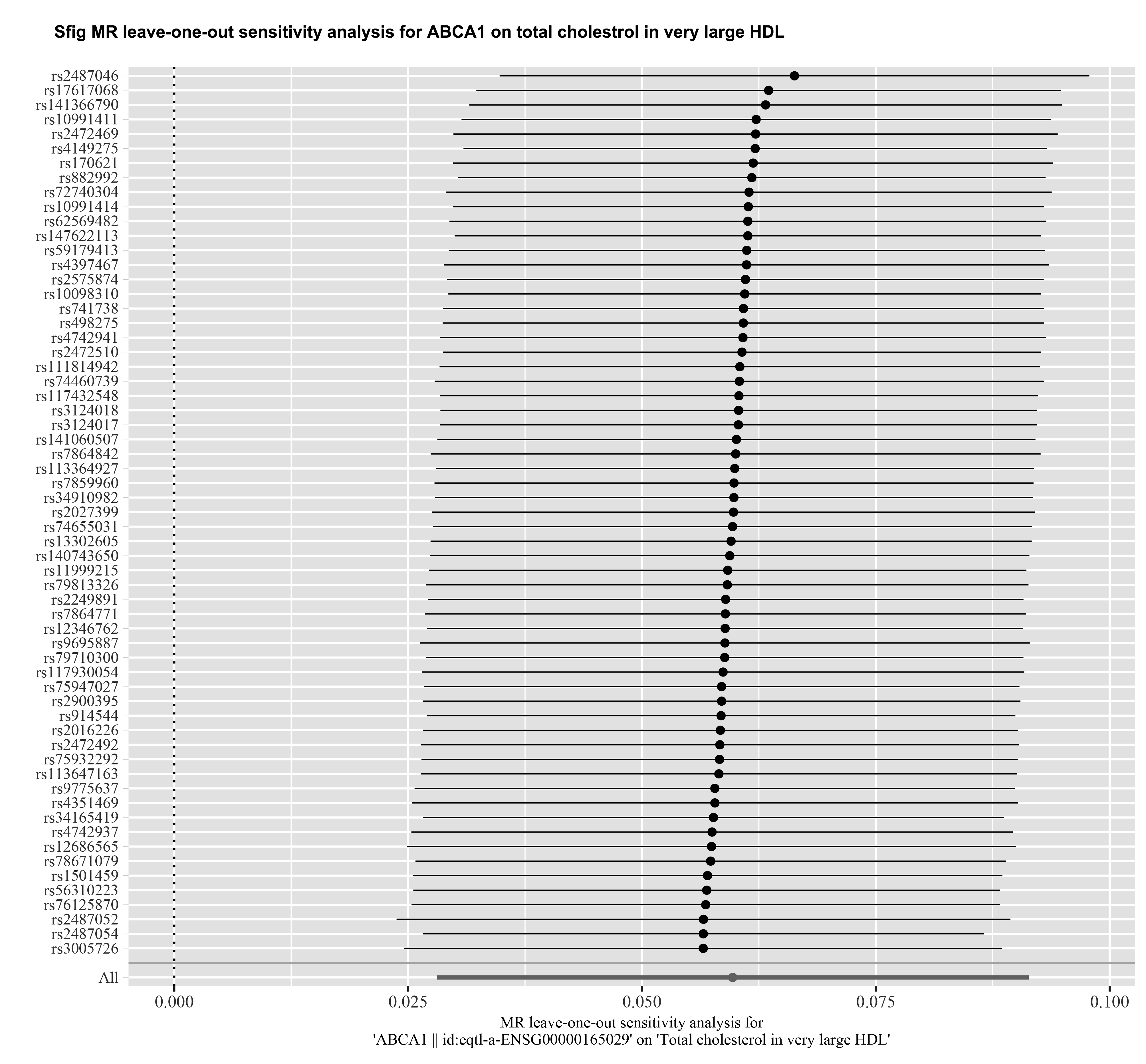

Supplement: Supplementary file 1 — Supplementary Information 1. [file 41598_2025_93644_MOESM1_ESM.zip › leave-one-out analysis/Sfig MR leave-one-out sensitivity analysis for ABCA1 on total cholestrol in very large HDL.tif]

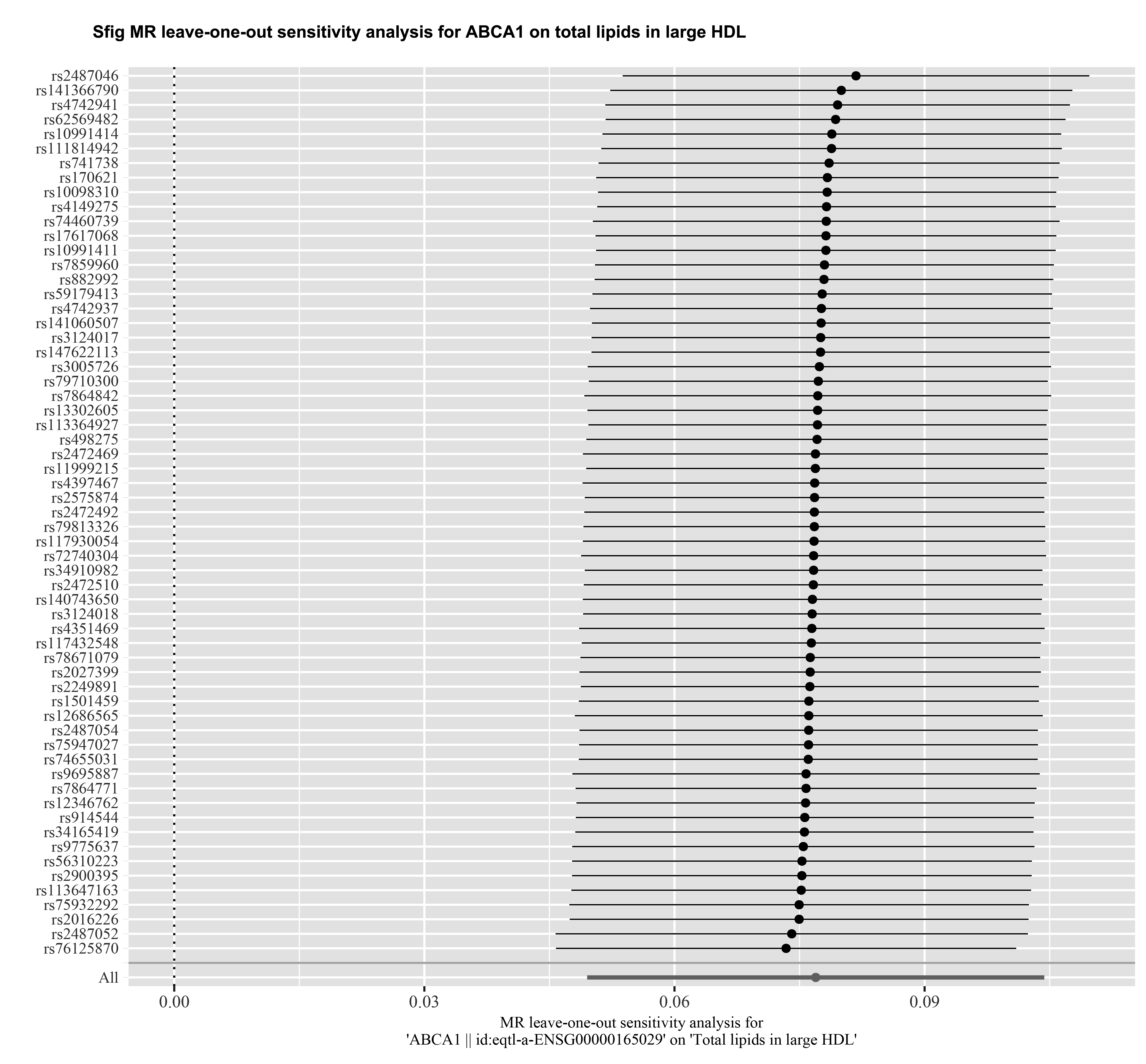

Supplement: Supplementary file 1 — Supplementary Information 1. [file 41598_2025_93644_MOESM1_ESM.zip › leave-one-out analysis/Sfig MR leave-one-out sensitivity analysis for ABCA1 on total lipids in large HDL.tif]

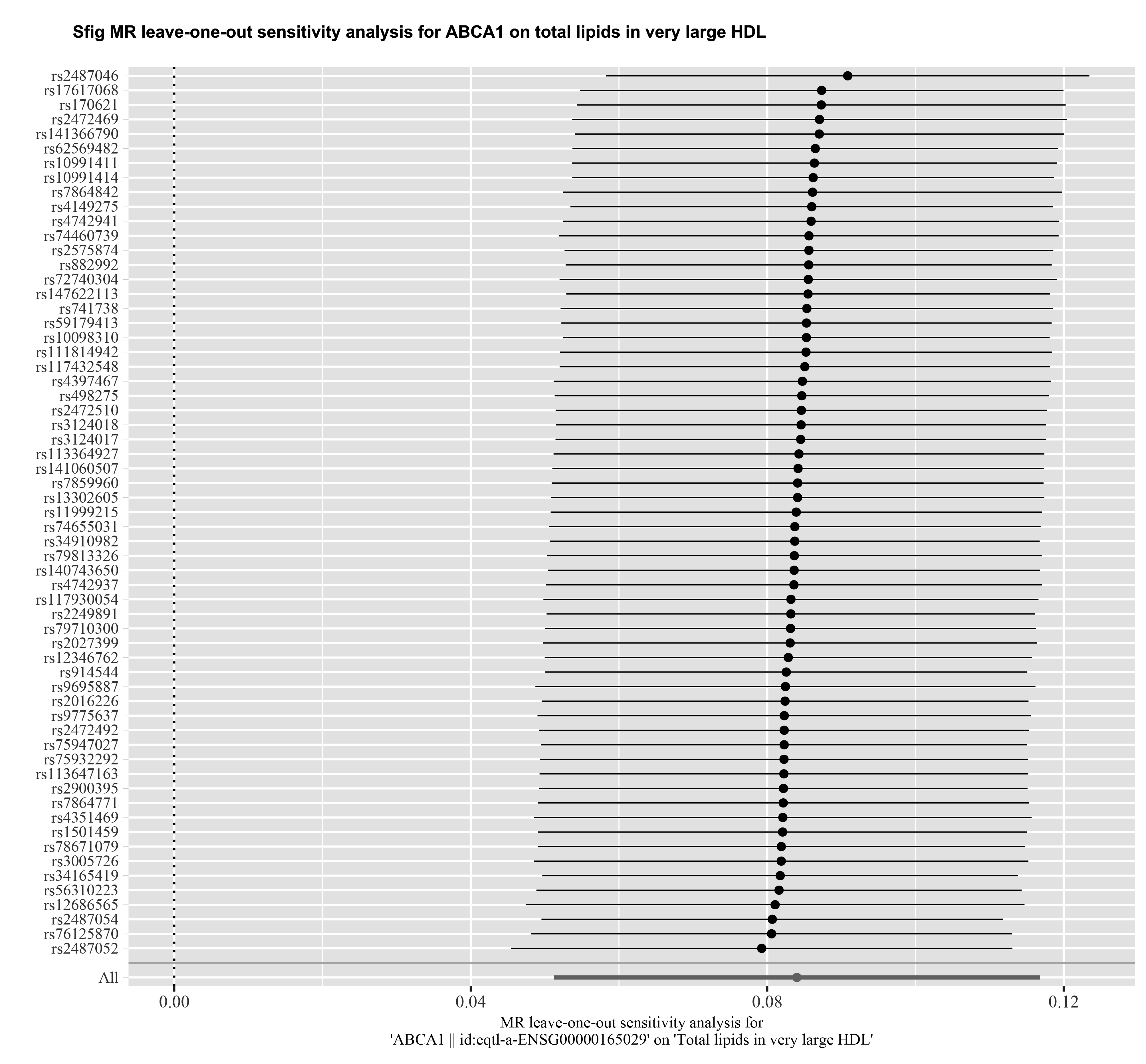

Supplement: Supplementary file 1 — Supplementary Information 1. [file 41598_2025_93644_MOESM1_ESM.zip › leave-one-out analysis/Sfig MR leave-one-out sensitivity analysis for ABCA1 on total lipids in very large HDL.tif]

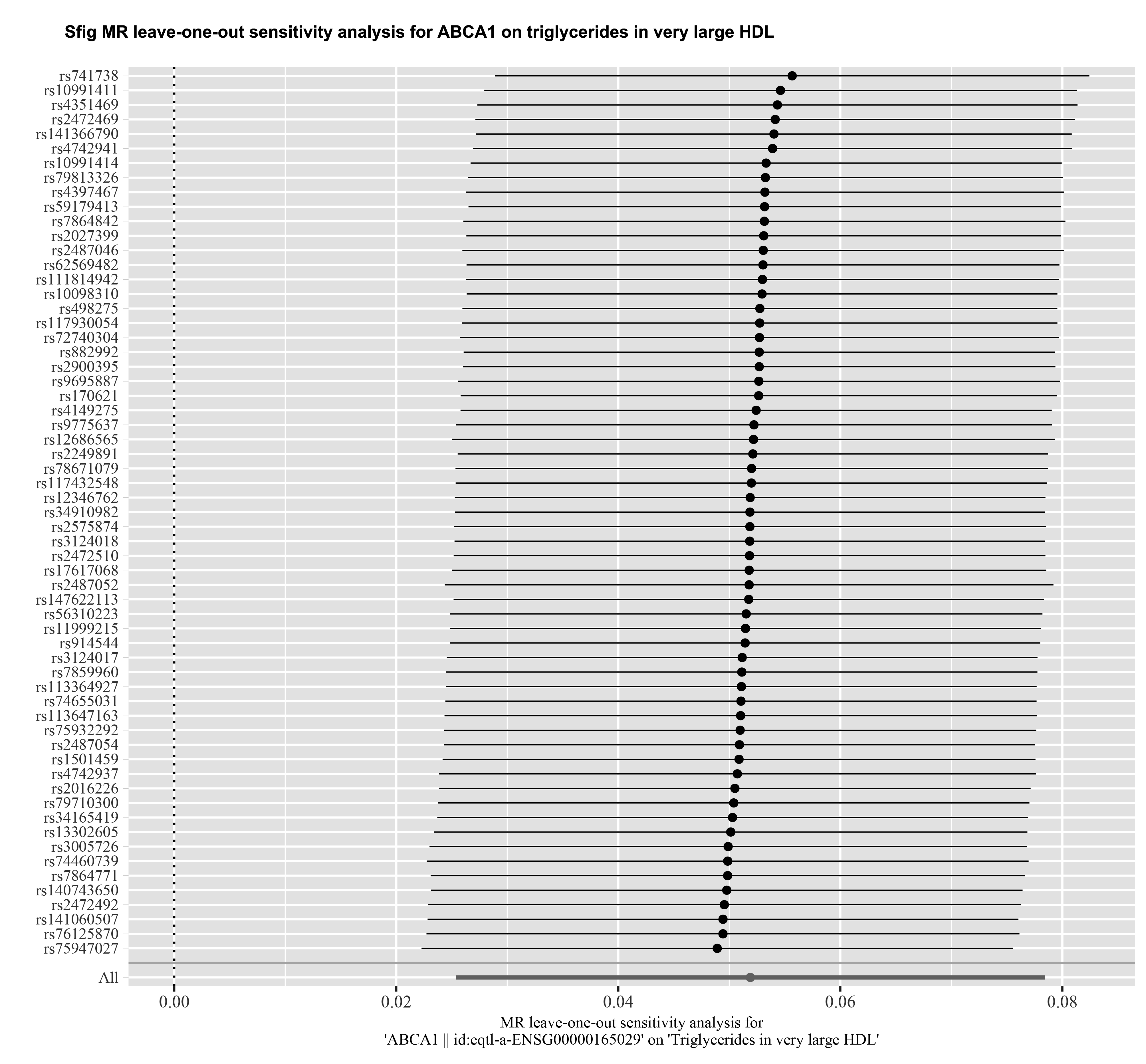

Supplement: Supplementary file 1 — Supplementary Information 1. [file 41598_2025_93644_MOESM1_ESM.zip › leave-one-out analysis/Sfig MR leave-one-out sensitivity analysis for ABCA1 on triglycerides in very large HDL.tif]

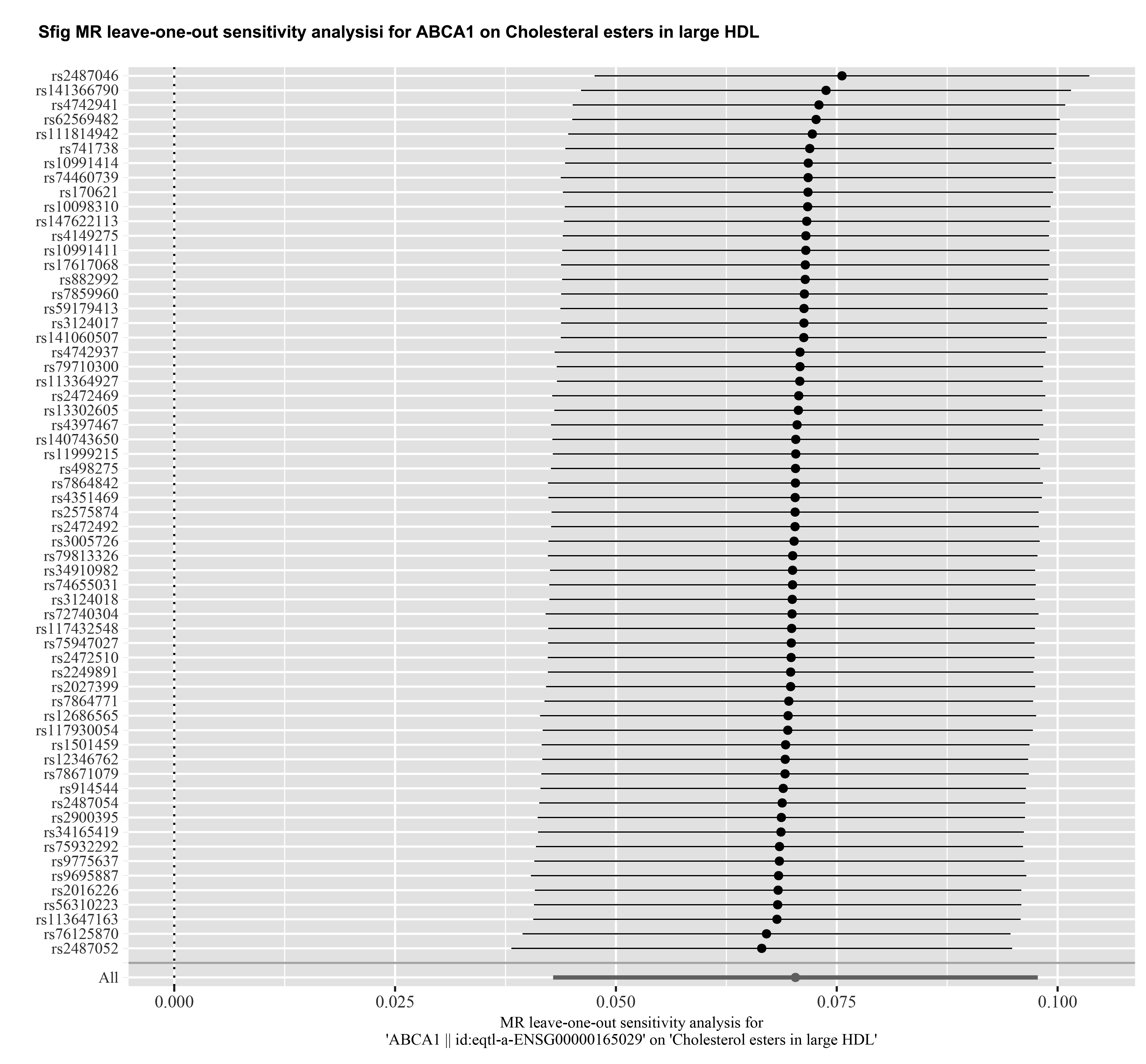

Supplement: Supplementary file 1 — Supplementary Information 1. [file 41598_2025_93644_MOESM1_ESM.zip › leave-one-out analysis/Sfig MR leave-one-out sensitivity analysisi for ABCA1 on Cholesteral esters in large HDL.tif]

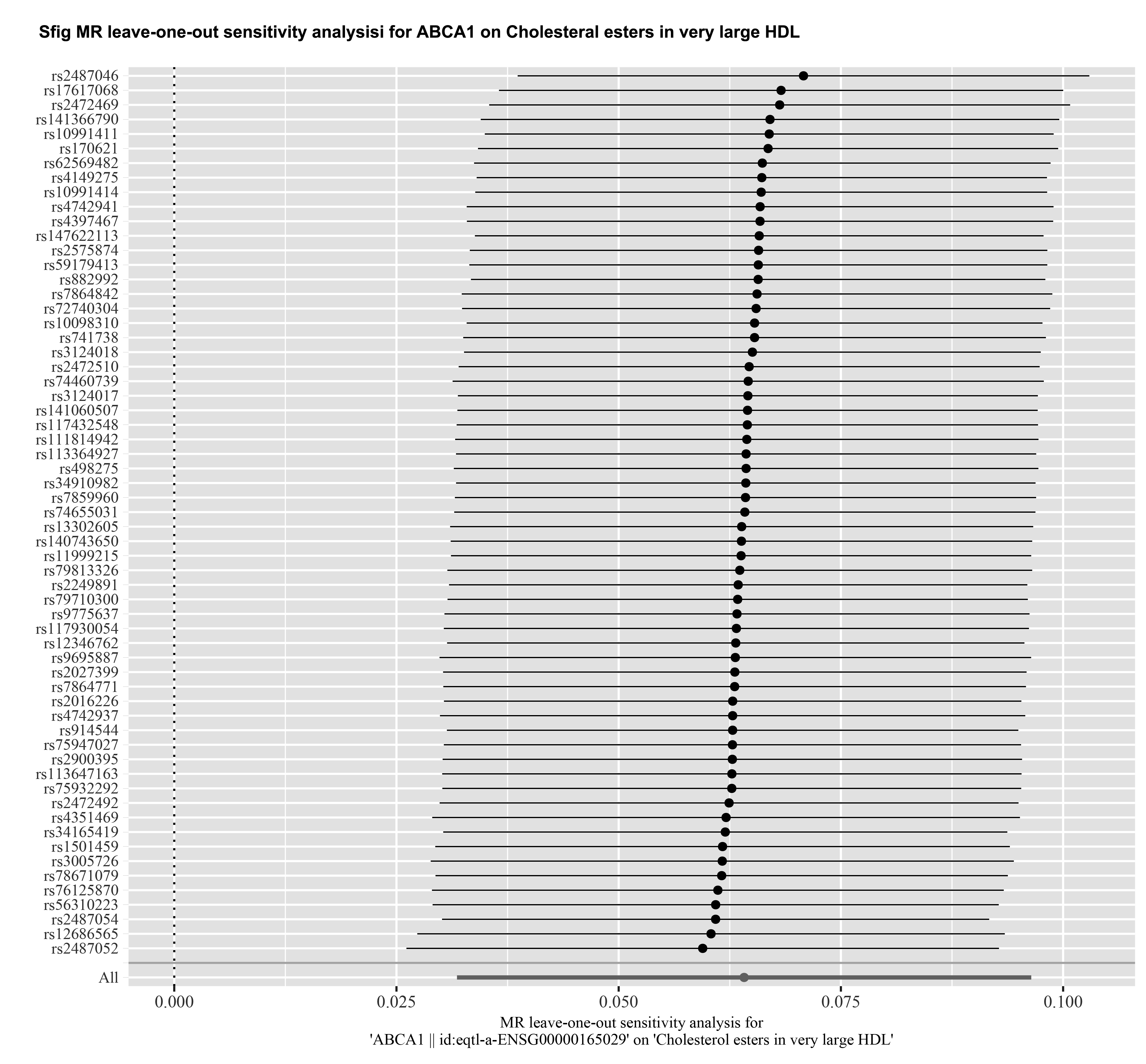

Supplement: Supplementary file 1 — Supplementary Information 1. [file 41598_2025_93644_MOESM1_ESM.zip › leave-one-out analysis/Sfig MR leave-one-out sensitivity analysisi for ABCA1 on Cholesteral esters in very large HDL.tif]

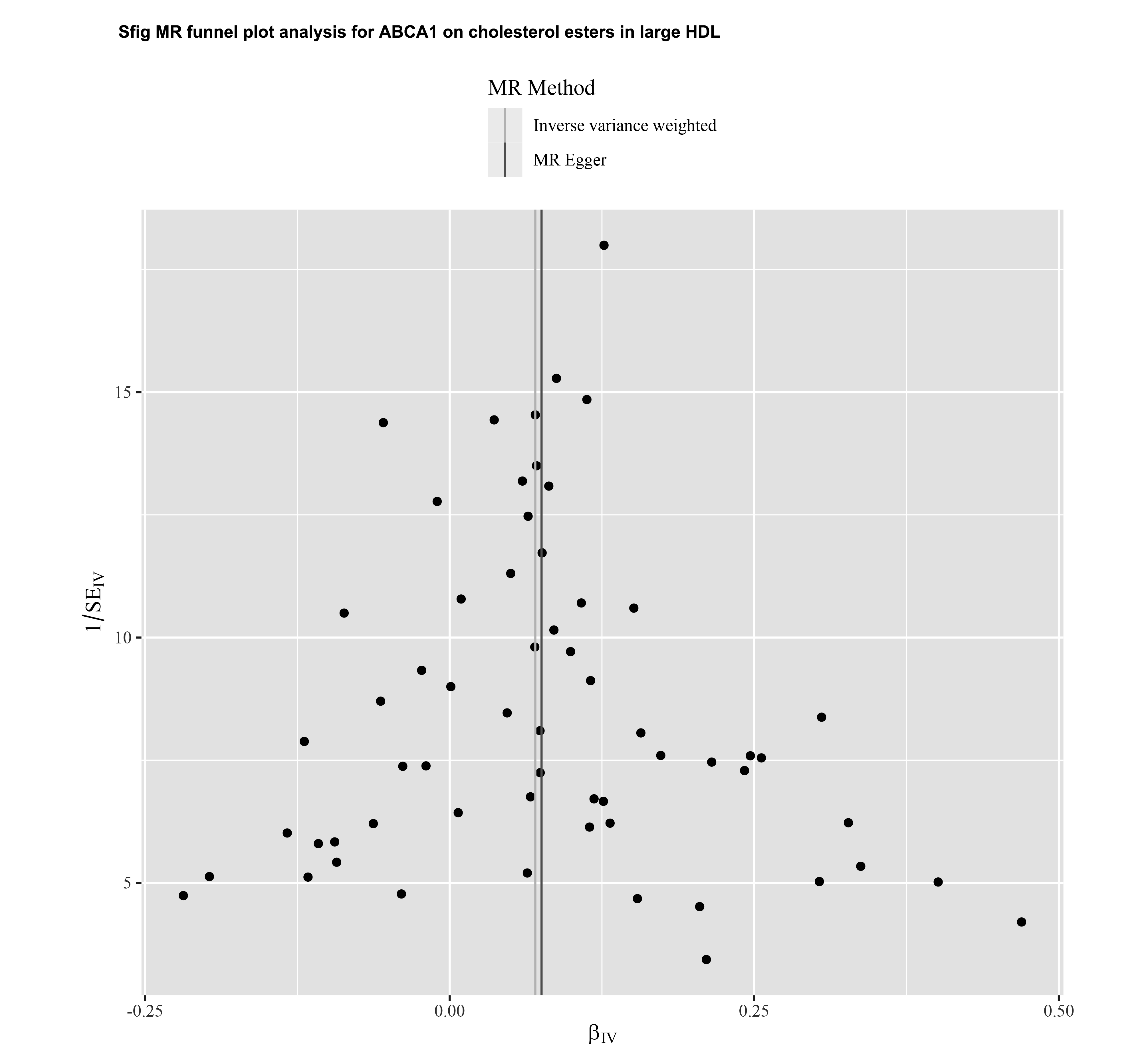

Supplement: Supplementary file 1 — Supplementary Information 1. [file 41598_2025_93644_MOESM1_ESM.zip › the funnel plot/Sfig MR funnel plot analysis for ABCA1 on cholesterol esters in large HDL.tif]

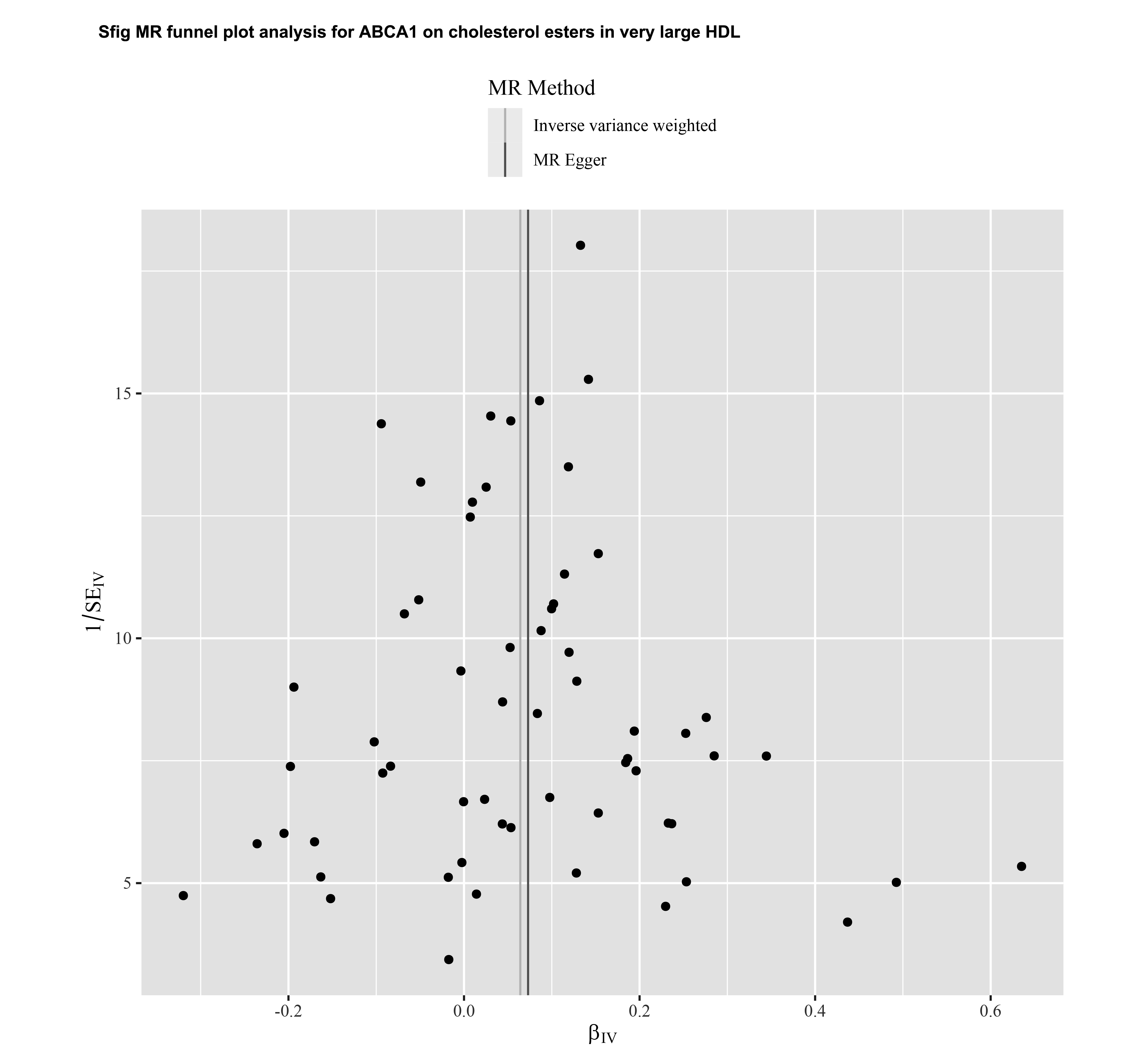

Supplement: Supplementary file 1 — Supplementary Information 1. [file 41598_2025_93644_MOESM1_ESM.zip › the funnel plot/Sfig MR funnel plot analysis for ABCA1 on cholesterol esters in very large HDL.tif]

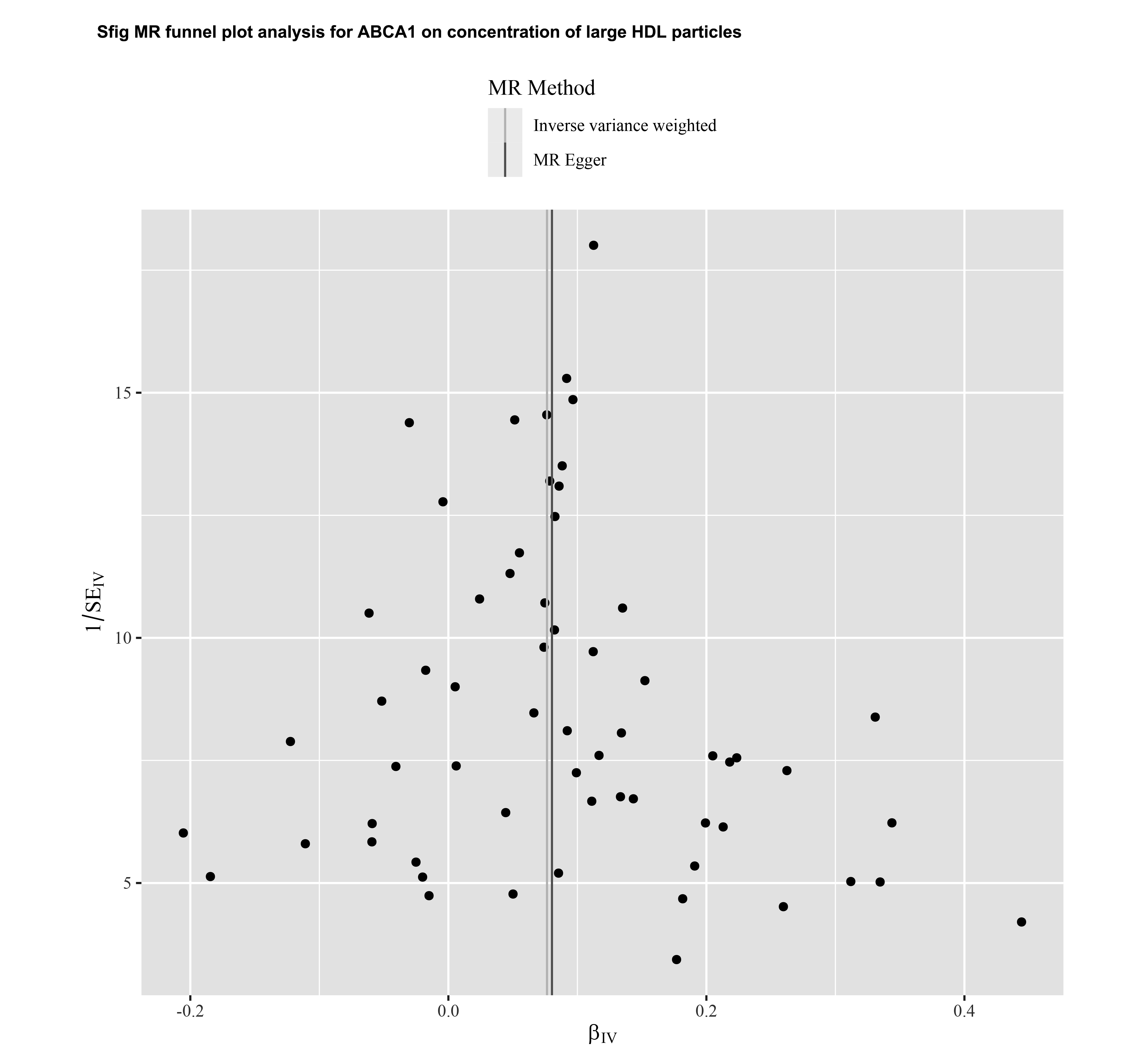

Supplement: Supplementary file 1 — Supplementary Information 1. [file 41598_2025_93644_MOESM1_ESM.zip › the funnel plot/Sfig MR funnel plot analysis for ABCA1 on concentration of large HDL particles.tif]

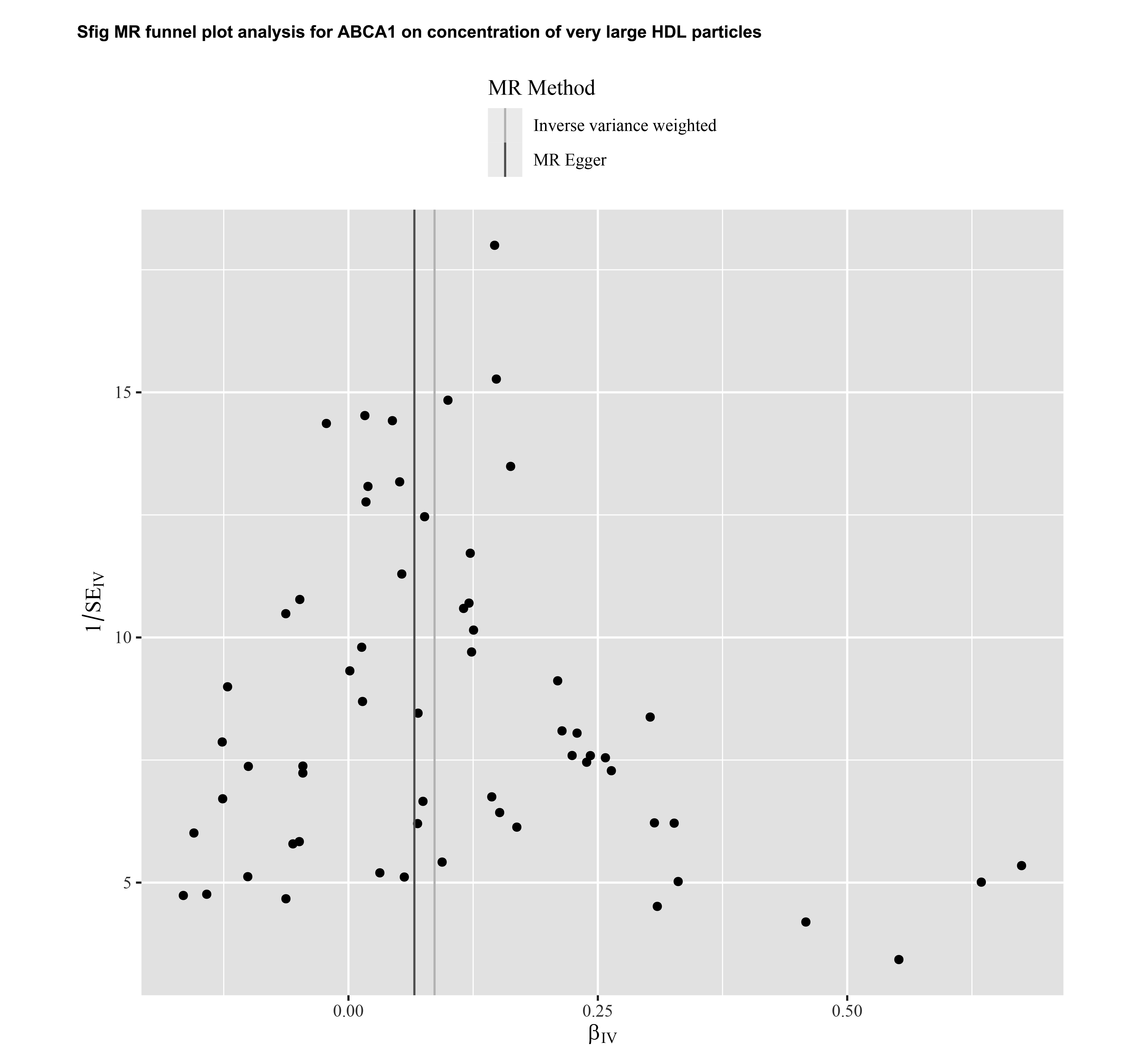

Supplement: Supplementary file 1 — Supplementary Information 1. [file 41598_2025_93644_MOESM1_ESM.zip › the funnel plot/Sfig MR funnel plot analysis for ABCA1 on concentration of very large HDL particles.tif]

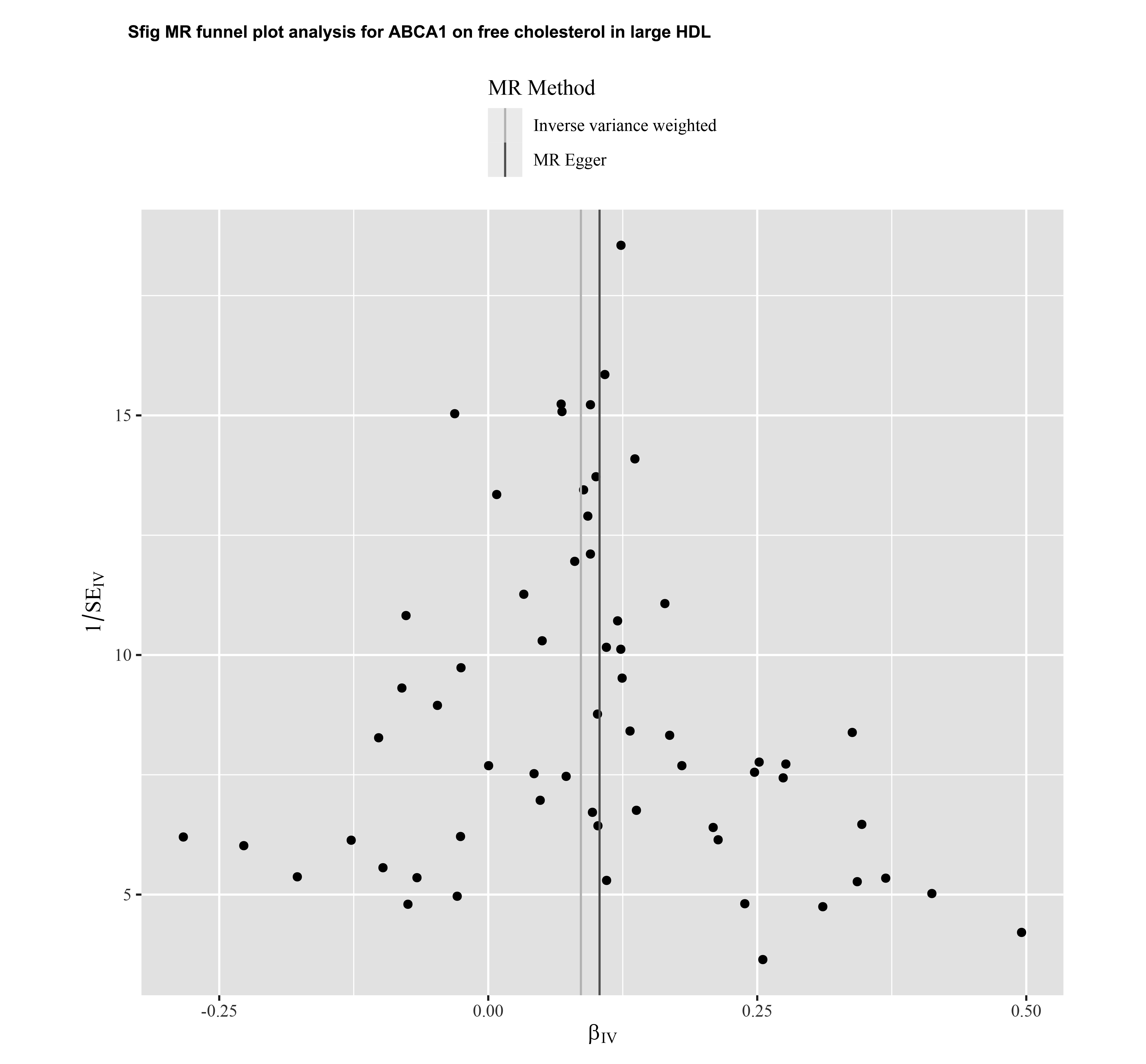

Supplement: Supplementary file 1 — Supplementary Information 1. [file 41598_2025_93644_MOESM1_ESM.zip › the funnel plot/Sfig MR funnel plot analysis for ABCA1 on free cholesterol in large HDL.tif]

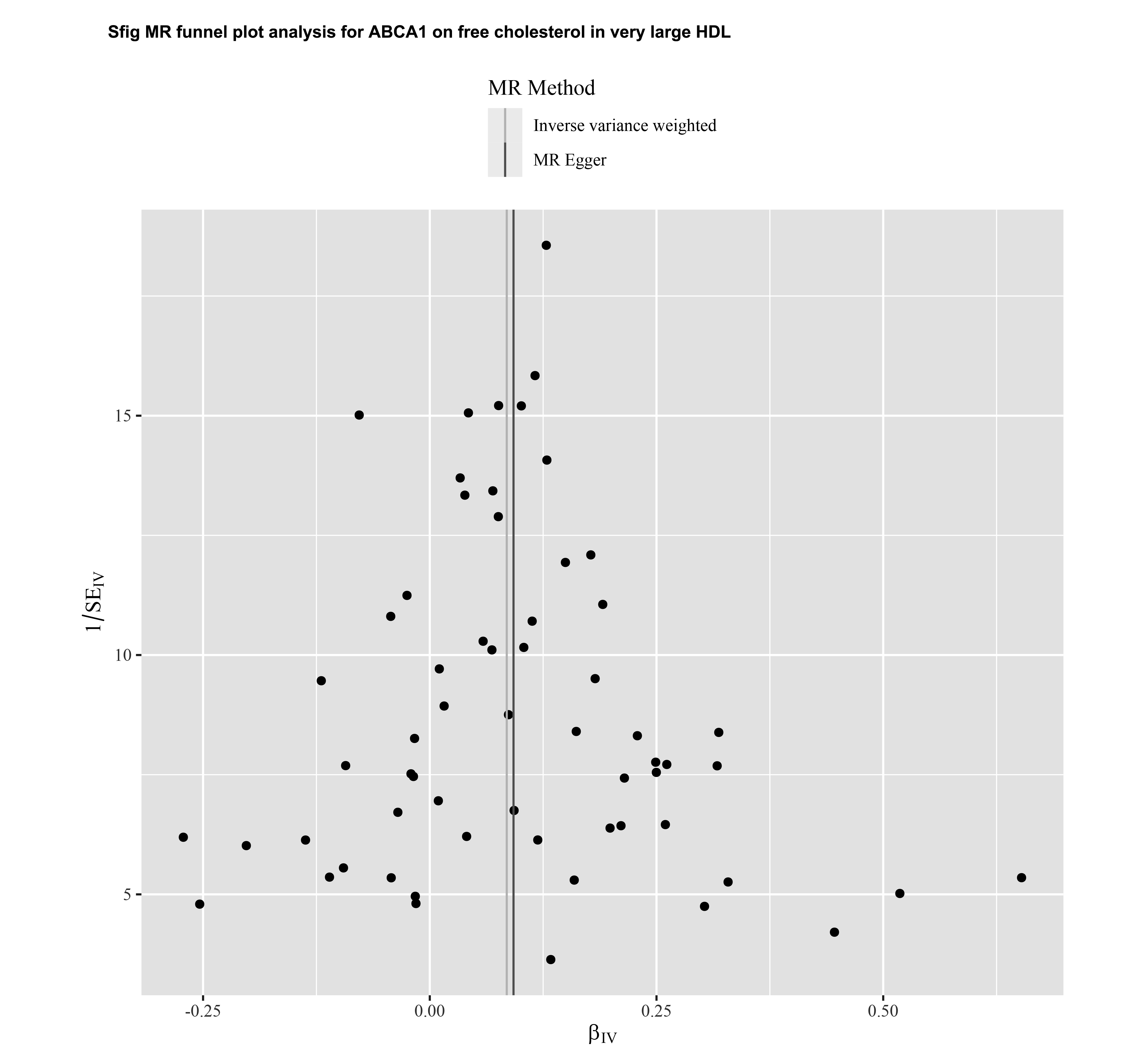

Supplement: Supplementary file 1 — Supplementary Information 1. [file 41598_2025_93644_MOESM1_ESM.zip › the funnel plot/Sfig MR funnel plot analysis for ABCA1 on free cholesterol in very large HDL.tif]

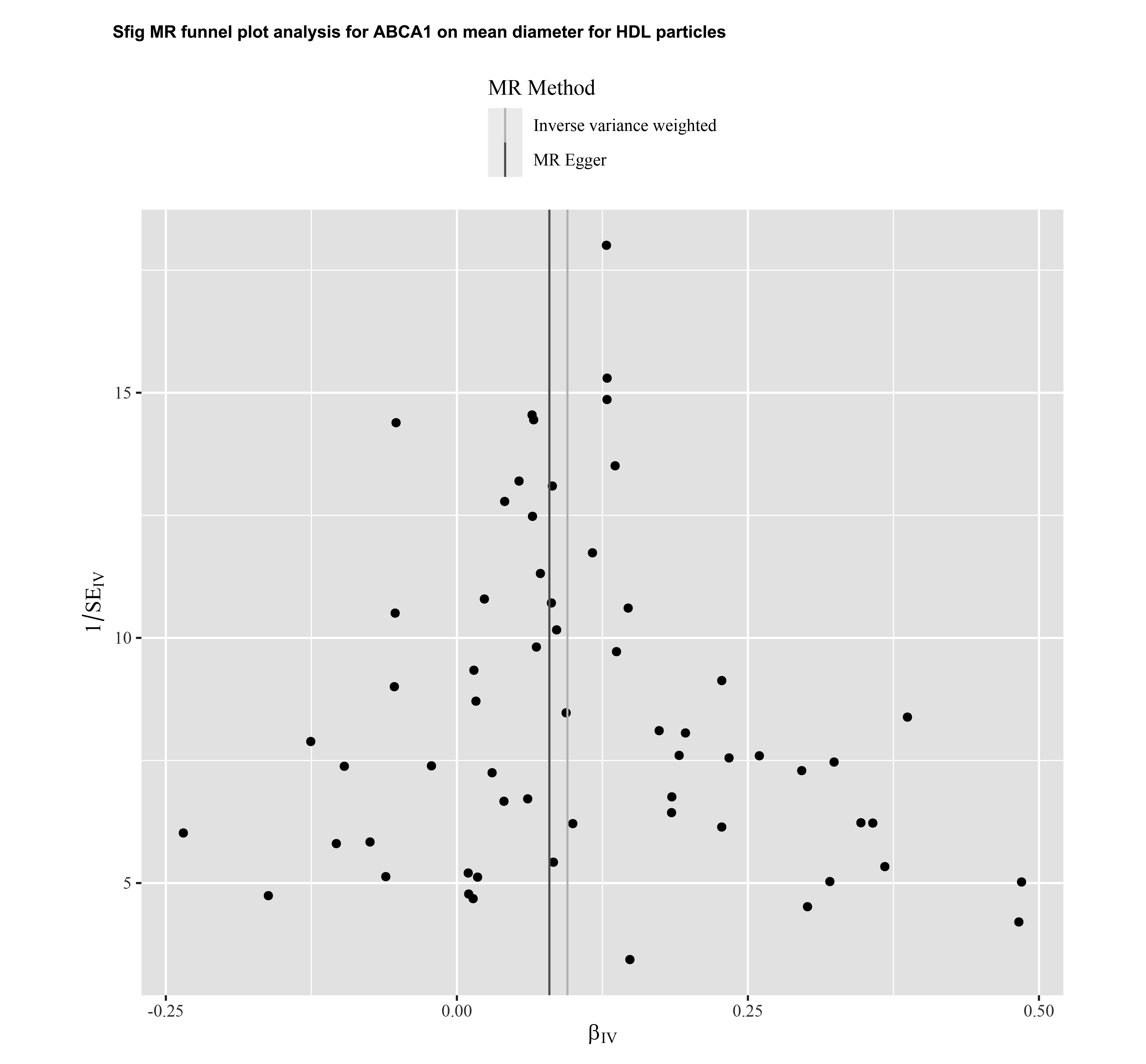

Supplement: Supplementary file 1 — Supplementary Information 1. [file 41598_2025_93644_MOESM1_ESM.zip › the funnel plot/Sfig MR funnel plot analysis for ABCA1 on mean diameter for HDL particles.tif]

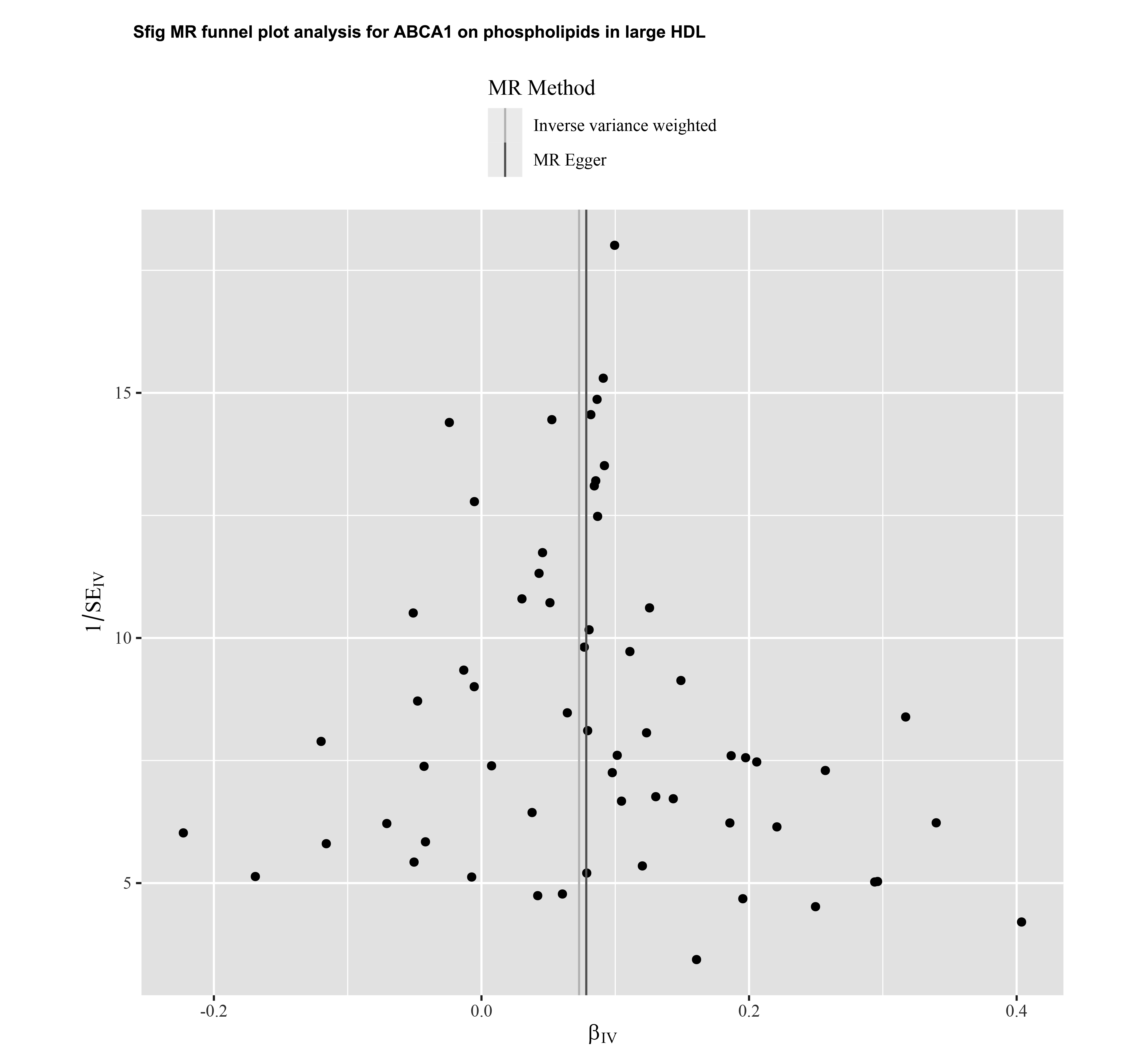

Supplement: Supplementary file 1 — Supplementary Information 1. [file 41598_2025_93644_MOESM1_ESM.zip › the funnel plot/Sfig MR funnel plot analysis for ABCA1 on phospholipids in large HDL.tif]

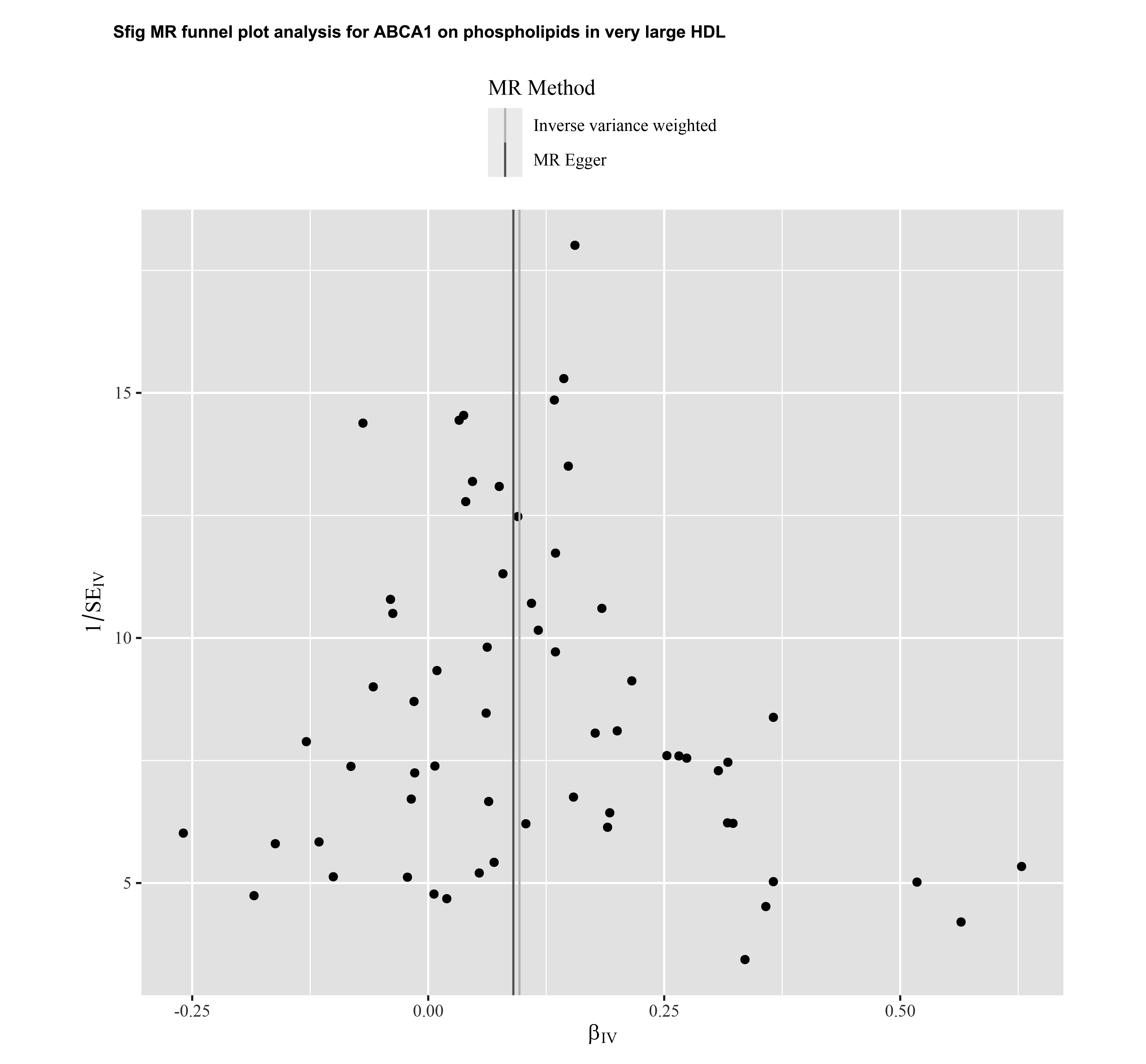

Supplement: Supplementary file 1 — Supplementary Information 1. [file 41598_2025_93644_MOESM1_ESM.zip › the funnel plot/Sfig MR funnel plot analysis for ABCA1 on phospholipids in very large HDL.tif]

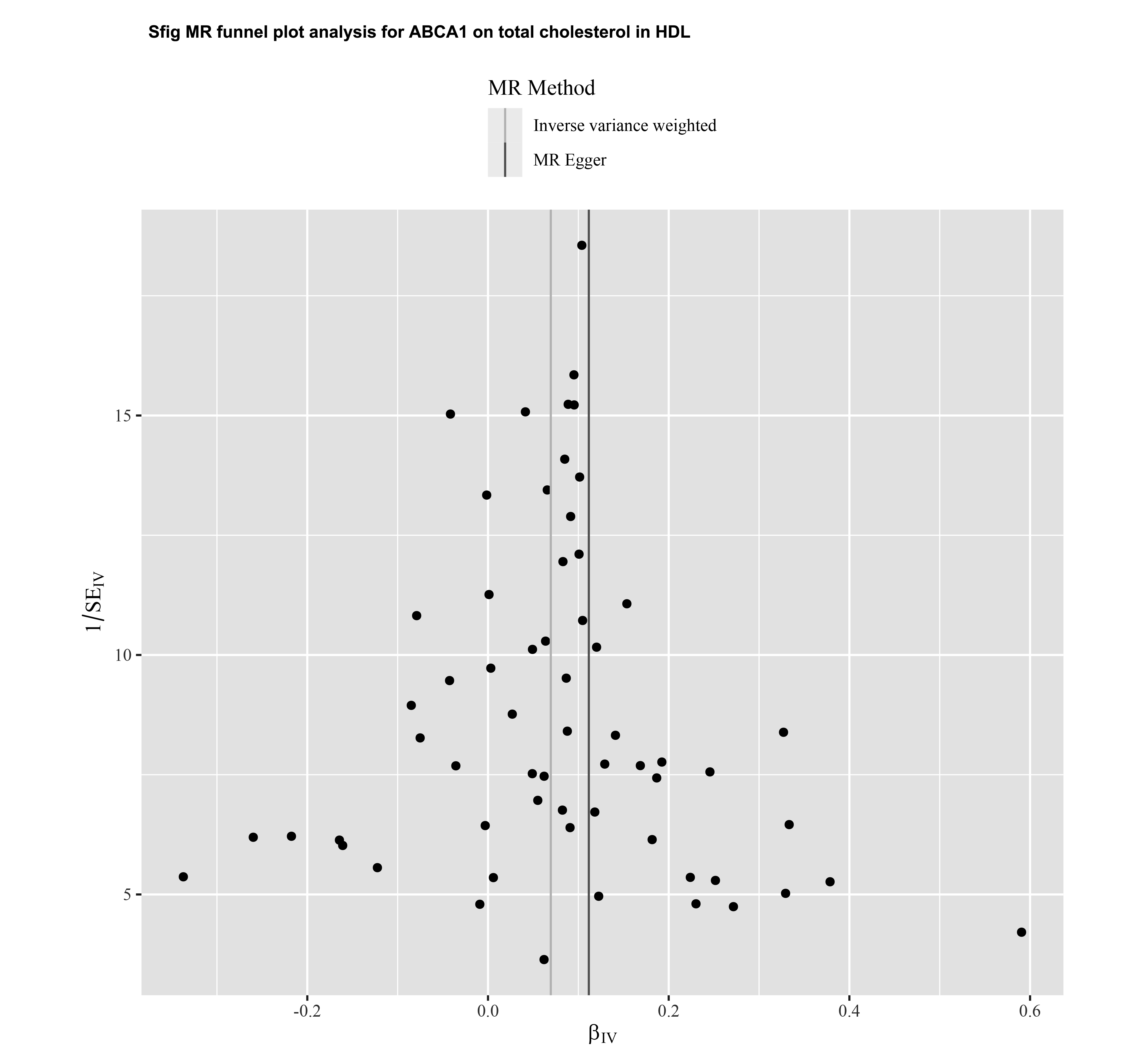

Supplement: Supplementary file 1 — Supplementary Information 1. [file 41598_2025_93644_MOESM1_ESM.zip › the funnel plot/Sfig MR funnel plot analysis for ABCA1 on total cholesterol in HDL.tif]

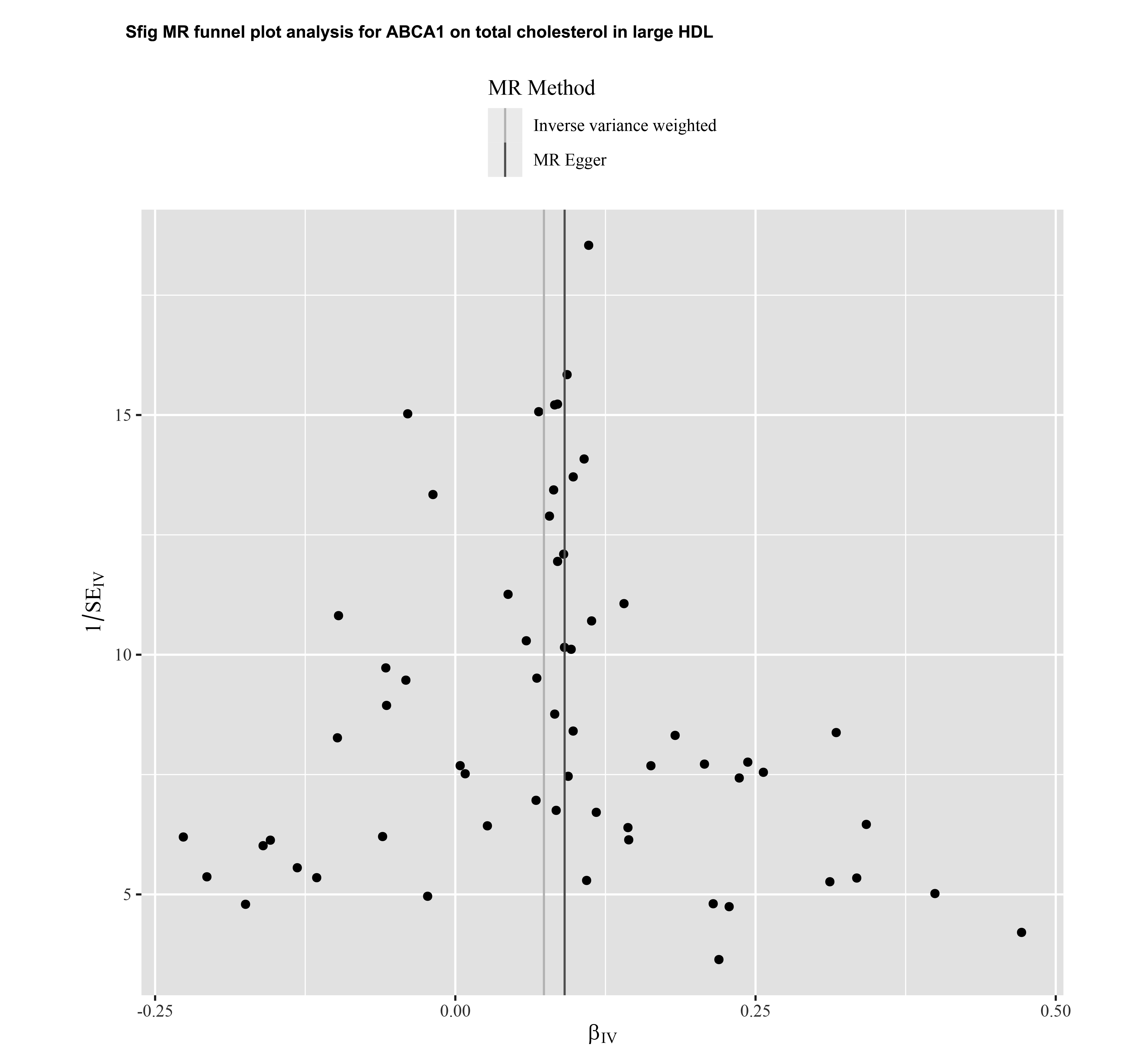

Supplement: Supplementary file 1 — Supplementary Information 1. [file 41598_2025_93644_MOESM1_ESM.zip › the funnel plot/Sfig MR funnel plot analysis for ABCA1 on total cholesterol in large HDL.tif]

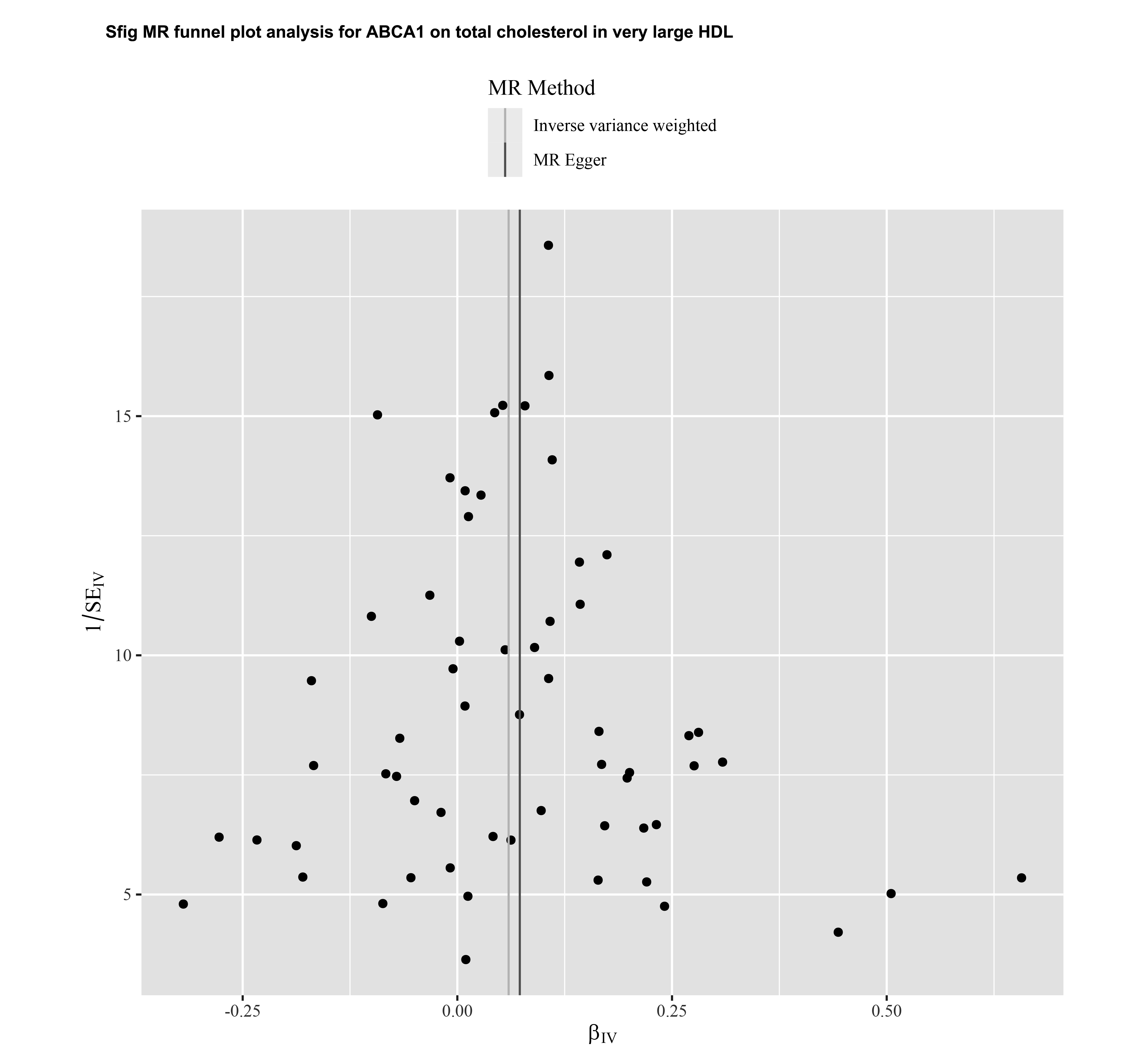

Supplement: Supplementary file 1 — Supplementary Information 1. [file 41598_2025_93644_MOESM1_ESM.zip › the funnel plot/Sfig MR funnel plot analysis for ABCA1 on total cholesterol in very large HDL.tif]

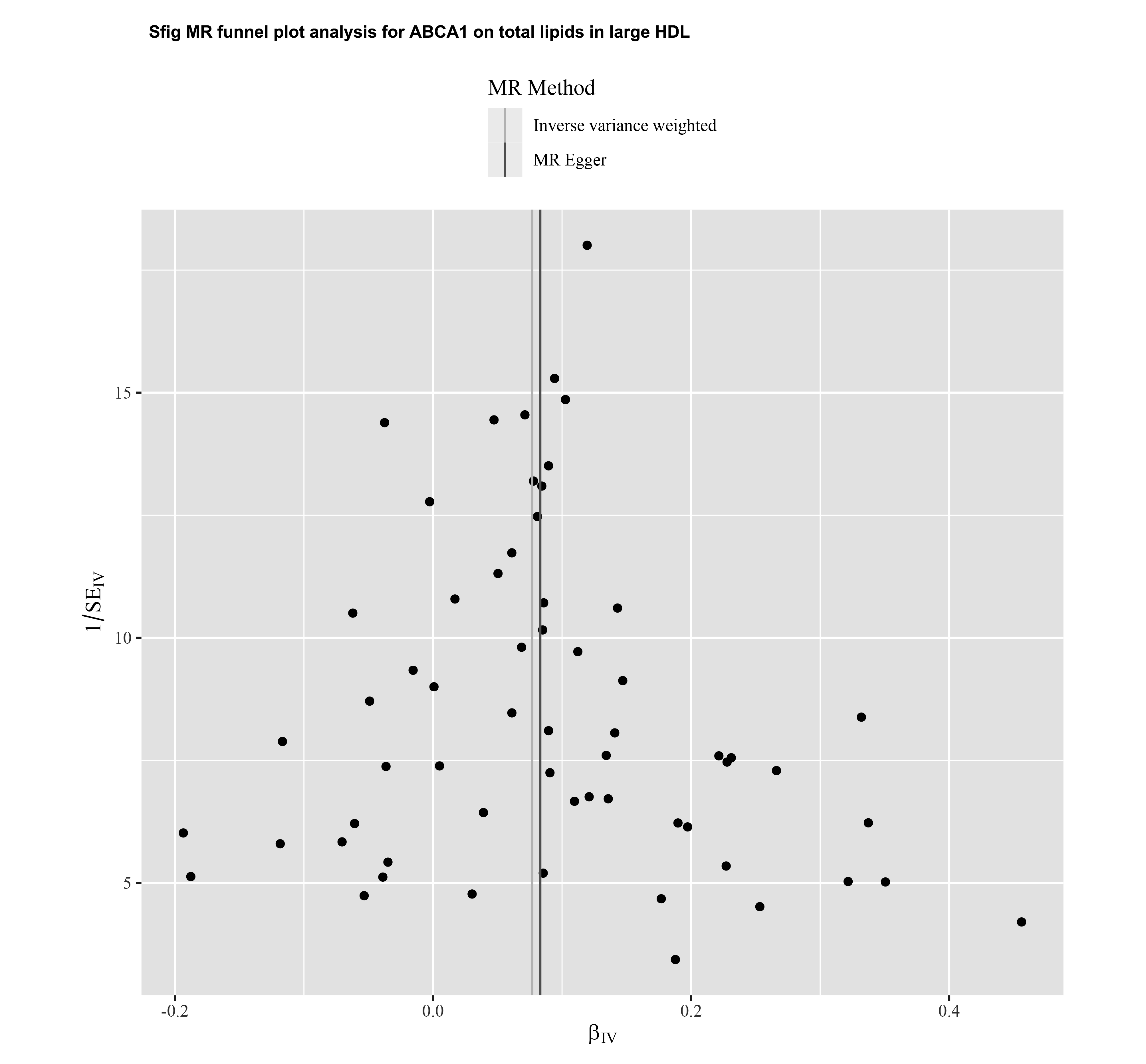

Supplement: Supplementary file 1 — Supplementary Information 1. [file 41598_2025_93644_MOESM1_ESM.zip › the funnel plot/Sfig MR funnel plot analysis for ABCA1 on total lipids in large HDL.tif]

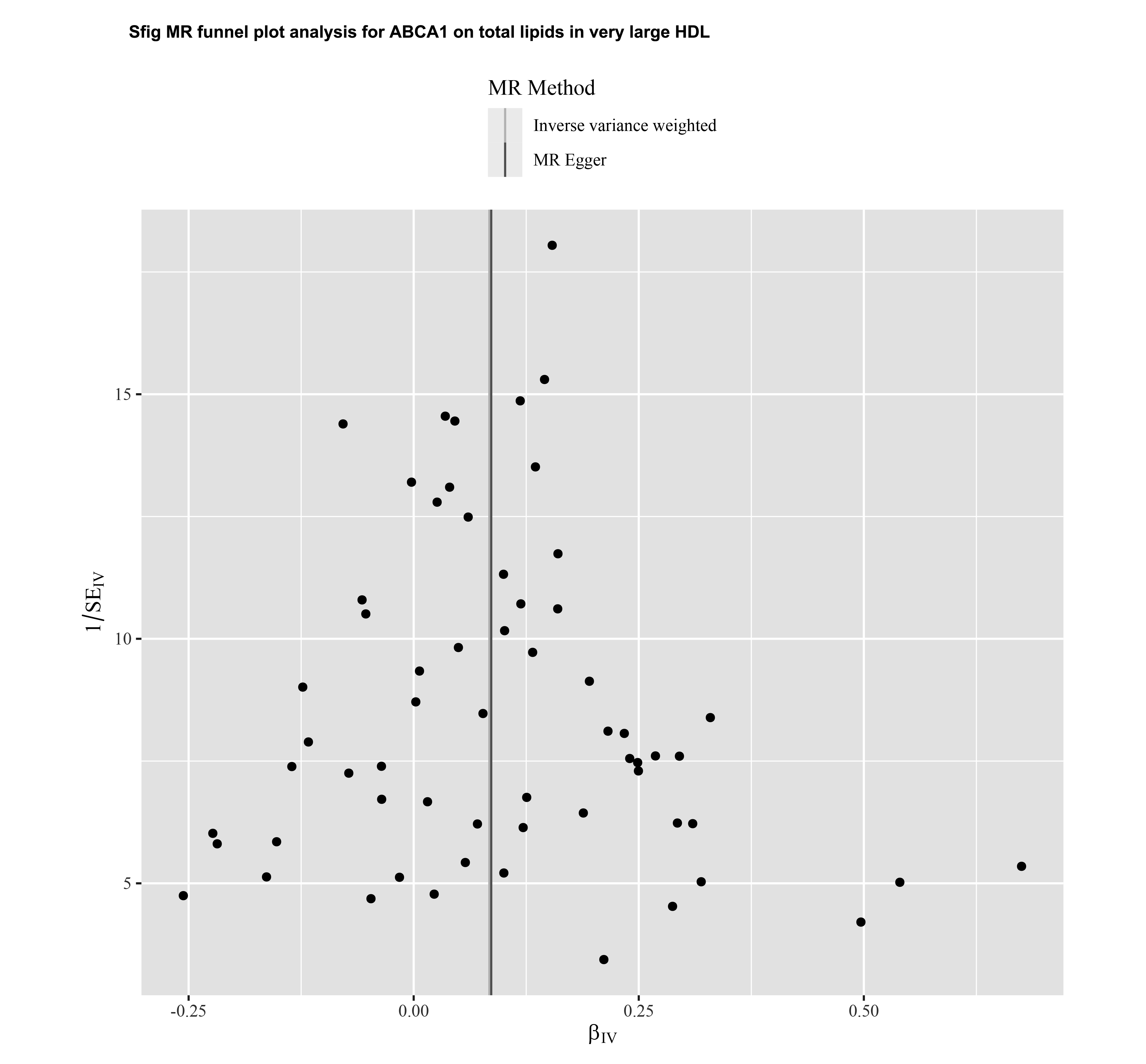

Supplement: Supplementary file 1 — Supplementary Information 1. [file 41598_2025_93644_MOESM1_ESM.zip › the funnel plot/Sfig MR funnel plot analysis for ABCA1 on total lipids in very large HDL.tif]

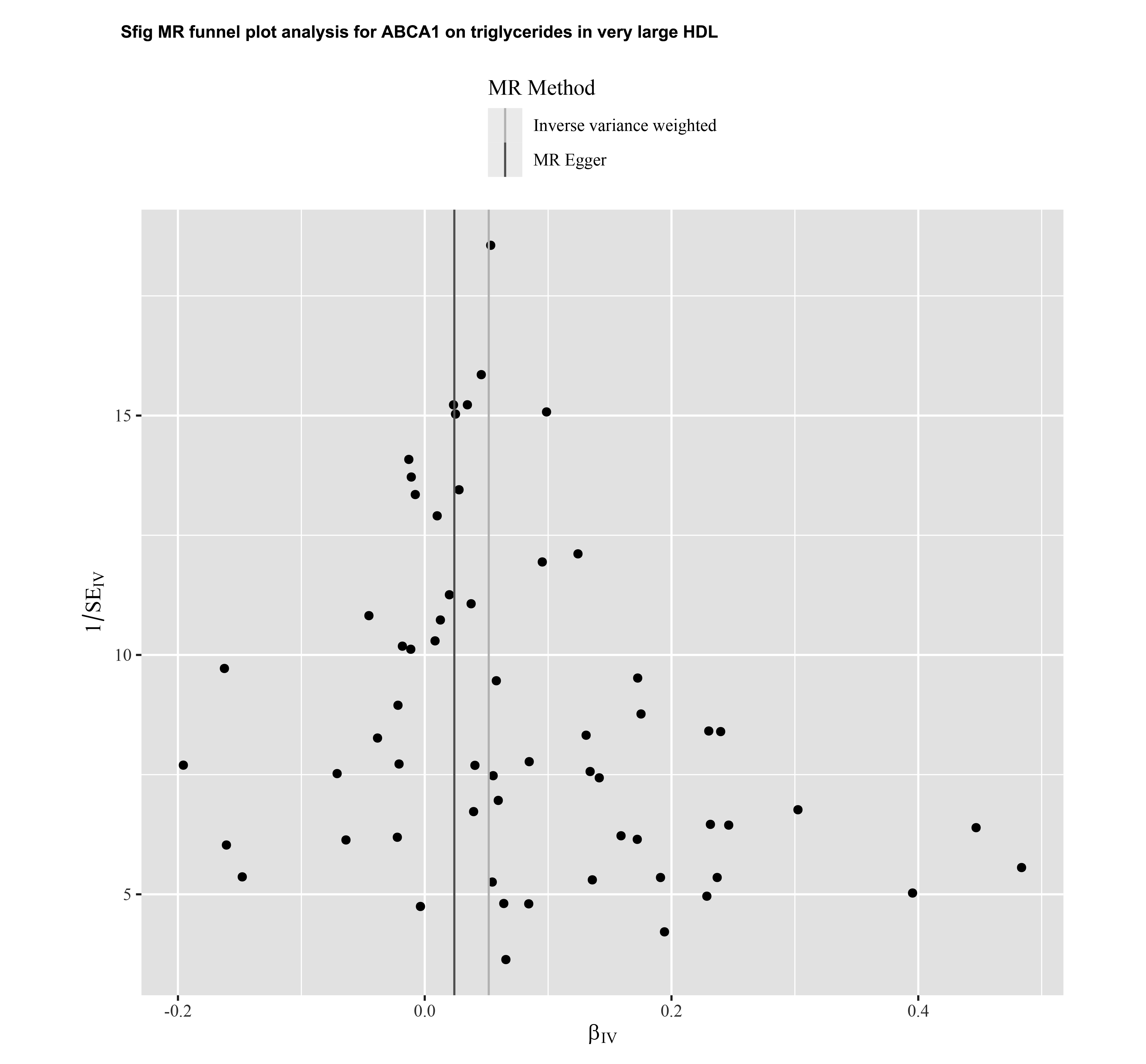

Supplement: Supplementary file 1 — Supplementary Information 1. [file 41598_2025_93644_MOESM1_ESM.zip › the funnel plot/Sfig MR funnel plot analysis for ABCA1 on triglycerides in very large HDL.tif]

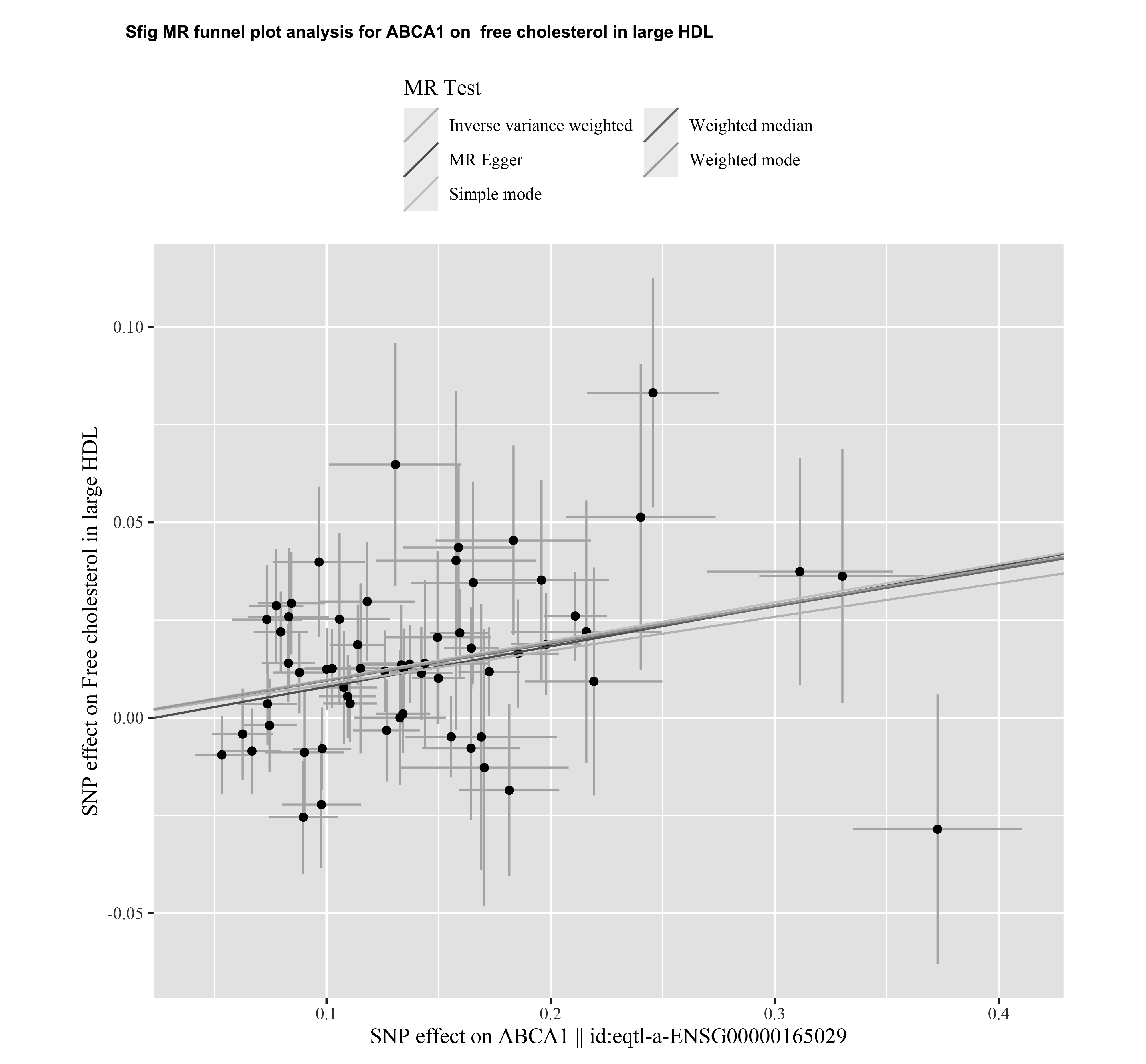

Supplement: Supplementary file 1 — Supplementary Information 1. [file 41598_2025_93644_MOESM1_ESM.zip › the scatter plot/Sfig MR funnel plot analysis for ABCA1 on free cholesterol in large HDL.tif]

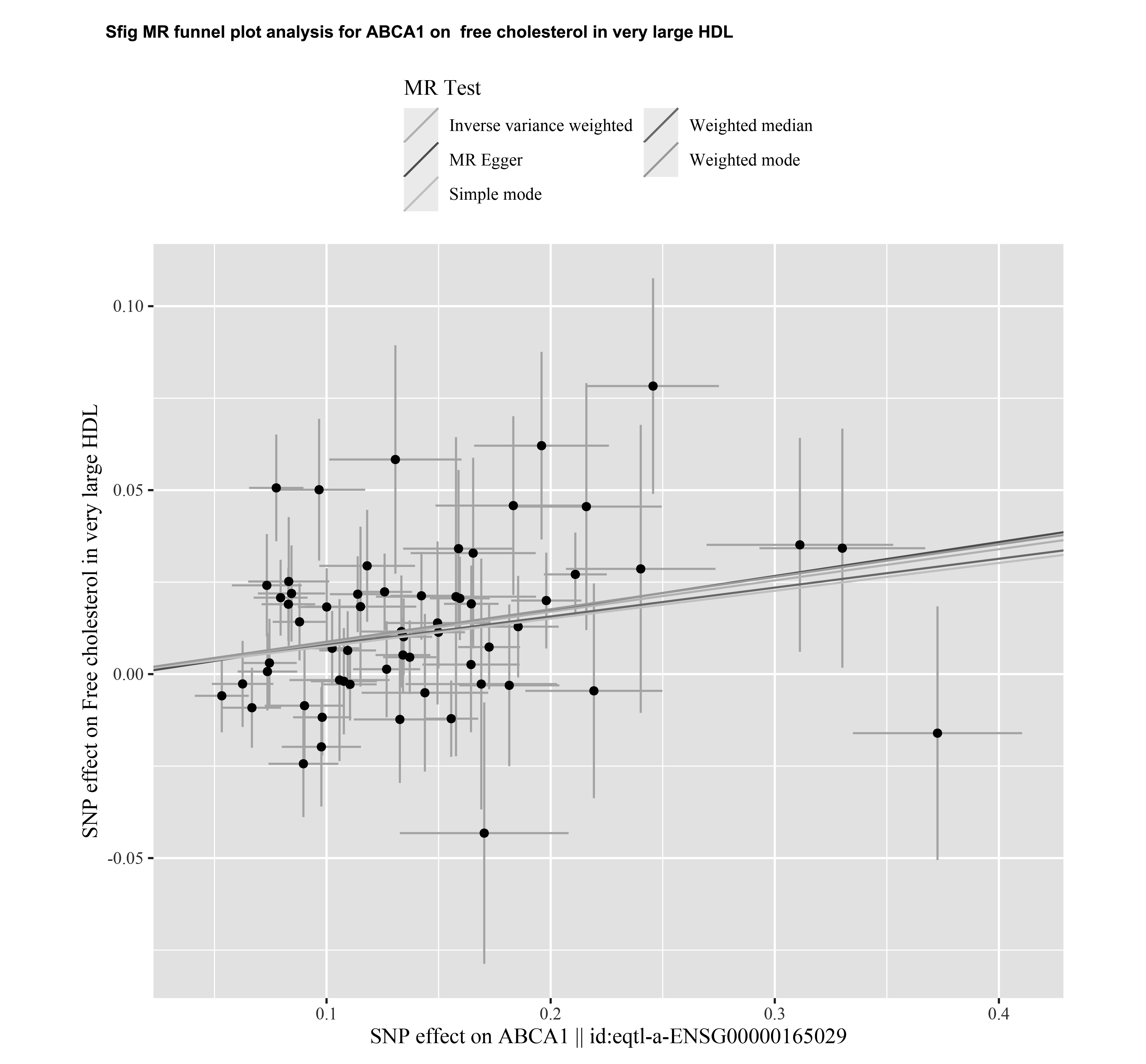

Supplement: Supplementary file 1 — Supplementary Information 1. [file 41598_2025_93644_MOESM1_ESM.zip › the scatter plot/Sfig MR funnel plot analysis for ABCA1 on free cholesterol in very large HDL.tif]

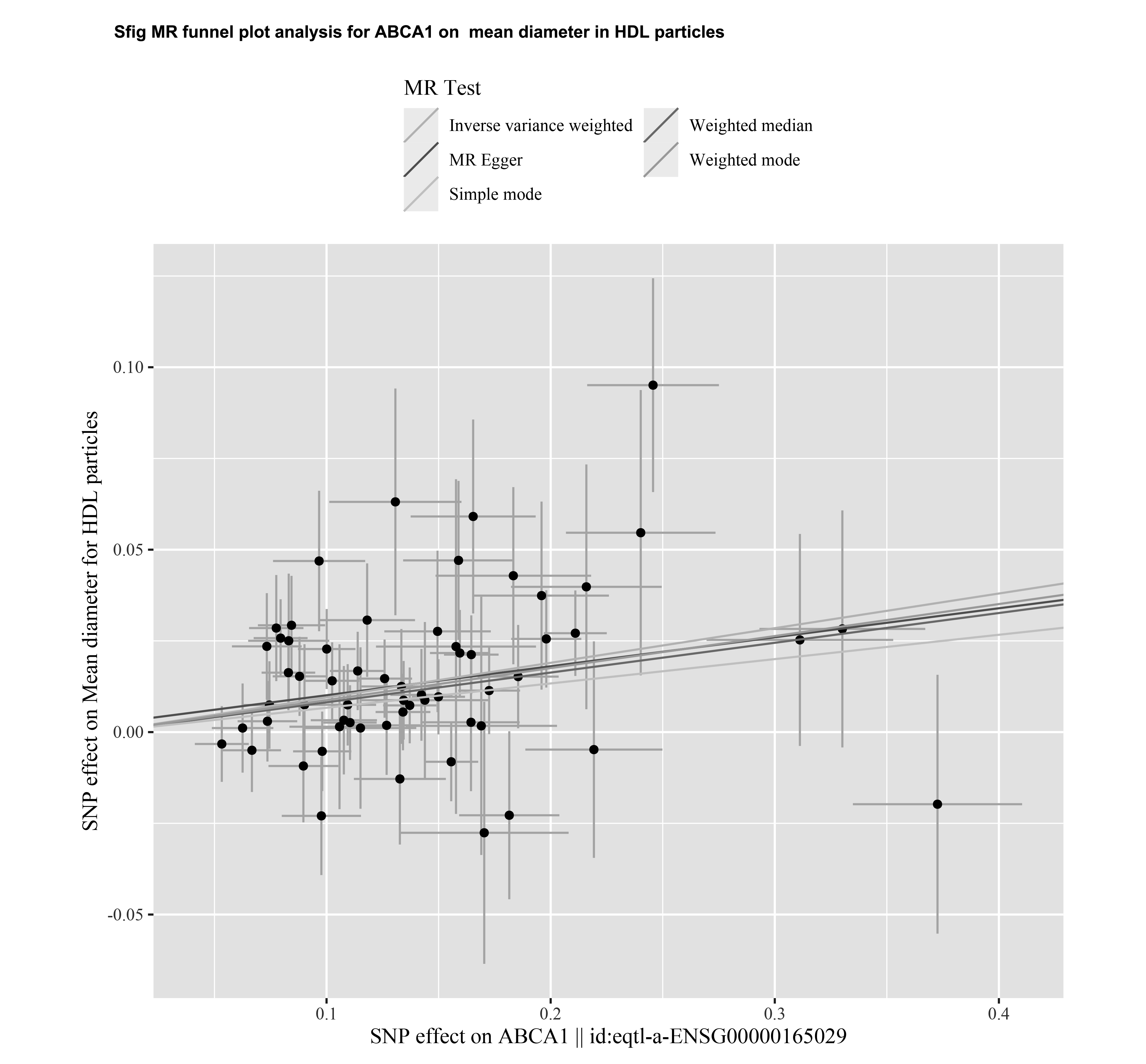

Supplement: Supplementary file 1 — Supplementary Information 1. [file 41598_2025_93644_MOESM1_ESM.zip › the scatter plot/Sfig MR funnel plot analysis for ABCA1 on mean diameter in HDL particles.tif]

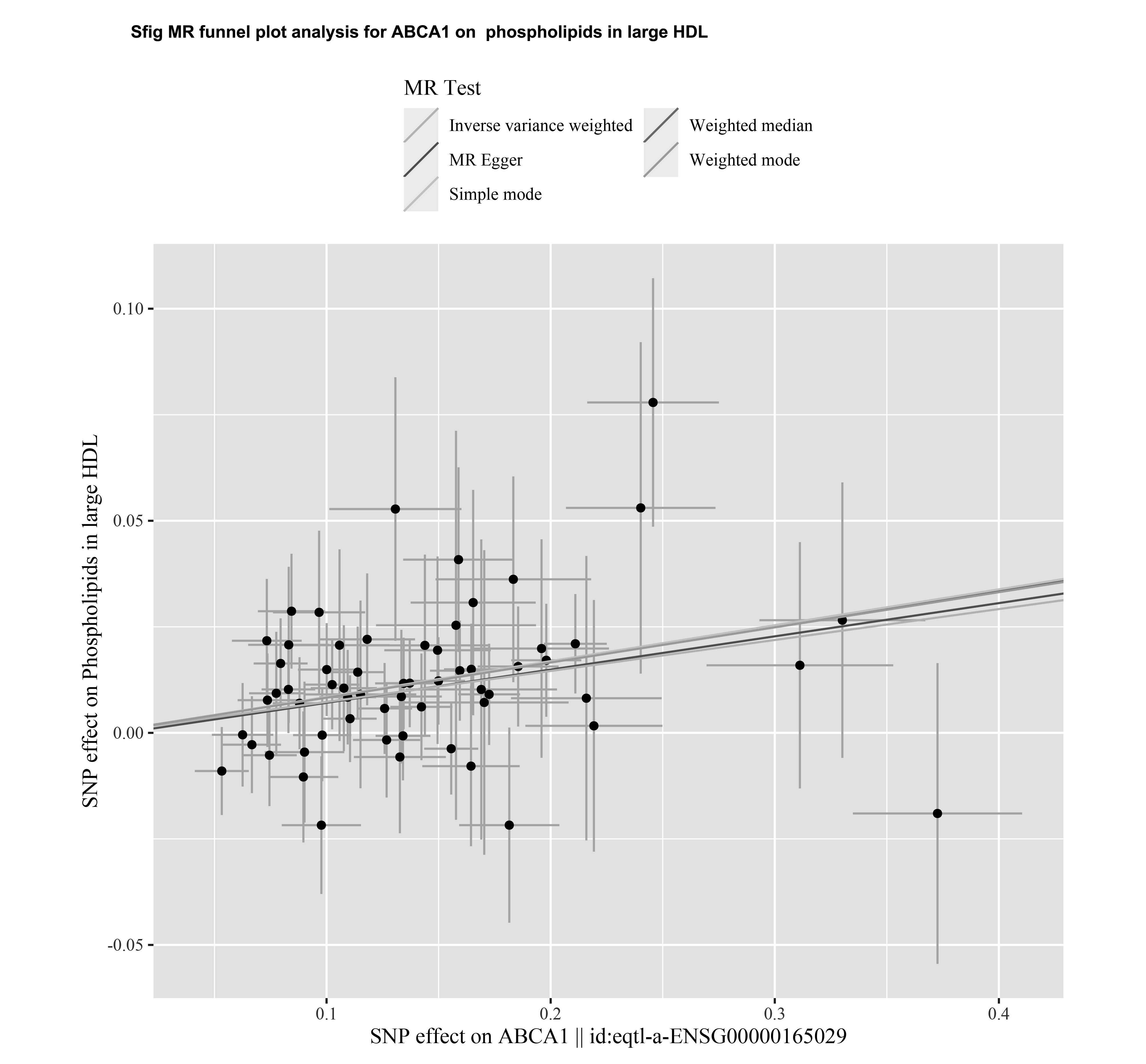

Supplement: Supplementary file 1 — Supplementary Information 1. [file 41598_2025_93644_MOESM1_ESM.zip › the scatter plot/Sfig MR funnel plot analysis for ABCA1 on phospholipids in large HDL.tif]

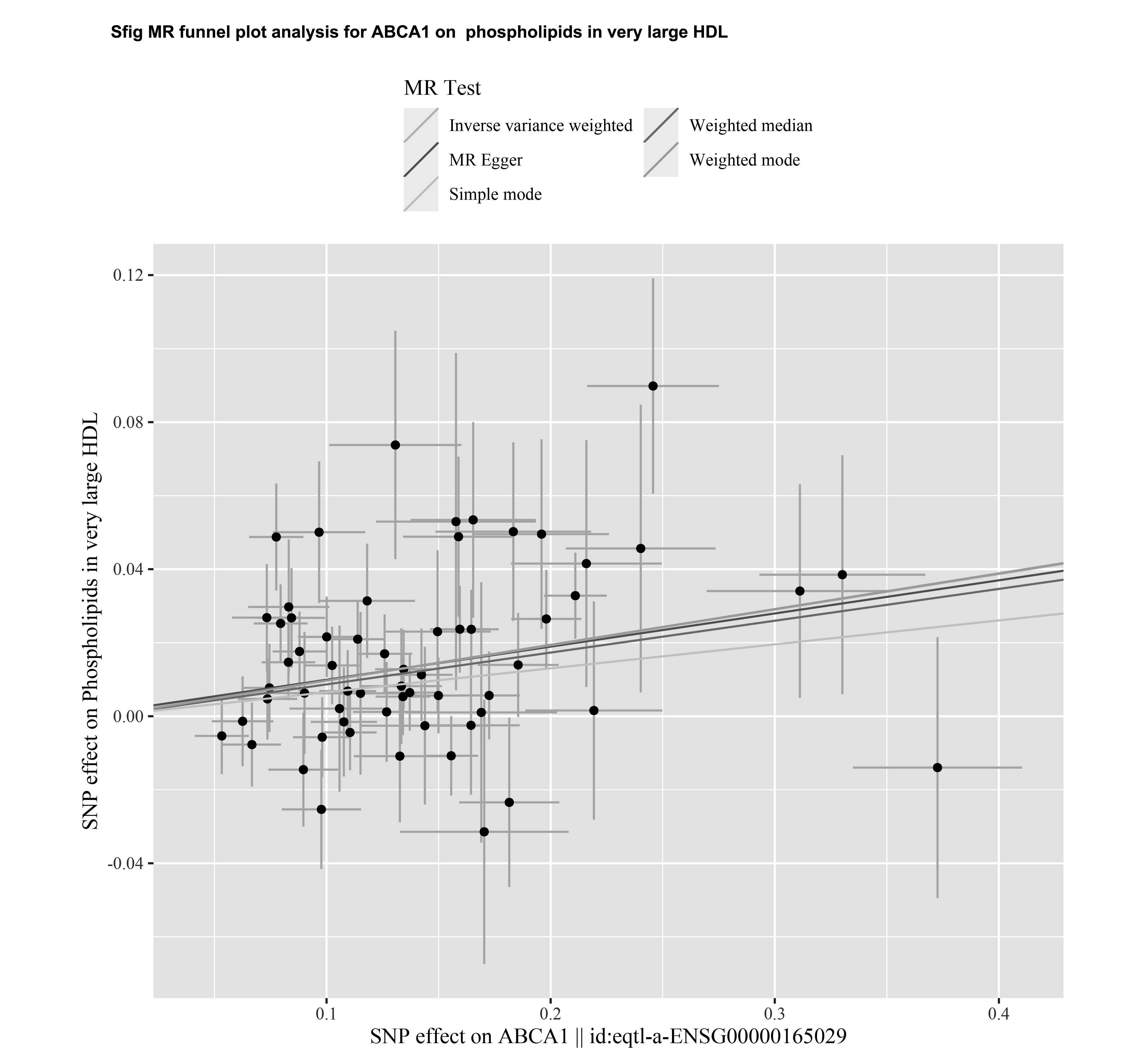

Supplement: Supplementary file 1 — Supplementary Information 1. [file 41598_2025_93644_MOESM1_ESM.zip › the scatter plot/Sfig MR funnel plot analysis for ABCA1 on phospholipids in very large HDL.tif]

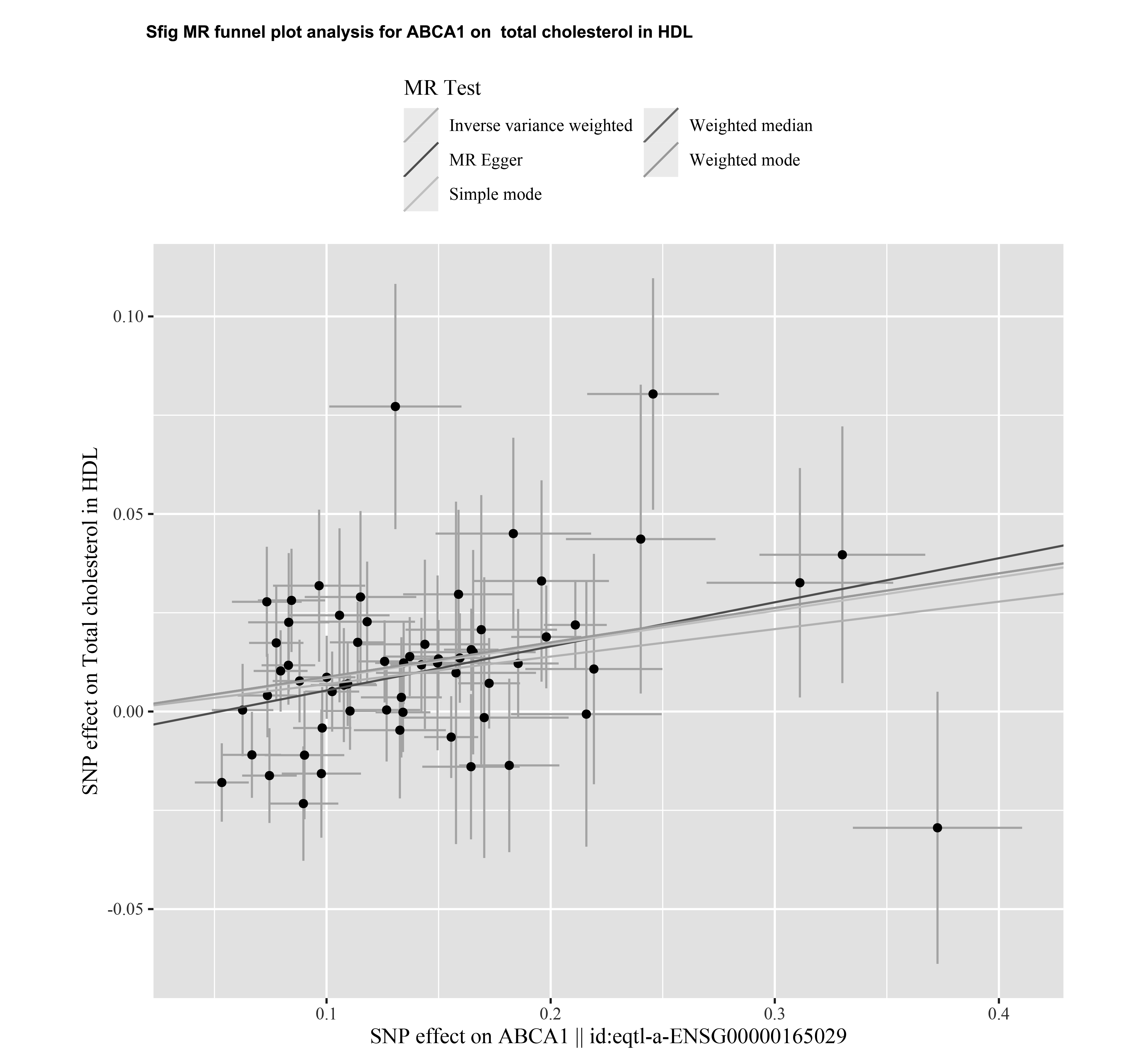

Supplement: Supplementary file 1 — Supplementary Information 1. [file 41598_2025_93644_MOESM1_ESM.zip › the scatter plot/Sfig MR funnel plot analysis for ABCA1 on total cholesterol in HDL.tif]

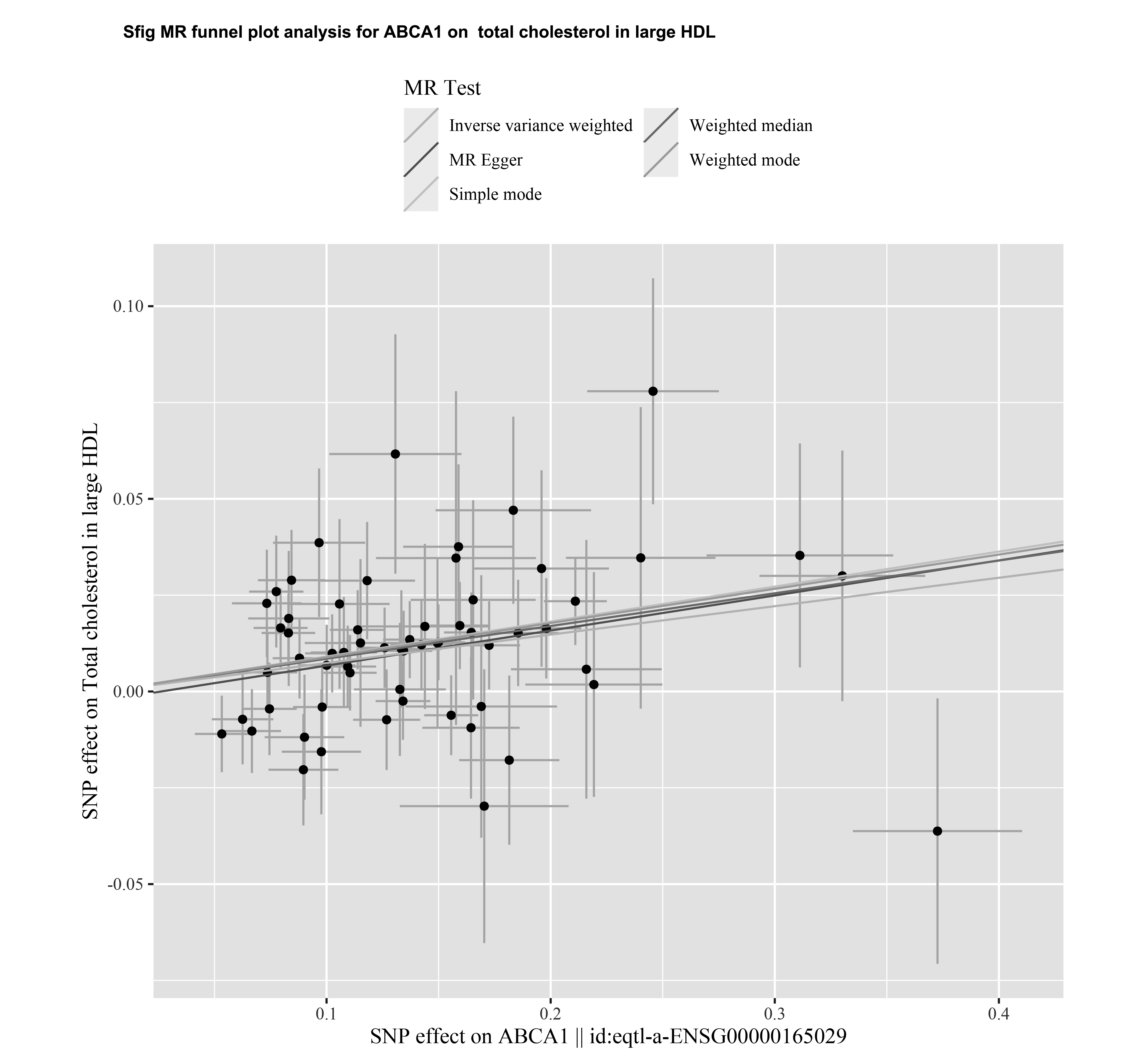

Supplement: Supplementary file 1 — Supplementary Information 1. [file 41598_2025_93644_MOESM1_ESM.zip › the scatter plot/Sfig MR funnel plot analysis for ABCA1 on total cholesterol in large HDL.tif]

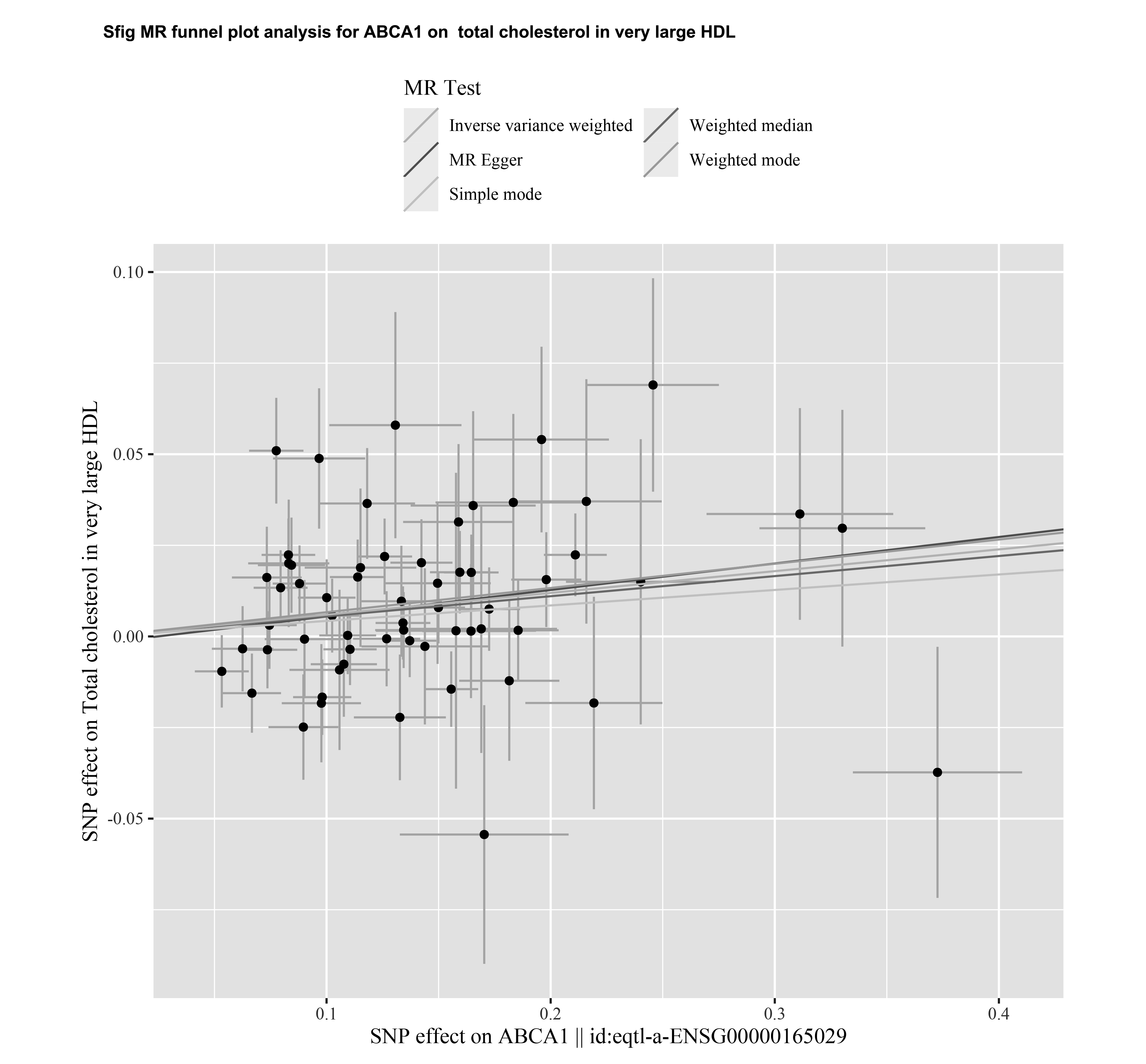

Supplement: Supplementary file 1 — Supplementary Information 1. [file 41598_2025_93644_MOESM1_ESM.zip › the scatter plot/Sfig MR funnel plot analysis for ABCA1 on total cholesterol in very large HDL.tif]

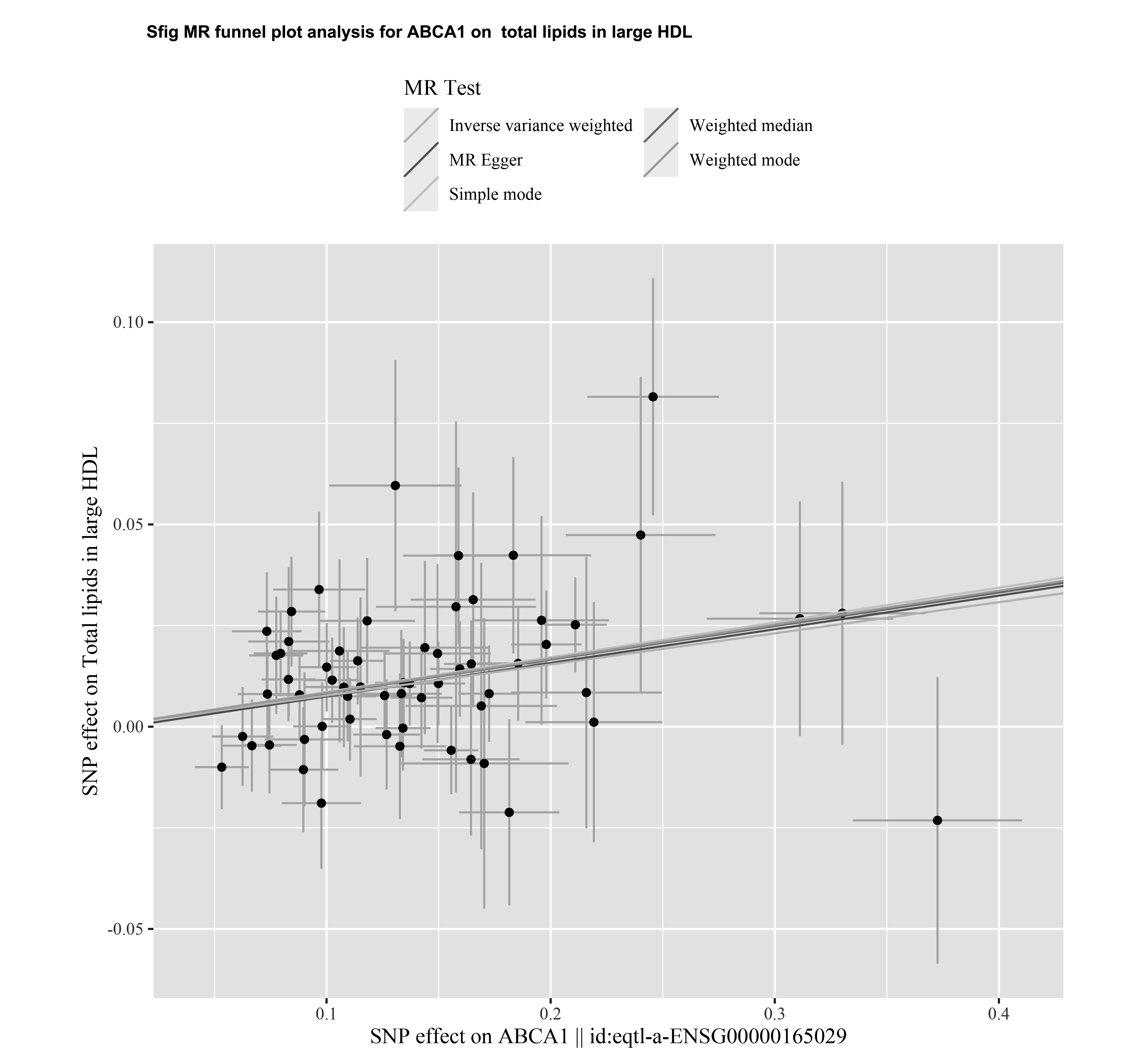

Supplement: Supplementary file 1 — Supplementary Information 1. [file 41598_2025_93644_MOESM1_ESM.zip › the scatter plot/Sfig MR funnel plot analysis for ABCA1 on total lipids in large HDL.tif]

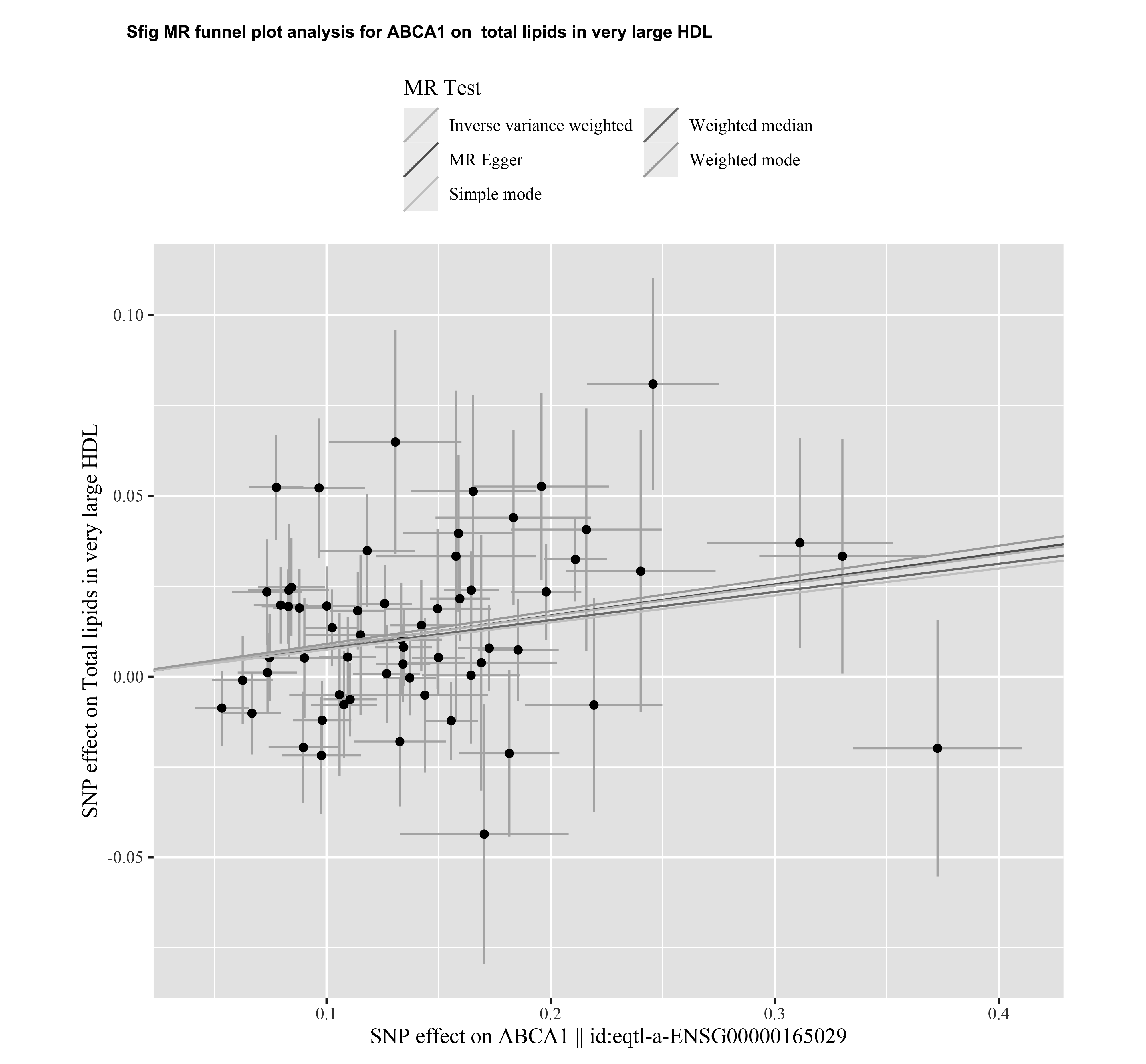

Supplement: Supplementary file 1 — Supplementary Information 1. [file 41598_2025_93644_MOESM1_ESM.zip › the scatter plot/Sfig MR funnel plot analysis for ABCA1 on total lipids in very large HDL.tif]

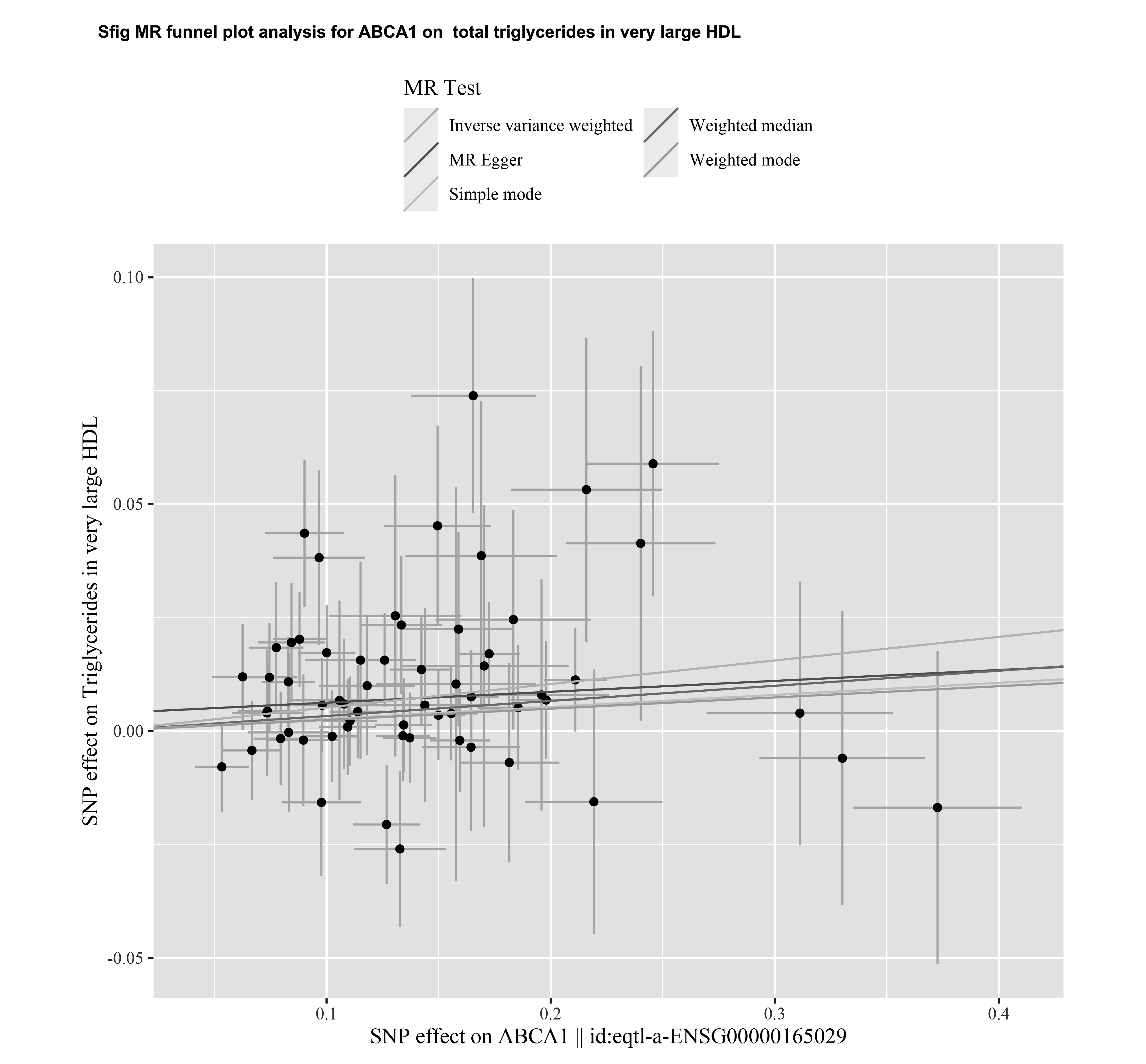

Supplement: Supplementary file 1 — Supplementary Information 1. [file 41598_2025_93644_MOESM1_ESM.zip › the scatter plot/Sfig MR funnel plot analysis for ABCA1 on total triglycerides in very large HDL.tif]

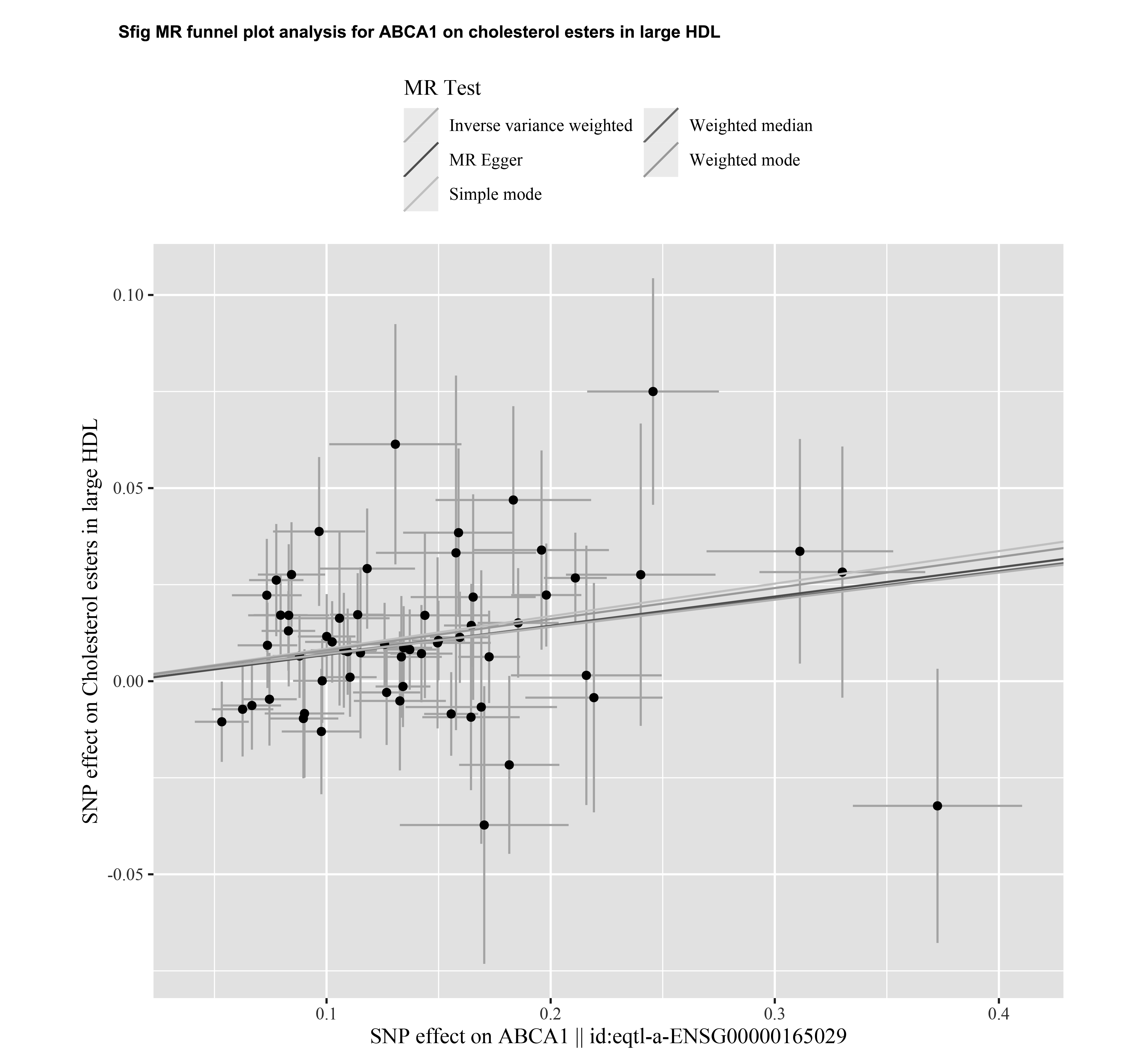

Supplement: Supplementary file 1 — Supplementary Information 1. [file 41598_2025_93644_MOESM1_ESM.zip › the scatter plot/Sfig MR funnel plot analysis for ABCA1 on cholesterol esters in large HDL.tif]

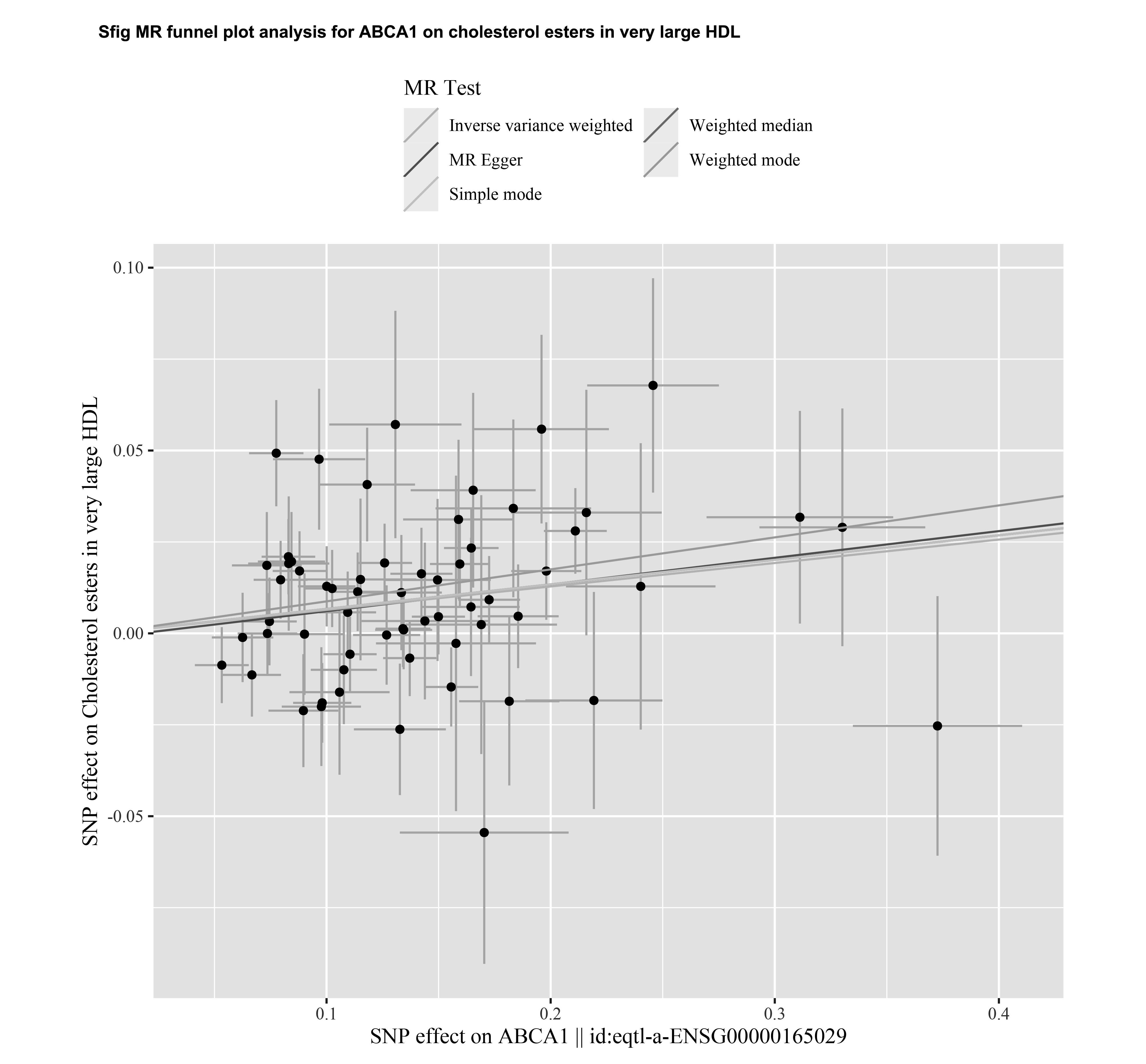

Supplement: Supplementary file 1 — Supplementary Information 1. [file 41598_2025_93644_MOESM1_ESM.zip › the scatter plot/Sfig MR funnel plot analysis for ABCA1 on cholesterol esters in very large HDL.tif]

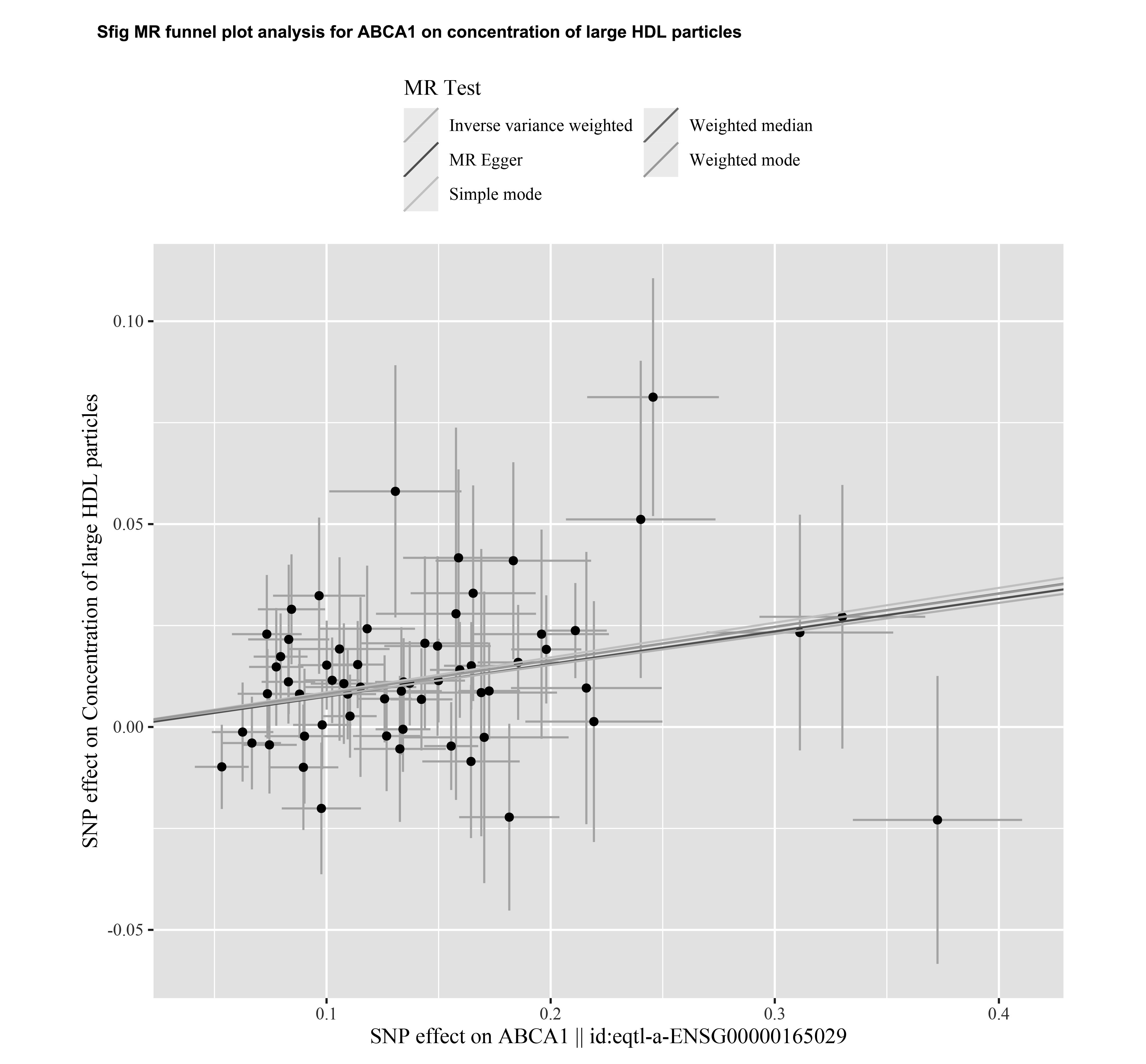

Supplement: Supplementary file 1 — Supplementary Information 1. [file 41598_2025_93644_MOESM1_ESM.zip › the scatter plot/Sfig MR funnel plot analysis for ABCA1 on concentration of large HDL particles.tif]

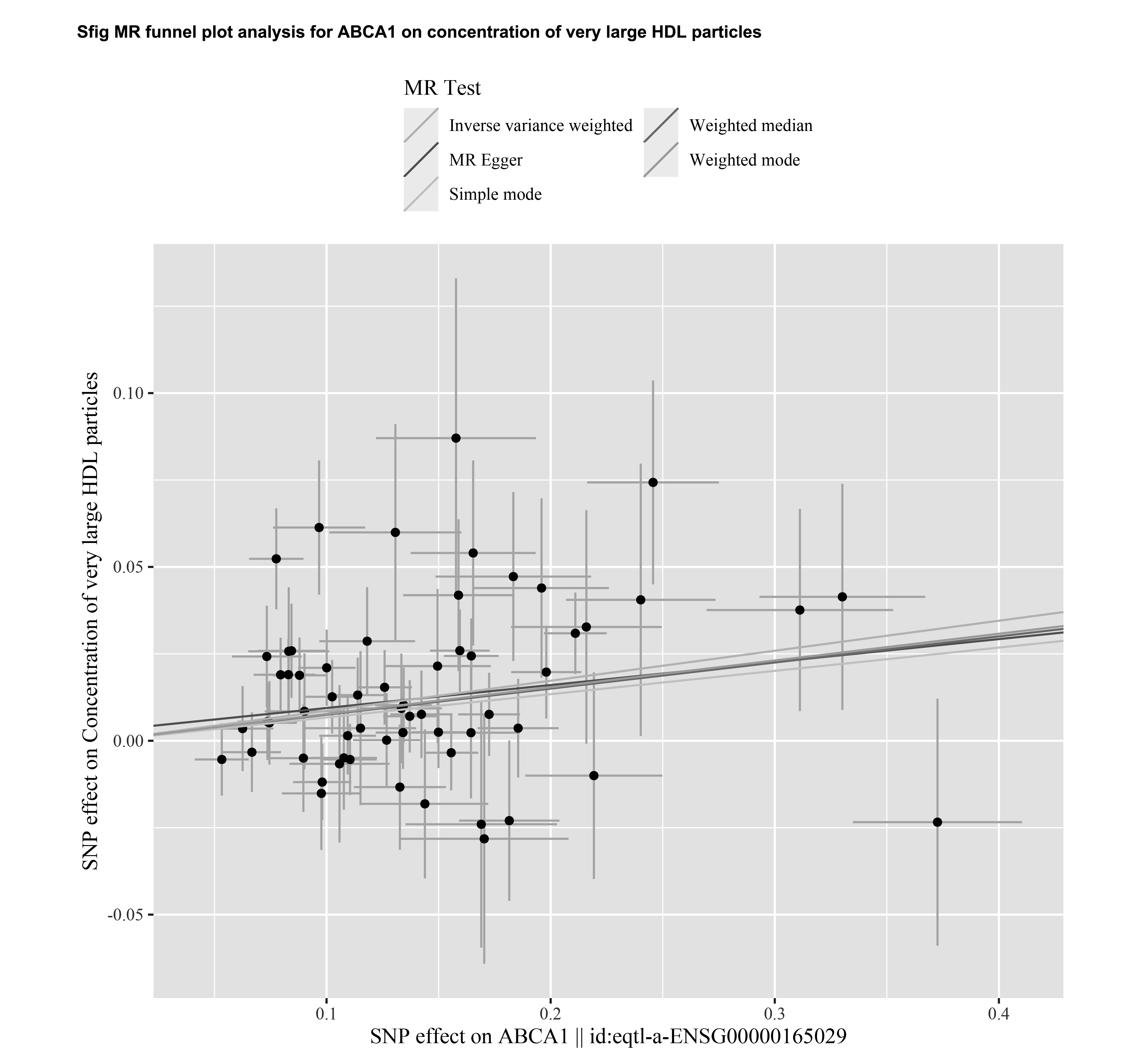

Supplement: Supplementary file 1 — Supplementary Information 1. [file 41598_2025_93644_MOESM1_ESM.zip › the scatter plot/Sfig MR funnel plot analysis for ABCA1 on concentration of very large HDL particles.tif]

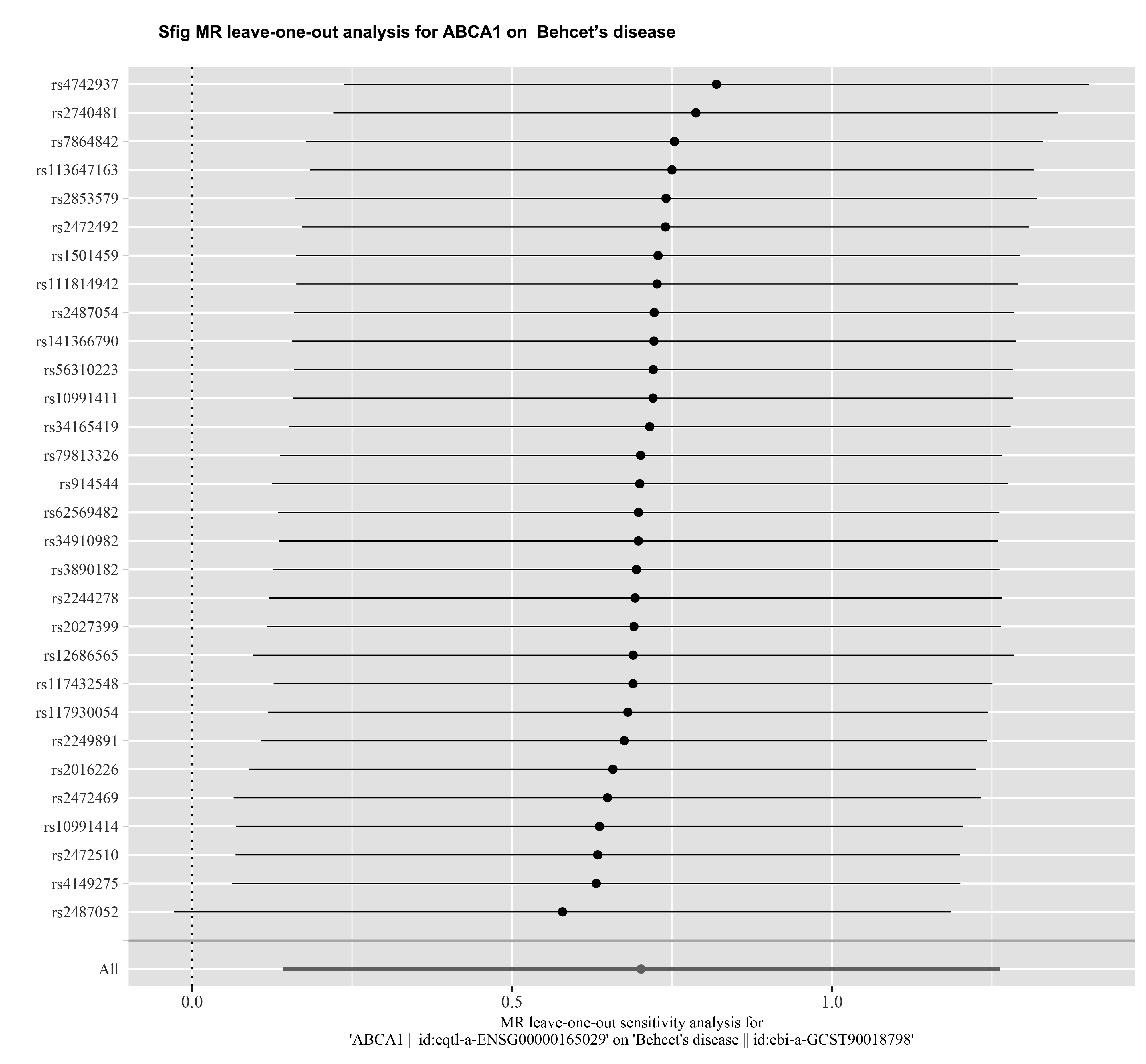

Supplement: Supplementary file 2 — Supplementary Information 2. [file 41598_2025_93644_MOESM2_ESM.zip › leave-one-out analysis/Sfig MR leave-one-out analysis for ABCA1 on Behcet’s disease.tif]

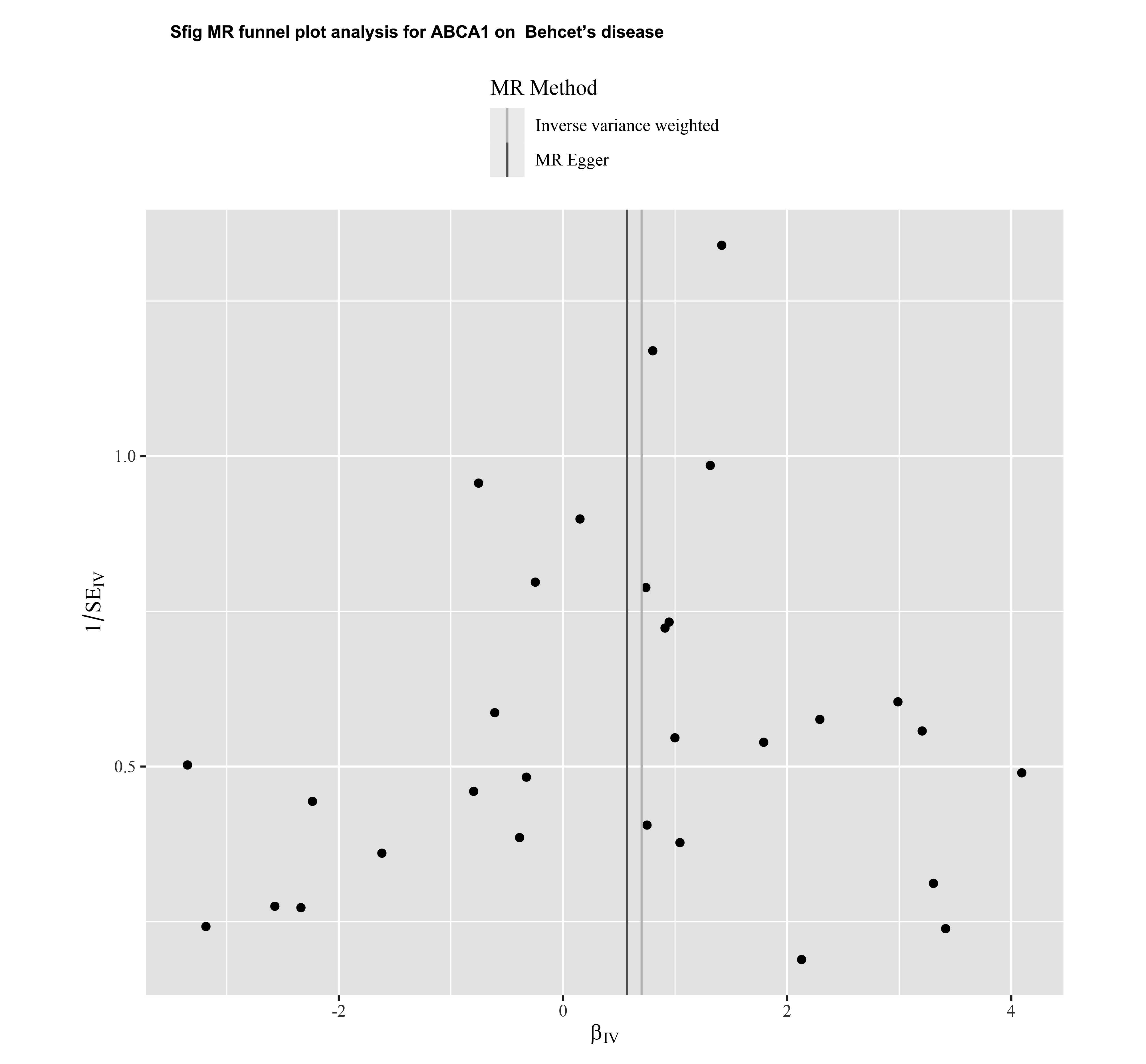

Supplement: Supplementary file 2 — Supplementary Information 2. [file 41598_2025_93644_MOESM2_ESM.zip › the funnel plot/Sfig MR funnel plot analysis for ABCA1 on Behcet’s disease.tif]

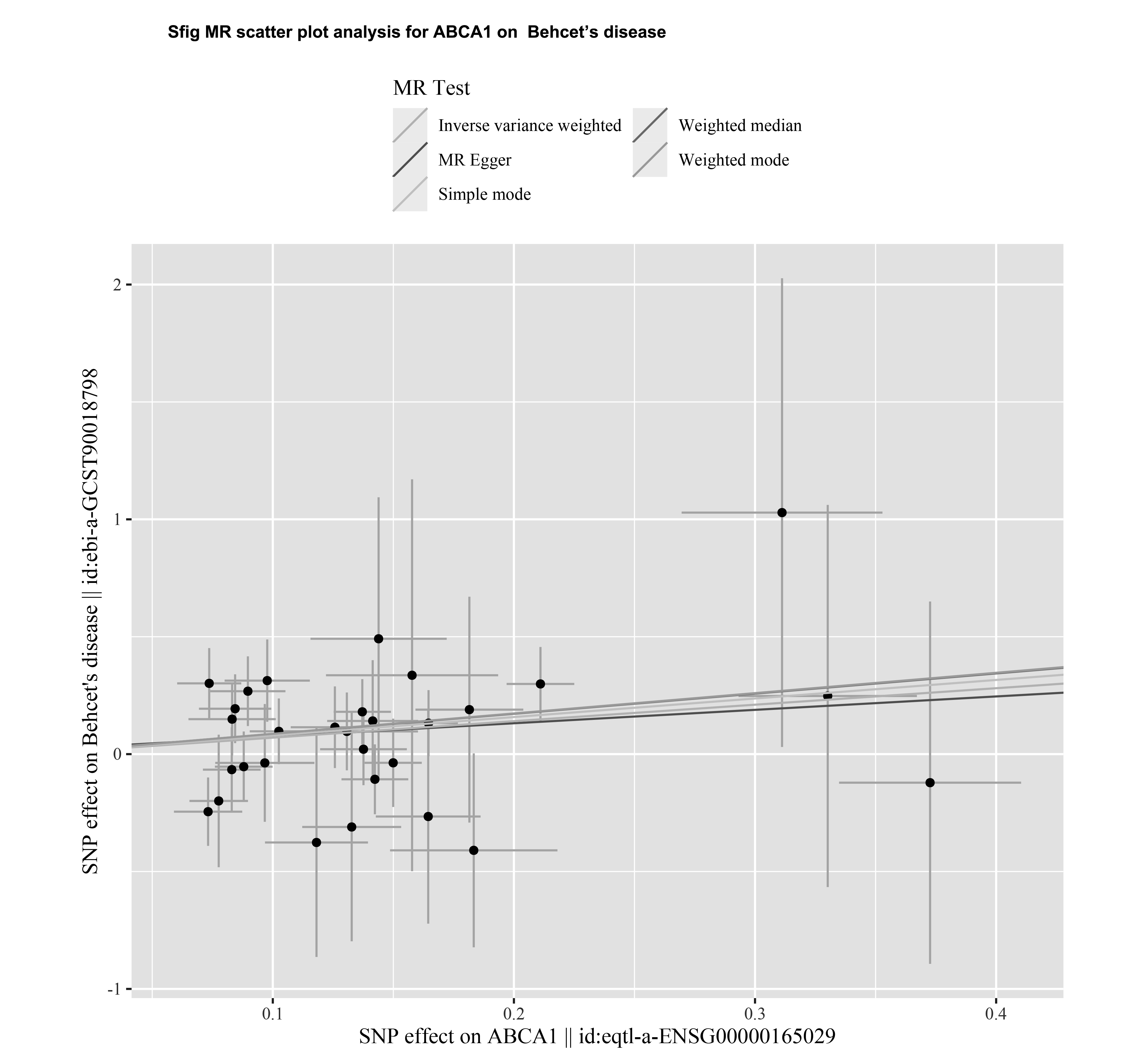

Supplement: Supplementary file 2 — Supplementary Information 2. [file 41598_2025_93644_MOESM2_ESM.zip › the scatter plot/Sfig MR scatter plot analysis for ABCA1 on Behcet’s disease.tif]

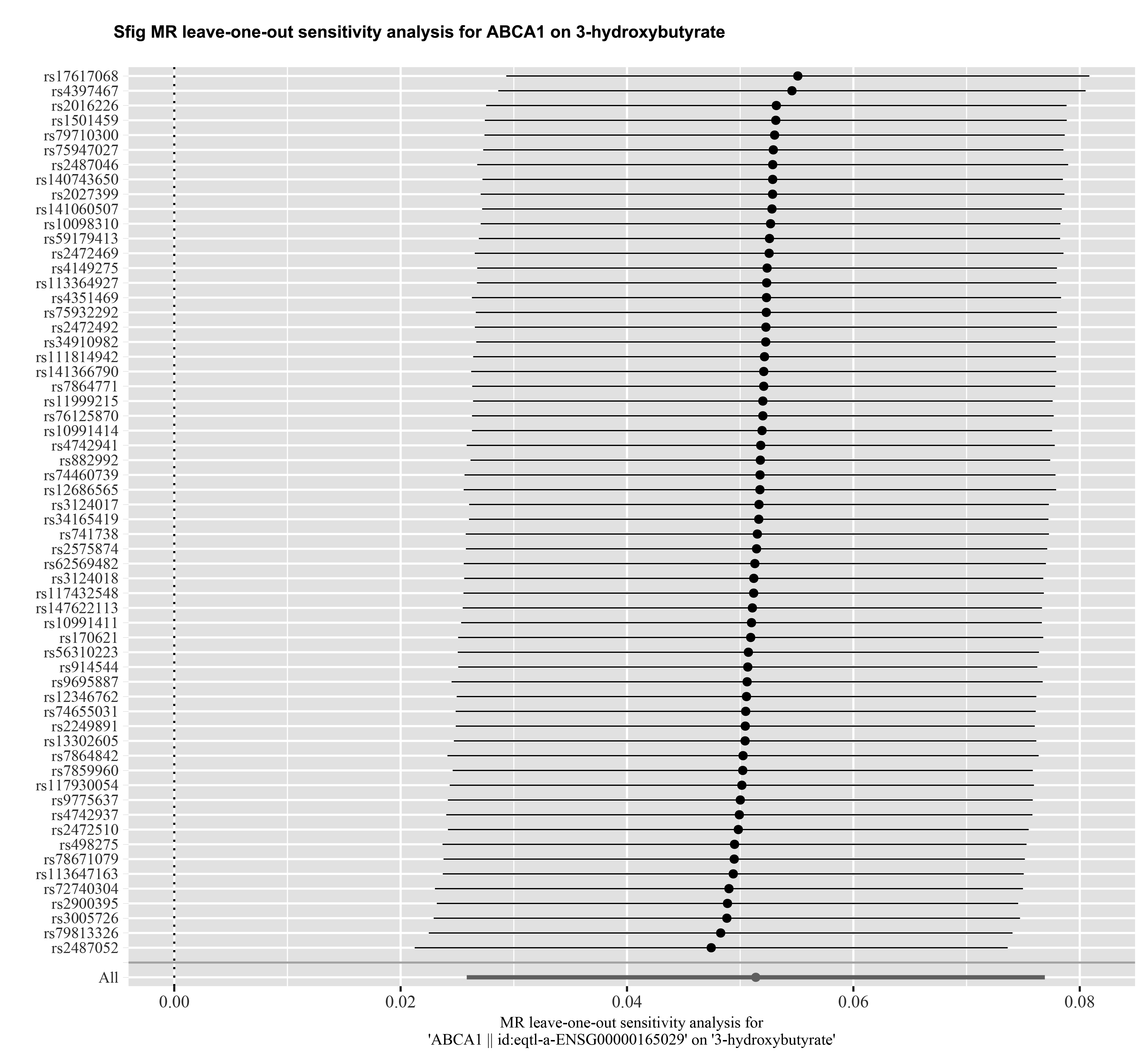

Supplement: Supplementary file 3 — Supplementary Information 3. [file 41598_2025_93644_MOESM3_ESM.zip › leave-one-out analysis/Sfig MR leave-one-out sensitivity analysis for ABCA1 on 3-hydroxybutyrate.tif]

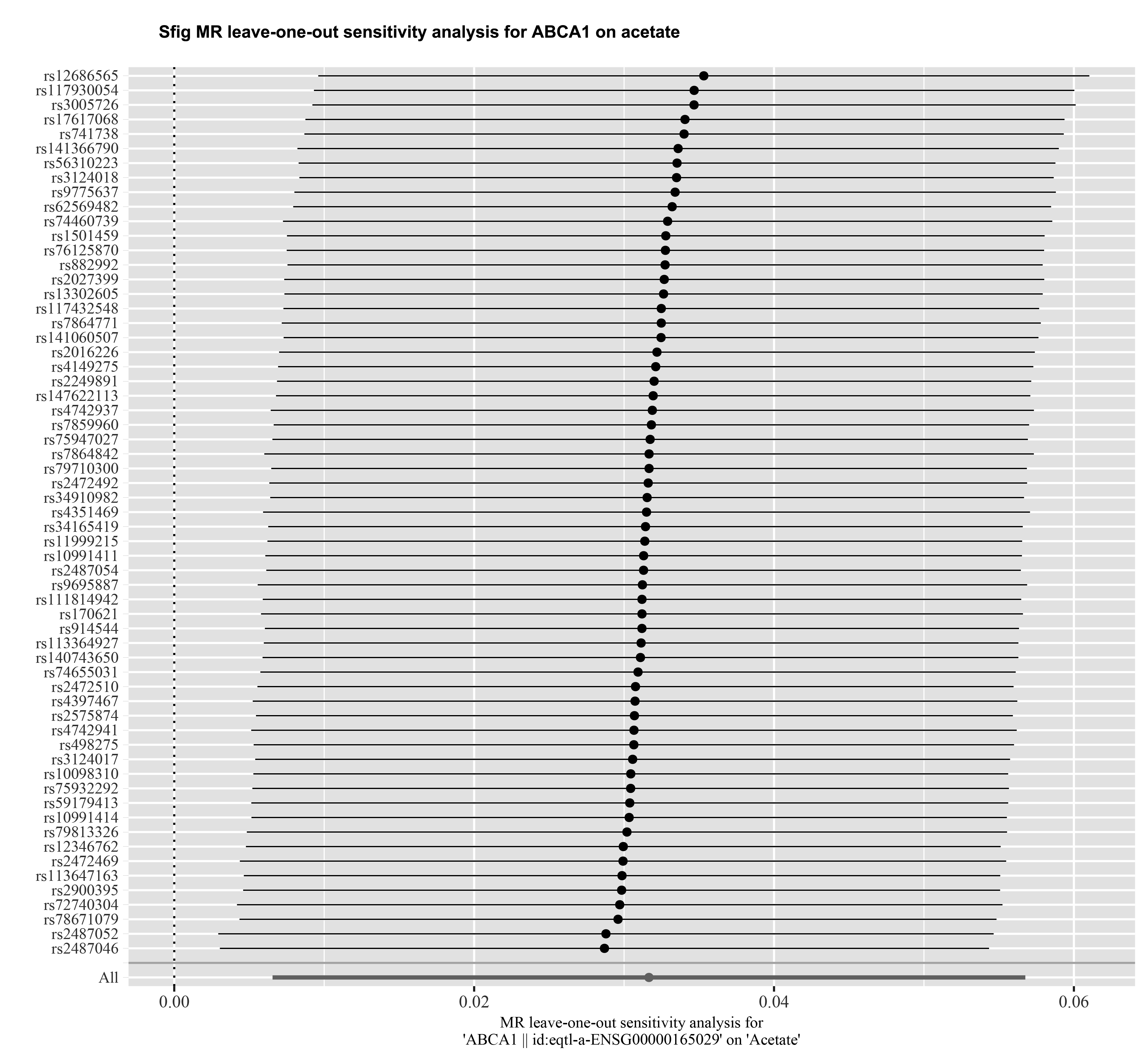

Supplement: Supplementary file 3 — Supplementary Information 3. [file 41598_2025_93644_MOESM3_ESM.zip › leave-one-out analysis/Sfig MR leave-one-out sensitivity analysis for ABCA1 on acetate.tif]

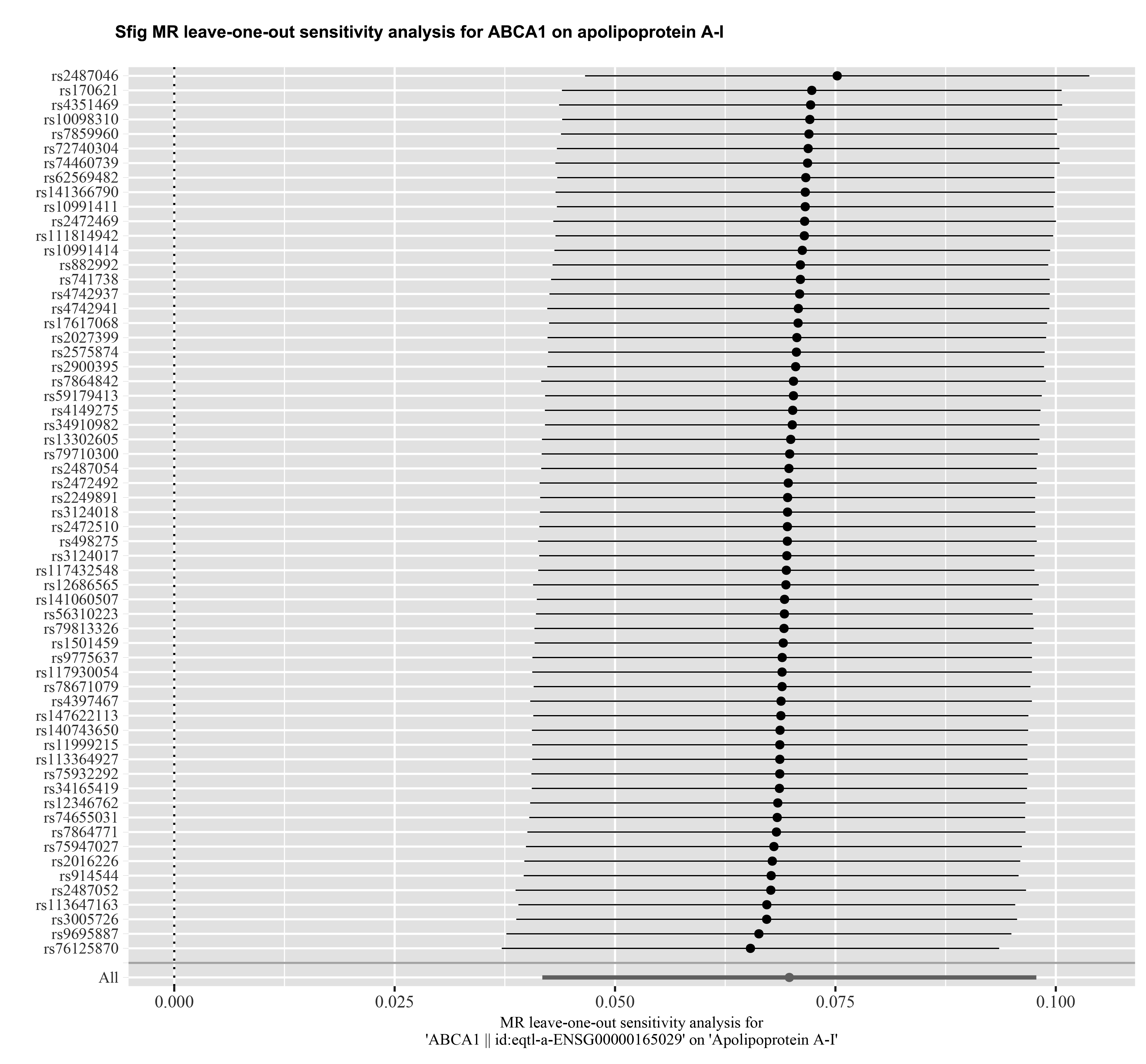

Supplement: Supplementary file 3 — Supplementary Information 3. [file 41598_2025_93644_MOESM3_ESM.zip › leave-one-out analysis/Sfig MR leave-one-out sensitivity analysis for ABCA1 on apolipoprotein A-I.tif]

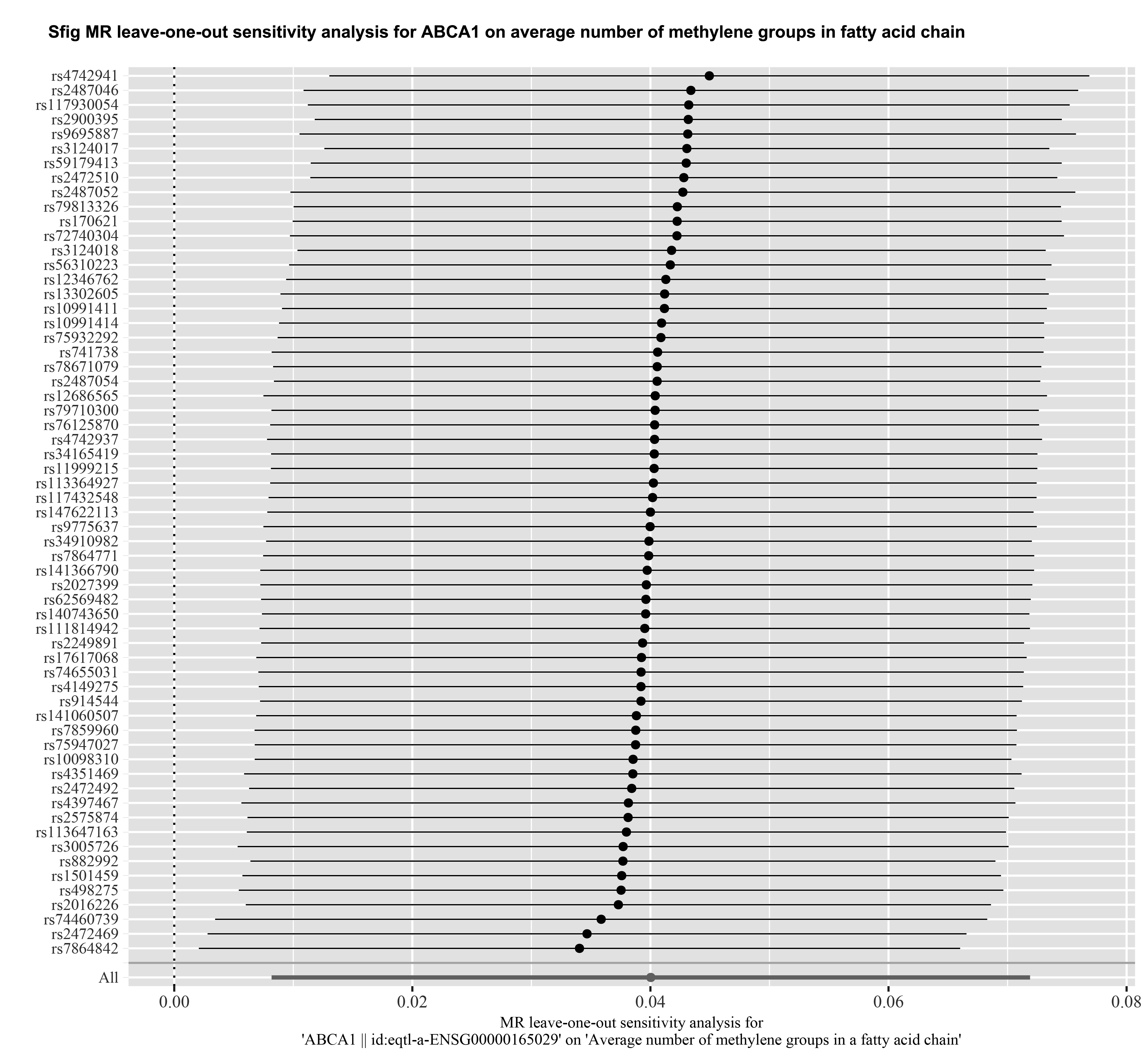

Supplement: Supplementary file 3 — Supplementary Information 3. [file 41598_2025_93644_MOESM3_ESM.zip › leave-one-out analysis/Sfig MR leave-one-out sensitivity analysis for ABCA1 on average number of methylene groups in fatty acid chain.tif]

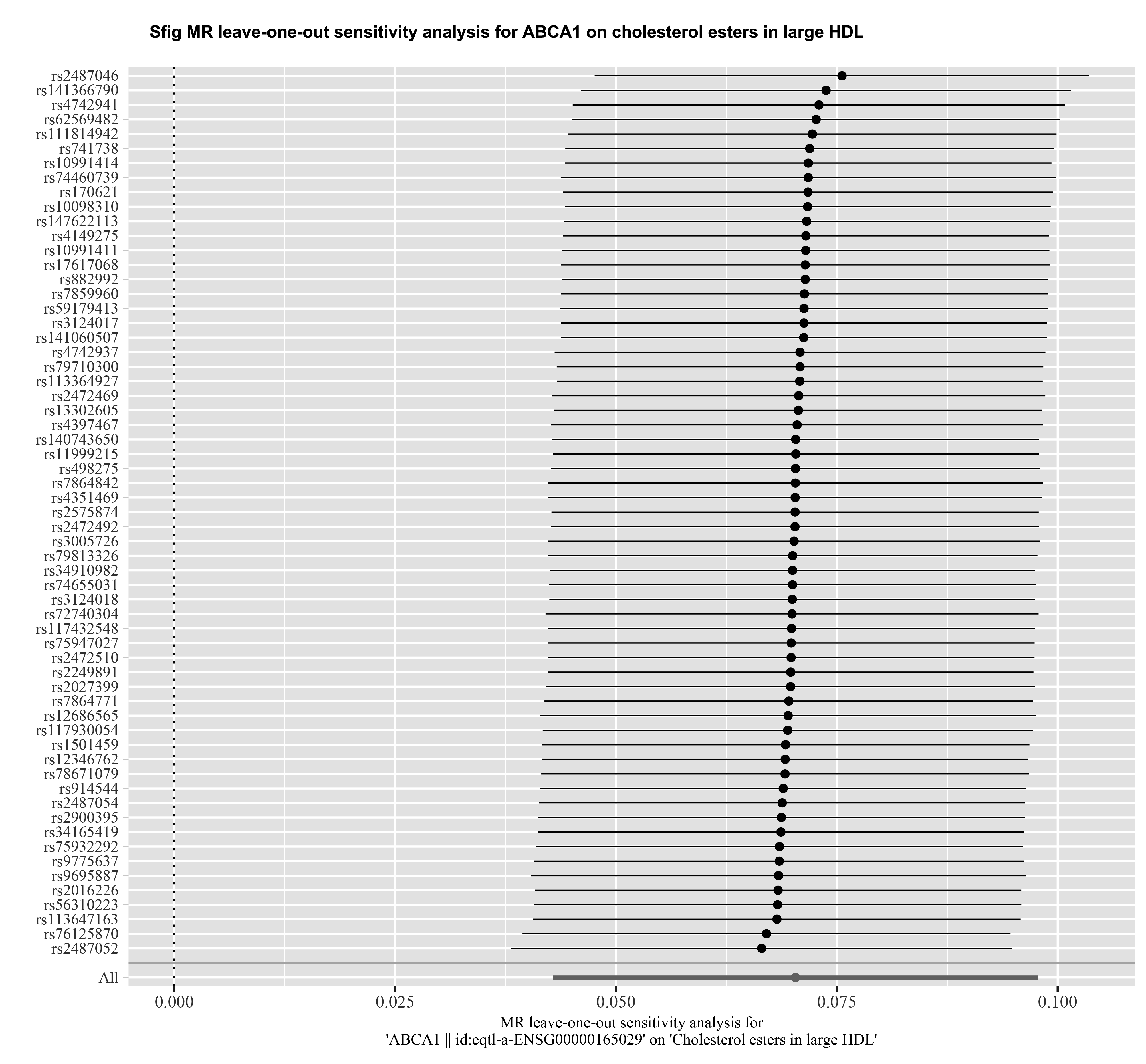

Supplement: Supplementary file 3 — Supplementary Information 3. [file 41598_2025_93644_MOESM3_ESM.zip › leave-one-out analysis/Sfig MR leave-one-out sensitivity analysis for ABCA1 on cholesterol esters in large HDL.tif]

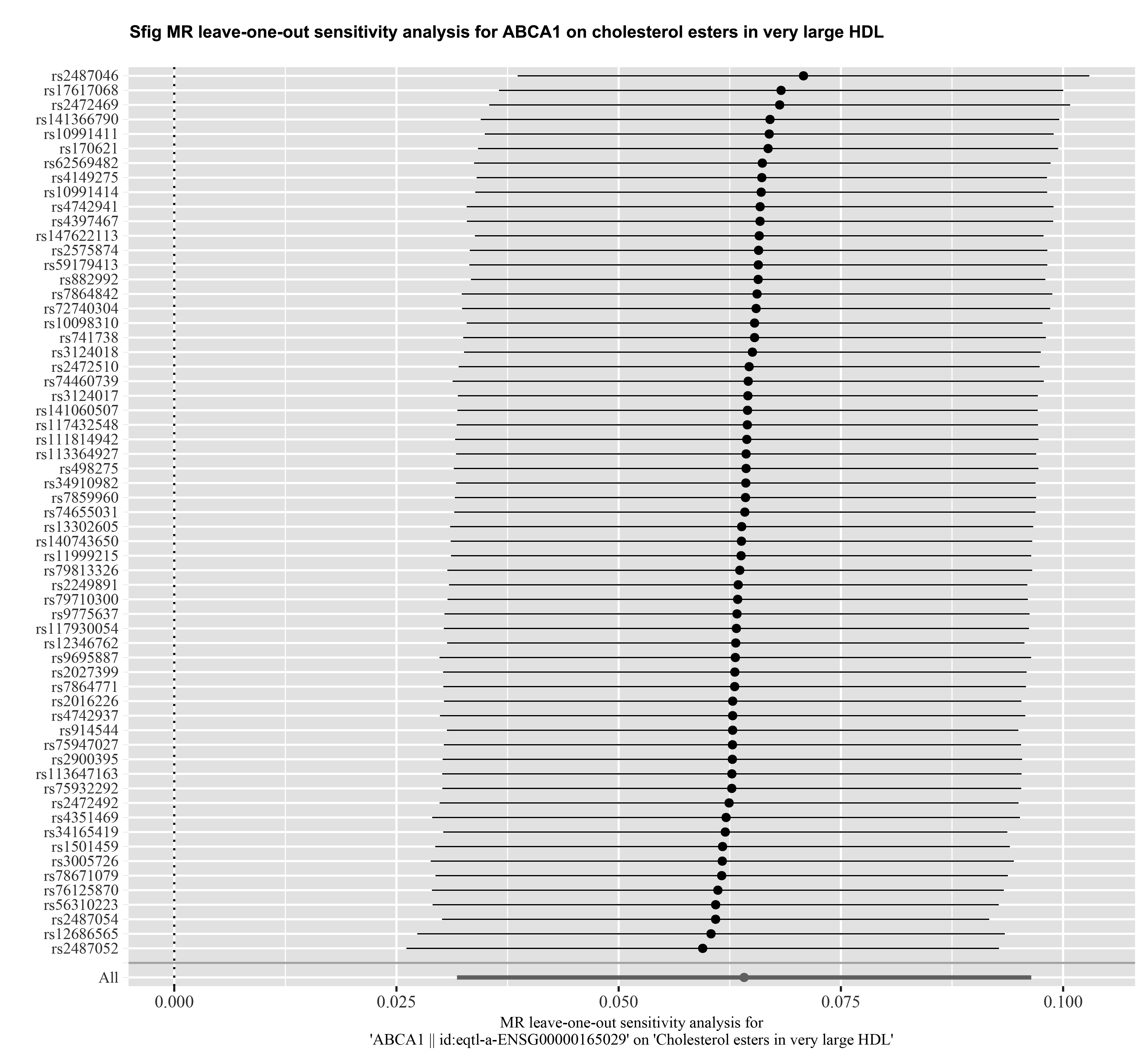

Supplement: Supplementary file 3 — Supplementary Information 3. [file 41598_2025_93644_MOESM3_ESM.zip › leave-one-out analysis/Sfig MR leave-one-out sensitivity analysis for ABCA1 on cholesterol esters in very large HDL.tif]

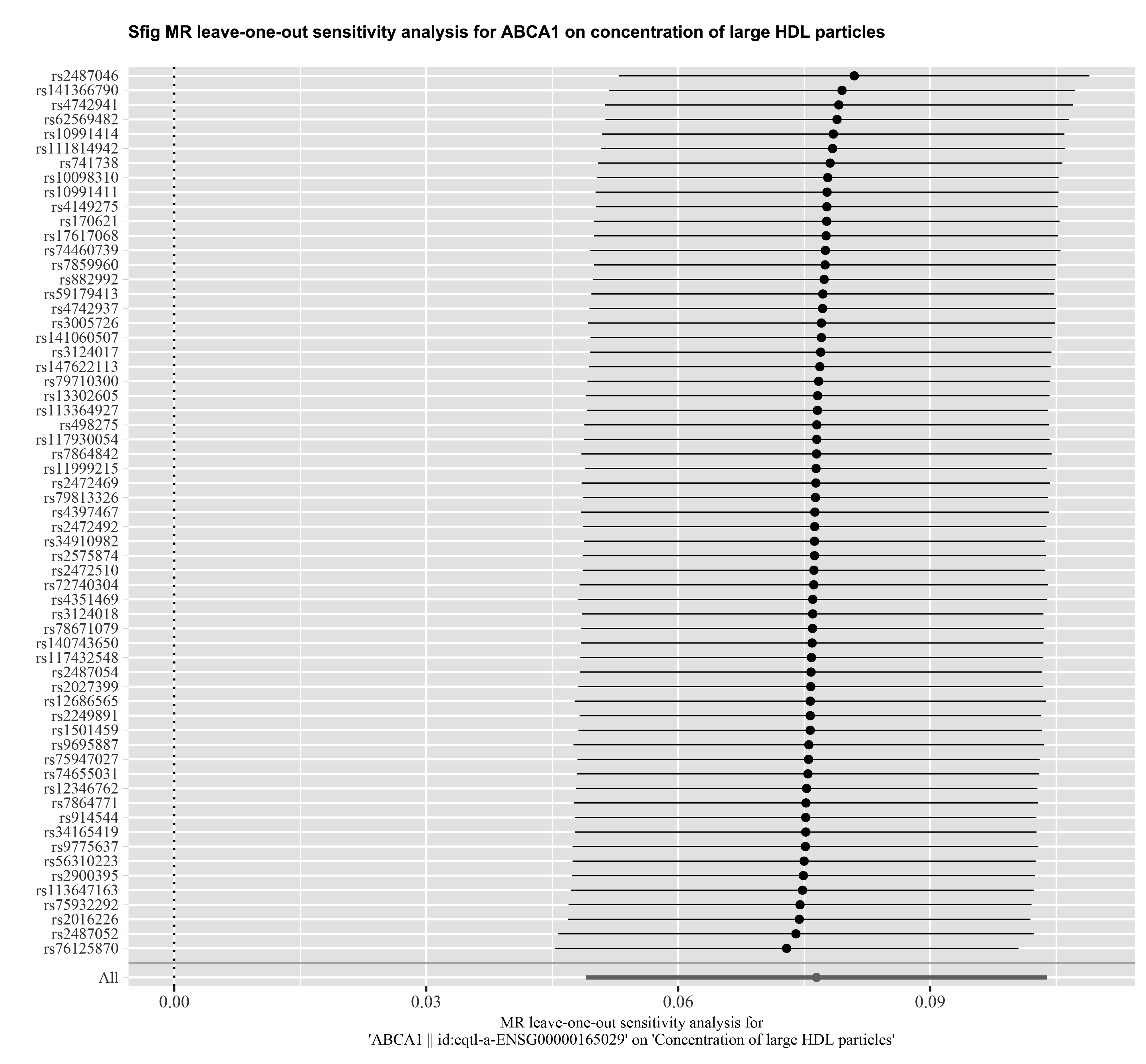

Supplement: Supplementary file 3 — Supplementary Information 3. [file 41598_2025_93644_MOESM3_ESM.zip › leave-one-out analysis/Sfig MR leave-one-out sensitivity analysis for ABCA1 on concentration of large HDL particles.tif]

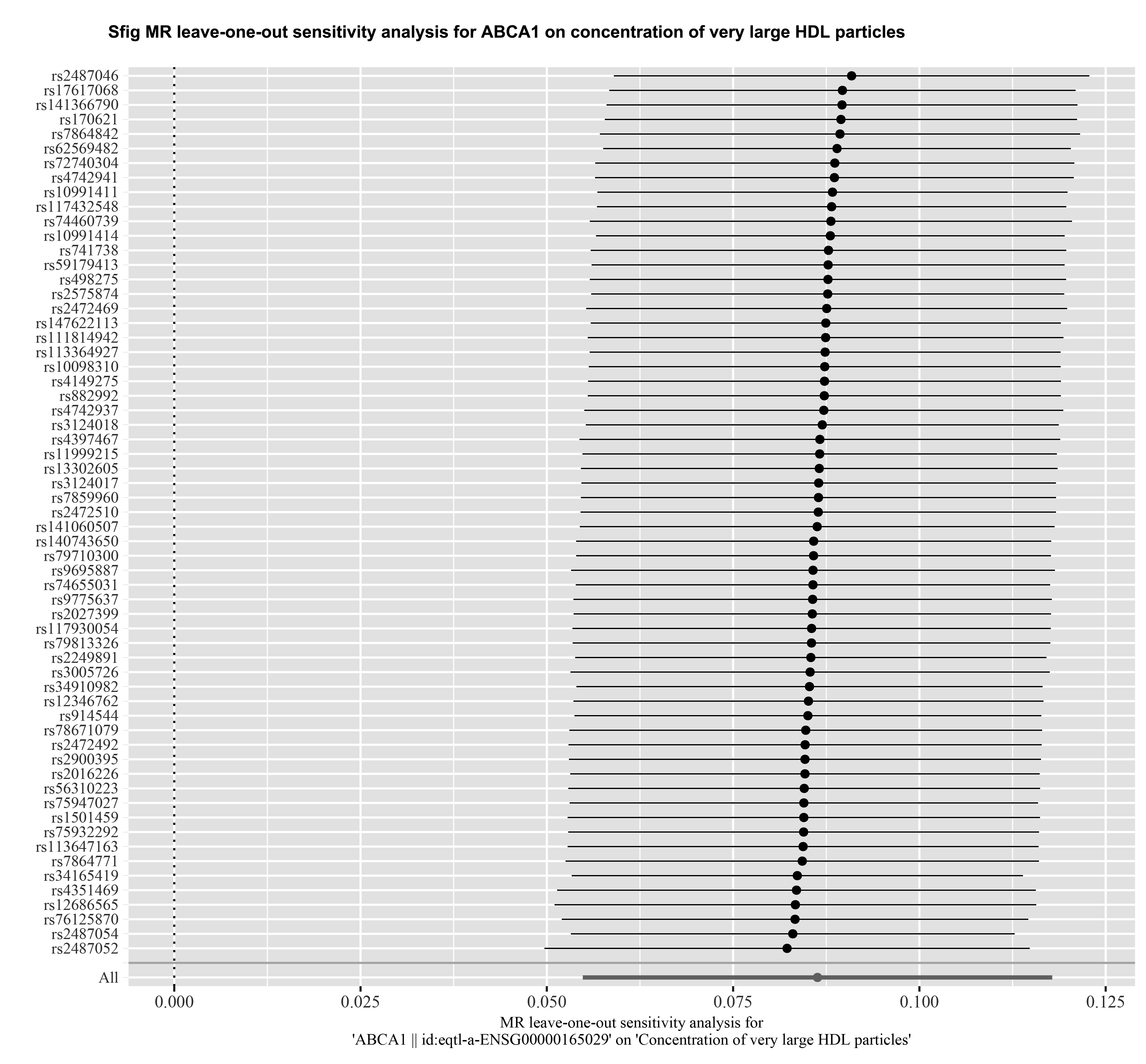

Supplement: Supplementary file 3 — Supplementary Information 3. [file 41598_2025_93644_MOESM3_ESM.zip › leave-one-out analysis/Sfig MR leave-one-out sensitivity analysis for ABCA1 on concentration of very large HDL particles.tif]

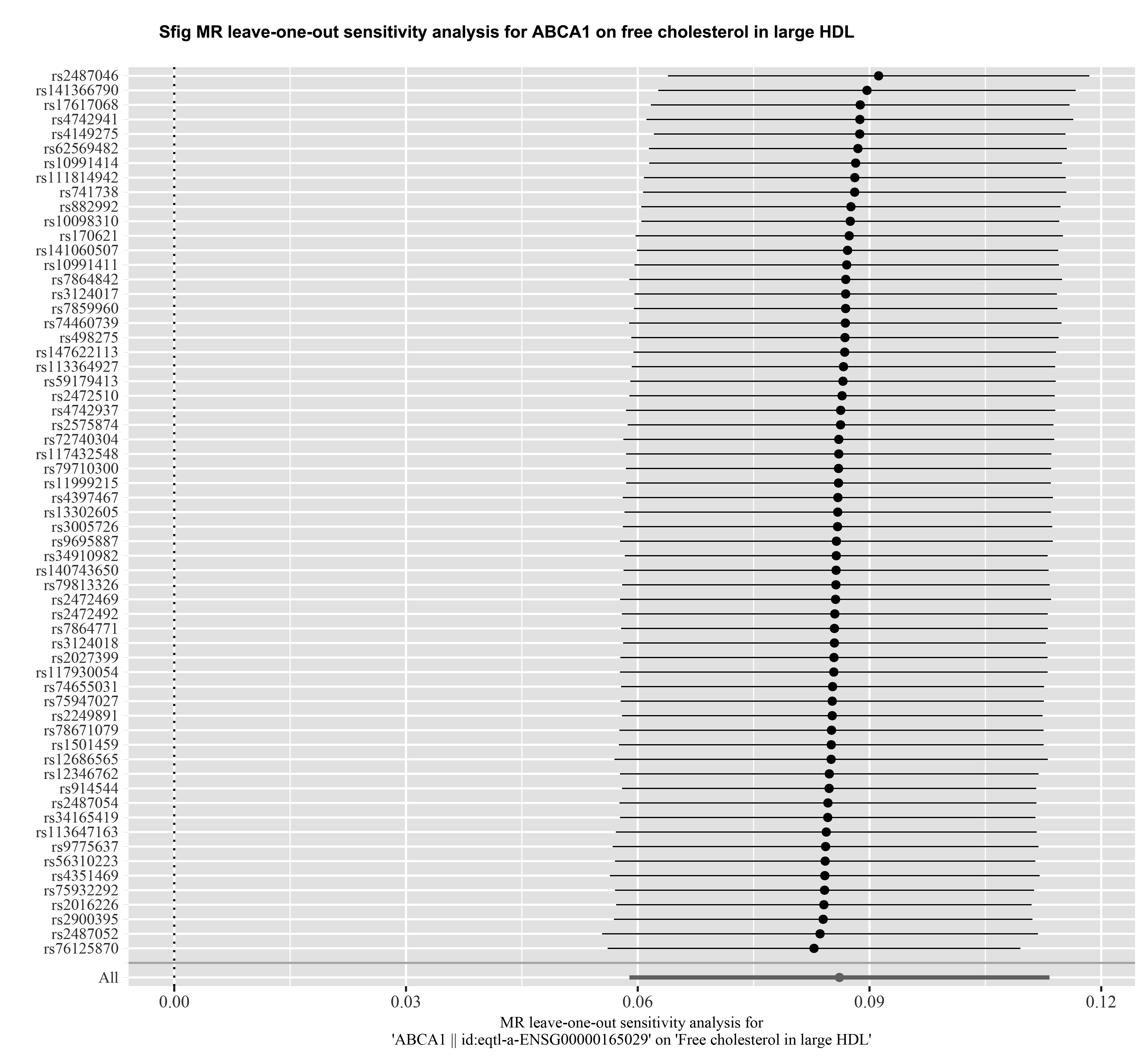

Supplement: Supplementary file 3 — Supplementary Information 3. [file 41598_2025_93644_MOESM3_ESM.zip › leave-one-out analysis/Sfig MR leave-one-out sensitivity analysis for ABCA1 on free cholesterol in large HDL.tif]

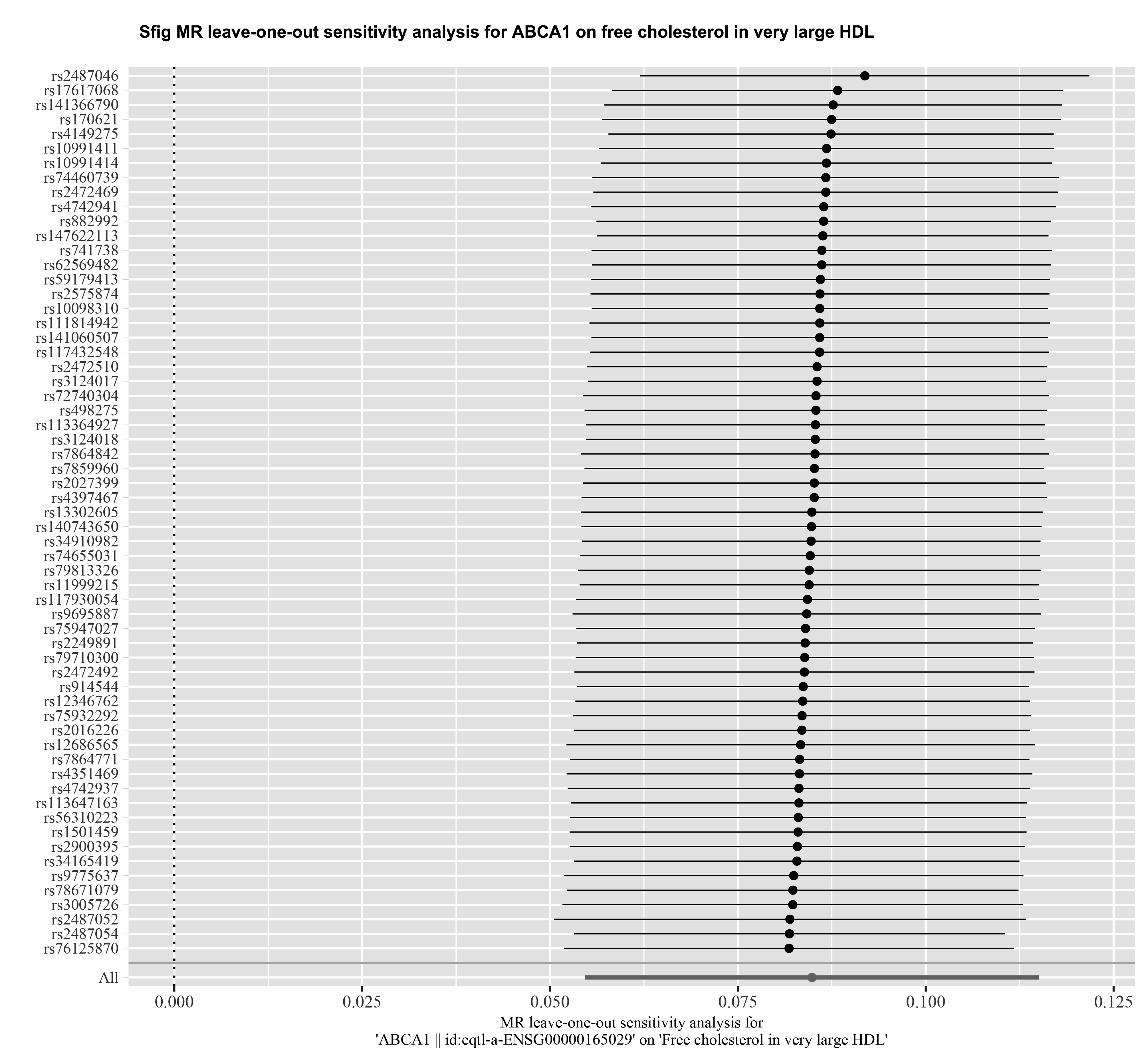

Supplement: Supplementary file 3 — Supplementary Information 3. [file 41598_2025_93644_MOESM3_ESM.zip › leave-one-out analysis/Sfig MR leave-one-out sensitivity analysis for ABCA1 on free cholesterol in very large HDL.tif]

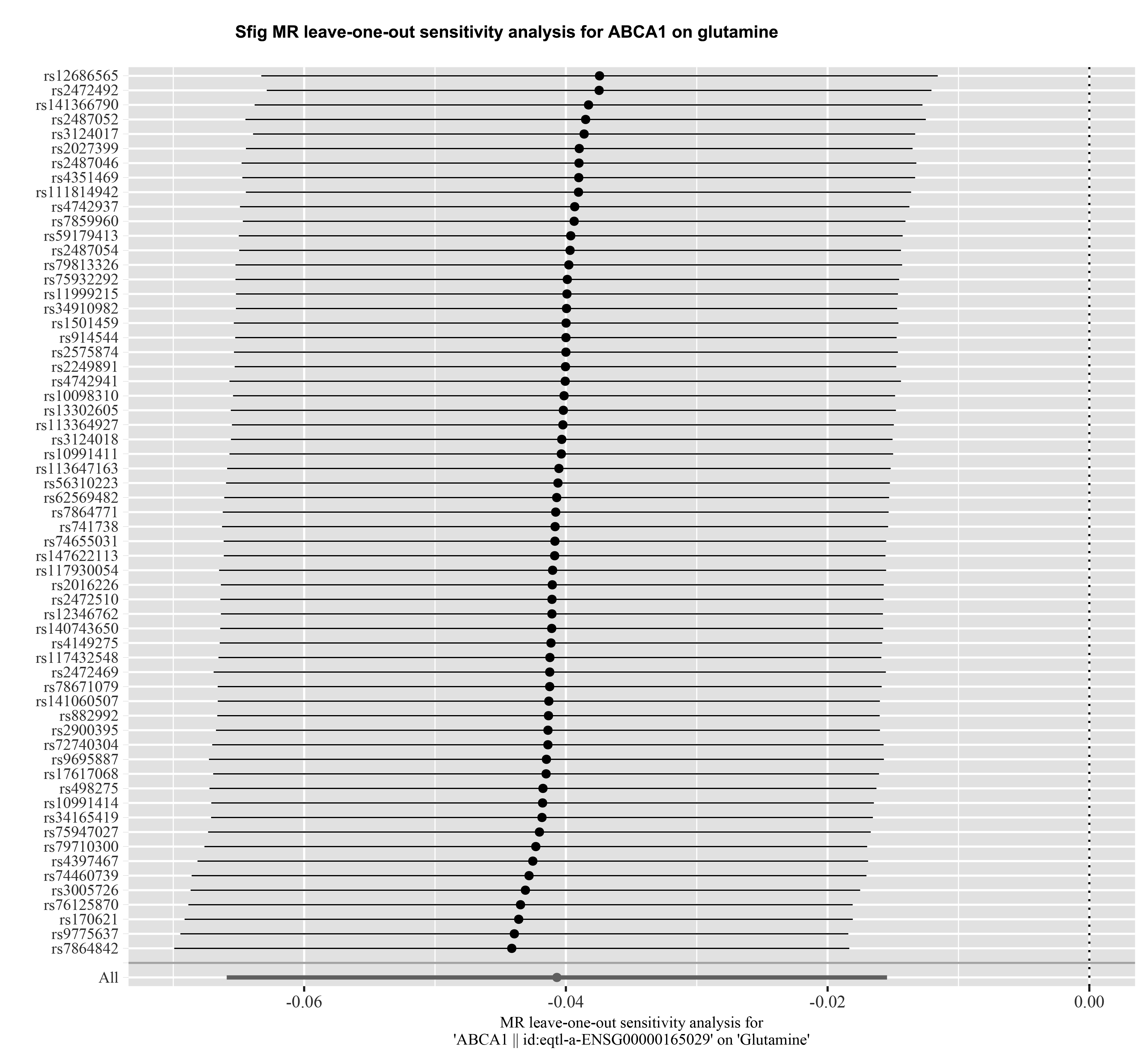

Supplement: Supplementary file 3 — Supplementary Information 3. [file 41598_2025_93644_MOESM3_ESM.zip › leave-one-out analysis/Sfig MR leave-one-out sensitivity analysis for ABCA1 on glutamine.tif]

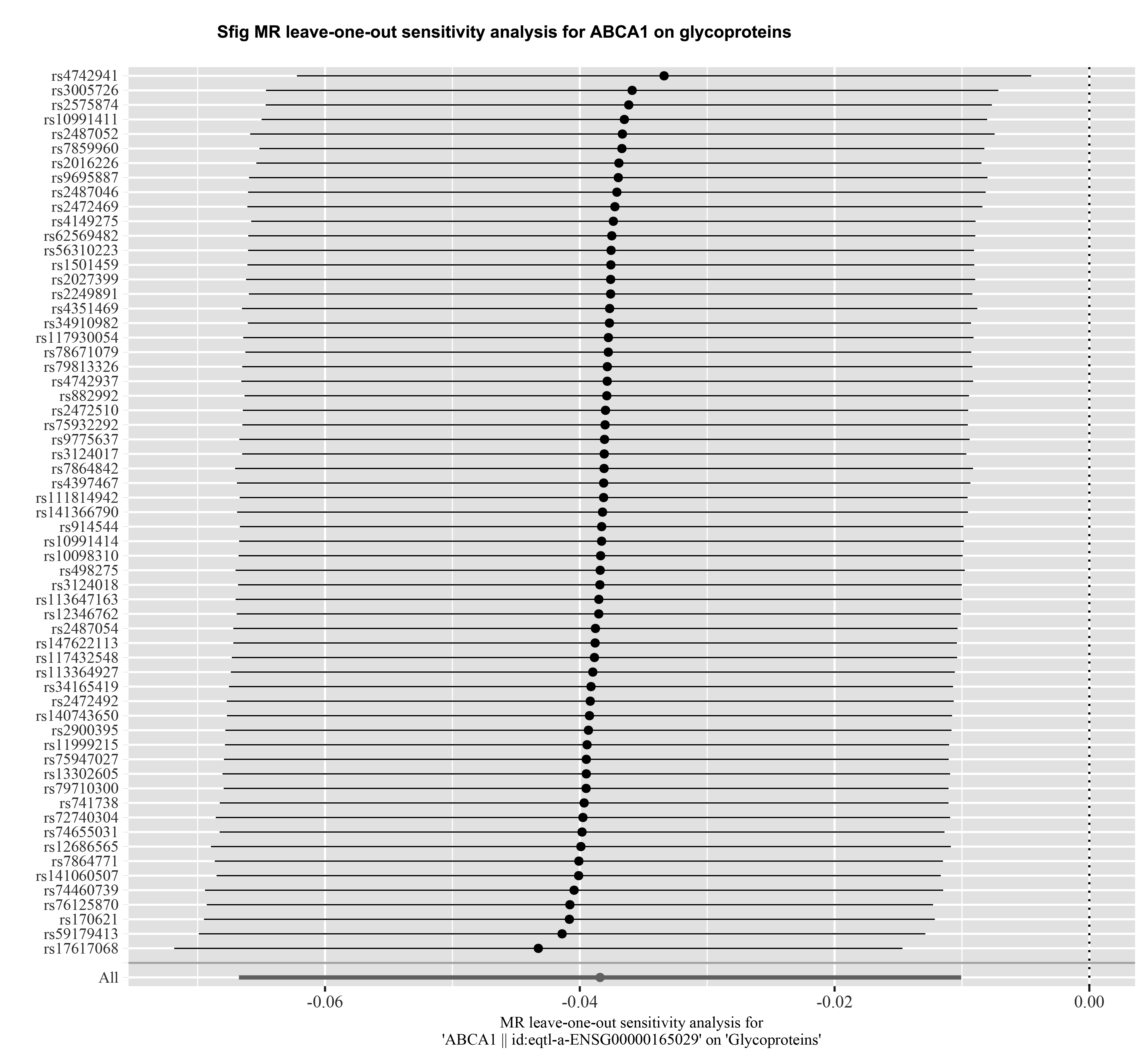

Supplement: Supplementary file 3 — Supplementary Information 3. [file 41598_2025_93644_MOESM3_ESM.zip › leave-one-out analysis/Sfig MR leave-one-out sensitivity analysis for ABCA1 on glycoproteins.tif]

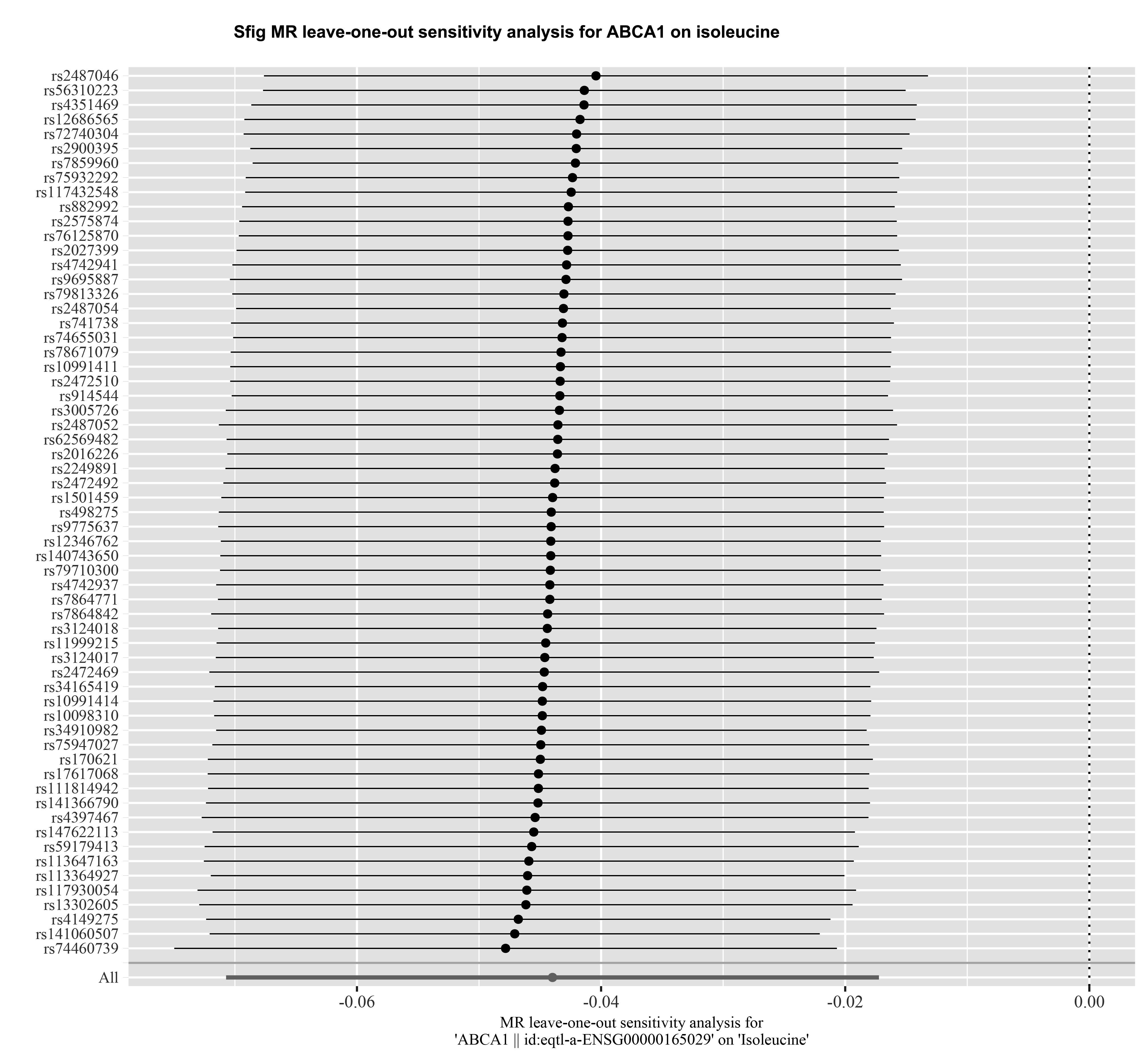

Supplement: Supplementary file 3 — Supplementary Information 3. [file 41598_2025_93644_MOESM3_ESM.zip › leave-one-out analysis/Sfig MR leave-one-out sensitivity analysis for ABCA1 on isoleucine.tif]

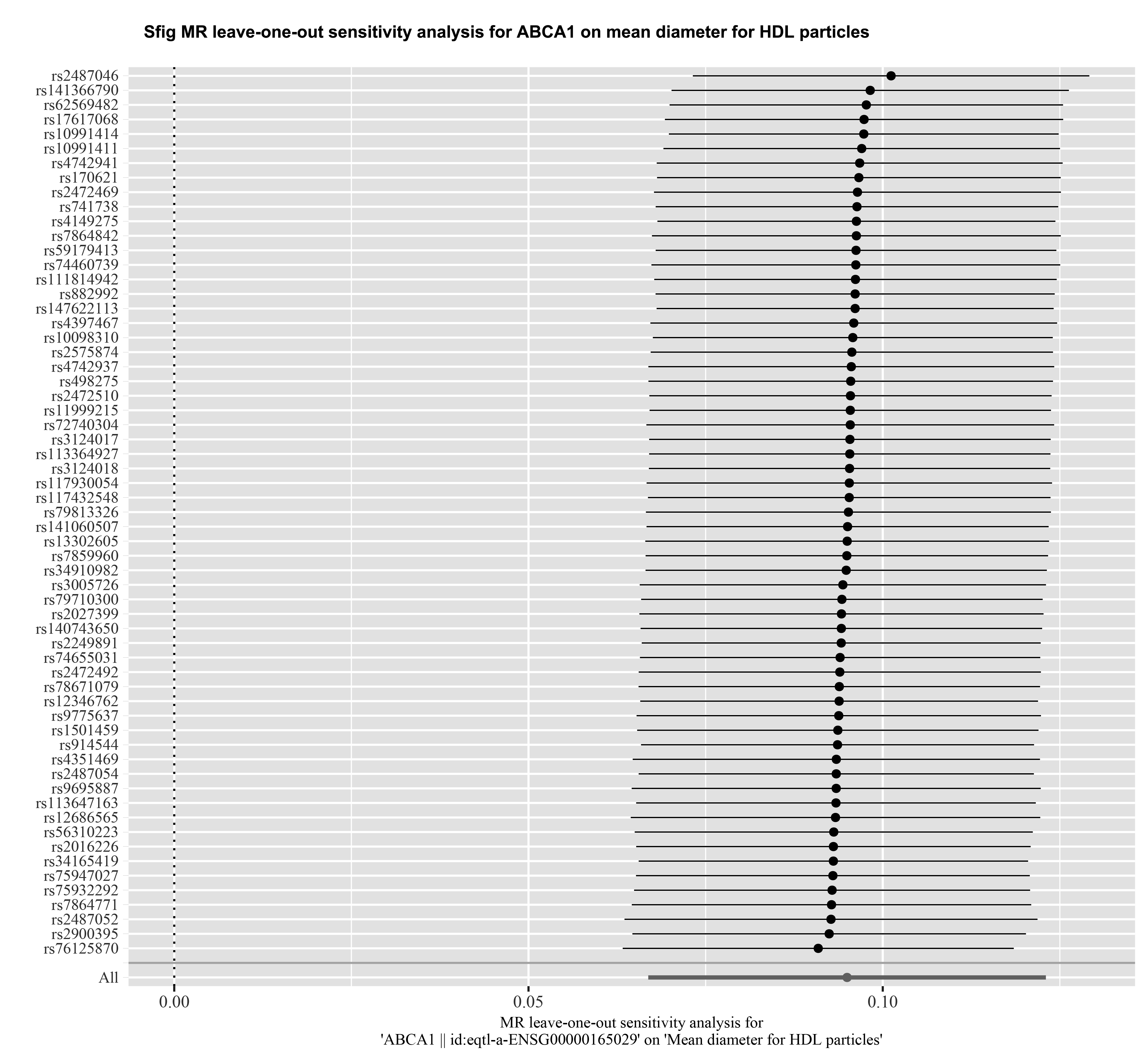

Supplement: Supplementary file 3 — Supplementary Information 3. [file 41598_2025_93644_MOESM3_ESM.zip › leave-one-out analysis/Sfig MR leave-one-out sensitivity analysis for ABCA1 on mean diameter for HDL particles.tif]

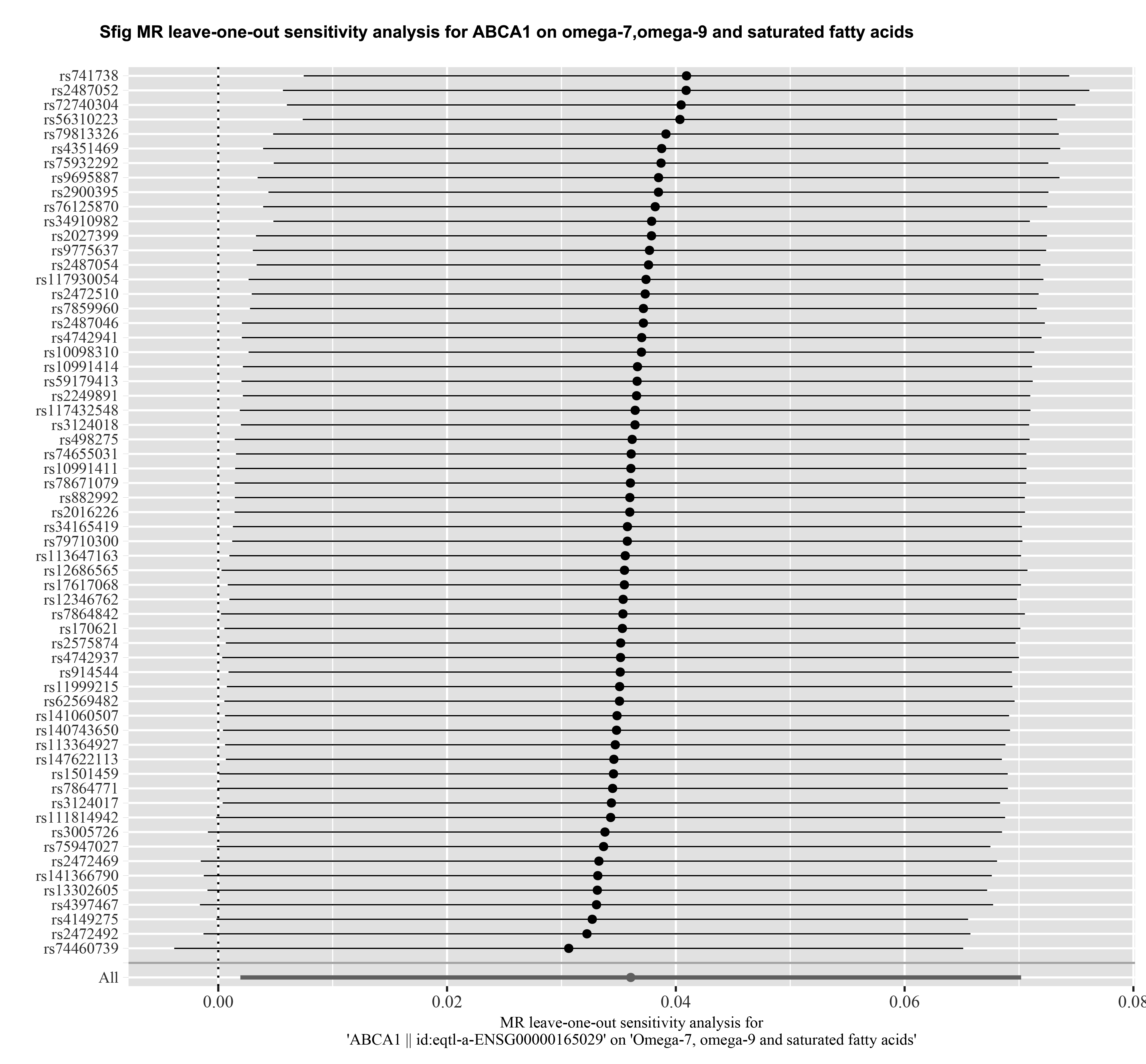

Supplement: Supplementary file 3 — Supplementary Information 3. [file 41598_2025_93644_MOESM3_ESM.zip › leave-one-out analysis/Sfig MR leave-one-out sensitivity analysis for ABCA1 on omega-7,omega-9 and saturated fatty acids.tif]

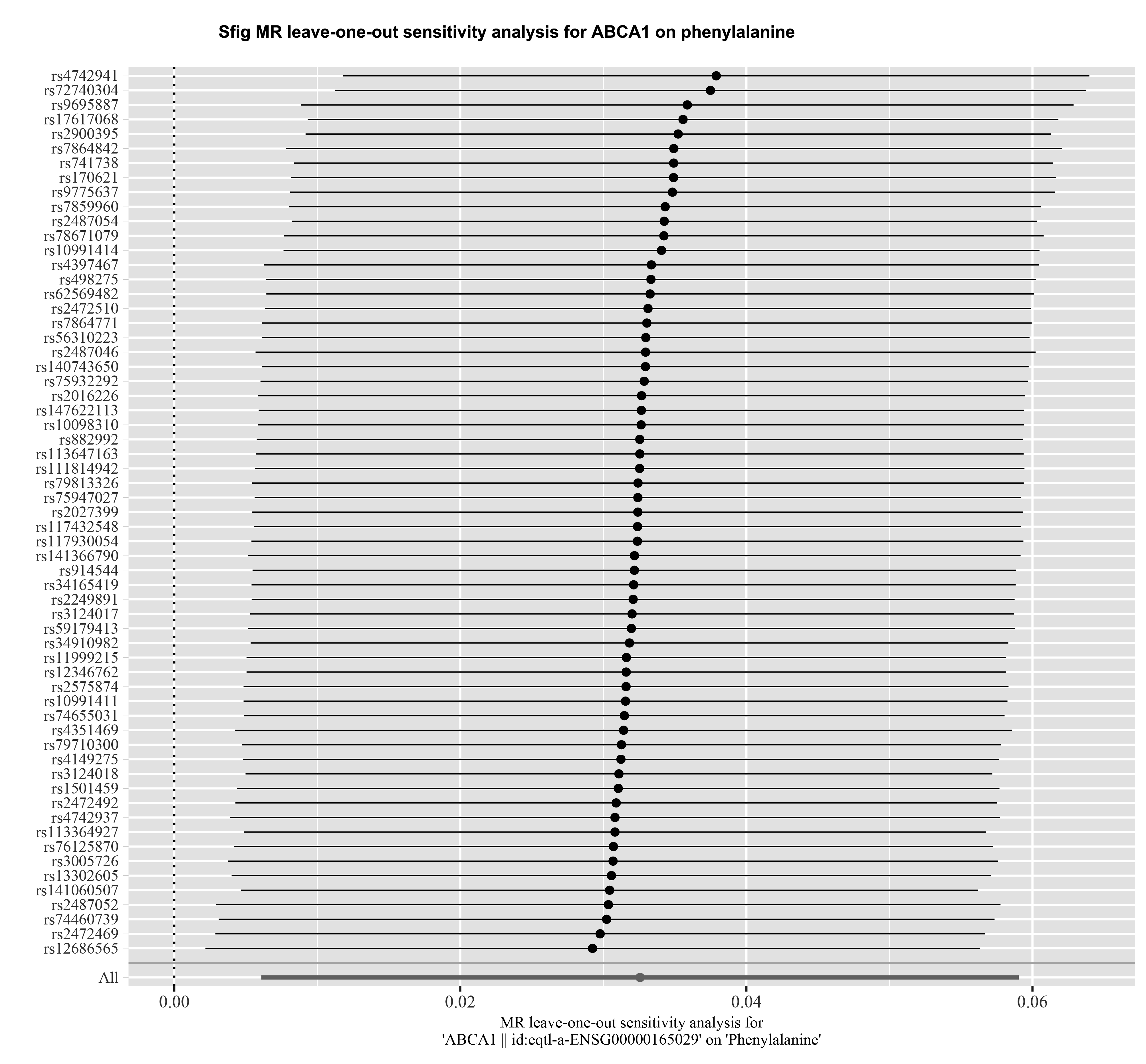

Supplement: Supplementary file 3 — Supplementary Information 3. [file 41598_2025_93644_MOESM3_ESM.zip › leave-one-out analysis/Sfig MR leave-one-out sensitivity analysis for ABCA1 on phenylalanine.tif]

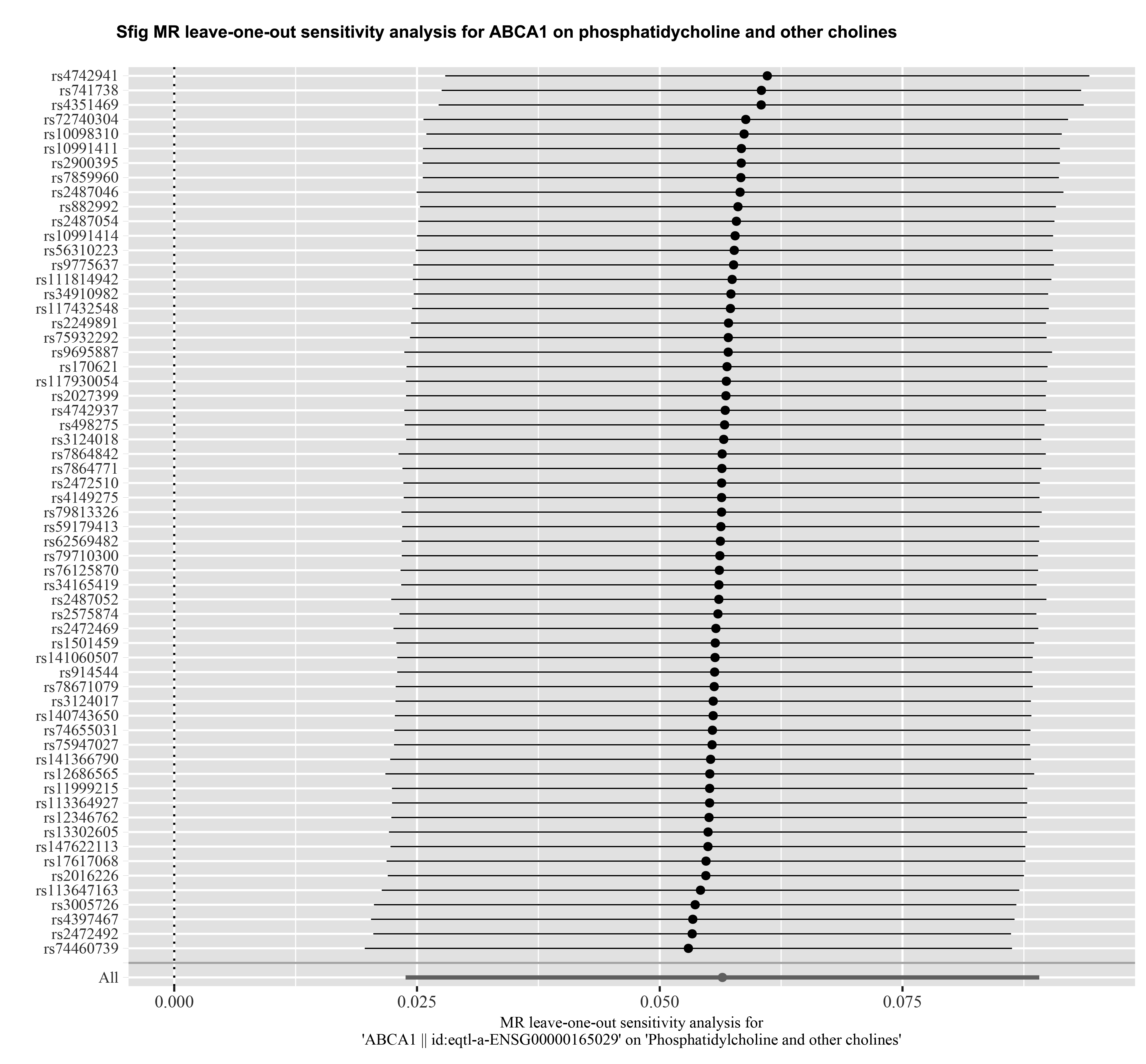

Supplement: Supplementary file 3 — Supplementary Information 3. [file 41598_2025_93644_MOESM3_ESM.zip › leave-one-out analysis/Sfig MR leave-one-out sensitivity analysis for ABCA1 on phosphatidycholine and other cholines.tif]

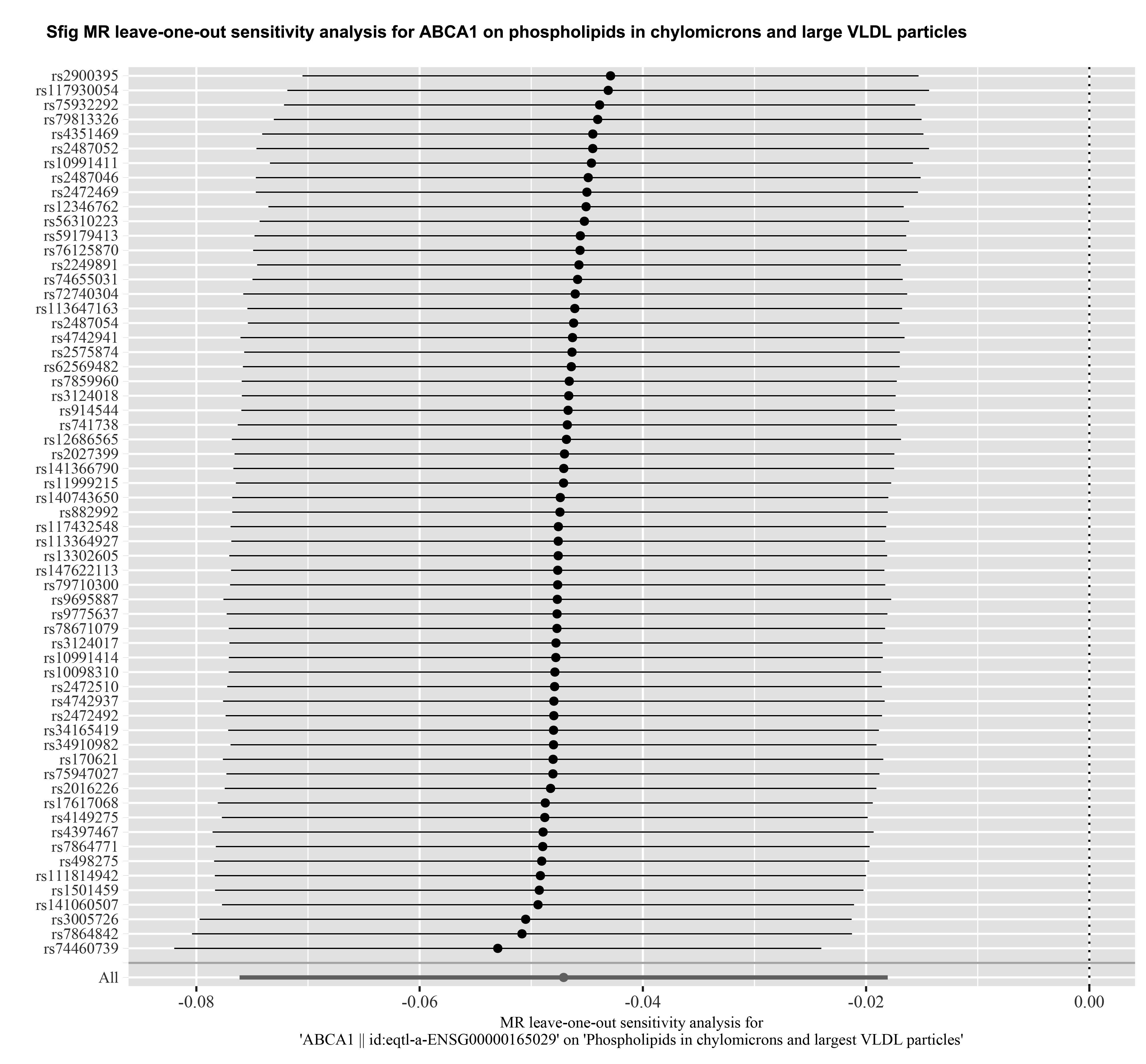

Supplement: Supplementary file 3 — Supplementary Information 3. [file 41598_2025_93644_MOESM3_ESM.zip › leave-one-out analysis/Sfig MR leave-one-out sensitivity analysis for ABCA1 on phospholipids in chylomicrons and large VLDL particles.tif]

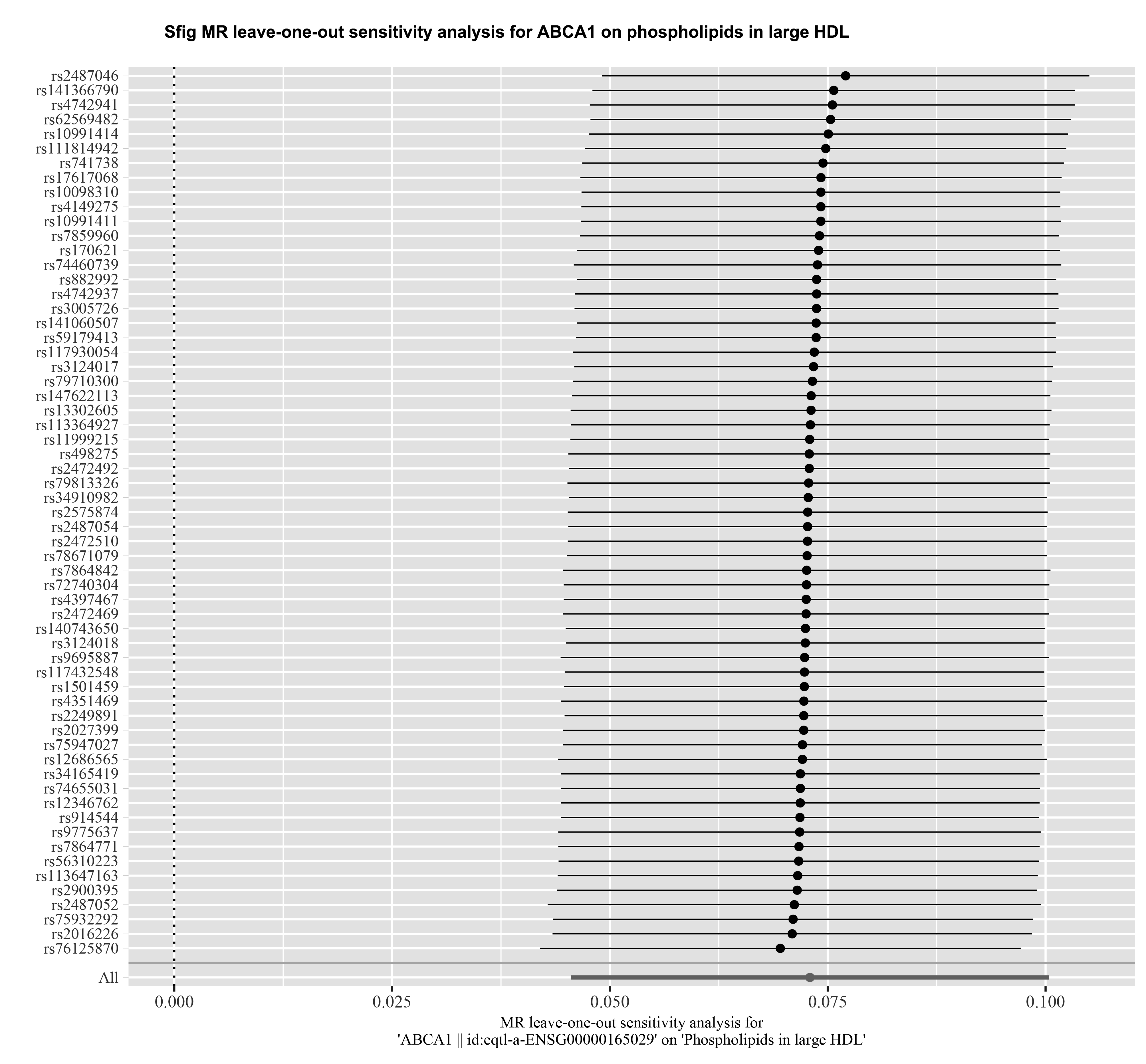

Supplement: Supplementary file 3 — Supplementary Information 3. [file 41598_2025_93644_MOESM3_ESM.zip › leave-one-out analysis/Sfig MR leave-one-out sensitivity analysis for ABCA1 on phospholipids in large HDL.tif]

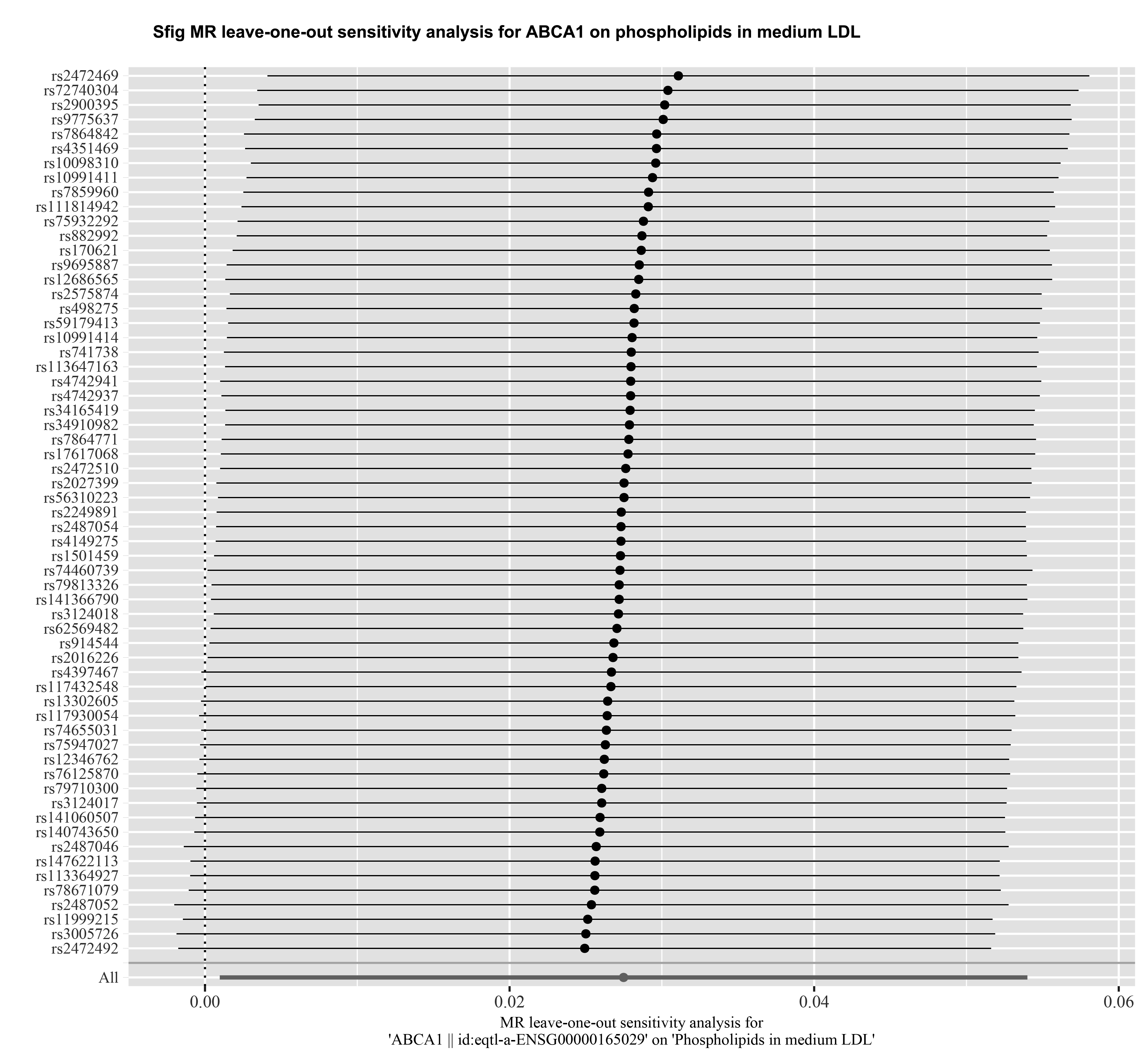

Supplement: Supplementary file 3 — Supplementary Information 3. [file 41598_2025_93644_MOESM3_ESM.zip › leave-one-out analysis/Sfig MR leave-one-out sensitivity analysis for ABCA1 on phospholipids in medium LDL.tif]

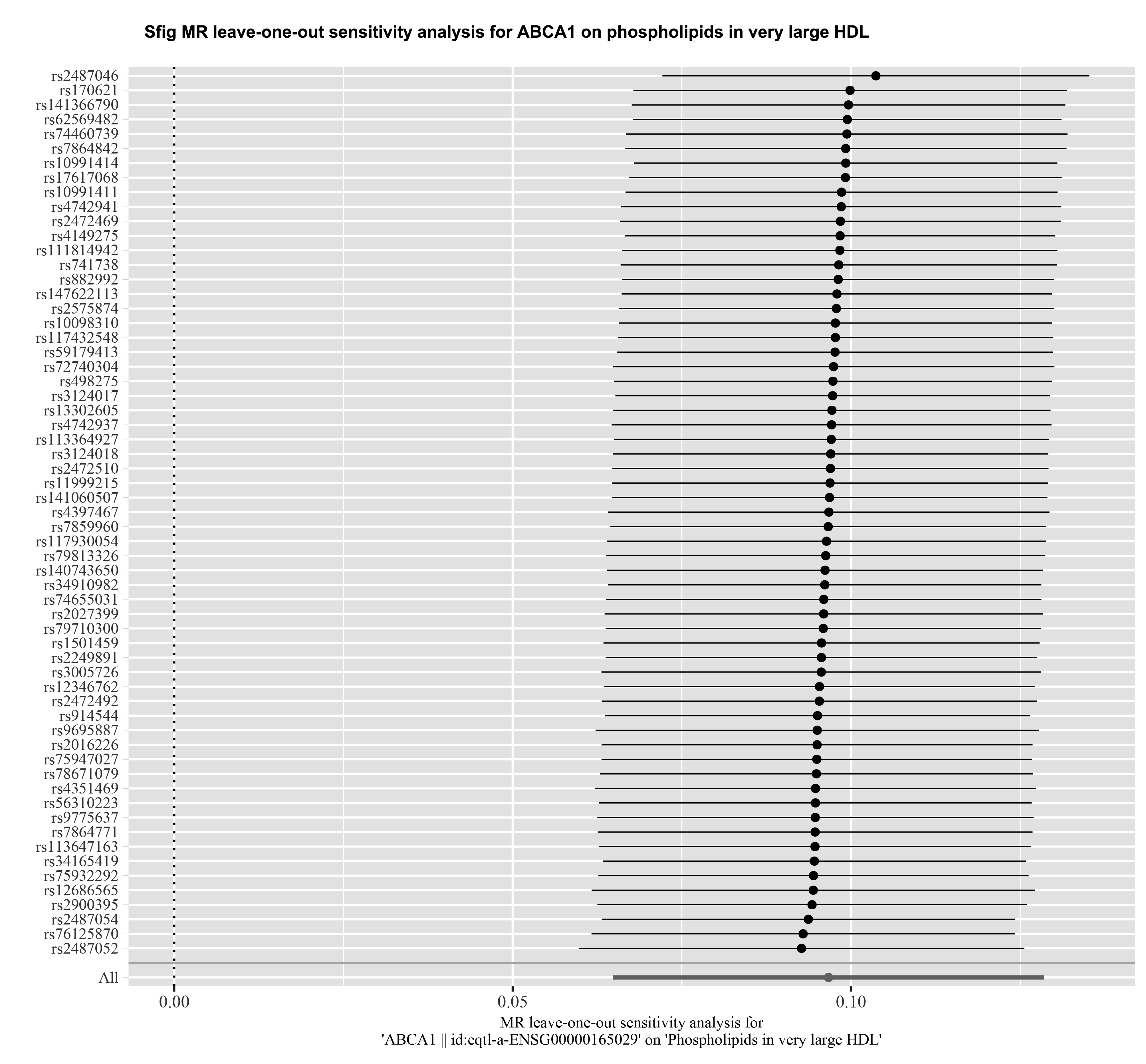

Supplement: Supplementary file 3 — Supplementary Information 3. [file 41598_2025_93644_MOESM3_ESM.zip › leave-one-out analysis/Sfig MR leave-one-out sensitivity analysis for ABCA1 on phospholipids in very large HDL.tif]

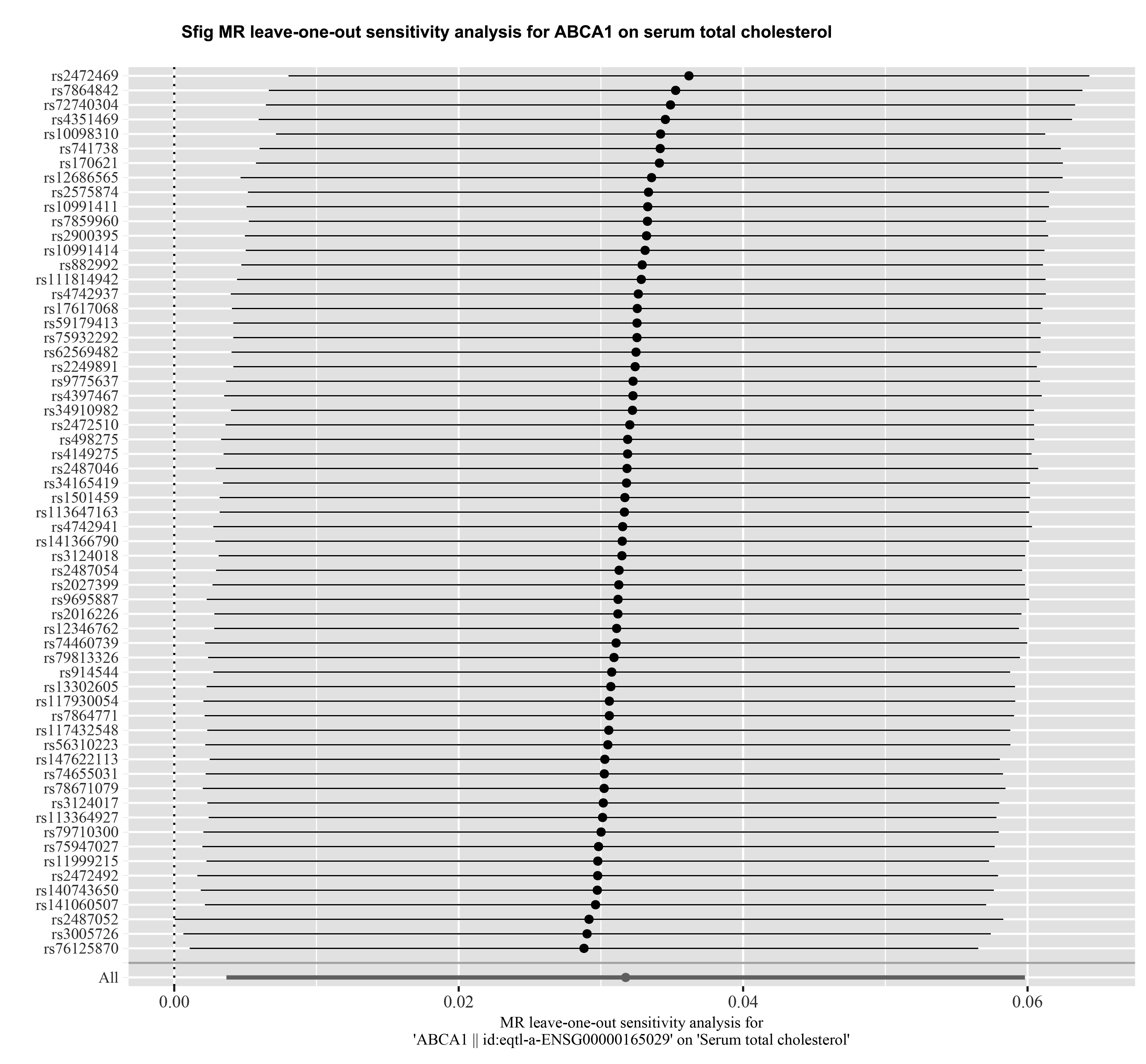

Supplement: Supplementary file 3 — Supplementary Information 3. [file 41598_2025_93644_MOESM3_ESM.zip › leave-one-out analysis/Sfig MR leave-one-out sensitivity analysis for ABCA1 on serum total cholesterol.tif]

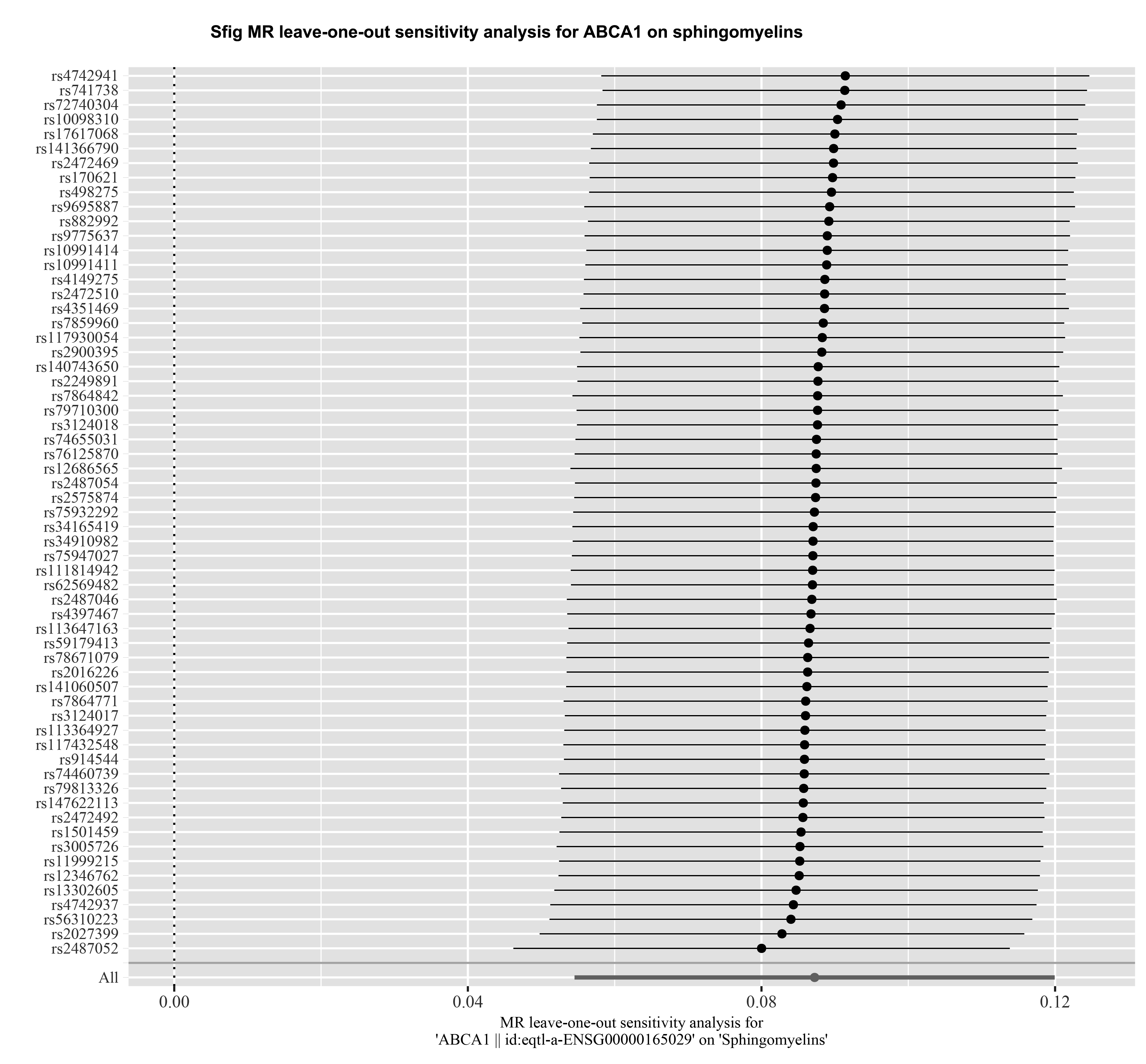

Supplement: Supplementary file 3 — Supplementary Information 3. [file 41598_2025_93644_MOESM3_ESM.zip › leave-one-out analysis/Sfig MR leave-one-out sensitivity analysis for ABCA1 on sphingomyelins.tif]

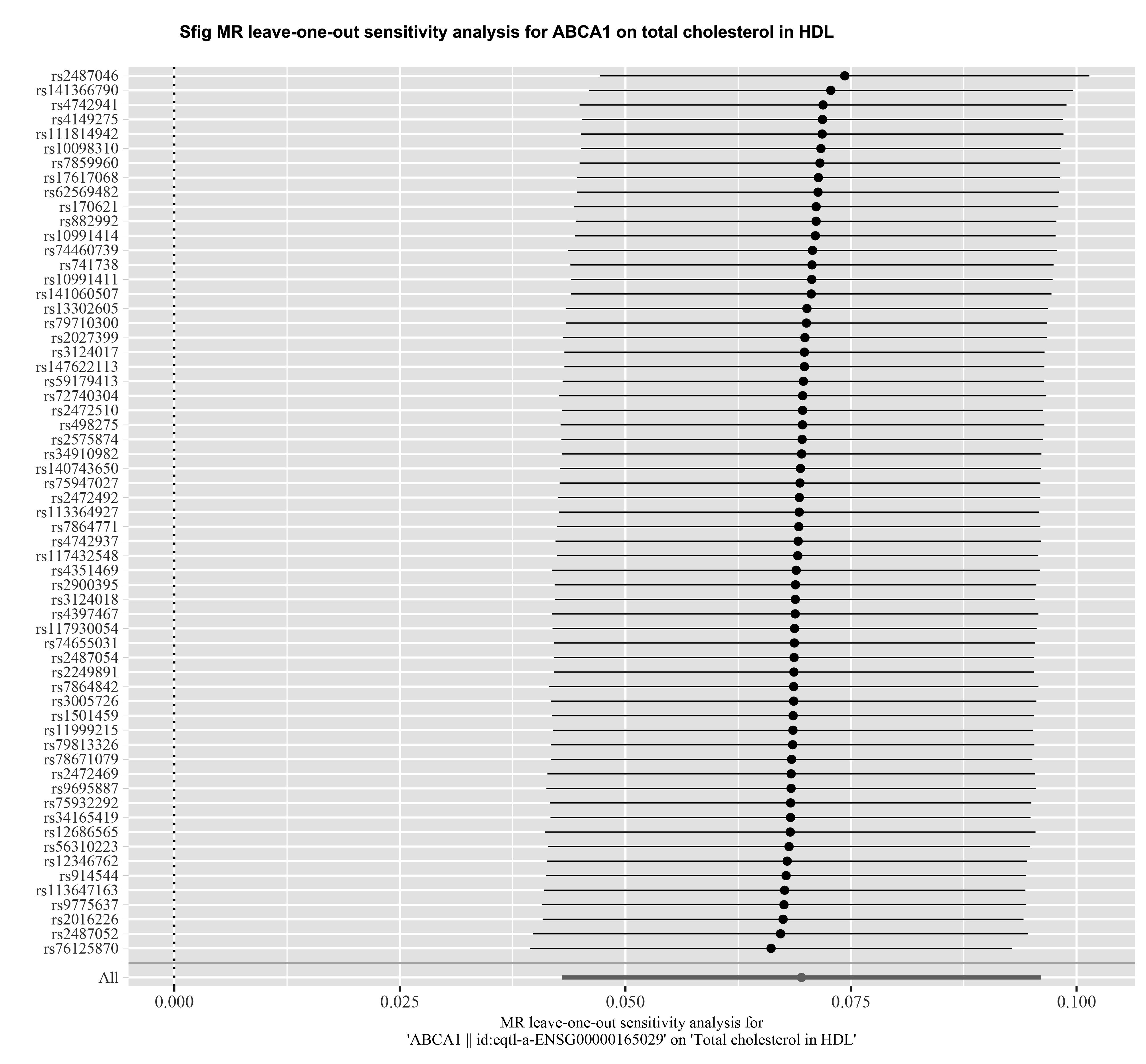

Supplement: Supplementary file 3 — Supplementary Information 3. [file 41598_2025_93644_MOESM3_ESM.zip › leave-one-out analysis/Sfig MR leave-one-out sensitivity analysis for ABCA1 on total cholesterol in HDL.tif]

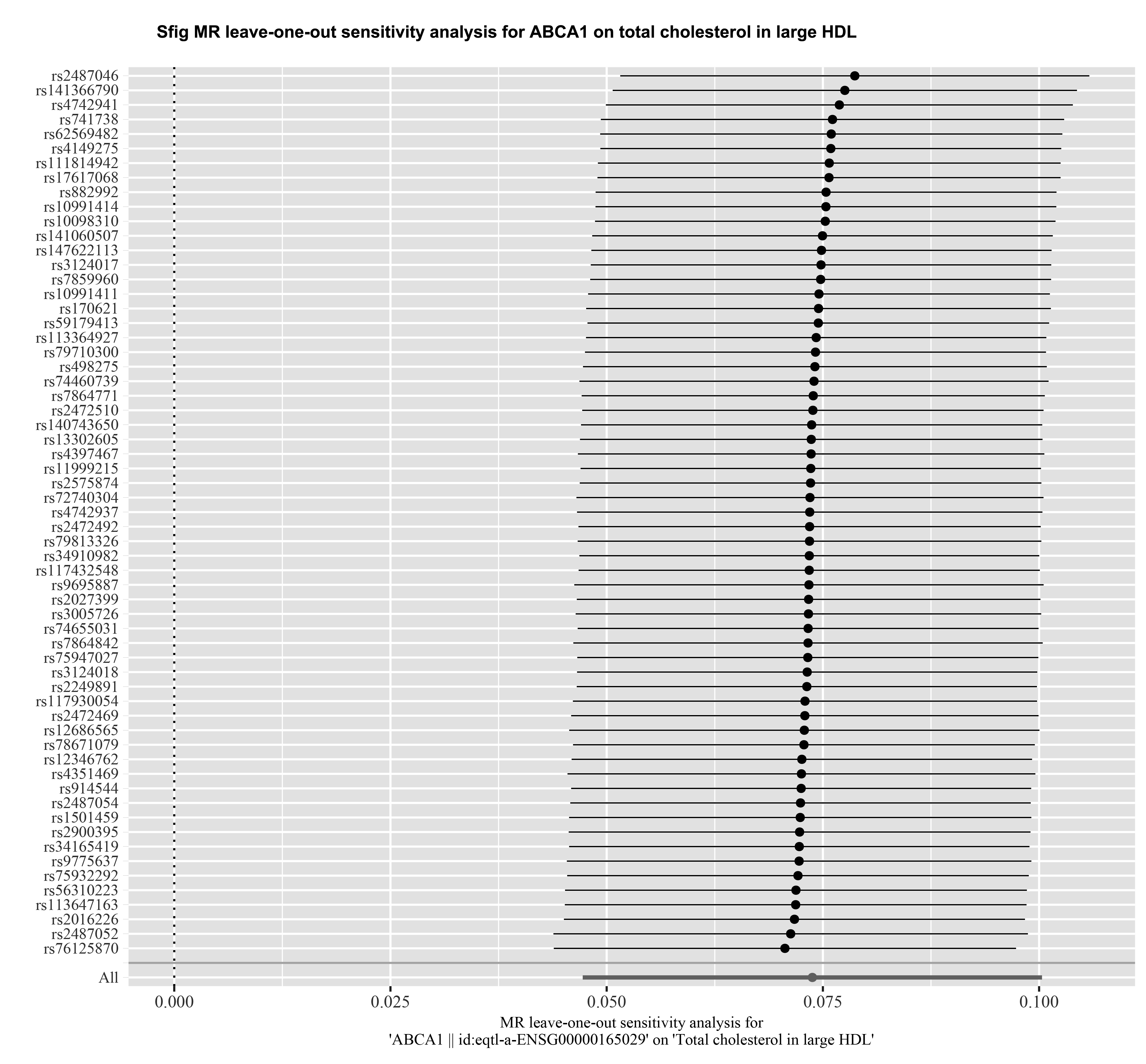

Supplement: Supplementary file 3 — Supplementary Information 3. [file 41598_2025_93644_MOESM3_ESM.zip › leave-one-out analysis/Sfig MR leave-one-out sensitivity analysis for ABCA1 on total cholesterol in large HDL.tif]

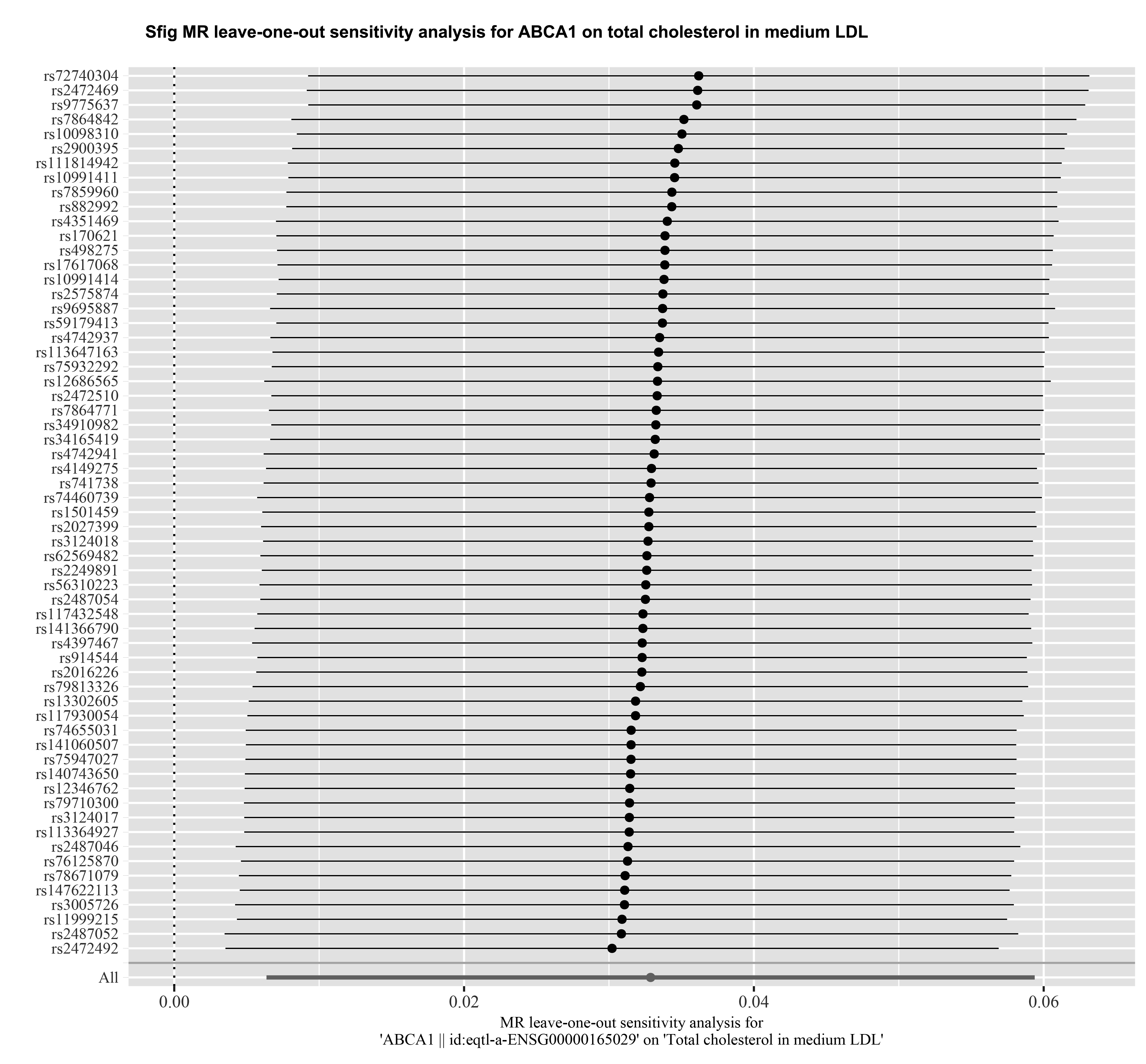

Supplement: Supplementary file 3 — Supplementary Information 3. [file 41598_2025_93644_MOESM3_ESM.zip › leave-one-out analysis/Sfig MR leave-one-out sensitivity analysis for ABCA1 on total cholesterol in medium LDL.tif]

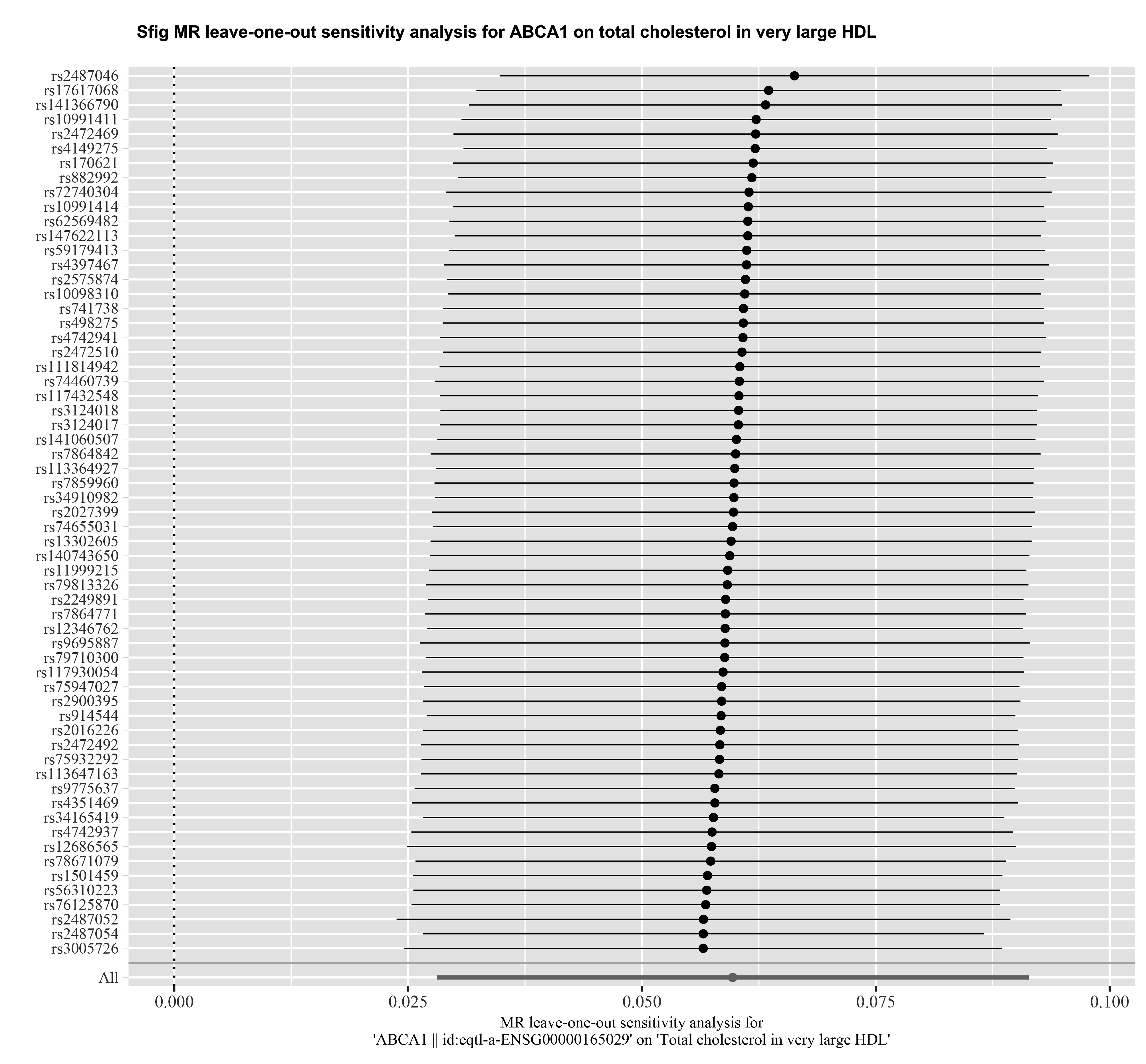

Supplement: Supplementary file 3 — Supplementary Information 3. [file 41598_2025_93644_MOESM3_ESM.zip › leave-one-out analysis/Sfig MR leave-one-out sensitivity analysis for ABCA1 on total cholesterol in very large HDL.tif]

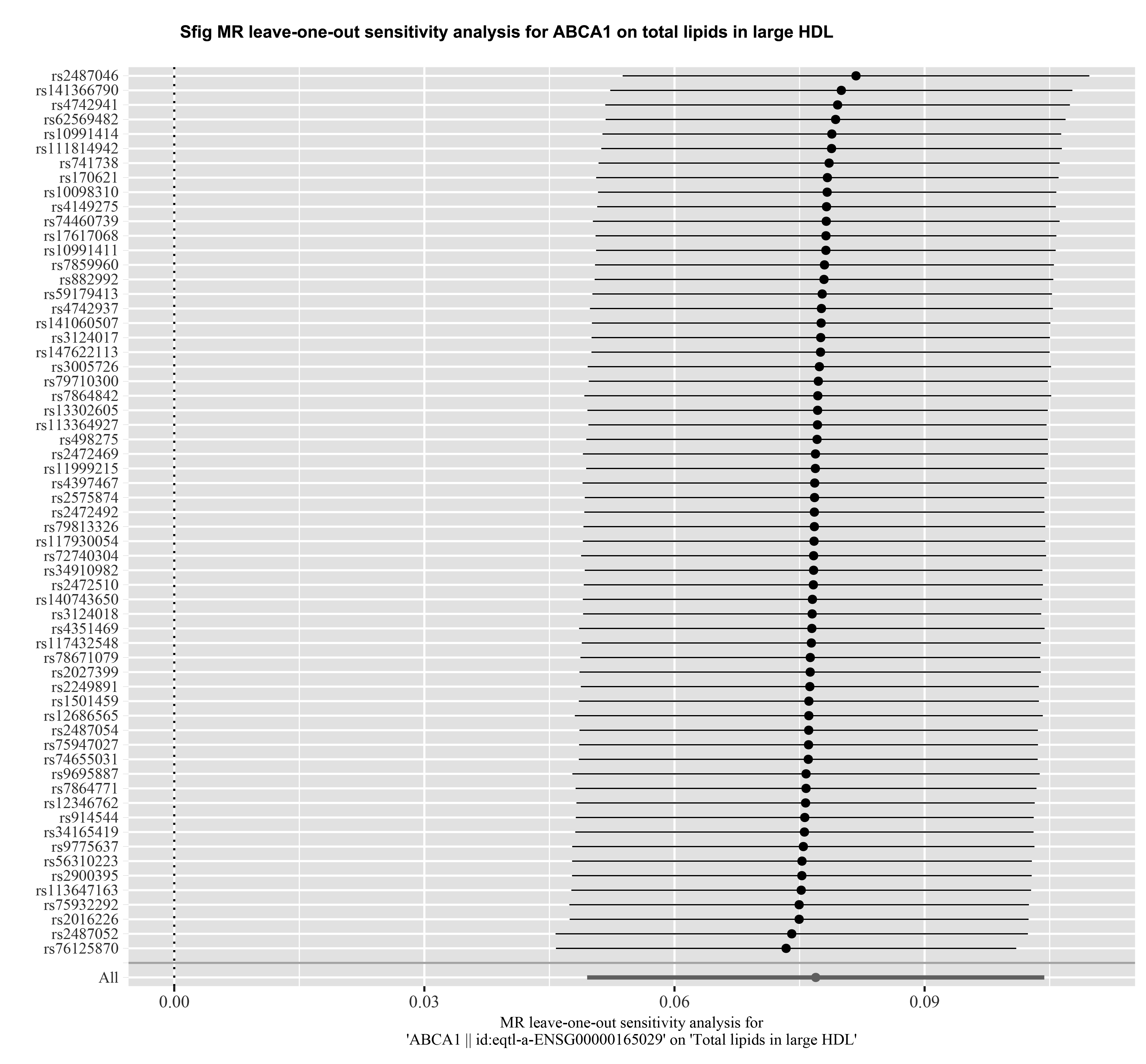

Supplement: Supplementary file 3 — Supplementary Information 3. [file 41598_2025_93644_MOESM3_ESM.zip › leave-one-out analysis/Sfig MR leave-one-out sensitivity analysis for ABCA1 on total lipids in large HDL.tif]

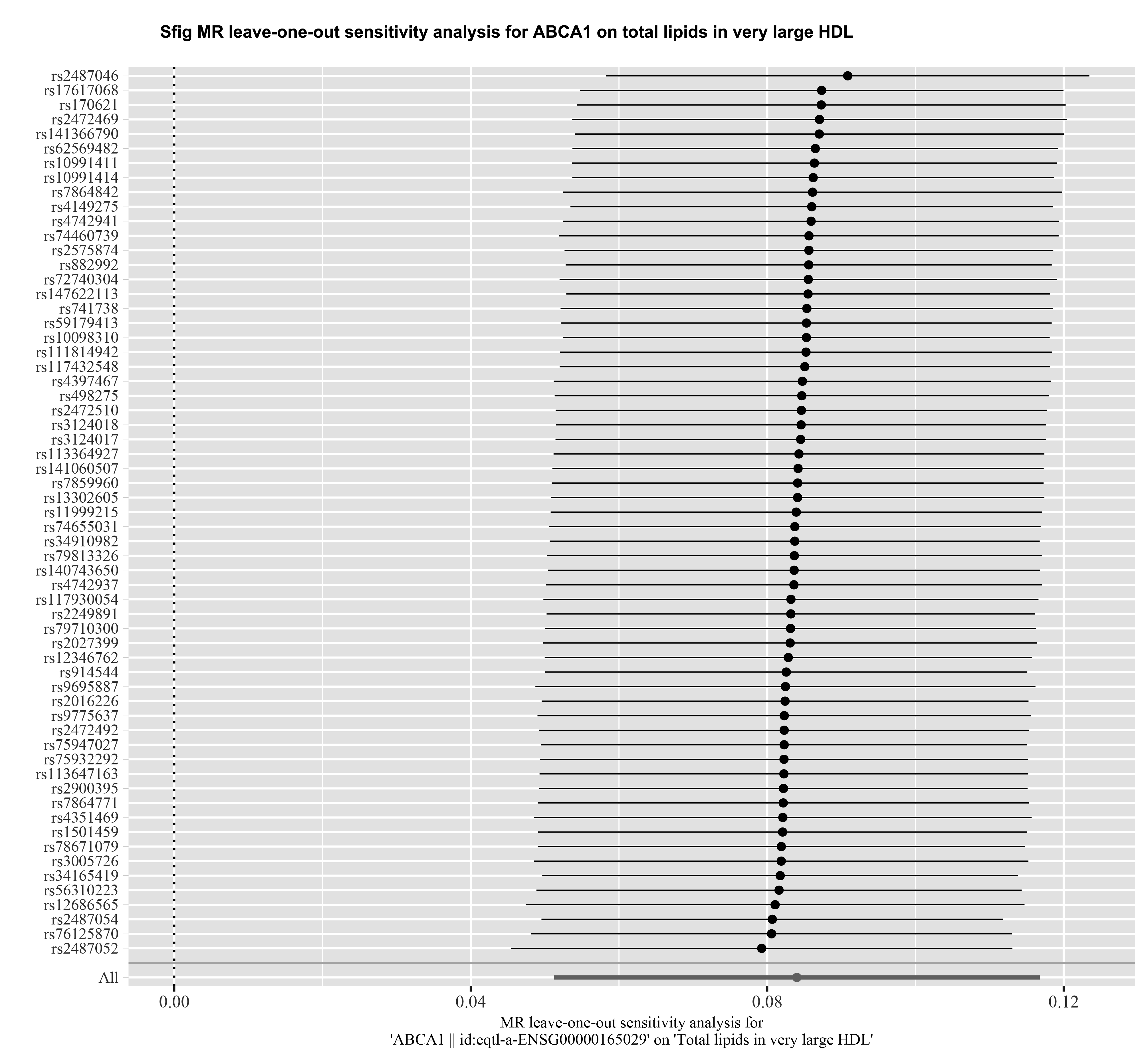

Supplement: Supplementary file 3 — Supplementary Information 3. [file 41598_2025_93644_MOESM3_ESM.zip › leave-one-out analysis/Sfig MR leave-one-out sensitivity analysis for ABCA1 on total lipids in very large HDL.tif]

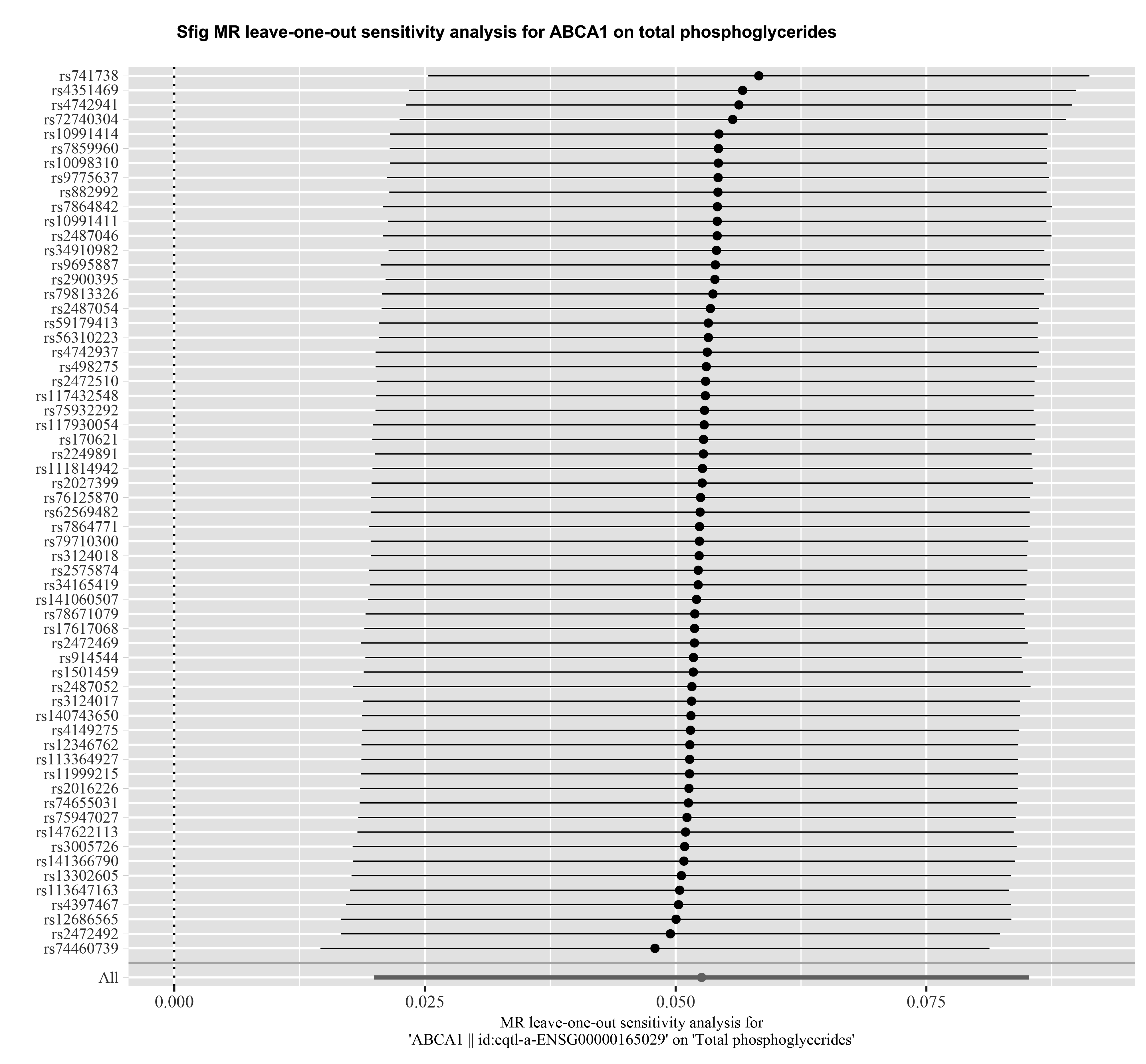

Supplement: Supplementary file 3 — Supplementary Information 3. [file 41598_2025_93644_MOESM3_ESM.zip › leave-one-out analysis/Sfig MR leave-one-out sensitivity analysis for ABCA1 on total phosphoglycerides.tif]

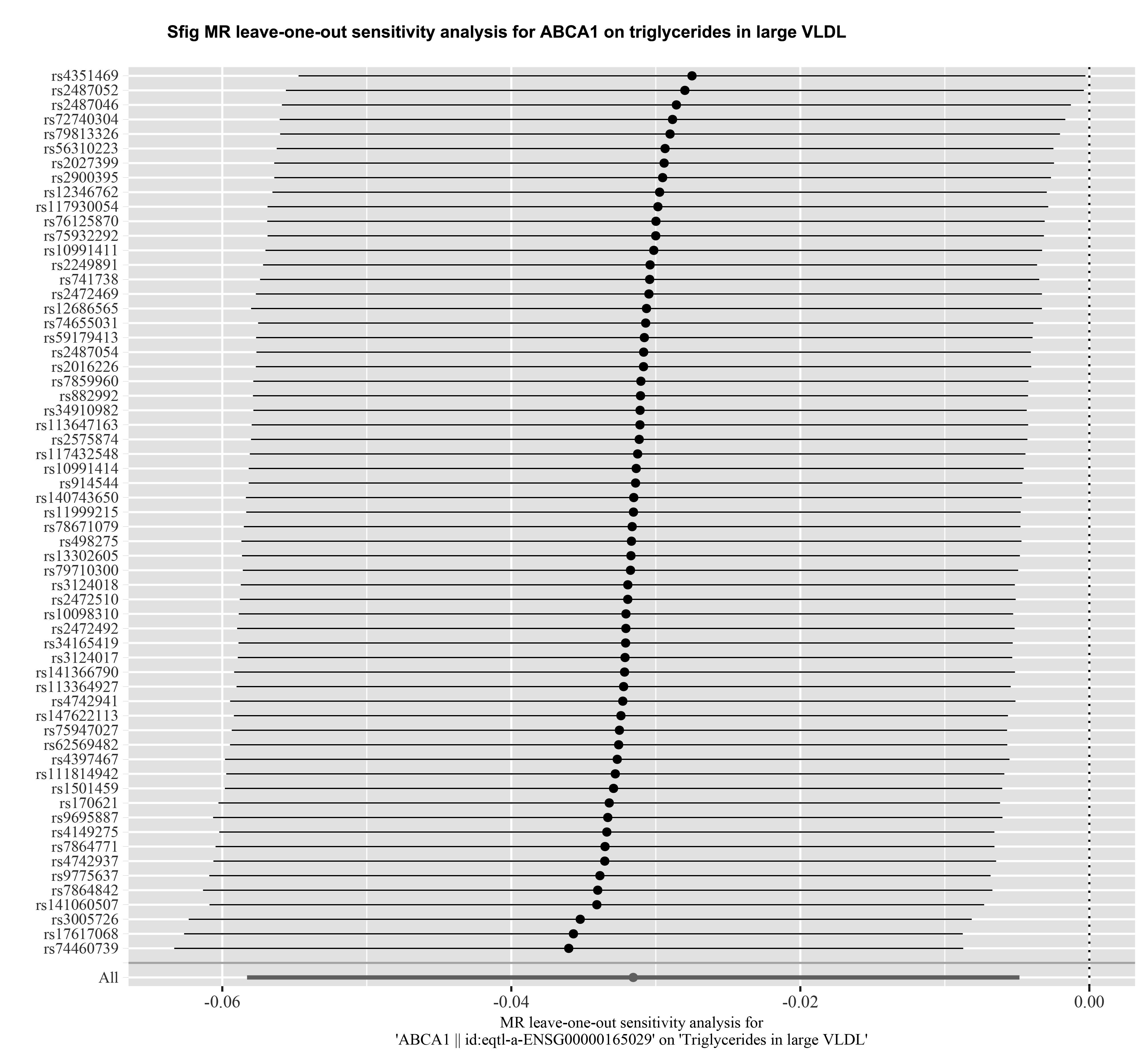

Supplement: Supplementary file 3 — Supplementary Information 3. [file 41598_2025_93644_MOESM3_ESM.zip › leave-one-out analysis/Sfig MR leave-one-out sensitivity analysis for ABCA1 on triglycerides in large VLDL.tif]

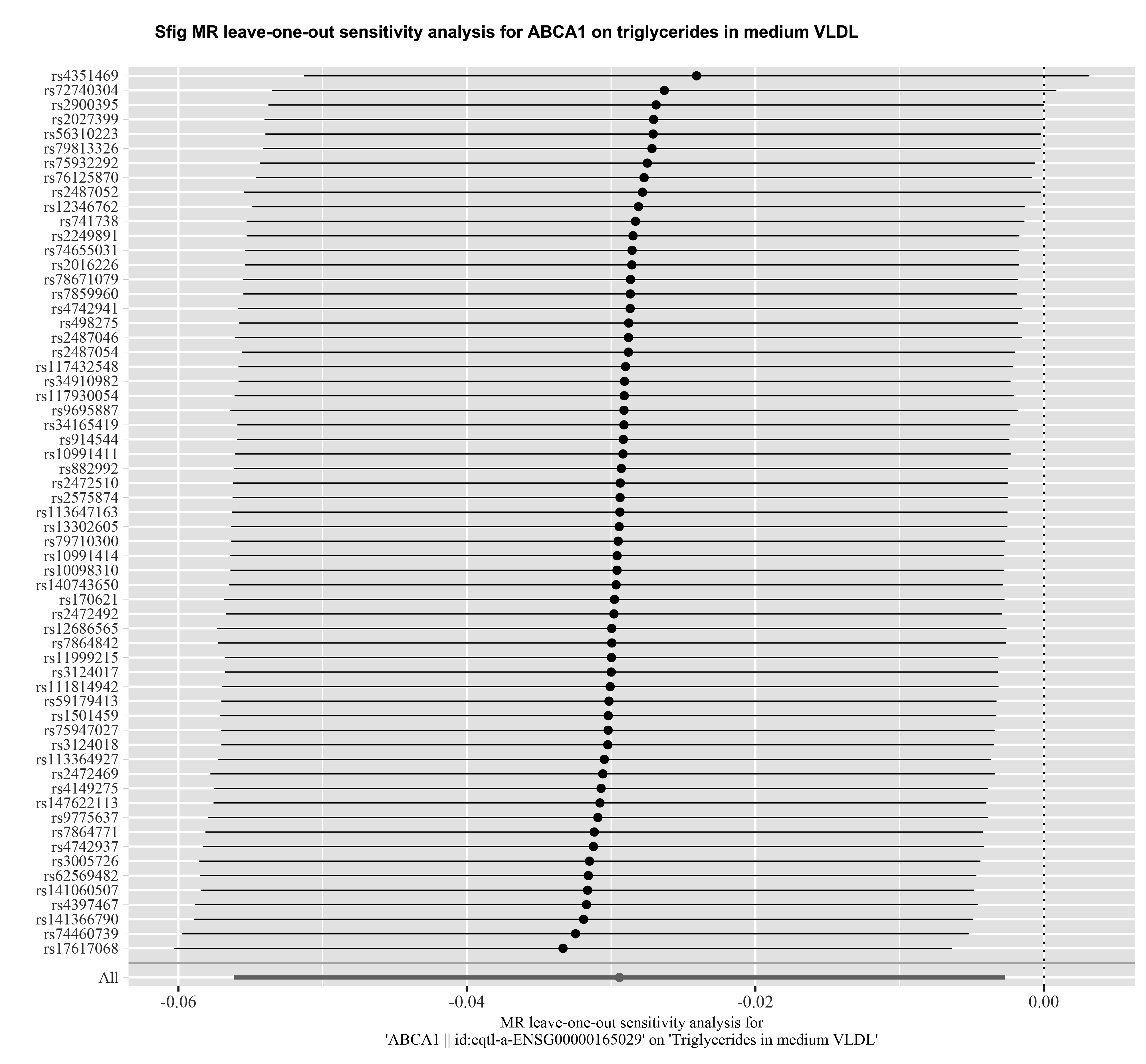

Supplement: Supplementary file 3 — Supplementary Information 3. [file 41598_2025_93644_MOESM3_ESM.zip › leave-one-out analysis/Sfig MR leave-one-out sensitivity analysis for ABCA1 on triglycerides in medium VLDL.tif]

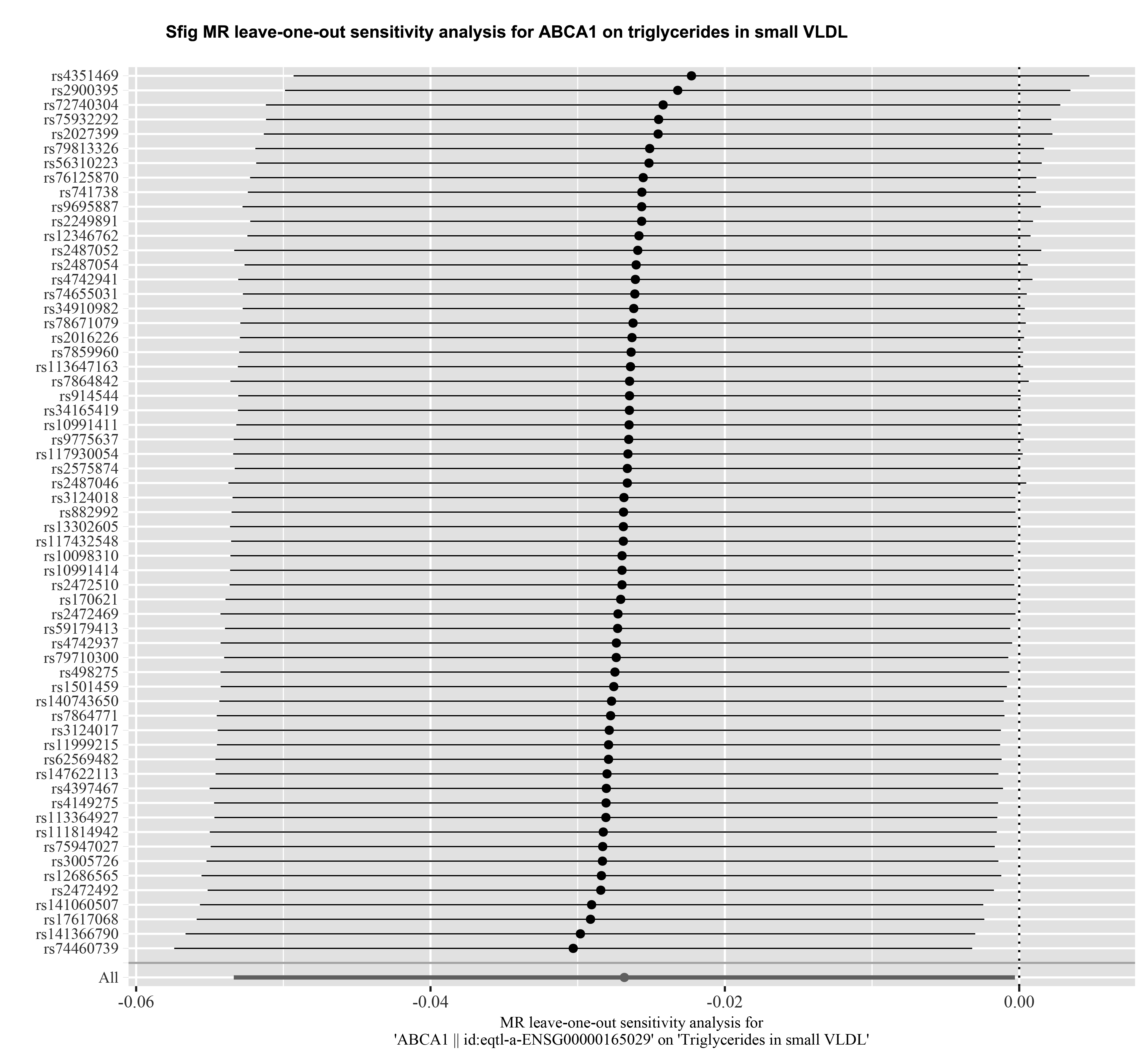

Supplement: Supplementary file 3 — Supplementary Information 3. [file 41598_2025_93644_MOESM3_ESM.zip › leave-one-out analysis/Sfig MR leave-one-out sensitivity analysis for ABCA1 on triglycerides in small VLDL.tif]

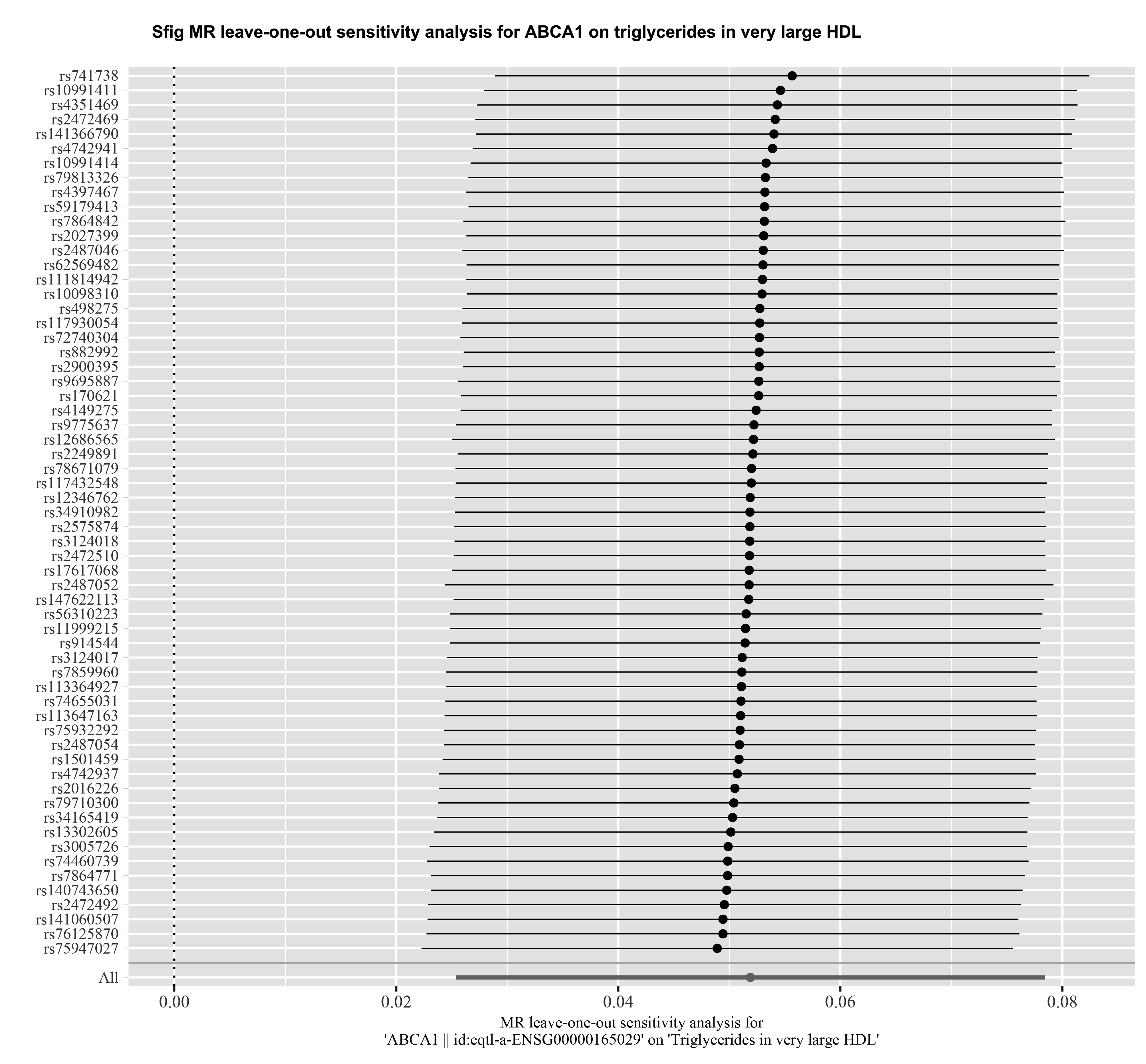

Supplement: Supplementary file 3 — Supplementary Information 3. [file 41598_2025_93644_MOESM3_ESM.zip › leave-one-out analysis/Sfig MR leave-one-out sensitivity analysis for ABCA1 on triglycerides in very large HDL.tif]

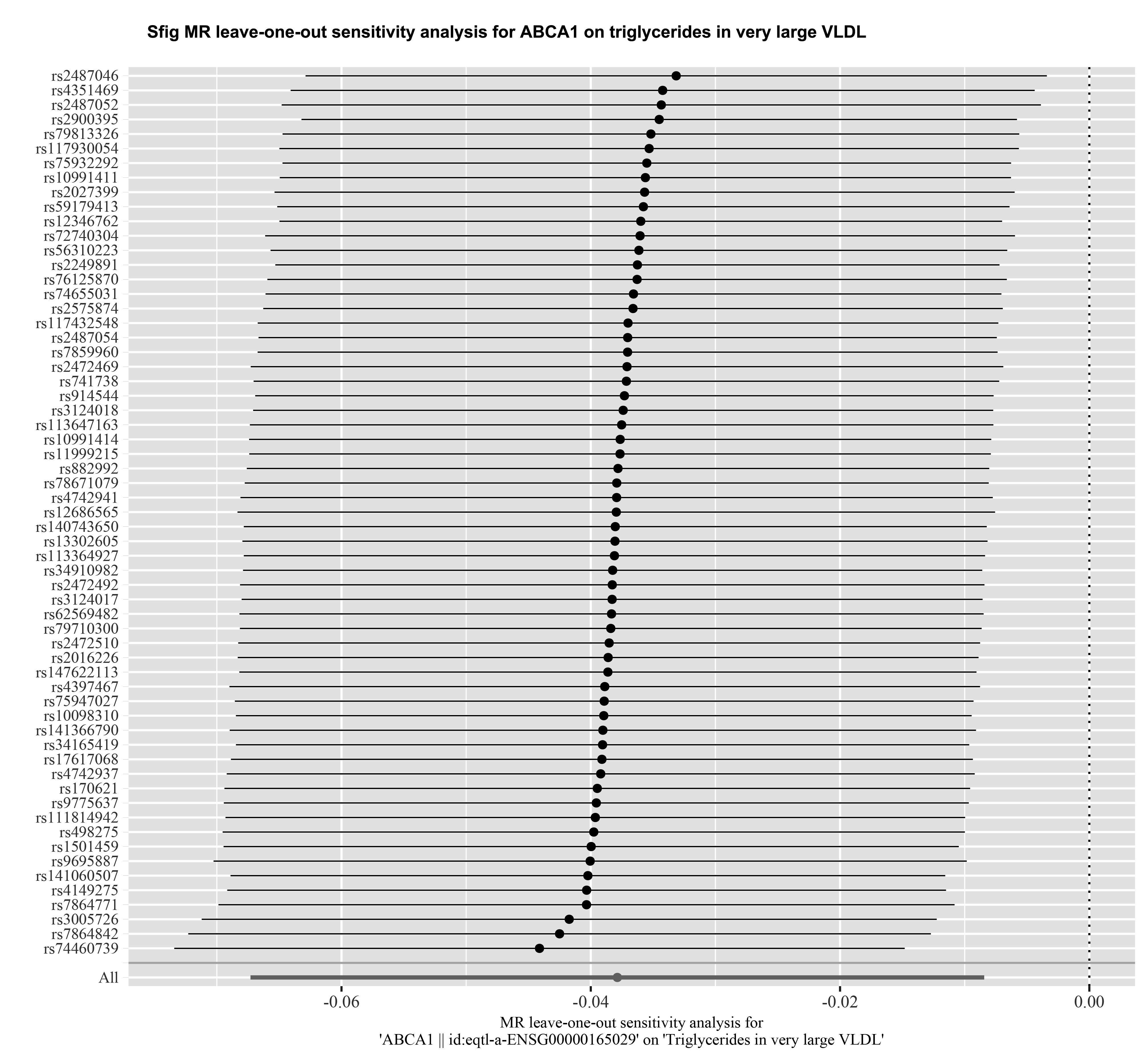

Supplement: Supplementary file 3 — Supplementary Information 3. [file 41598_2025_93644_MOESM3_ESM.zip › leave-one-out analysis/Sfig MR leave-one-out sensitivity analysis for ABCA1 on triglycerides in very large VLDL.tif]

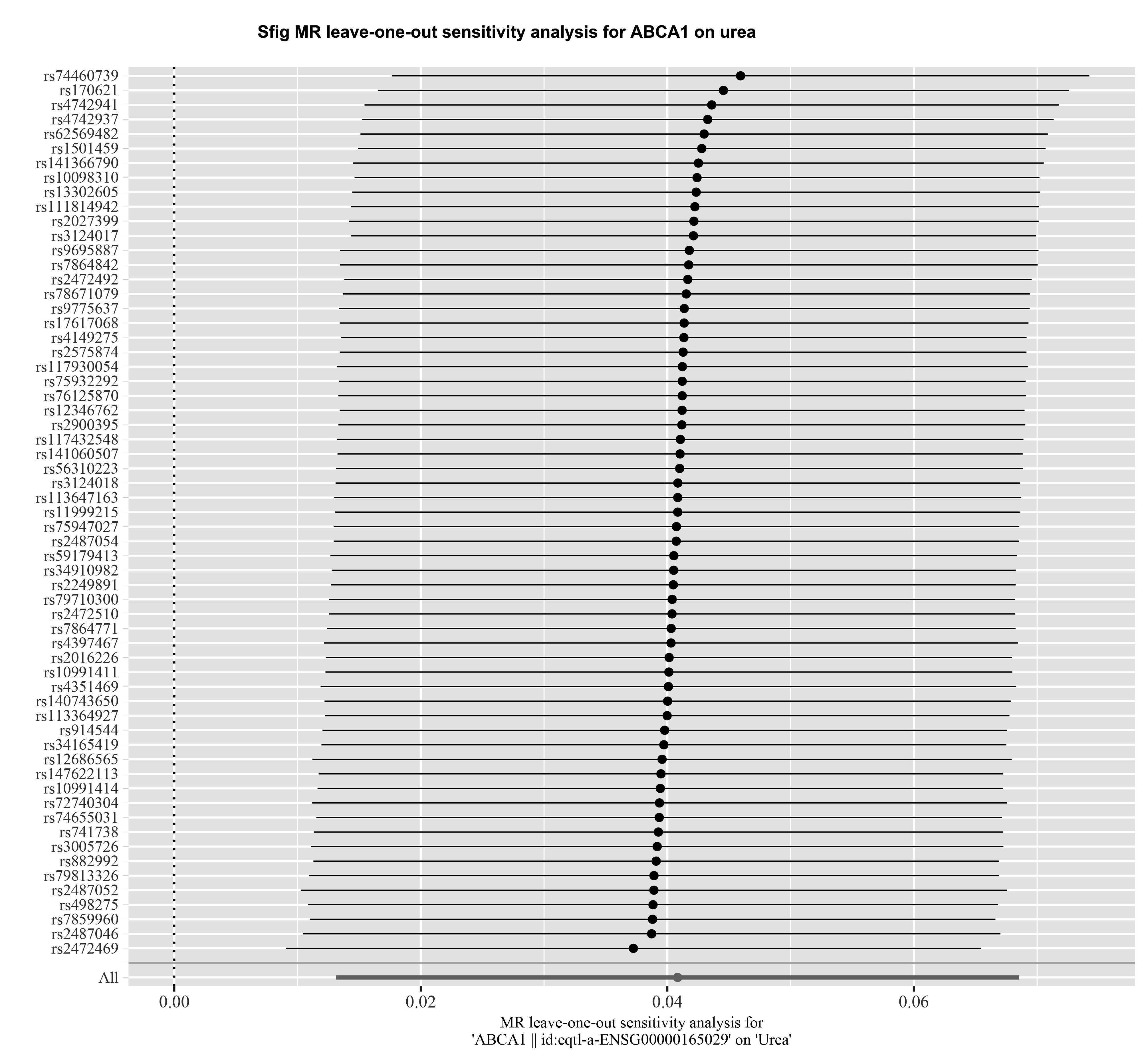

Supplement: Supplementary file 3 — Supplementary Information 3. [file 41598_2025_93644_MOESM3_ESM.zip › leave-one-out analysis/Sfig MR leave-one-out sensitivity analysis for ABCA1 on urea.tif]

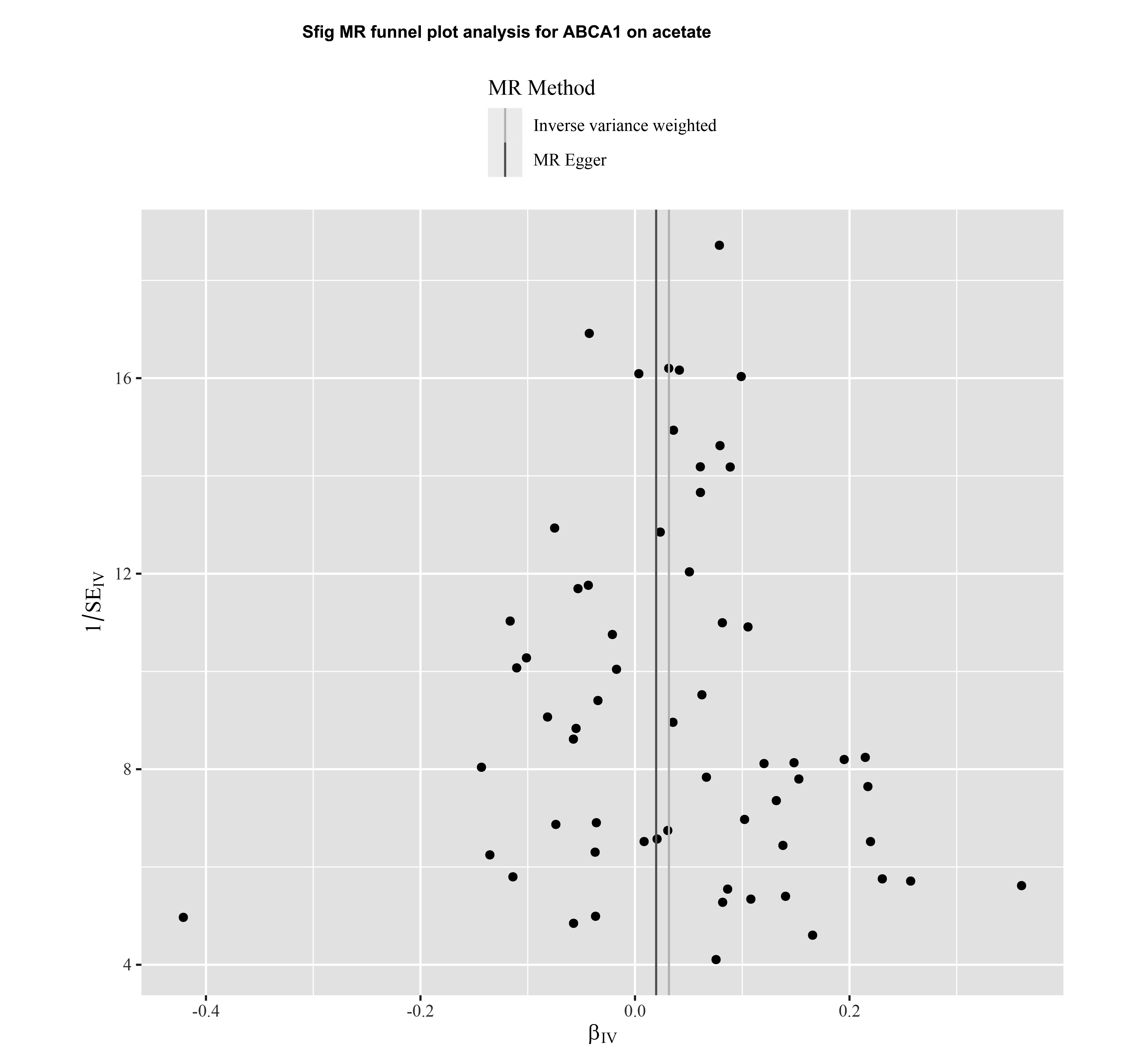

Supplement: Supplementary file 3 — Supplementary Information 3. [file 41598_2025_93644_MOESM3_ESM.zip › the funnel plot/Sfig MR funnel plot analysis for ABCA1 on acetate.tif]

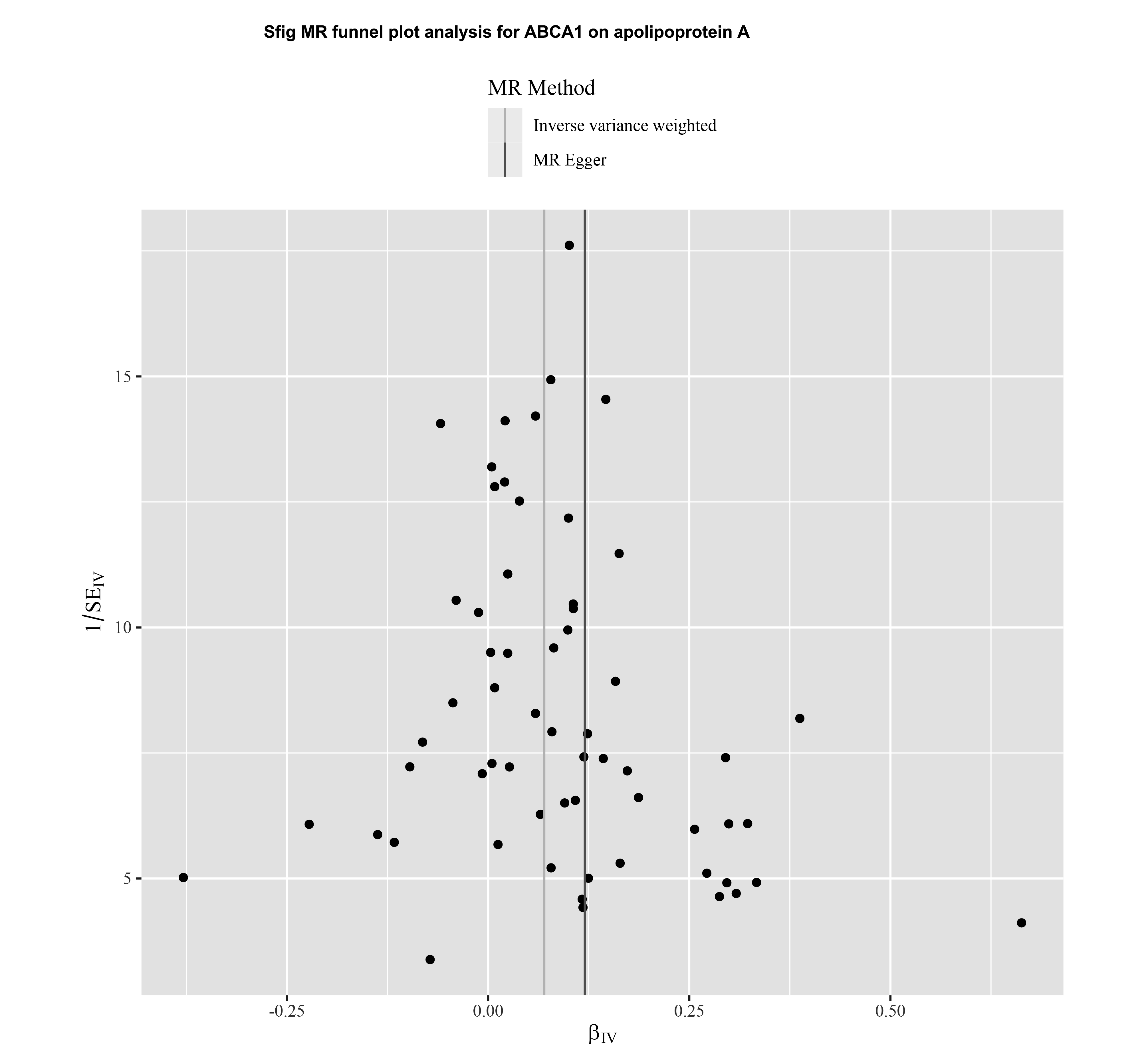

Supplement: Supplementary file 3 — Supplementary Information 3. [file 41598_2025_93644_MOESM3_ESM.zip › the funnel plot/Sfig MR funnel plot analysis for ABCA1 on apolipoprotein A.tif]

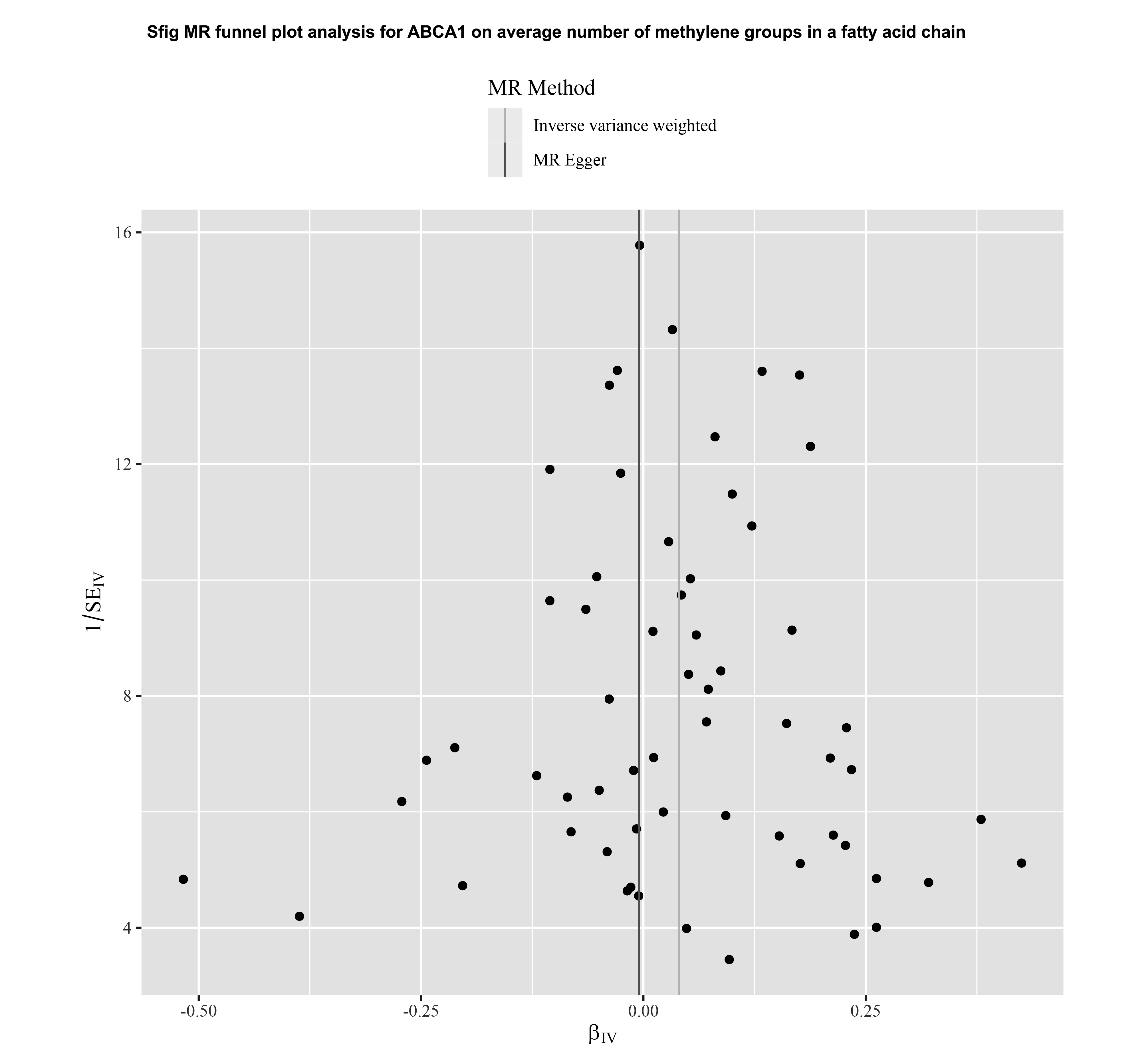

Supplement: Supplementary file 3 — Supplementary Information 3. [file 41598_2025_93644_MOESM3_ESM.zip › the funnel plot/Sfig MR funnel plot analysis for ABCA1 on average number of methylene groups in a fatty acid chain.tif]

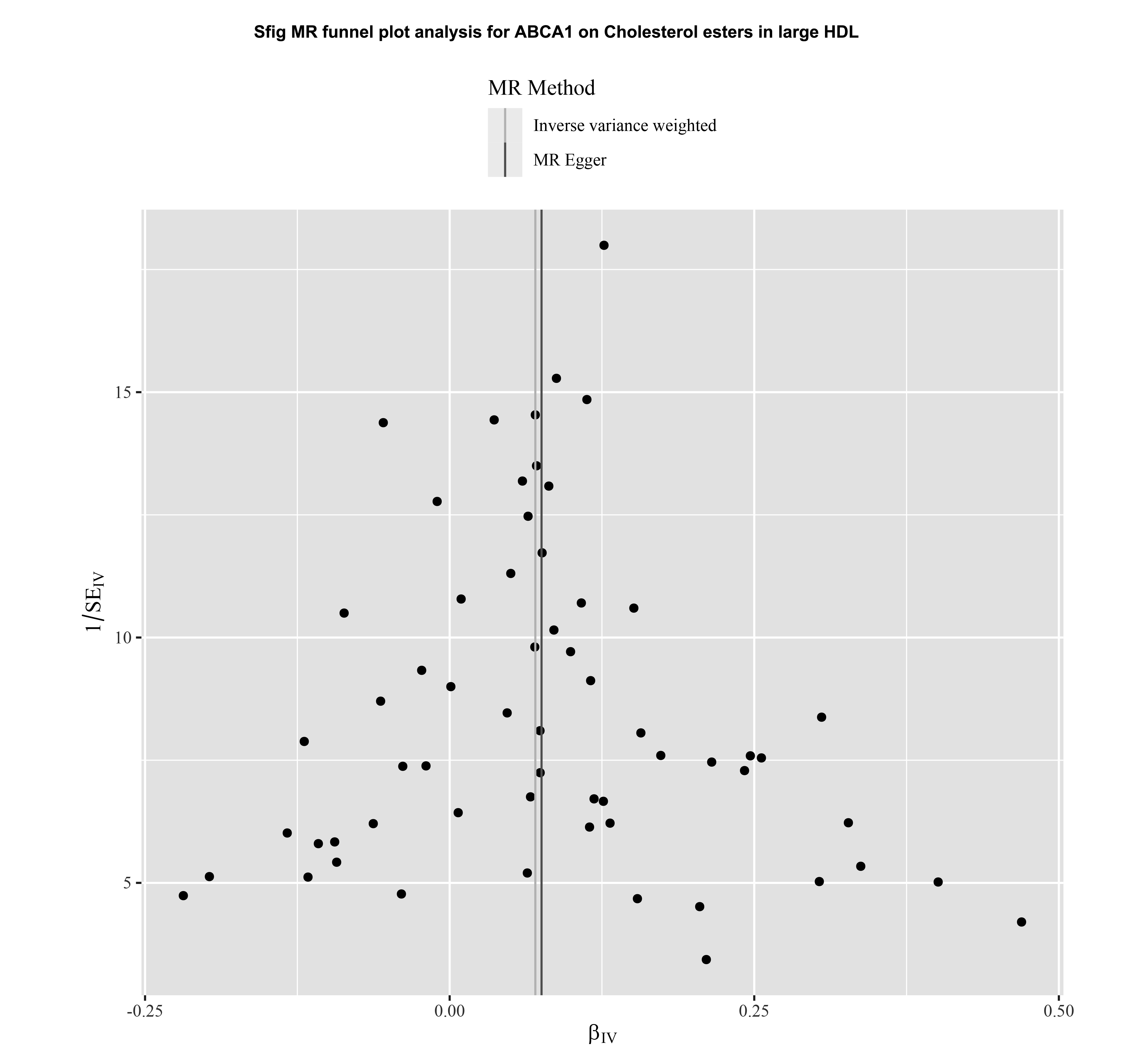

Supplement: Supplementary file 3 — Supplementary Information 3. [file 41598_2025_93644_MOESM3_ESM.zip › the funnel plot/Sfig MR funnel plot analysis for ABCA1 on Cholesterol esters in large HDL.tif]

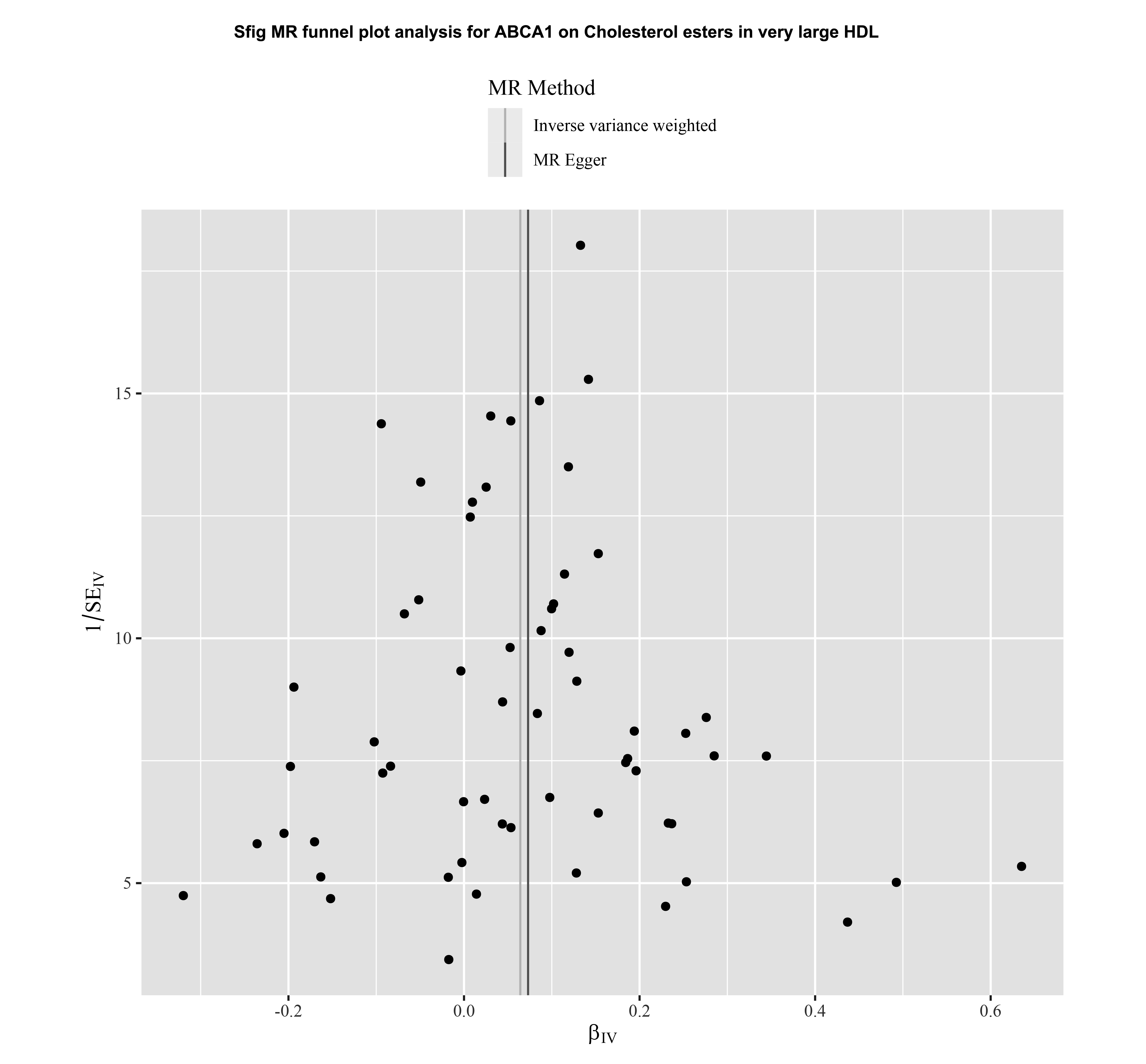

Supplement: Supplementary file 3 — Supplementary Information 3. [file 41598_2025_93644_MOESM3_ESM.zip › the funnel plot/Sfig MR funnel plot analysis for ABCA1 on Cholesterol esters in very large HDL.tif]

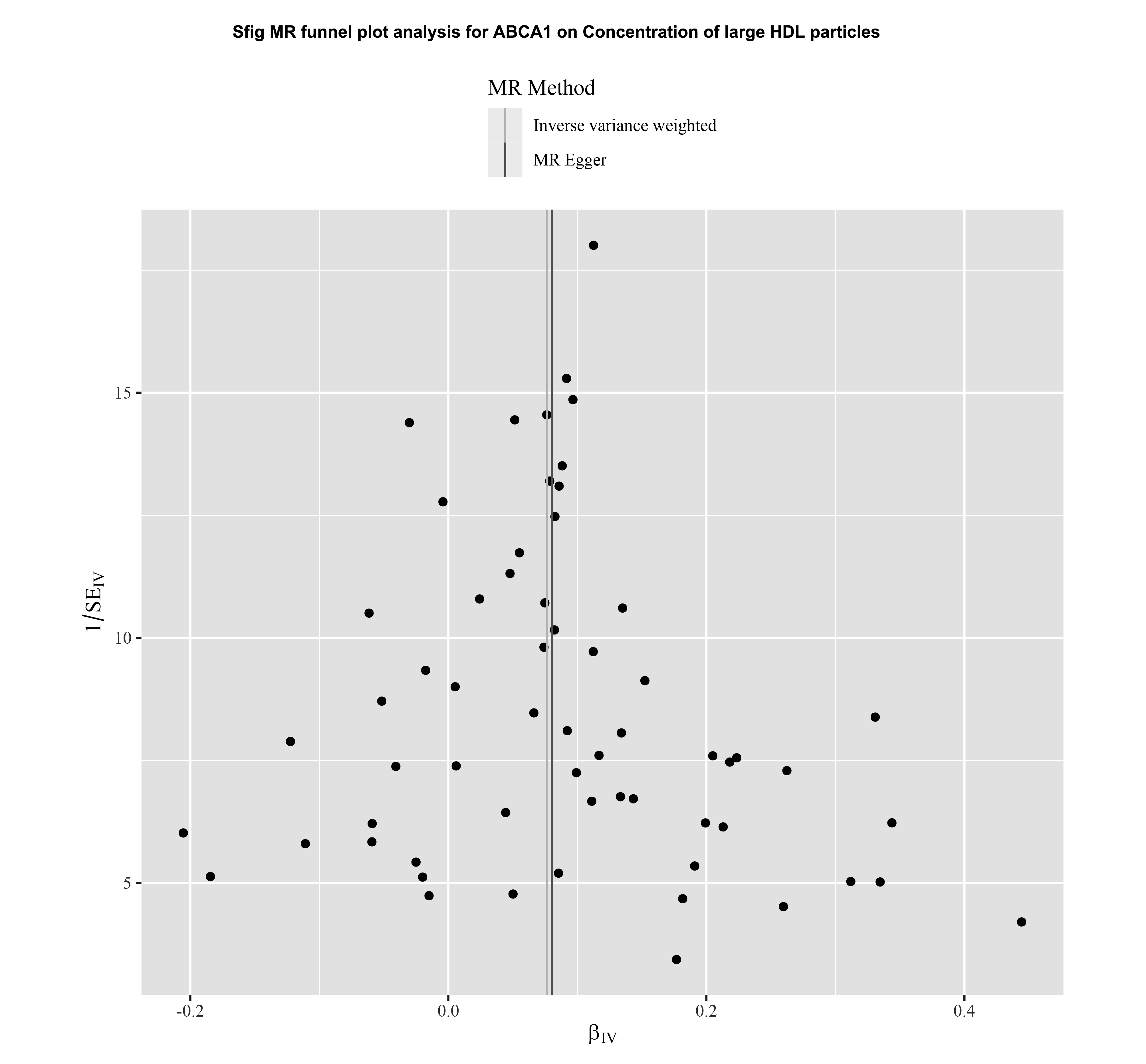

Supplement: Supplementary file 3 — Supplementary Information 3. [file 41598_2025_93644_MOESM3_ESM.zip › the funnel plot/Sfig MR funnel plot analysis for ABCA1 on Concentration of large HDL particles.tif]

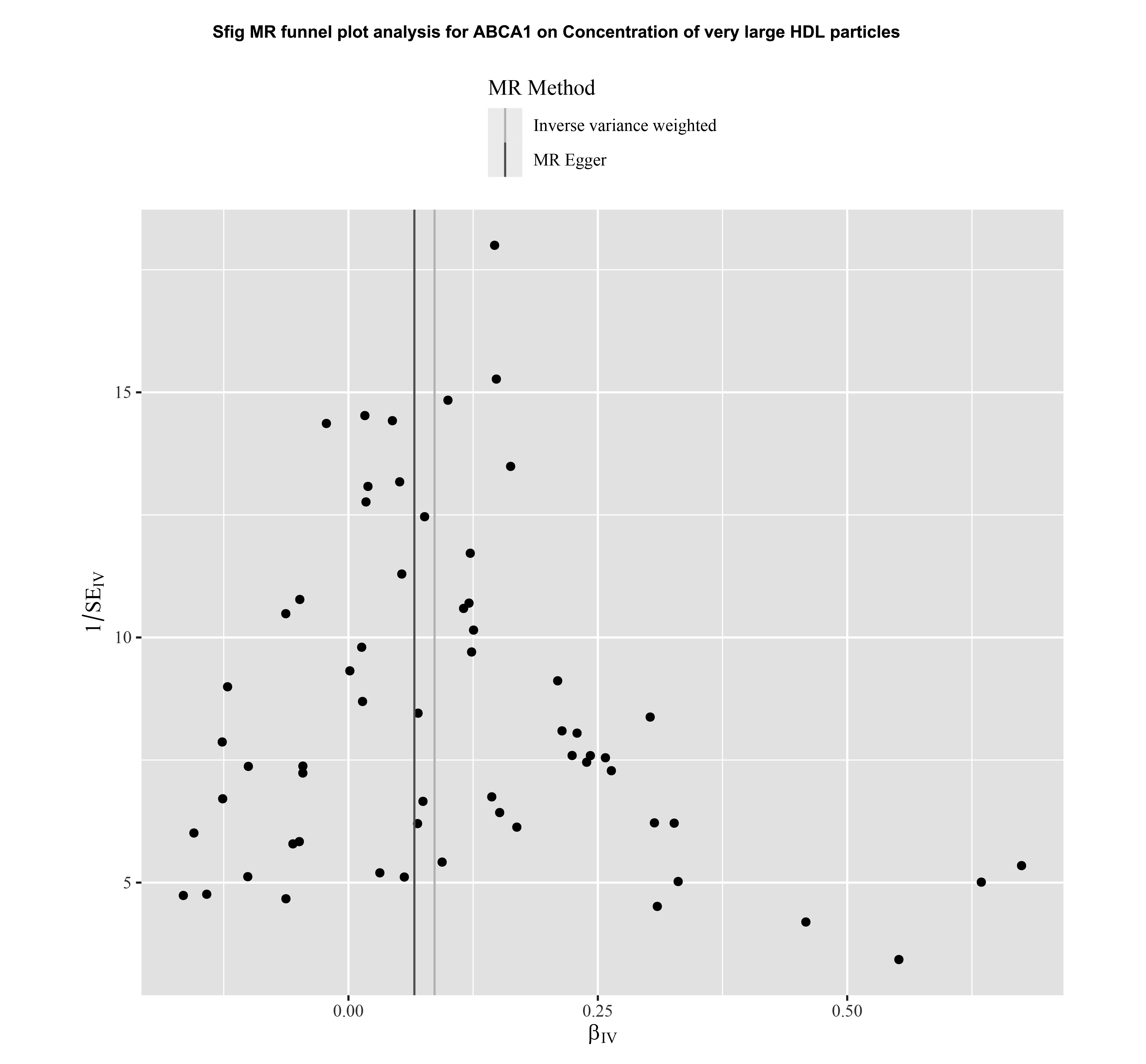

Supplement: Supplementary file 3 — Supplementary Information 3. [file 41598_2025_93644_MOESM3_ESM.zip › the funnel plot/Sfig MR funnel plot analysis for ABCA1 on Concentration of very large HDL particles.tif]

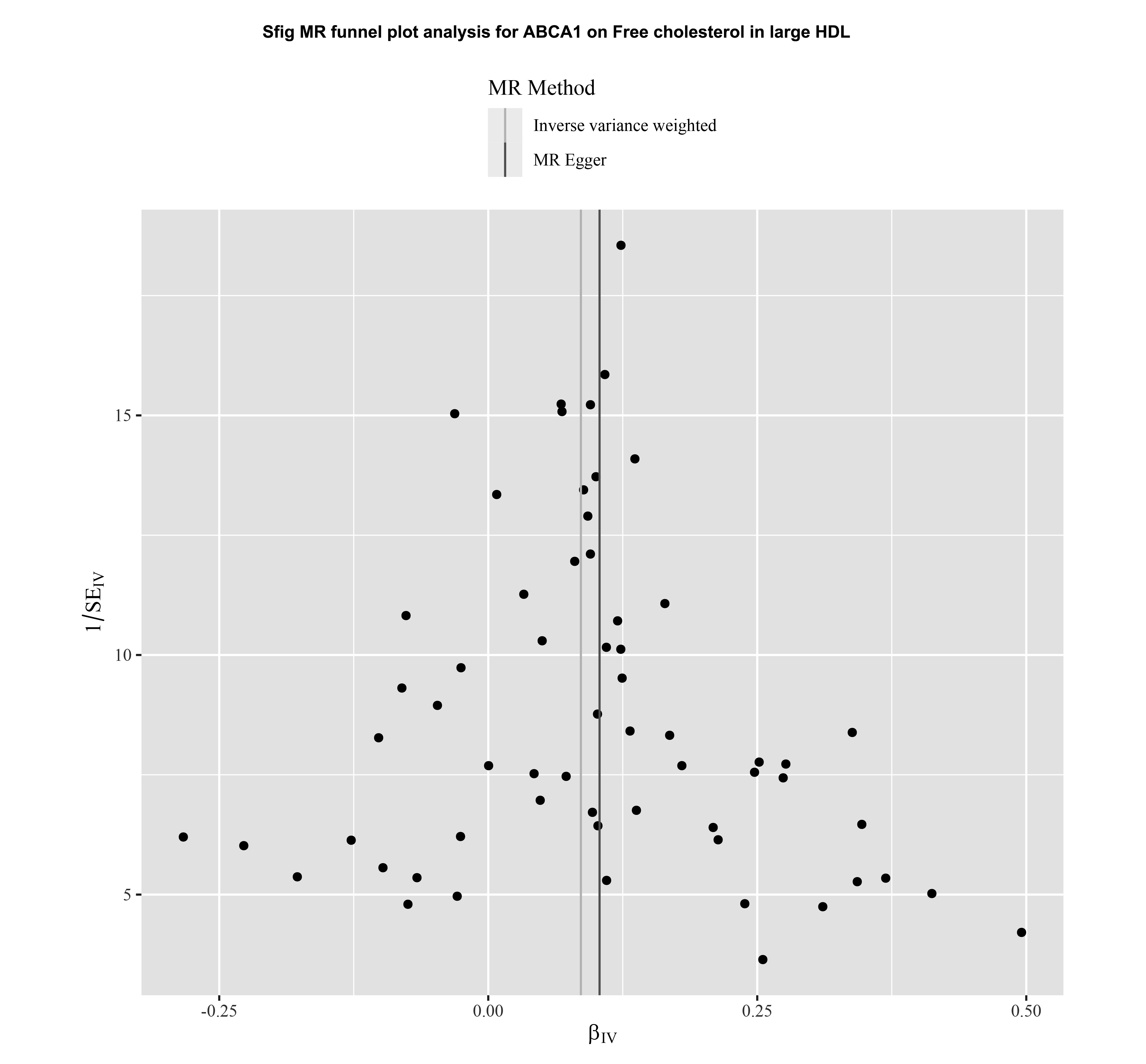

Supplement: Supplementary file 3 — Supplementary Information 3. [file 41598_2025_93644_MOESM3_ESM.zip › the funnel plot/Sfig MR funnel plot analysis for ABCA1 on Free cholesterol in large HDL.tif]

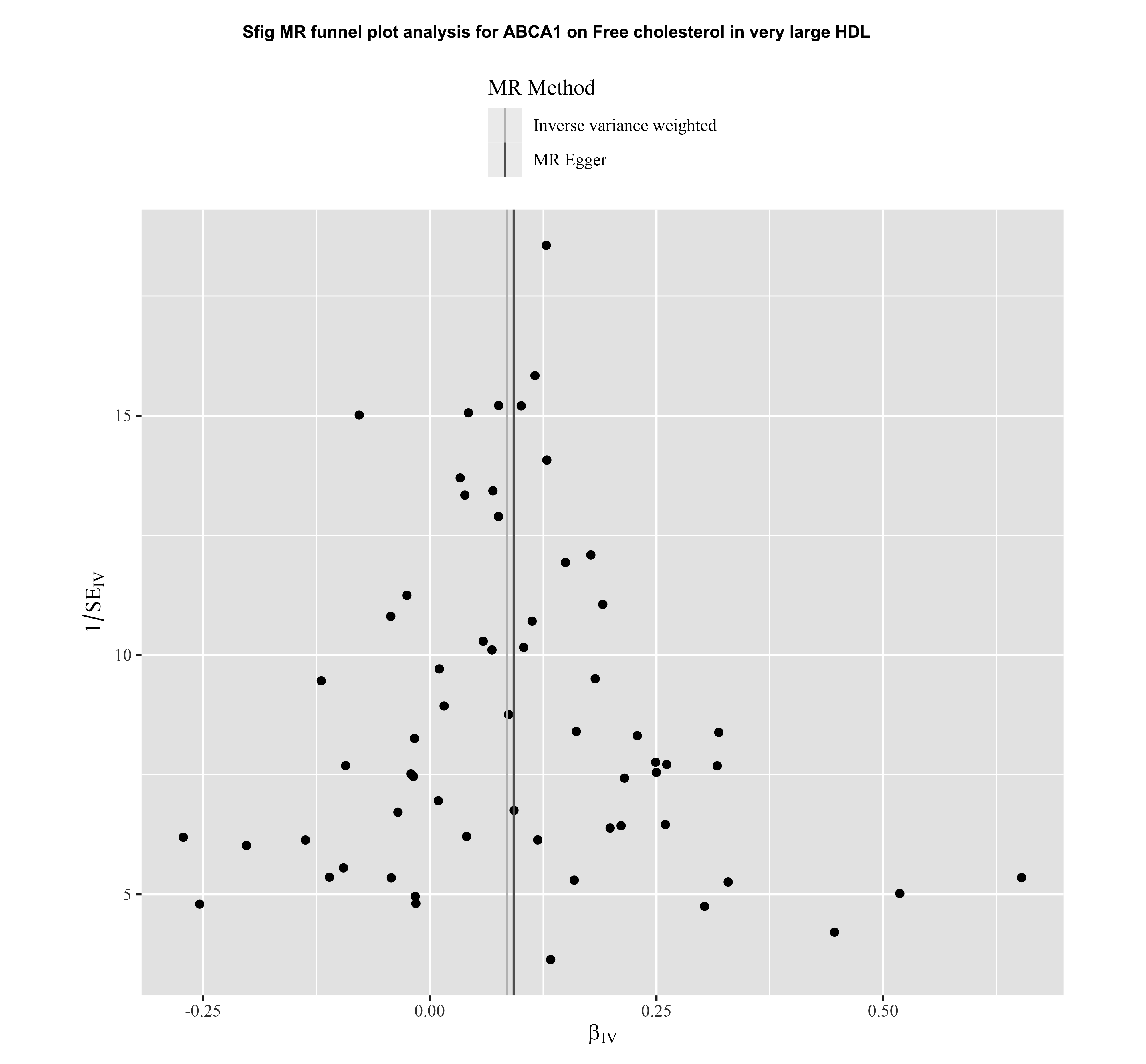

Supplement: Supplementary file 3 — Supplementary Information 3. [file 41598_2025_93644_MOESM3_ESM.zip › the funnel plot/Sfig MR funnel plot analysis for ABCA1 on Free cholesterol in very large HDL.tif]

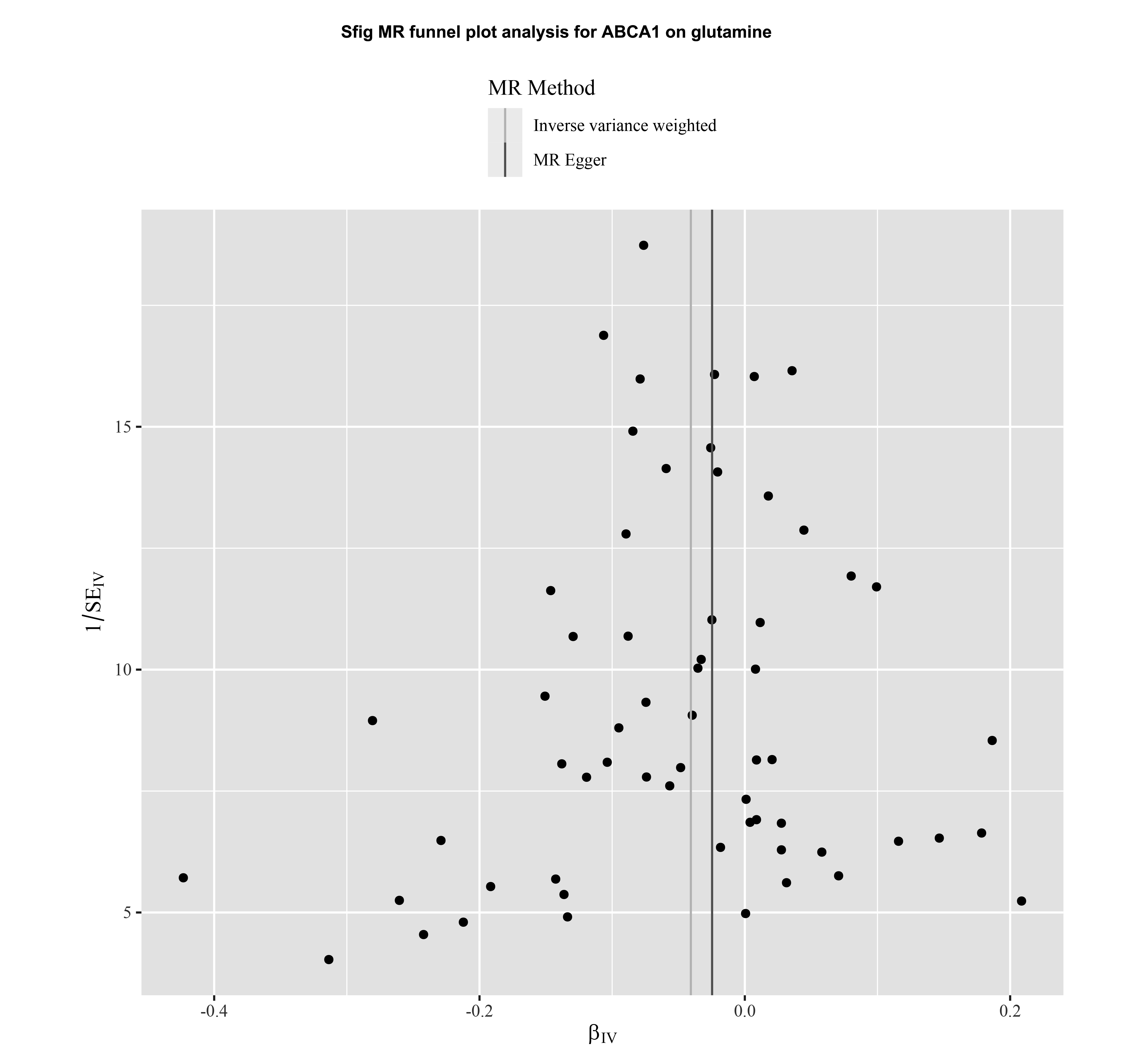

Supplement: Supplementary file 3 — Supplementary Information 3. [file 41598_2025_93644_MOESM3_ESM.zip › the funnel plot/Sfig MR funnel plot analysis for ABCA1 on glutamine.tif]

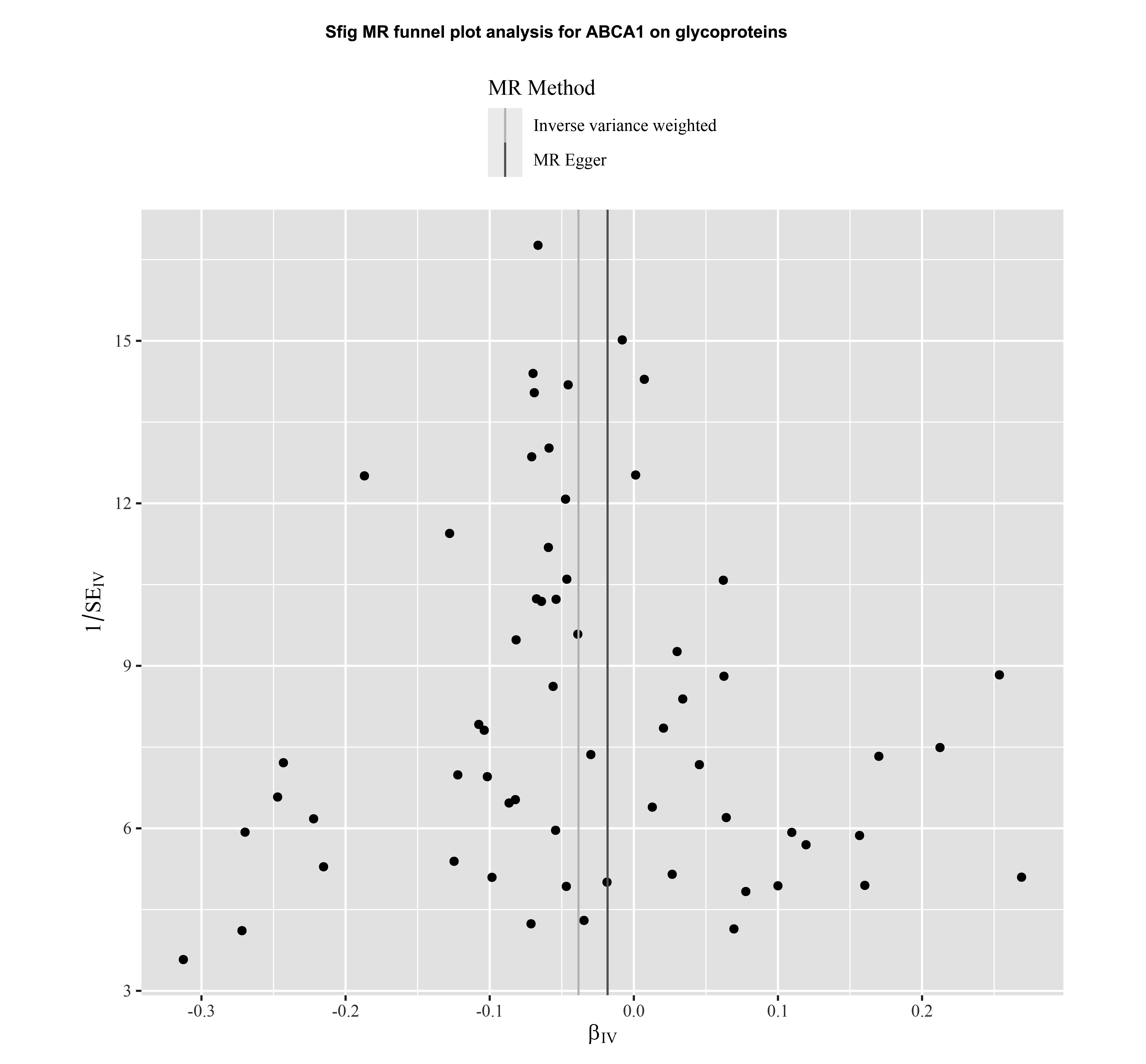

Supplement: Supplementary file 3 — Supplementary Information 3. [file 41598_2025_93644_MOESM3_ESM.zip › the funnel plot/Sfig MR funnel plot analysis for ABCA1 on glycoproteins.tif]

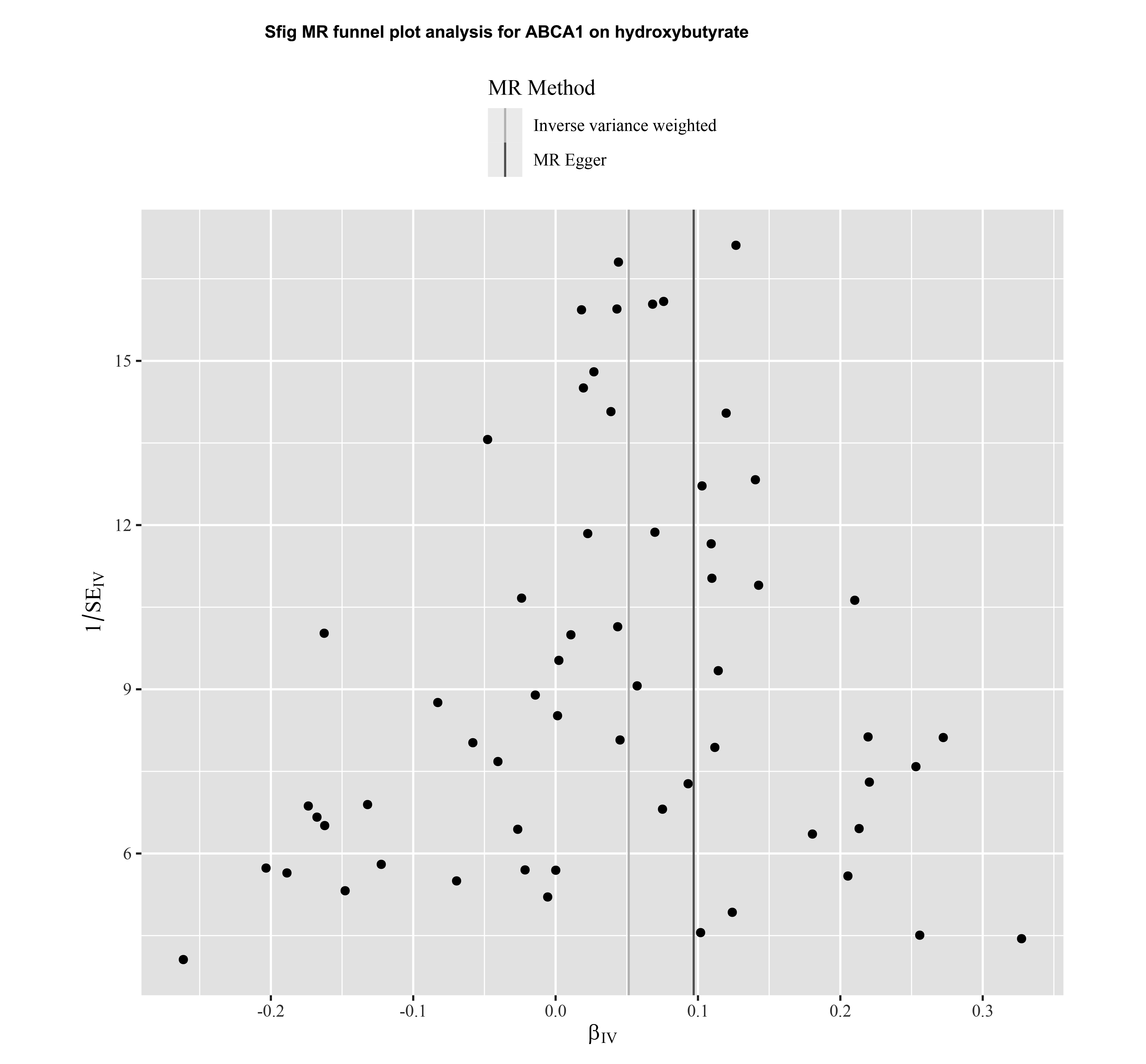

Supplement: Supplementary file 3 — Supplementary Information 3. [file 41598_2025_93644_MOESM3_ESM.zip › the funnel plot/Sfig MR funnel plot analysis for ABCA1 on hydroxybutyrate.tif]

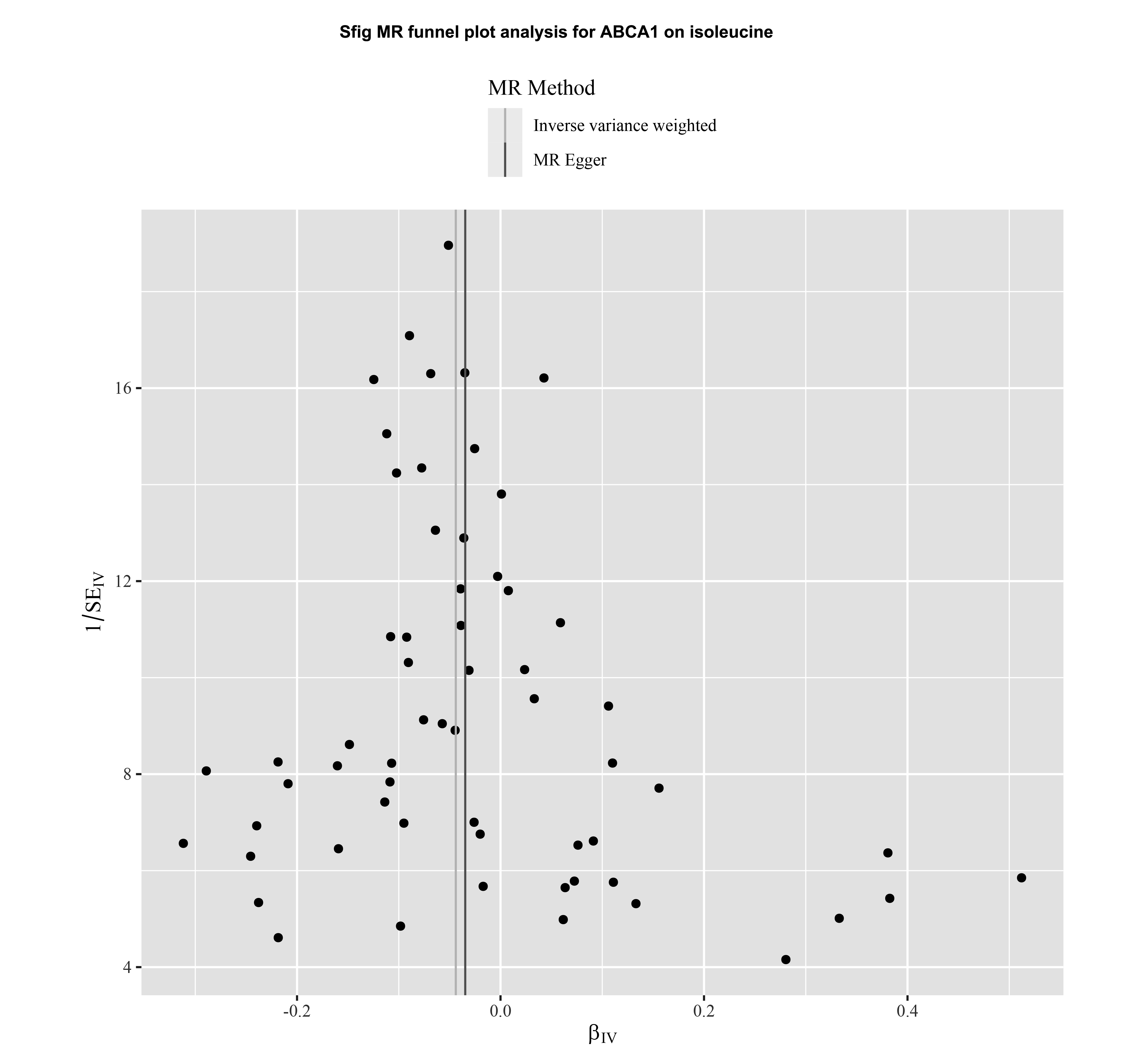

Supplement: Supplementary file 3 — Supplementary Information 3. [file 41598_2025_93644_MOESM3_ESM.zip › the funnel plot/Sfig MR funnel plot analysis for ABCA1 on isoleucine.tif]

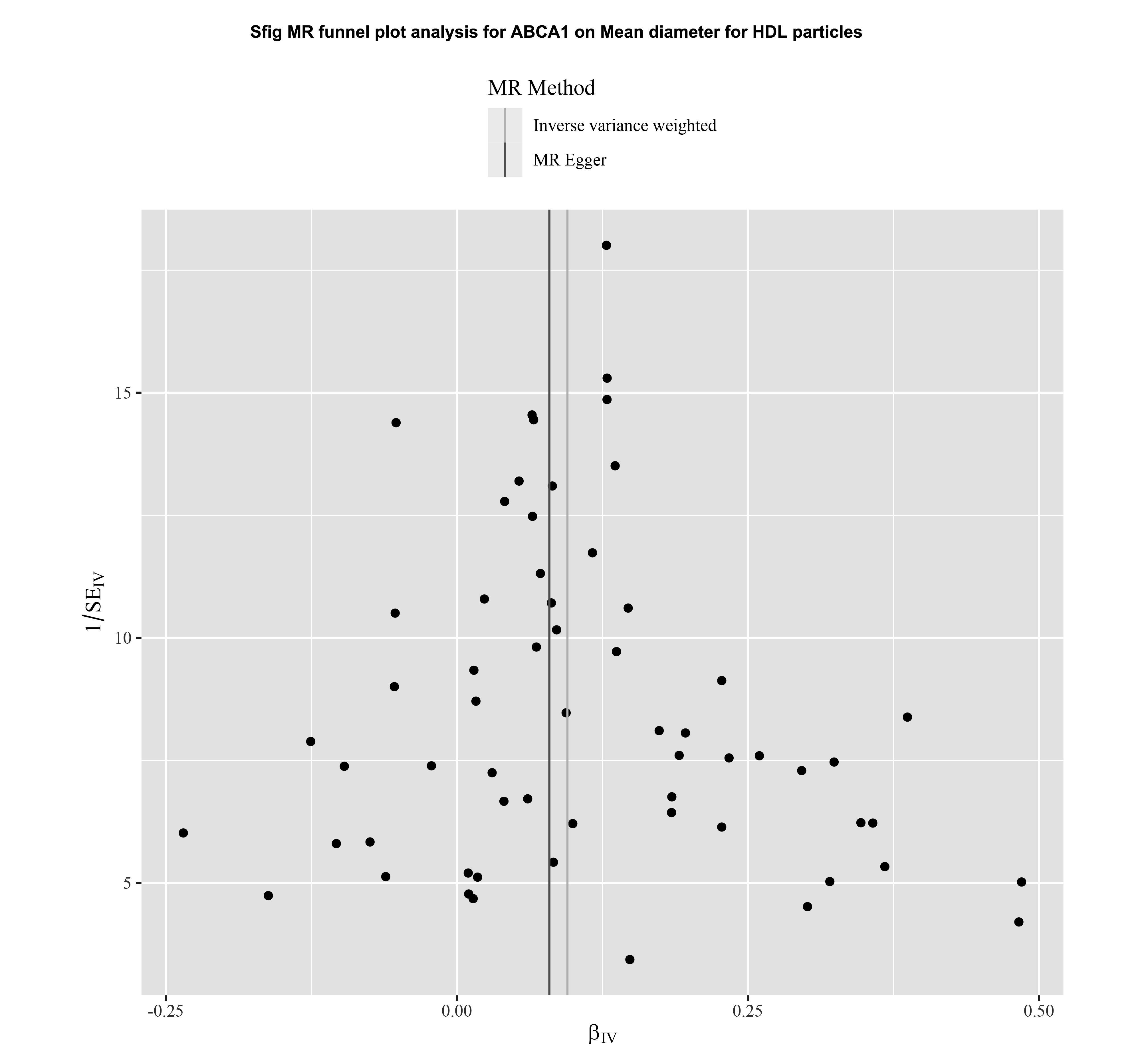

Supplement: Supplementary file 3 — Supplementary Information 3. [file 41598_2025_93644_MOESM3_ESM.zip › the funnel plot/Sfig MR funnel plot analysis for ABCA1 on Mean diameter for HDL particles.tif]

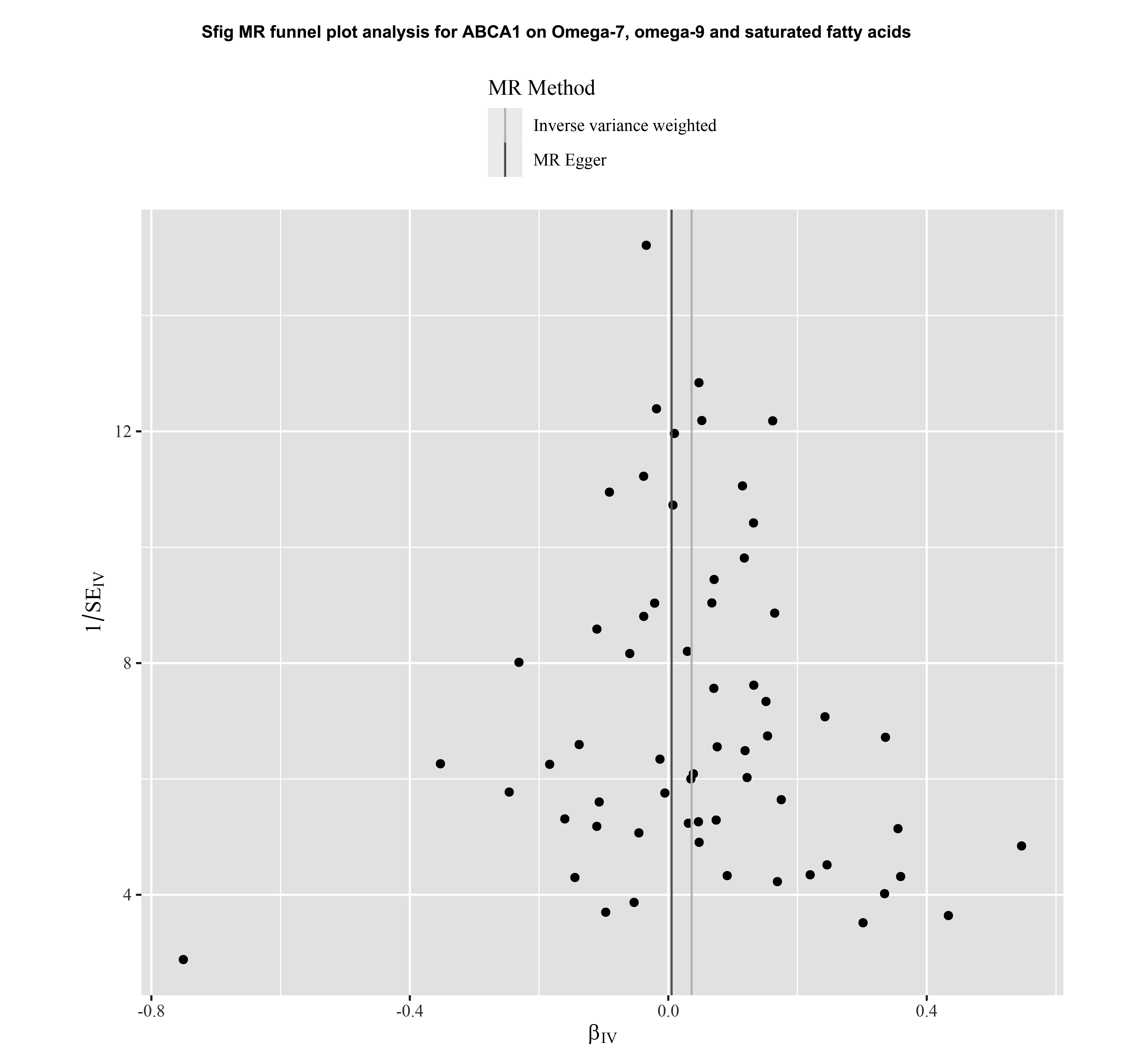

Supplement: Supplementary file 3 — Supplementary Information 3. [file 41598_2025_93644_MOESM3_ESM.zip › the funnel plot/Sfig MR funnel plot analysis for ABCA1 on Omega-7, omega-9 and saturated fatty acids.tif]

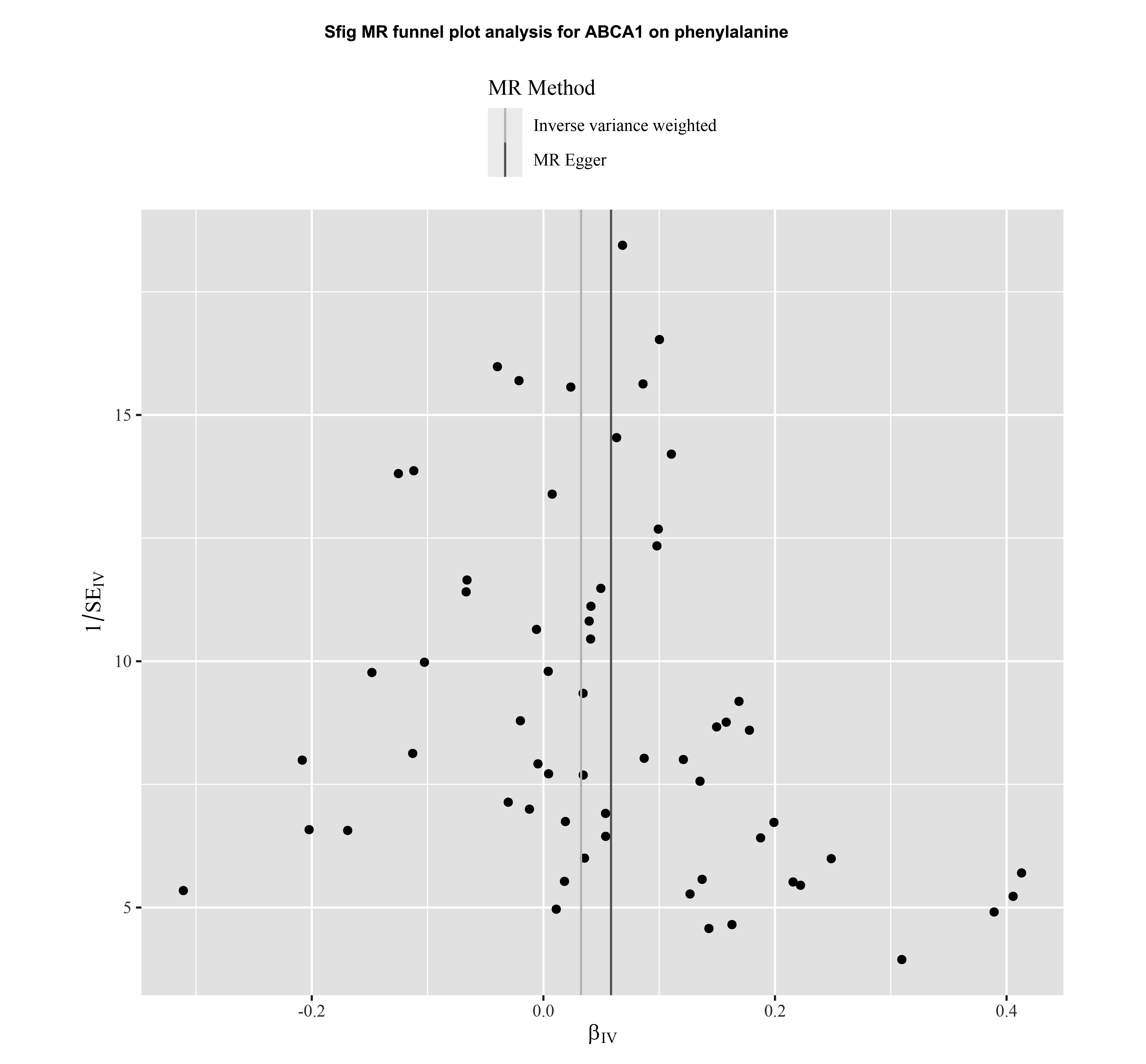

Supplement: Supplementary file 3 — Supplementary Information 3. [file 41598_2025_93644_MOESM3_ESM.zip › the funnel plot/Sfig MR funnel plot analysis for ABCA1 on phenylalanine.tif]
