# Supplementary figures and images for: Mendelian randomization reveals probucol’s preventive role in Behçet’s disease via circulating metabolites (part 2 of 2)
Source: Sci Rep. 2025 Mar 21;15:9722. doi: 10.1038/s41598-025-93644-8 (PMC11928609; doi:10.1038/s41598-025-93644-8)

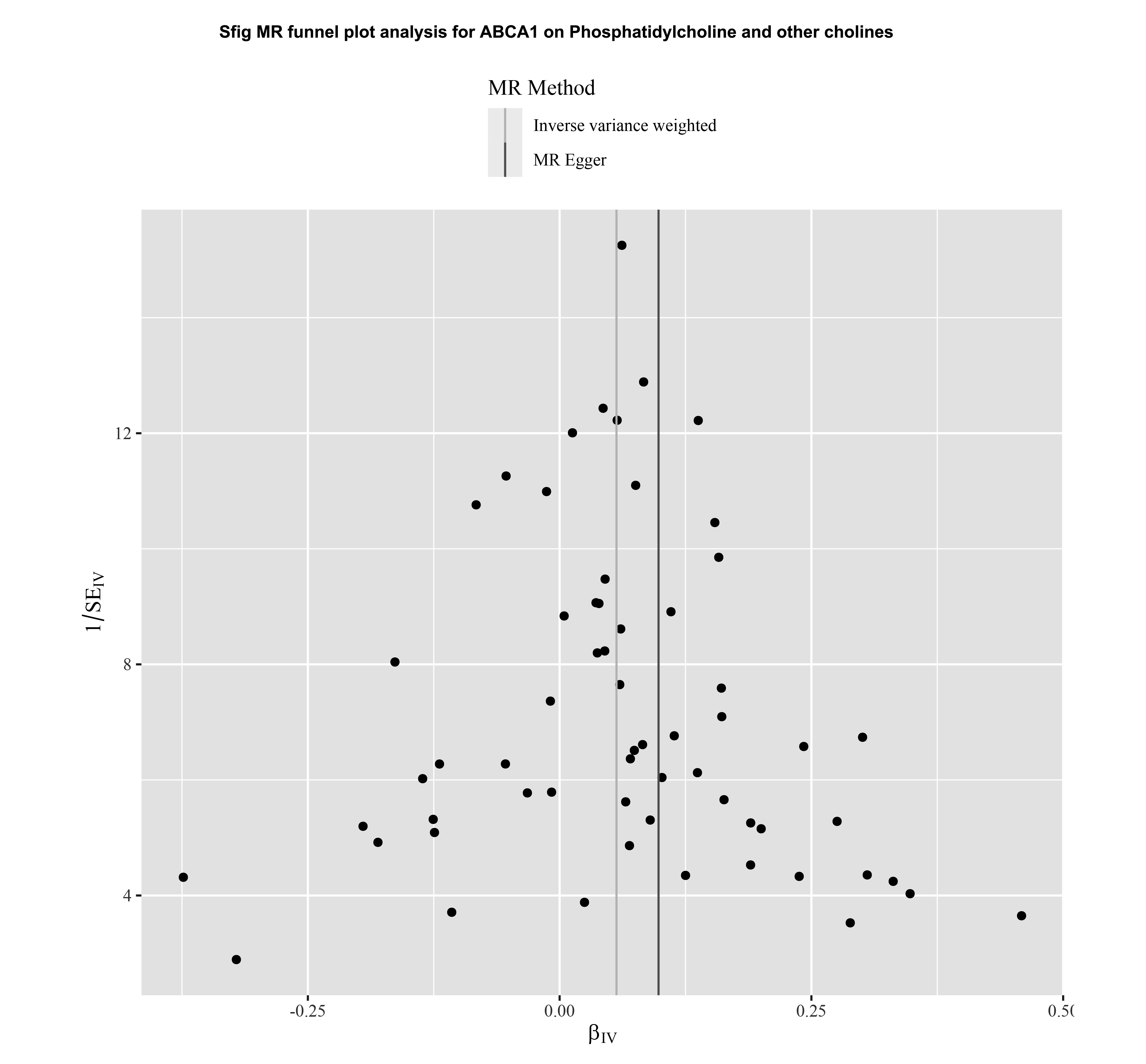

Supplement: Supplementary file 3 — Supplementary Information 3. [file 41598_2025_93644_MOESM3_ESM.zip › the funnel plot/Sfig MR funnel plot analysis for ABCA1 on Phosphatidylcholine and other cholines.tif]

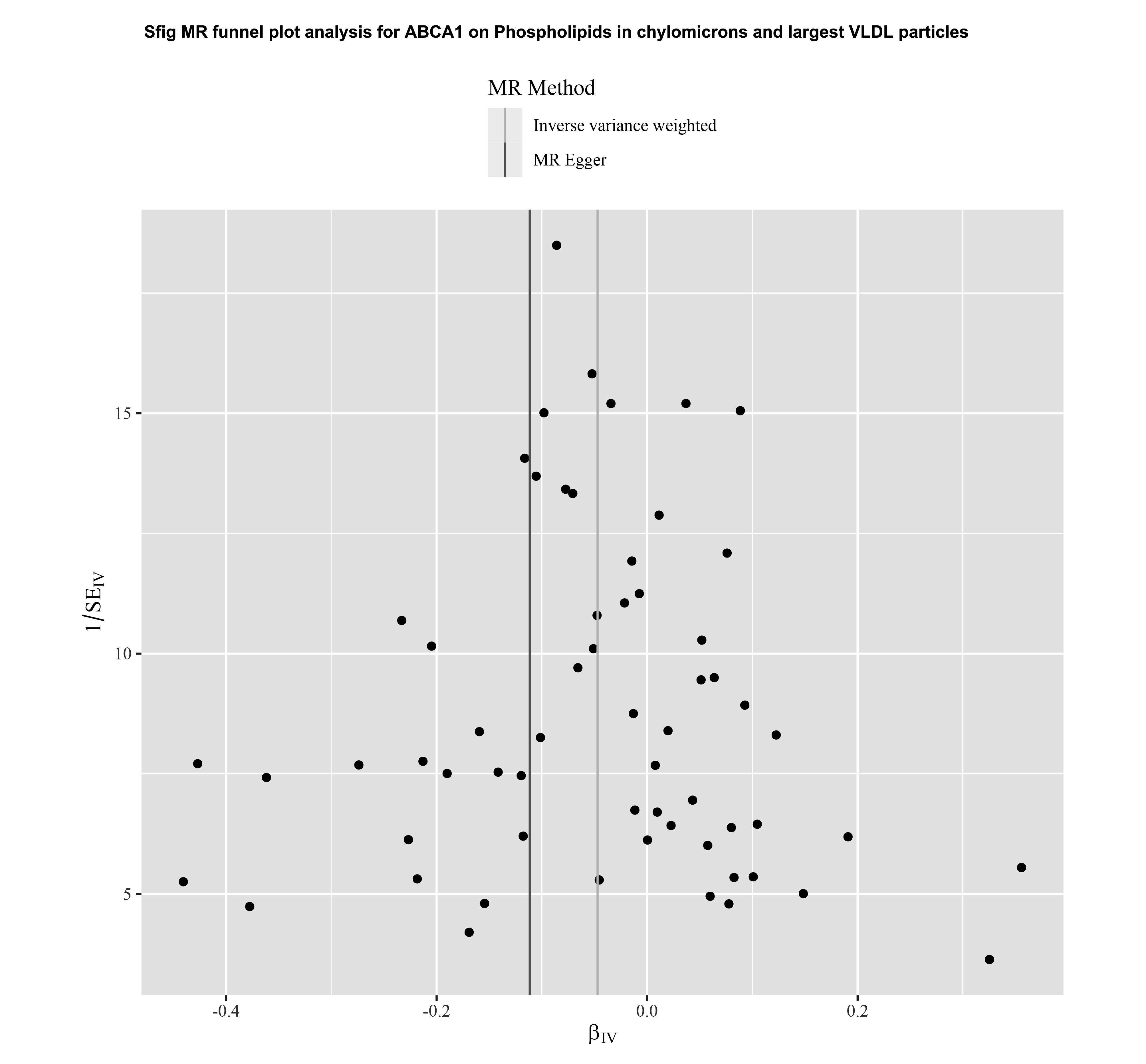

Supplement: Supplementary file 3 — Supplementary Information 3. [file 41598_2025_93644_MOESM3_ESM.zip › the funnel plot/Sfig MR funnel plot analysis for ABCA1 on Phospholipids in chylomicrons and largest VLDL particles.tif]

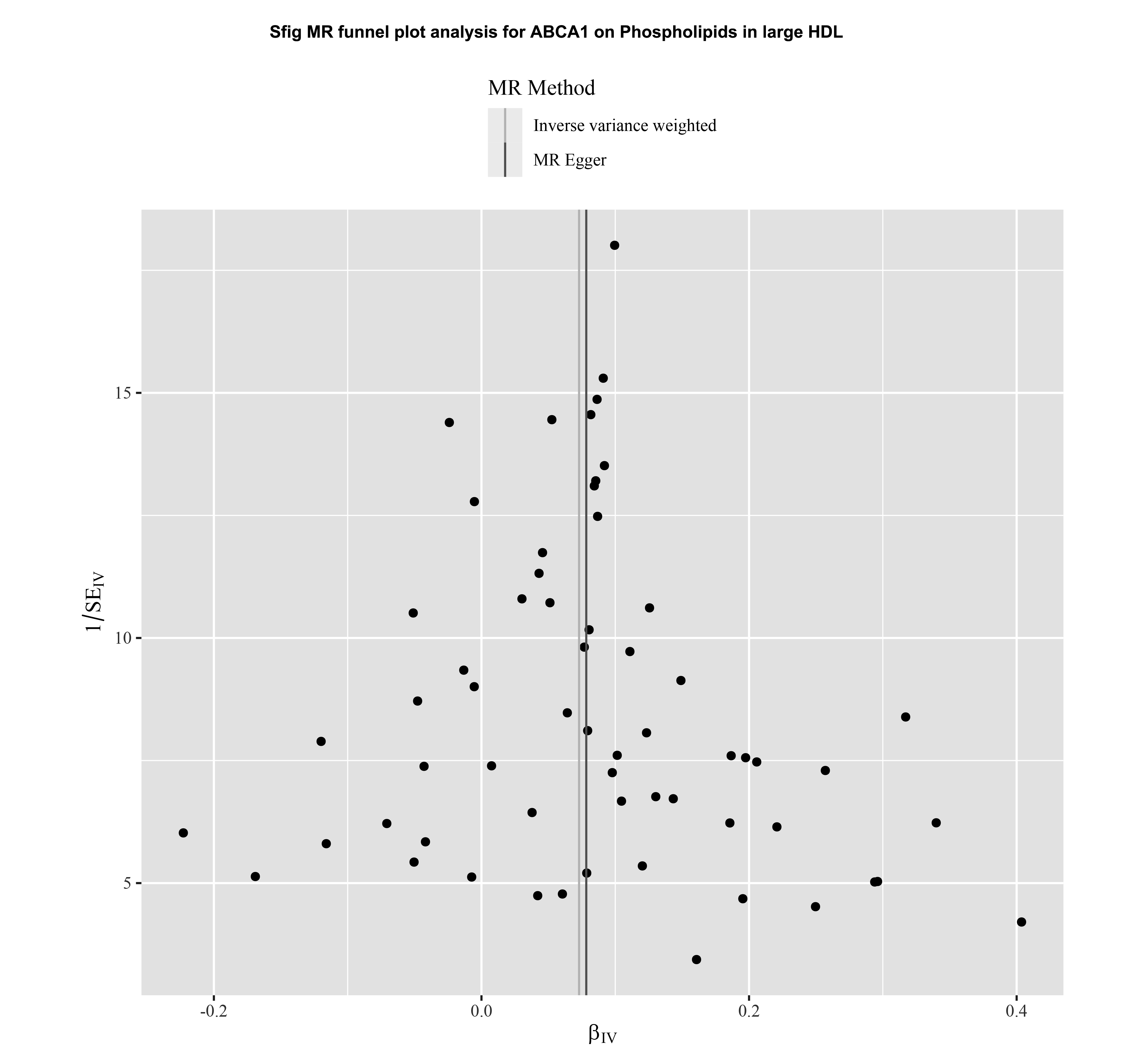

Supplement: Supplementary file 3 — Supplementary Information 3. [file 41598_2025_93644_MOESM3_ESM.zip › the funnel plot/Sfig MR funnel plot analysis for ABCA1 on Phospholipids in large HDL.tif]

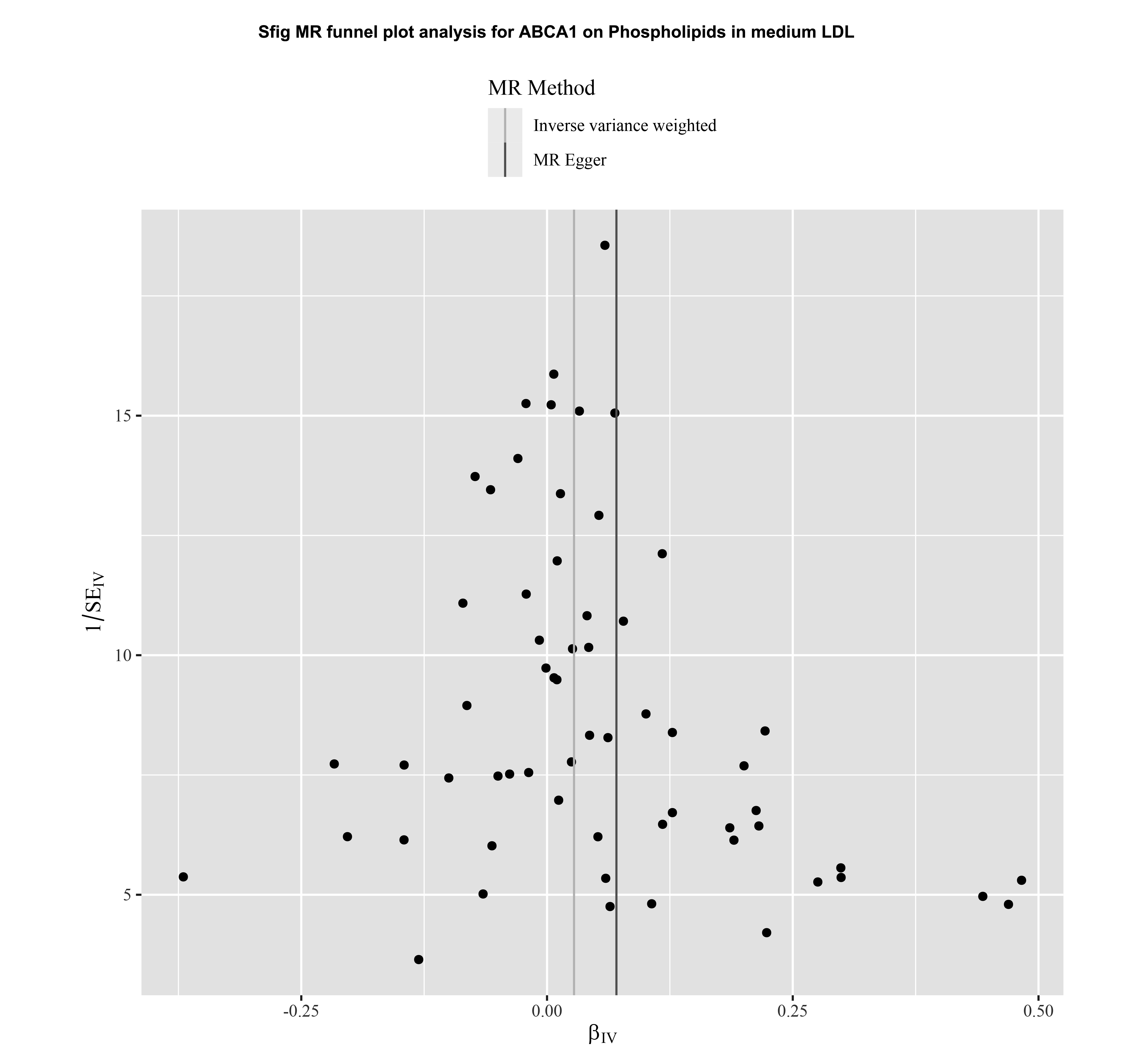

Supplement: Supplementary file 3 — Supplementary Information 3. [file 41598_2025_93644_MOESM3_ESM.zip › the funnel plot/Sfig MR funnel plot analysis for ABCA1 on Phospholipids in medium LDL.tif]

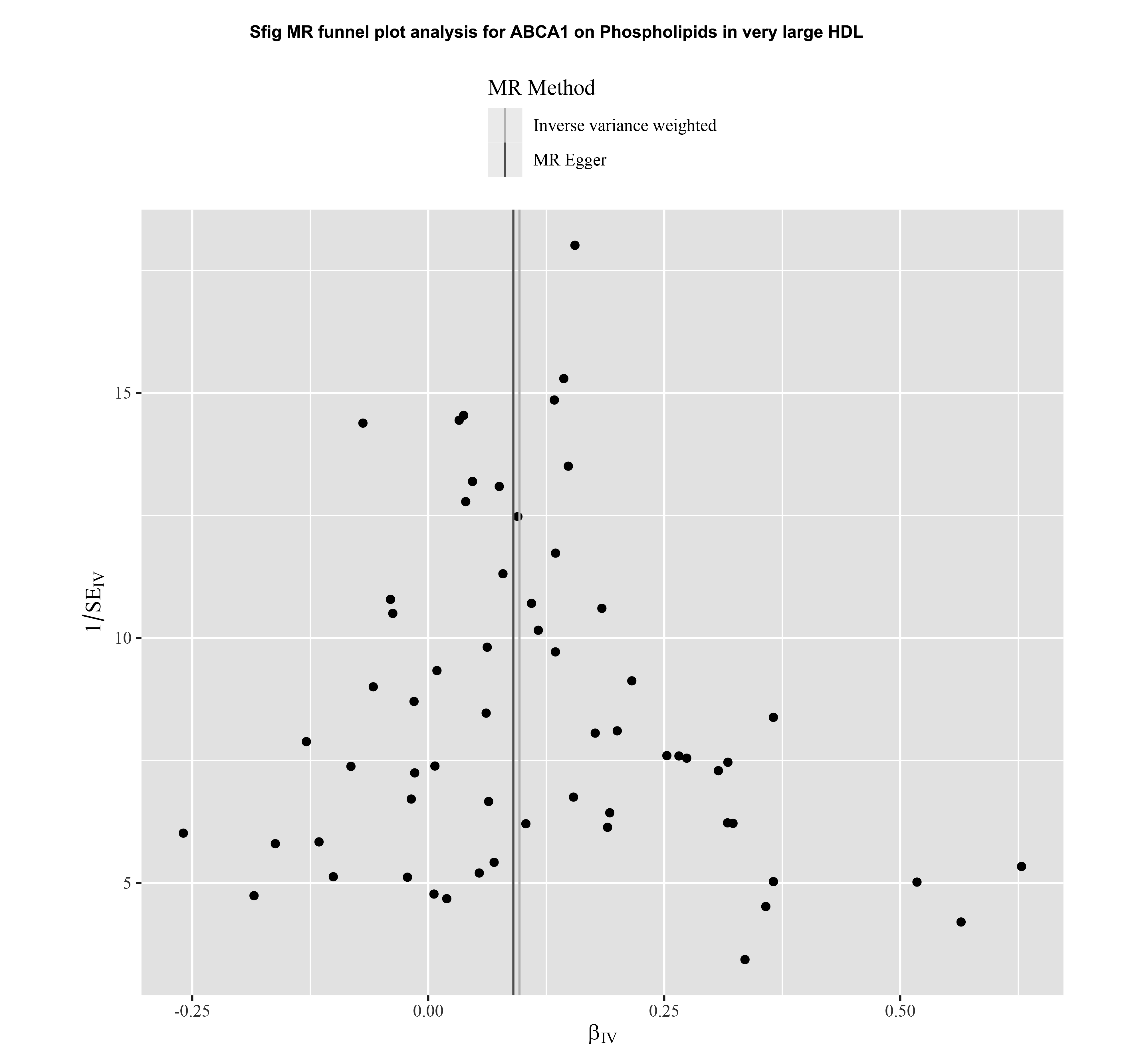

Supplement: Supplementary file 3 — Supplementary Information 3. [file 41598_2025_93644_MOESM3_ESM.zip › the funnel plot/Sfig MR funnel plot analysis for ABCA1 on Phospholipids in very large HDL.tif]

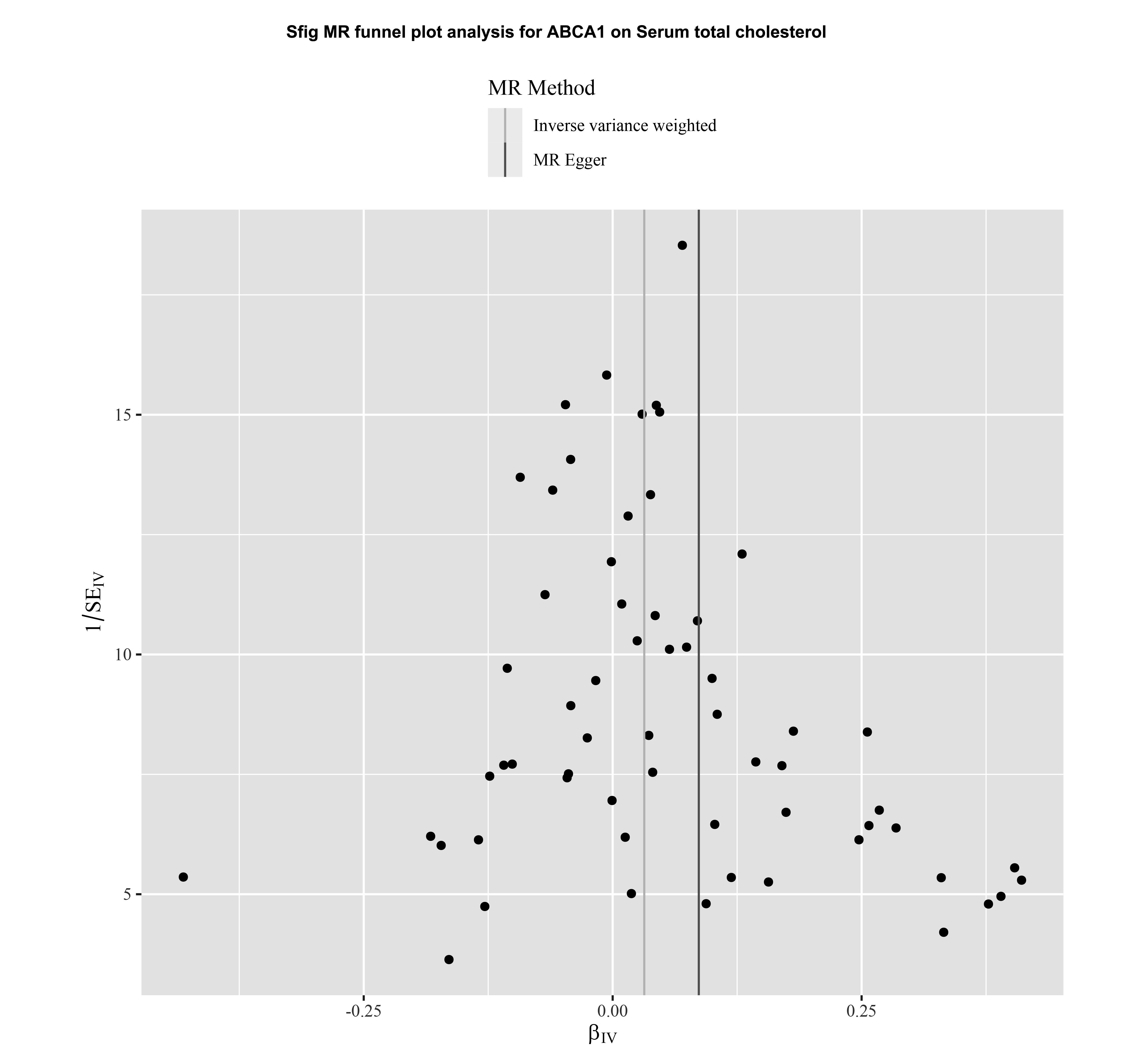

Supplement: Supplementary file 3 — Supplementary Information 3. [file 41598_2025_93644_MOESM3_ESM.zip › the funnel plot/Sfig MR funnel plot analysis for ABCA1 on Serum total cholesterol.tif]

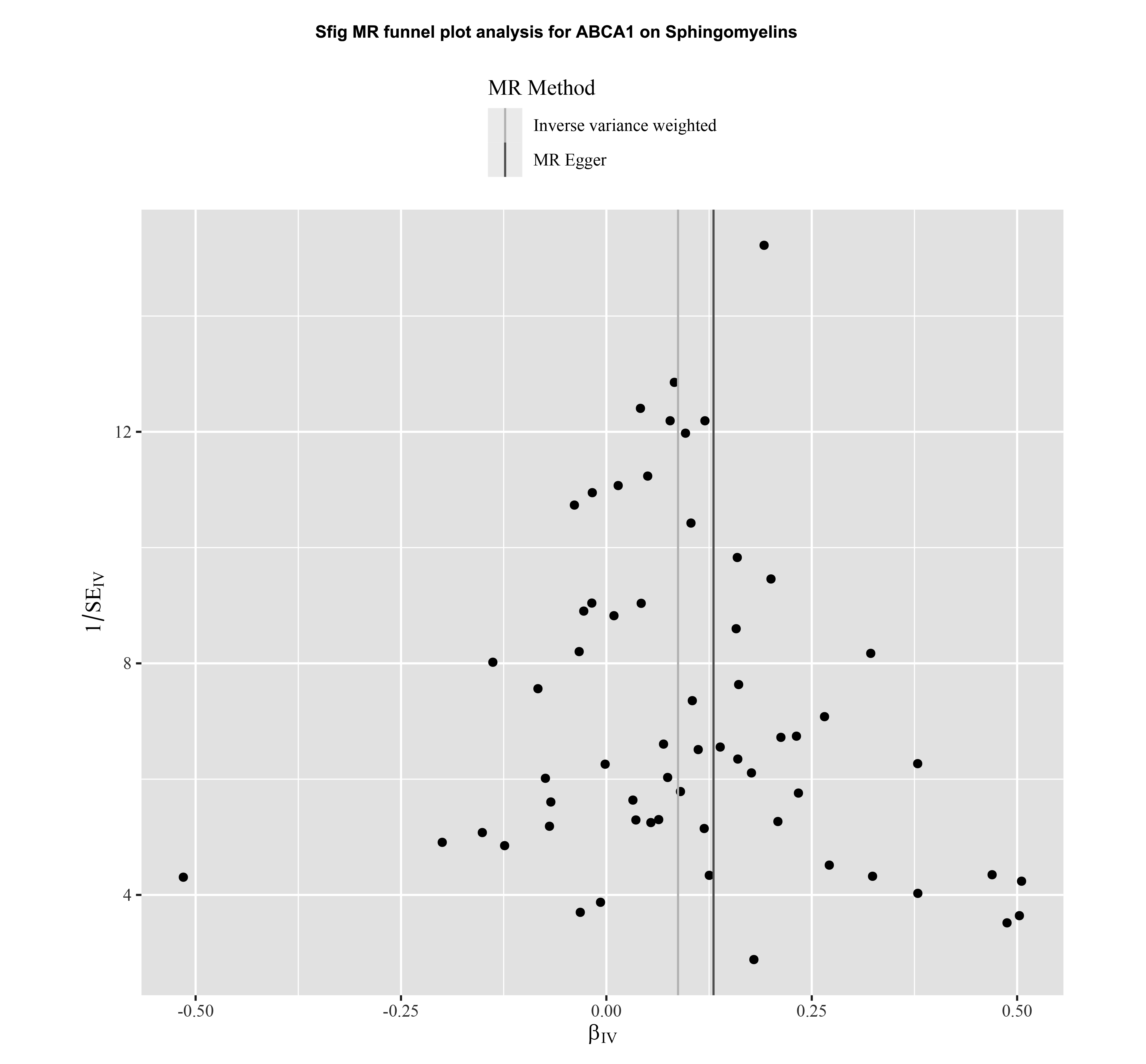

Supplement: Supplementary file 3 — Supplementary Information 3. [file 41598_2025_93644_MOESM3_ESM.zip › the funnel plot/Sfig MR funnel plot analysis for ABCA1 on Sphingomyelins.tif]

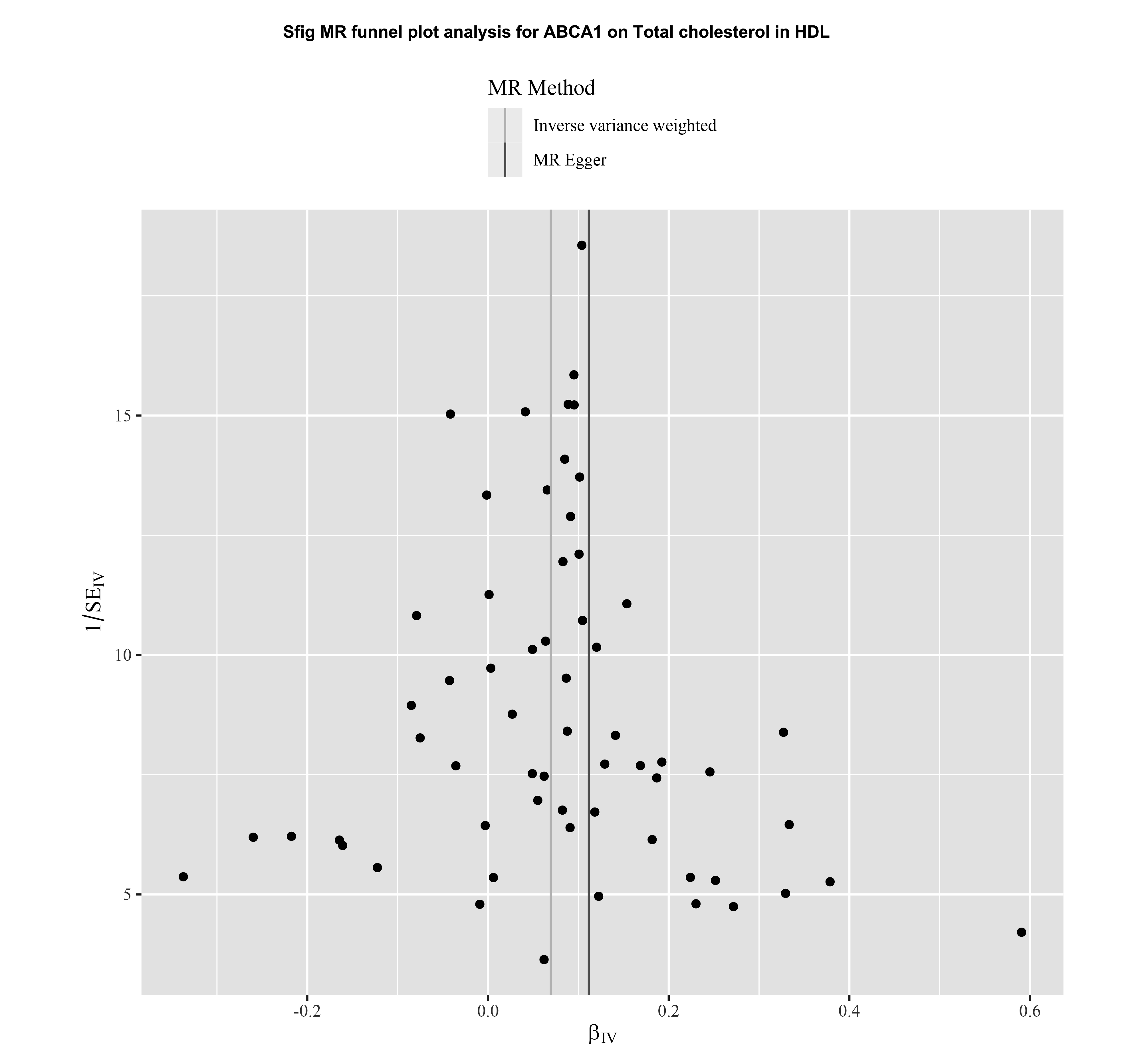

Supplement: Supplementary file 3 — Supplementary Information 3. [file 41598_2025_93644_MOESM3_ESM.zip › the funnel plot/Sfig MR funnel plot analysis for ABCA1 on Total cholesterol in HDL.tif]

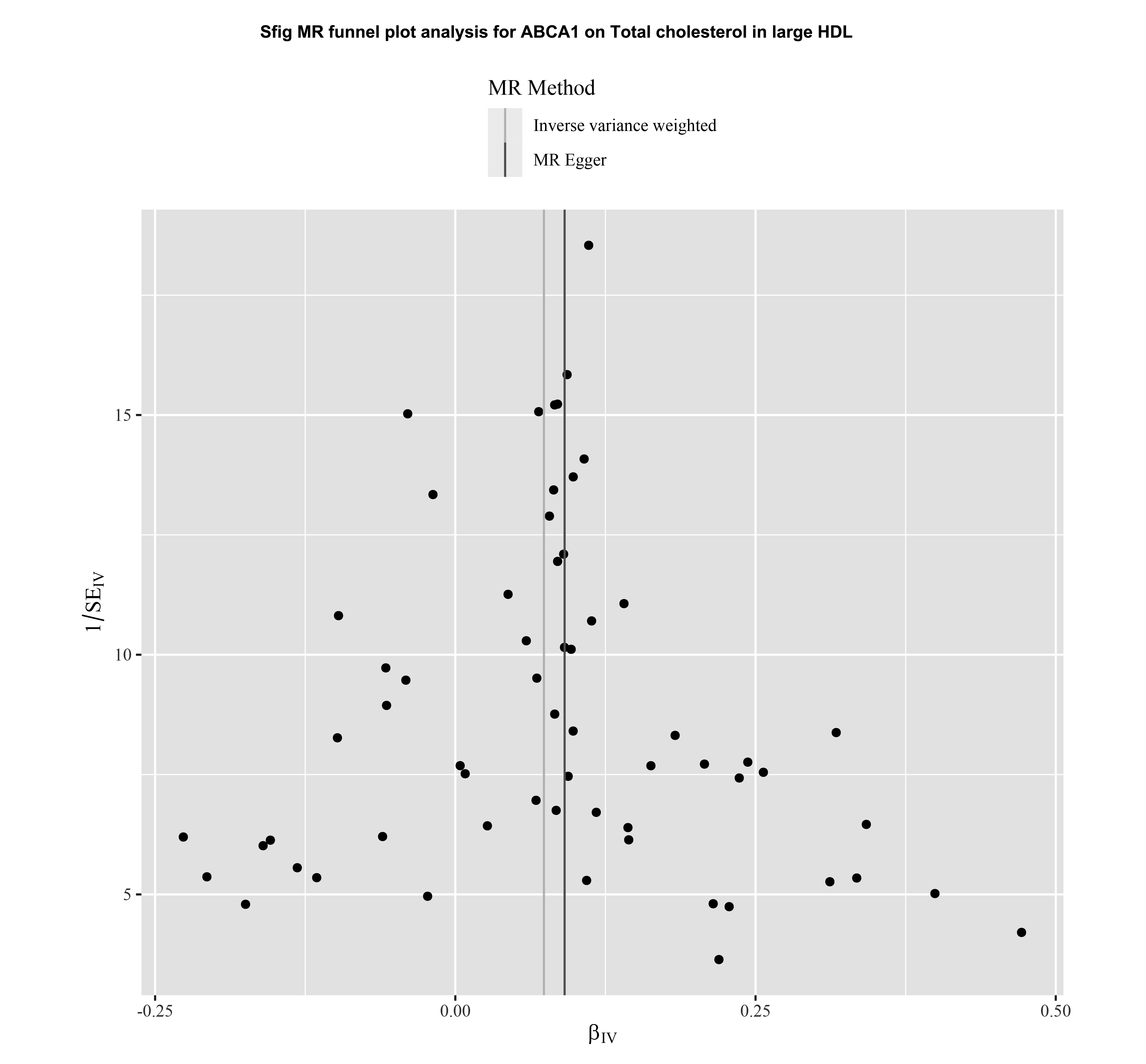

Supplement: Supplementary file 3 — Supplementary Information 3. [file 41598_2025_93644_MOESM3_ESM.zip › the funnel plot/Sfig MR funnel plot analysis for ABCA1 on Total cholesterol in large HDL.tif]

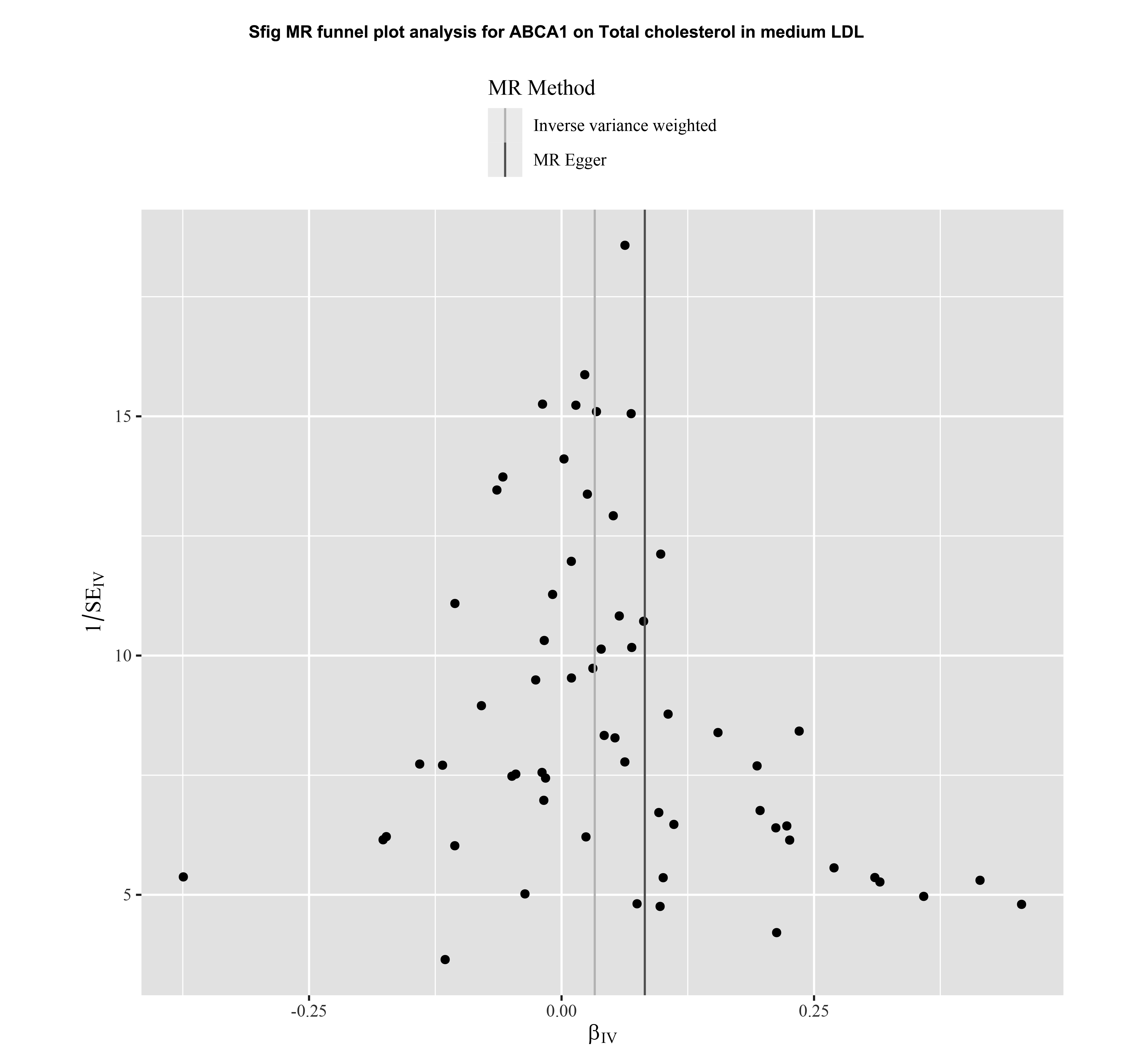

Supplement: Supplementary file 3 — Supplementary Information 3. [file 41598_2025_93644_MOESM3_ESM.zip › the funnel plot/Sfig MR funnel plot analysis for ABCA1 on Total cholesterol in medium LDL.tif]

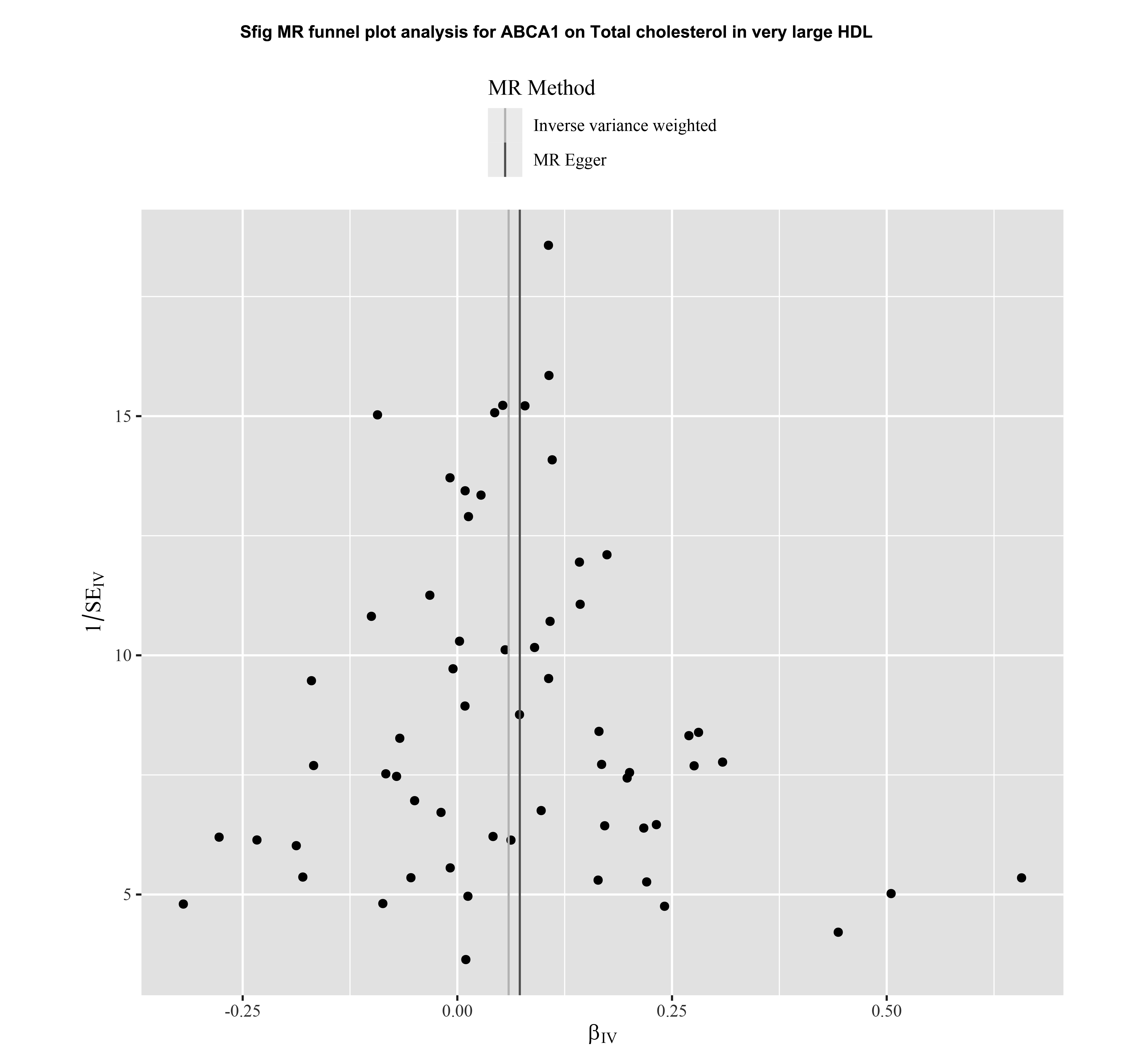

Supplement: Supplementary file 3 — Supplementary Information 3. [file 41598_2025_93644_MOESM3_ESM.zip › the funnel plot/Sfig MR funnel plot analysis for ABCA1 on Total cholesterol in very large HDL.tif]

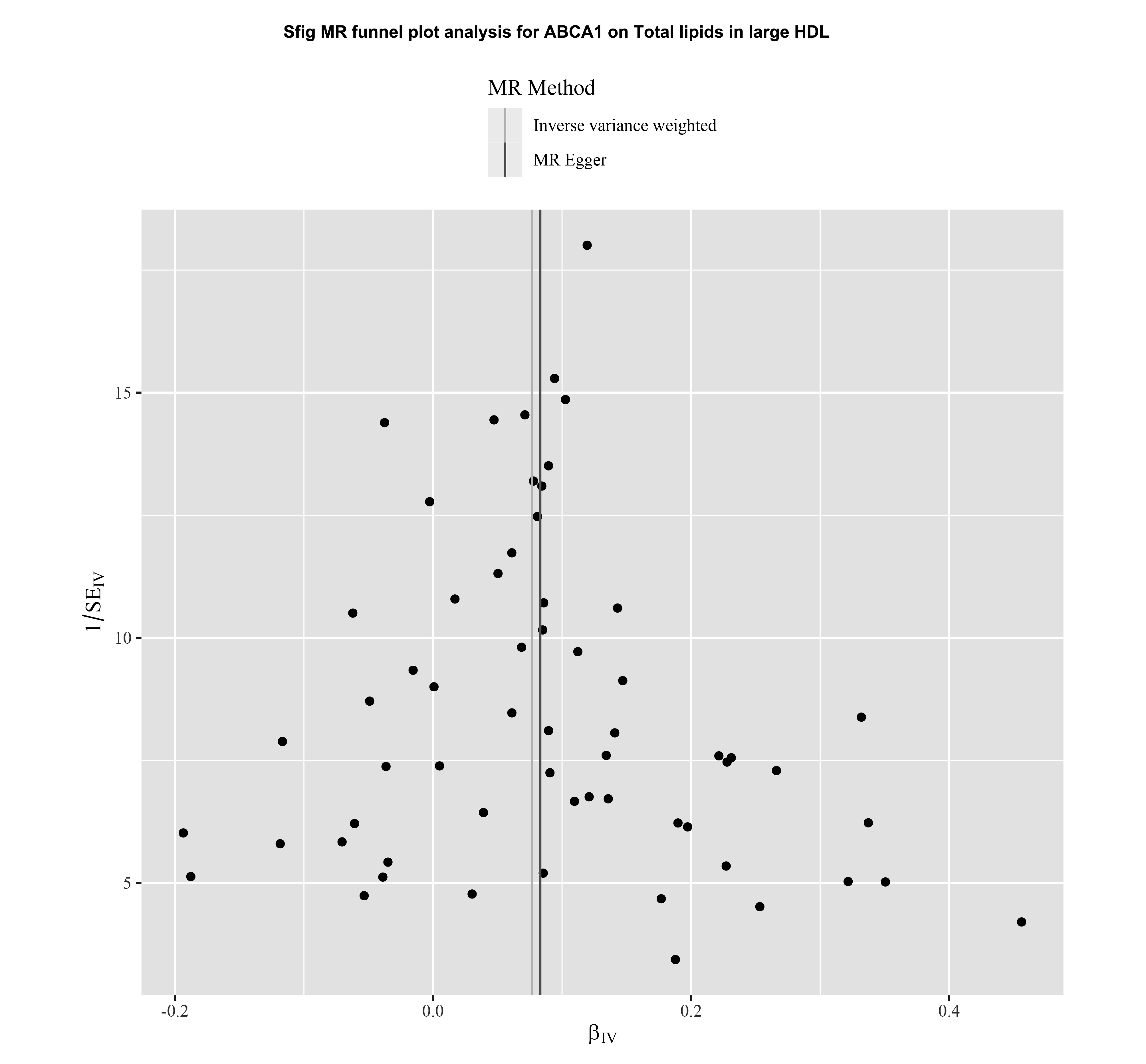

Supplement: Supplementary file 3 — Supplementary Information 3. [file 41598_2025_93644_MOESM3_ESM.zip › the funnel plot/Sfig MR funnel plot analysis for ABCA1 on Total lipids in large HDL.tif]

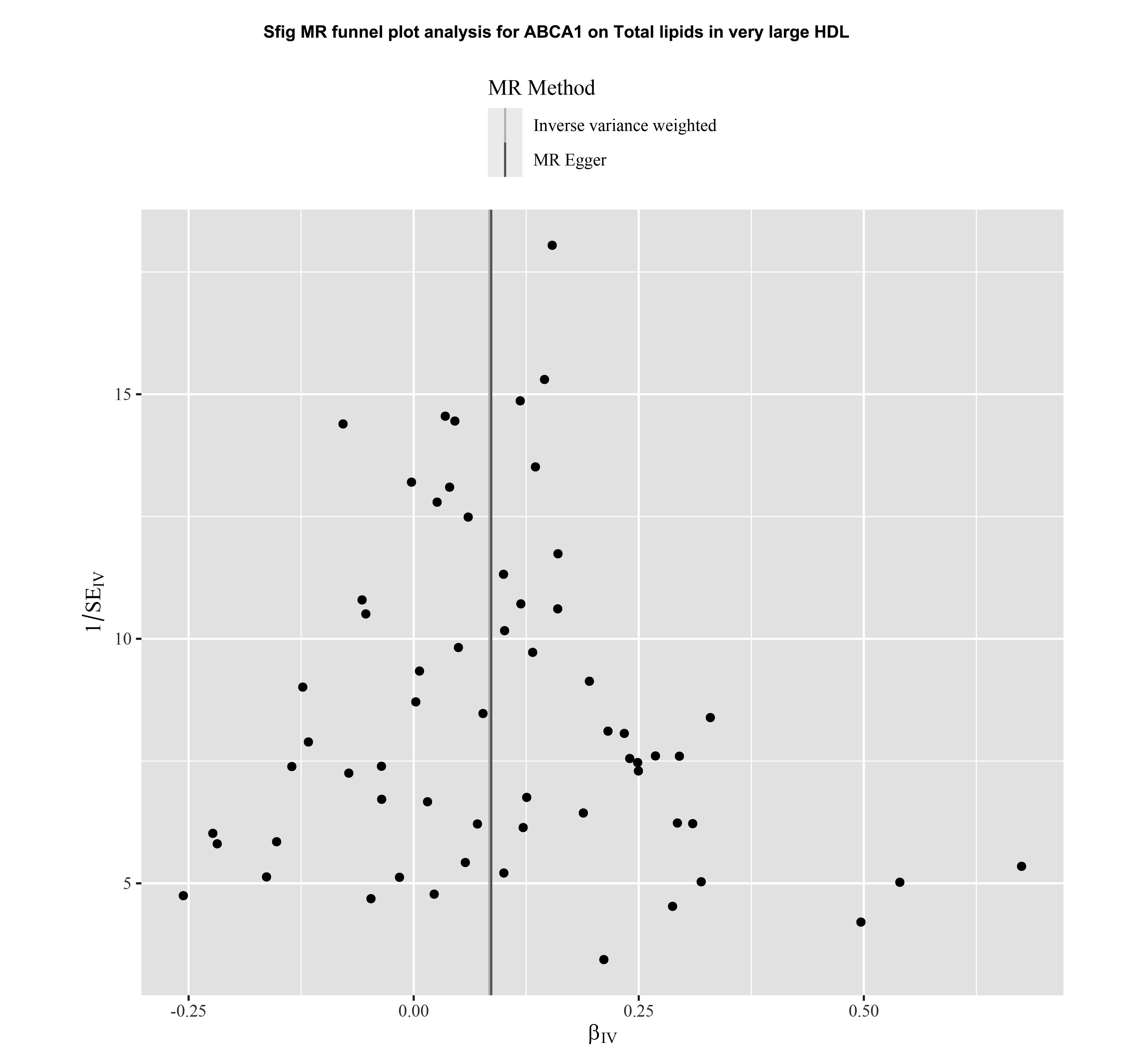

Supplement: Supplementary file 3 — Supplementary Information 3. [file 41598_2025_93644_MOESM3_ESM.zip › the funnel plot/Sfig MR funnel plot analysis for ABCA1 on Total lipids in very large HDL.tif]

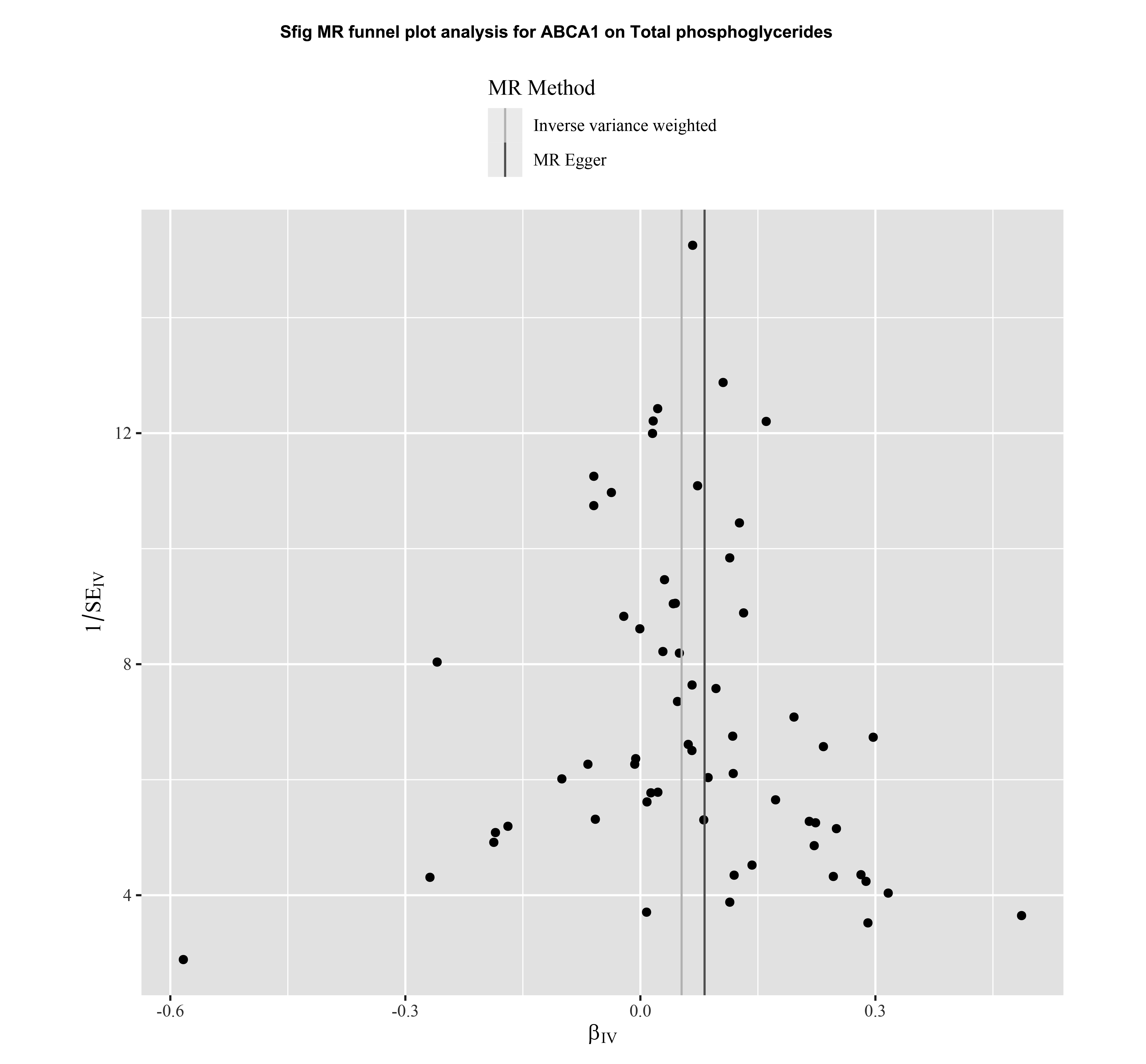

Supplement: Supplementary file 3 — Supplementary Information 3. [file 41598_2025_93644_MOESM3_ESM.zip › the funnel plot/Sfig MR funnel plot analysis for ABCA1 on Total phosphoglycerides.tif]

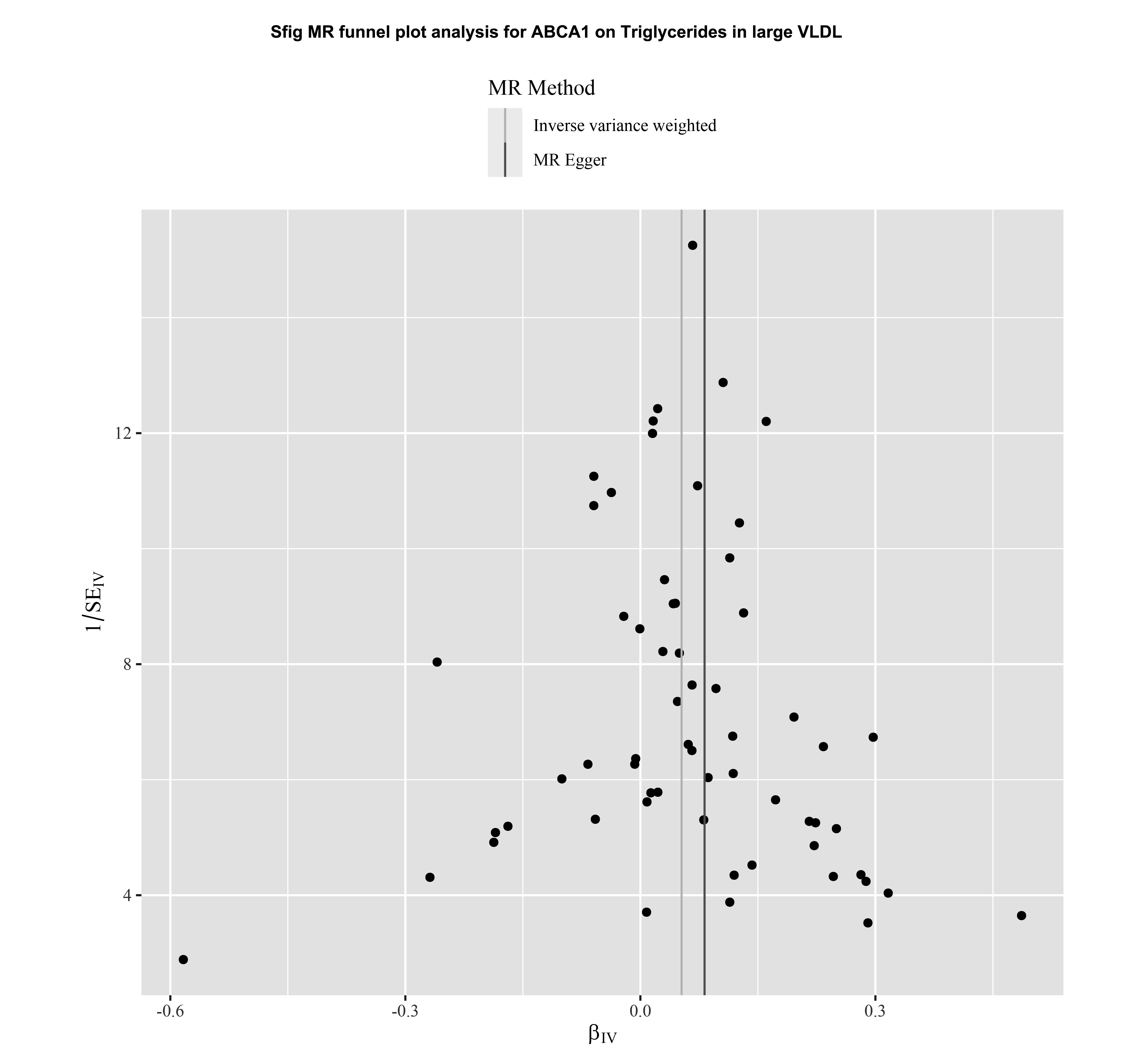

Supplement: Supplementary file 3 — Supplementary Information 3. [file 41598_2025_93644_MOESM3_ESM.zip › the funnel plot/Sfig MR funnel plot analysis for ABCA1 on Triglycerides in large VLDL.tif]

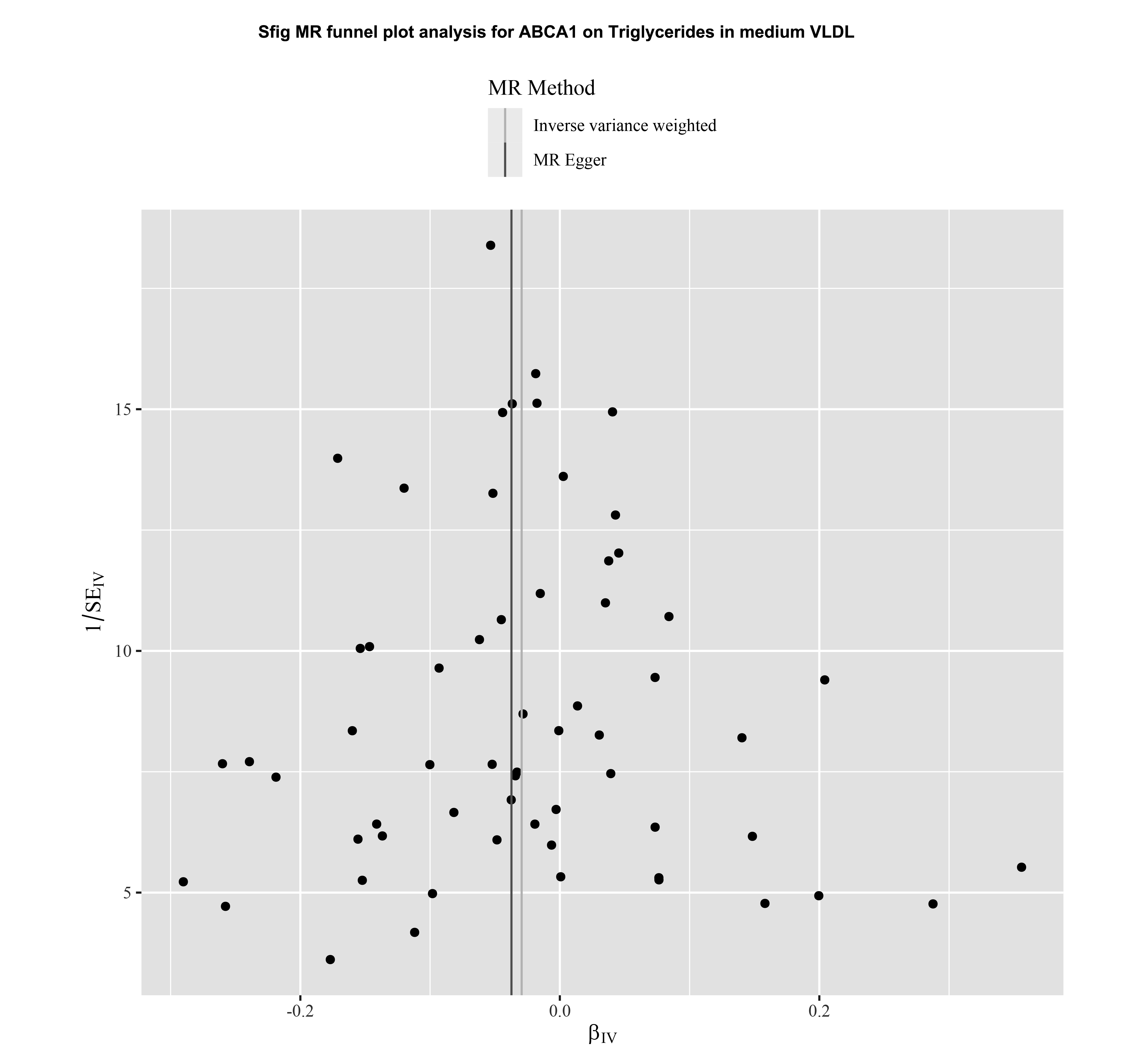

Supplement: Supplementary file 3 — Supplementary Information 3. [file 41598_2025_93644_MOESM3_ESM.zip › the funnel plot/Sfig MR funnel plot analysis for ABCA1 on Triglycerides in medium VLDL.tif]

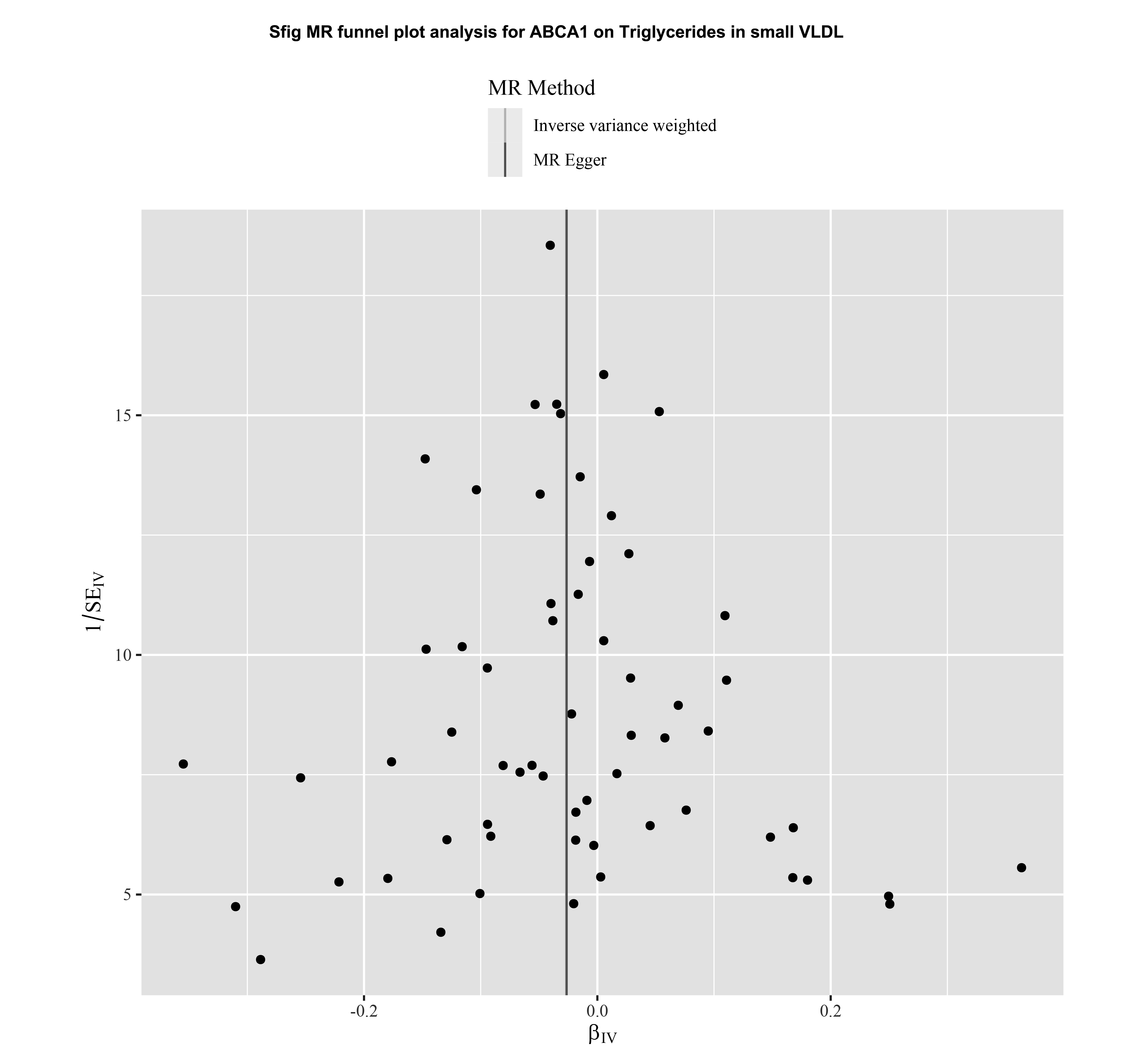

Supplement: Supplementary file 3 — Supplementary Information 3. [file 41598_2025_93644_MOESM3_ESM.zip › the funnel plot/Sfig MR funnel plot analysis for ABCA1 on Triglycerides in small VLDL.tif]

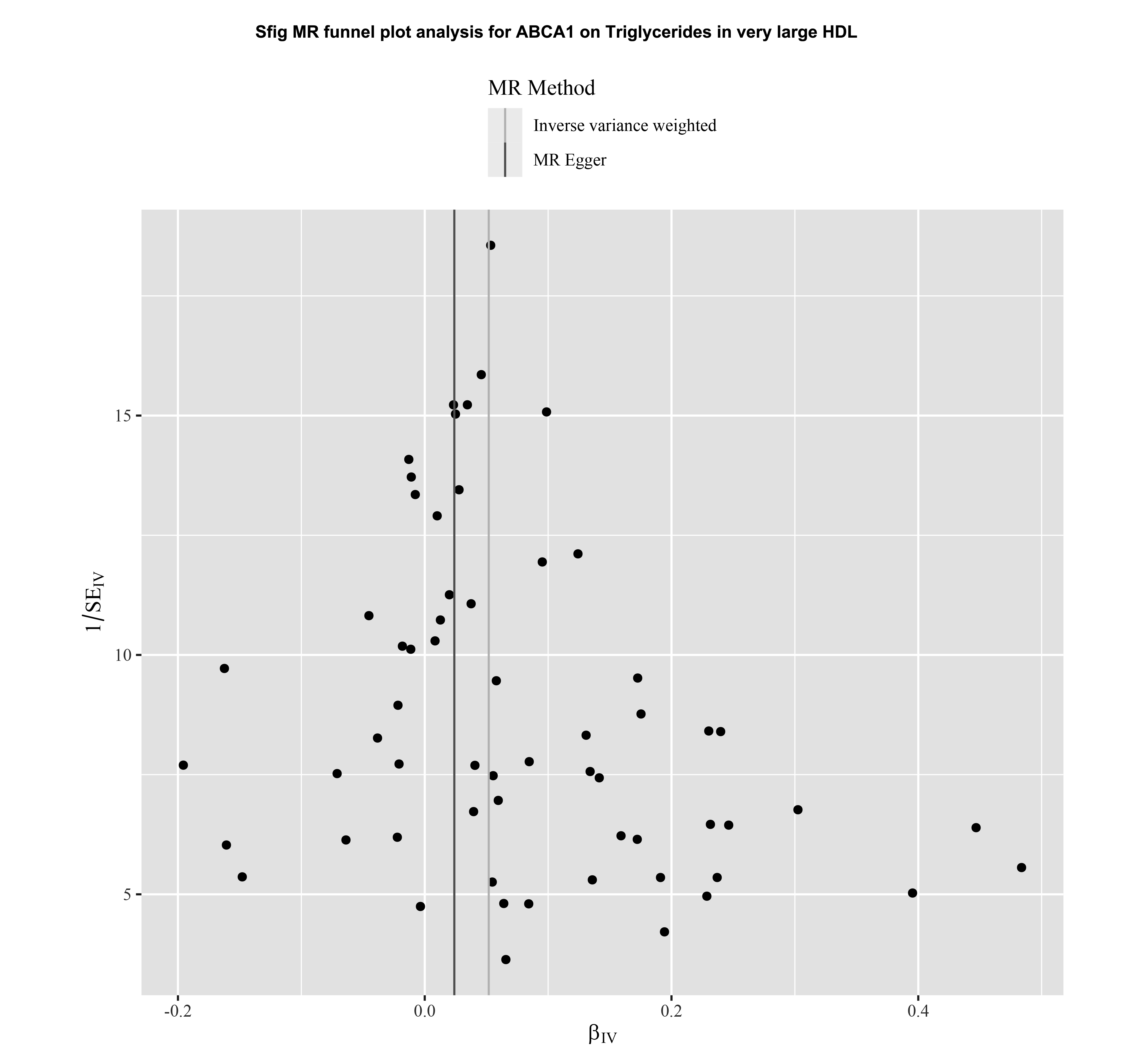

Supplement: Supplementary file 3 — Supplementary Information 3. [file 41598_2025_93644_MOESM3_ESM.zip › the funnel plot/Sfig MR funnel plot analysis for ABCA1 on Triglycerides in very large HDL.tif]

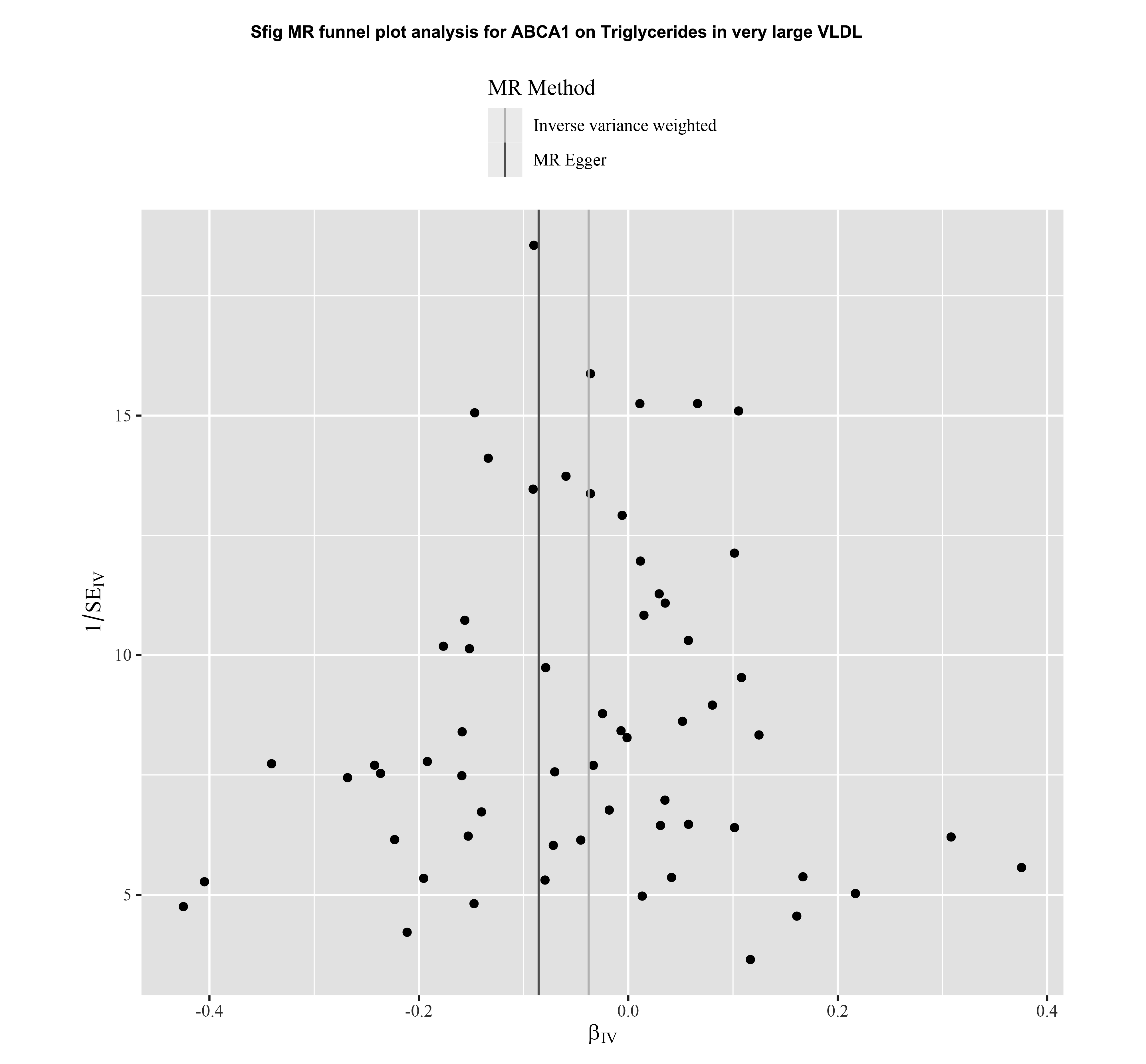

Supplement: Supplementary file 3 — Supplementary Information 3. [file 41598_2025_93644_MOESM3_ESM.zip › the funnel plot/Sfig MR funnel plot analysis for ABCA1 on Triglycerides in very large VLDL.tif]

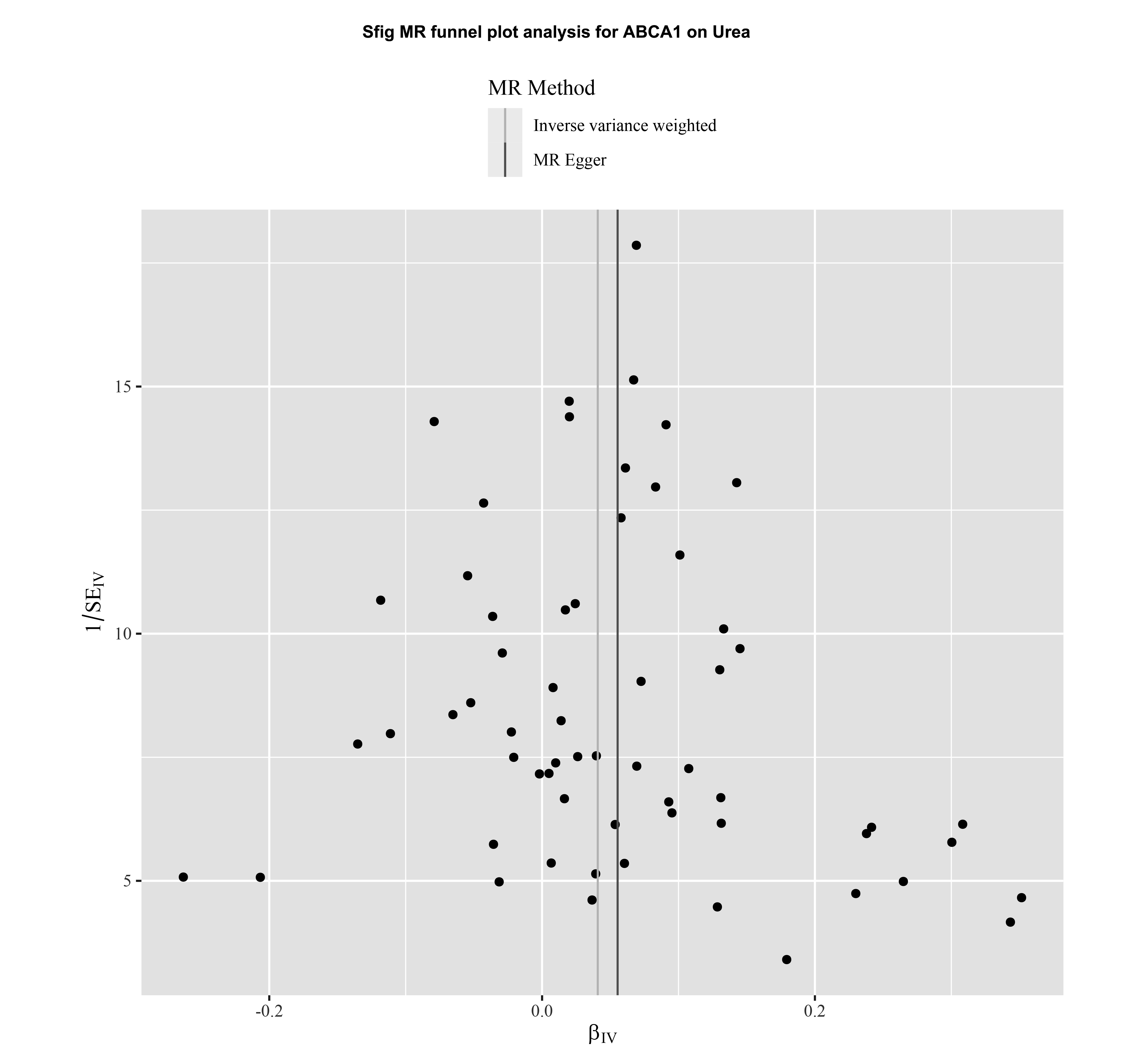

Supplement: Supplementary file 3 — Supplementary Information 3. [file 41598_2025_93644_MOESM3_ESM.zip › the funnel plot/Sfig MR funnel plot analysis for ABCA1 on Urea.tif]

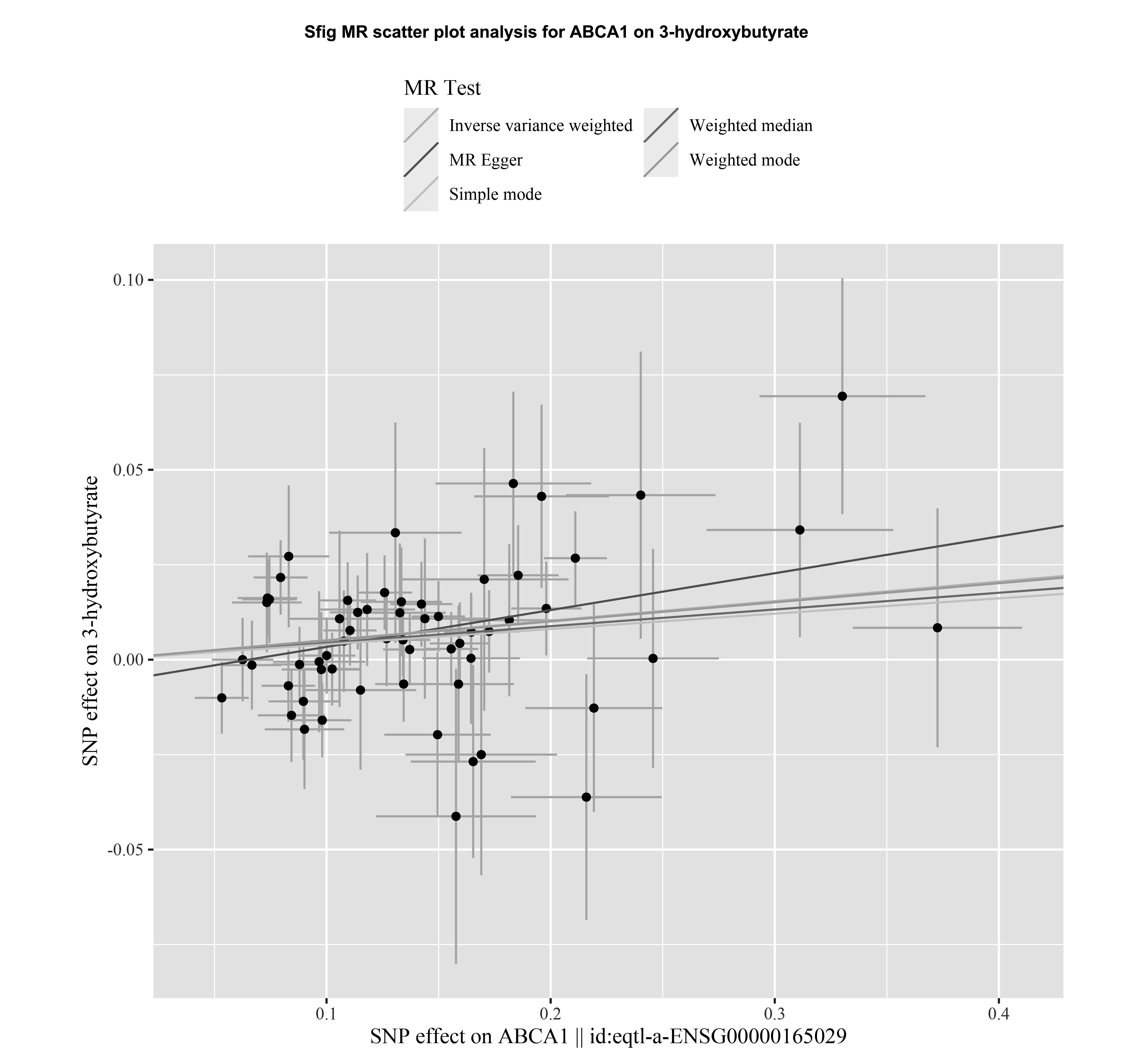

Supplement: Supplementary file 3 — Supplementary Information 3. [file 41598_2025_93644_MOESM3_ESM.zip › the scatter plot/Sfig MR scatter plot analysis for ABCA1 on 3-hydroxybutyrate.tif]

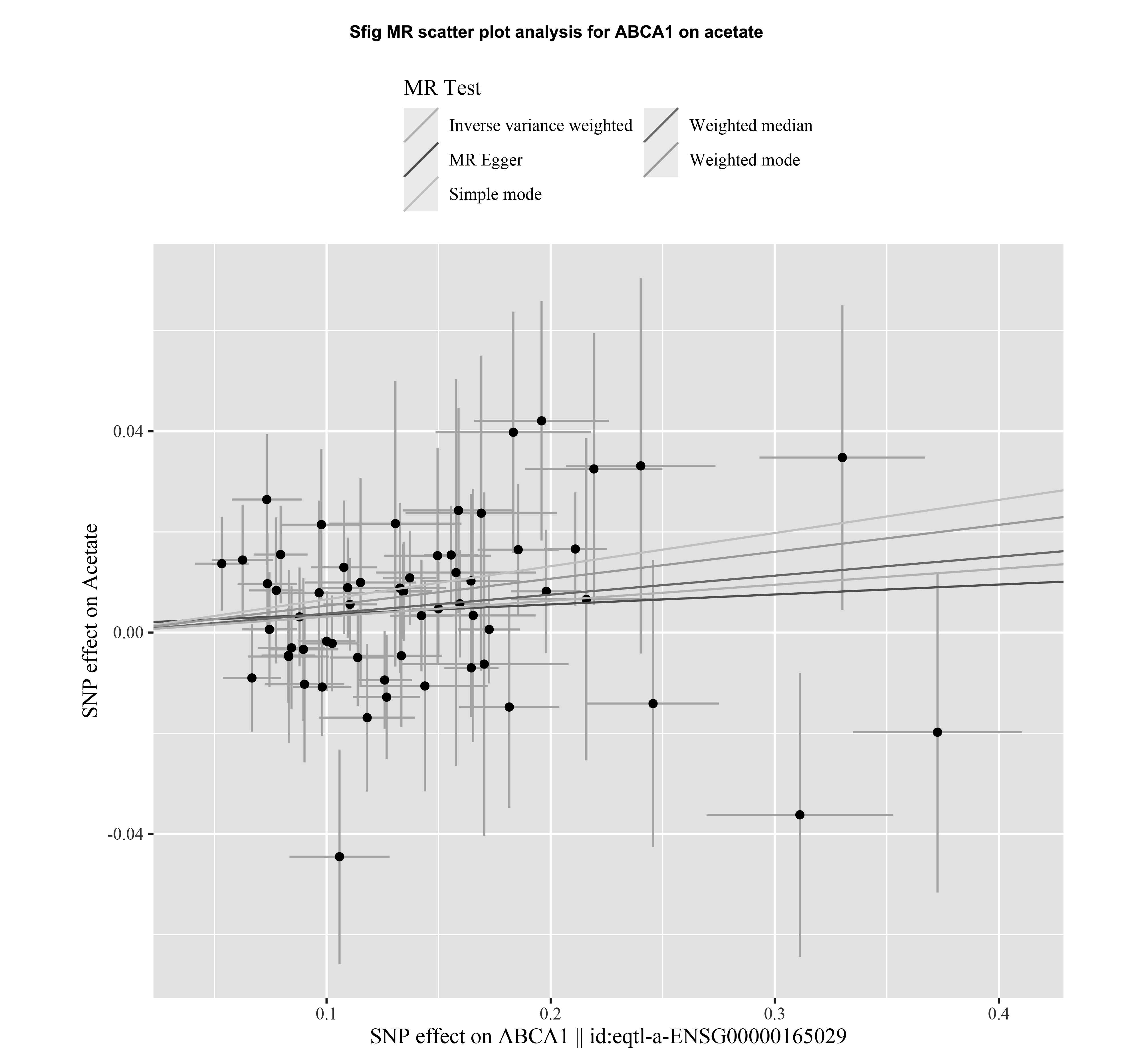

Supplement: Supplementary file 3 — Supplementary Information 3. [file 41598_2025_93644_MOESM3_ESM.zip › the scatter plot/Sfig MR scatter plot analysis for ABCA1 on acetate.tif]

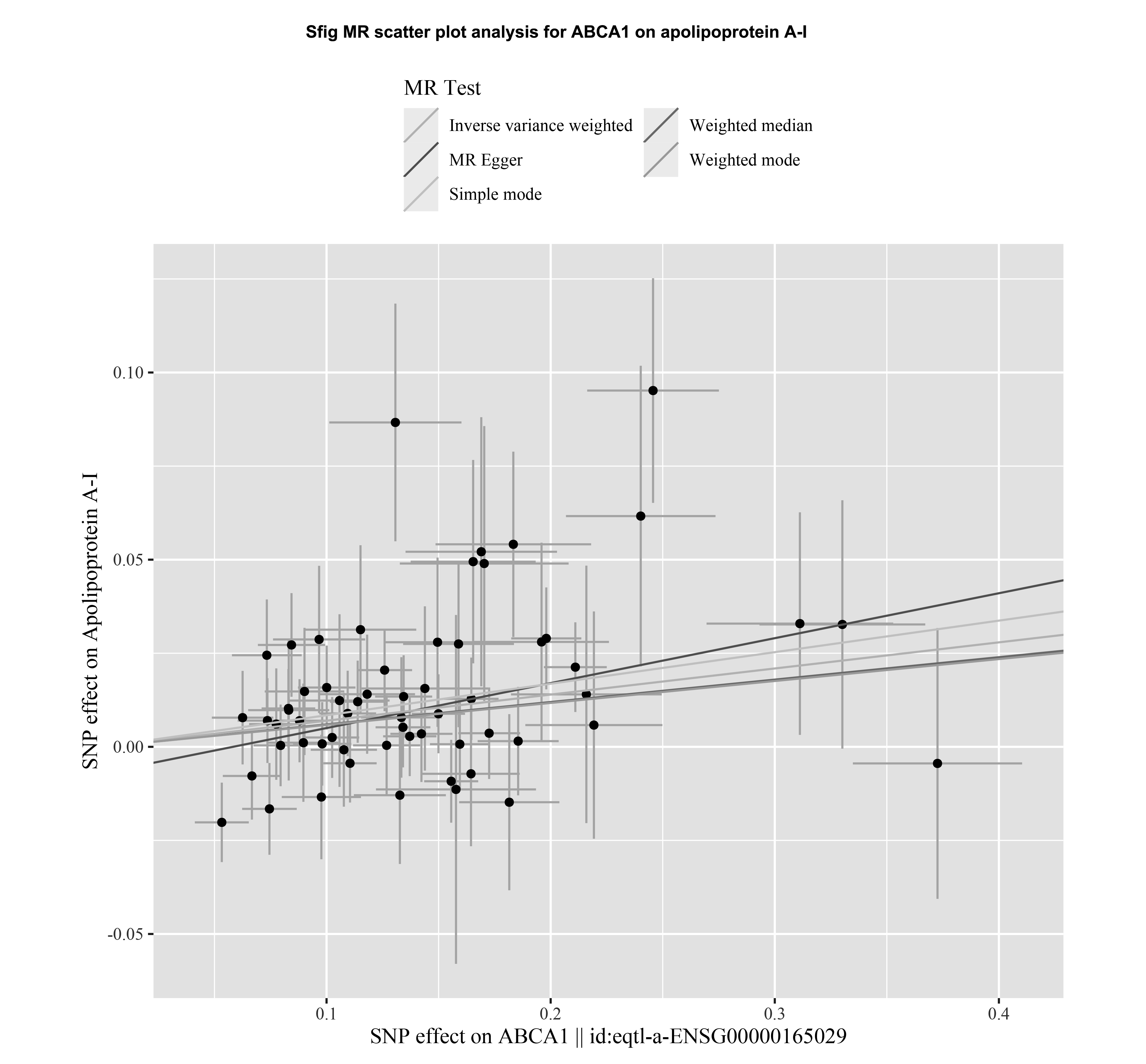

Supplement: Supplementary file 3 — Supplementary Information 3. [file 41598_2025_93644_MOESM3_ESM.zip › the scatter plot/Sfig MR scatter plot analysis for ABCA1 on apolipoprotein A-I.tif]

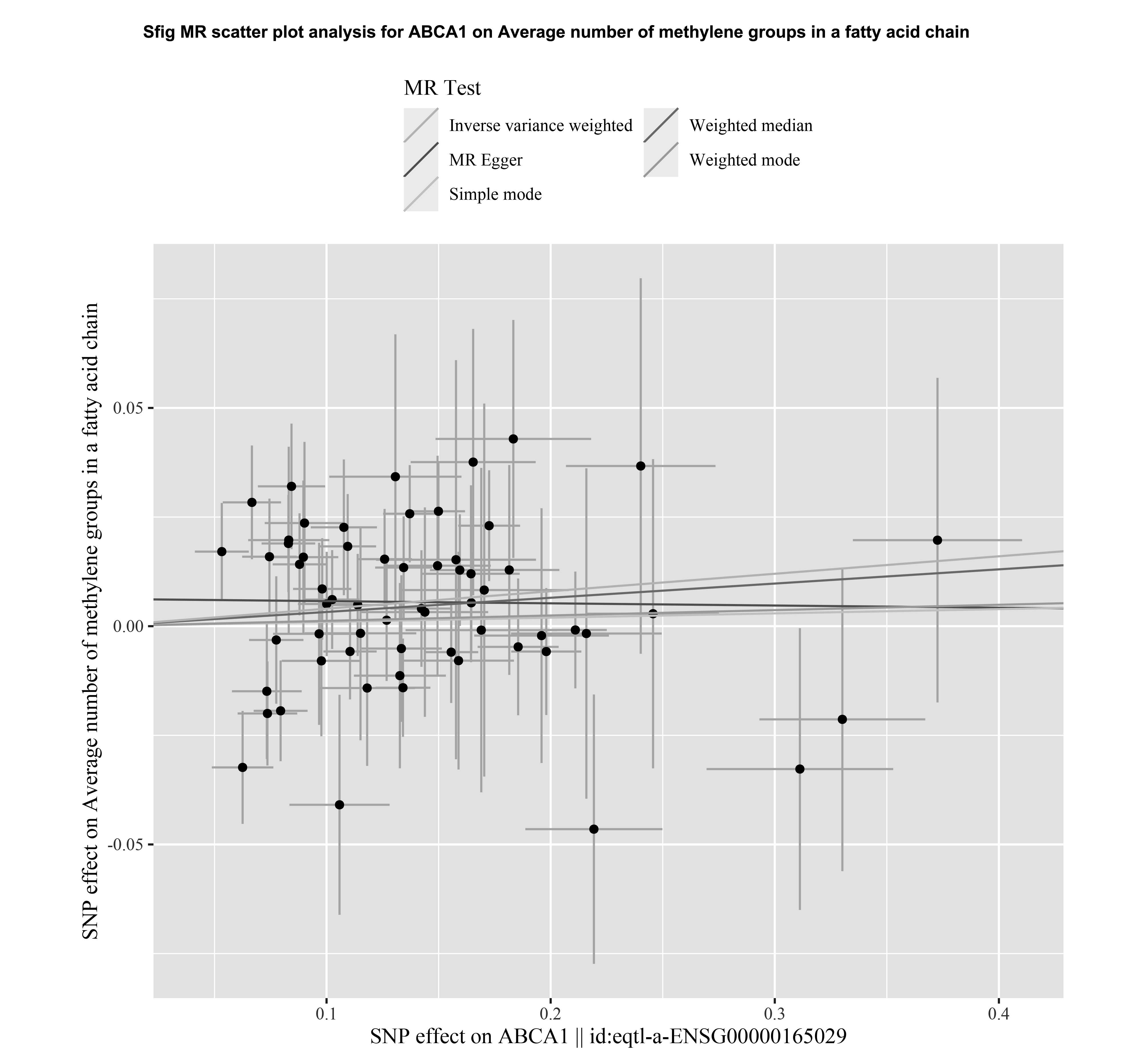

Supplement: Supplementary file 3 — Supplementary Information 3. [file 41598_2025_93644_MOESM3_ESM.zip › the scatter plot/Sfig MR scatter plot analysis for ABCA1 on Average number of methylene groups in a fatty acid chain.tif]

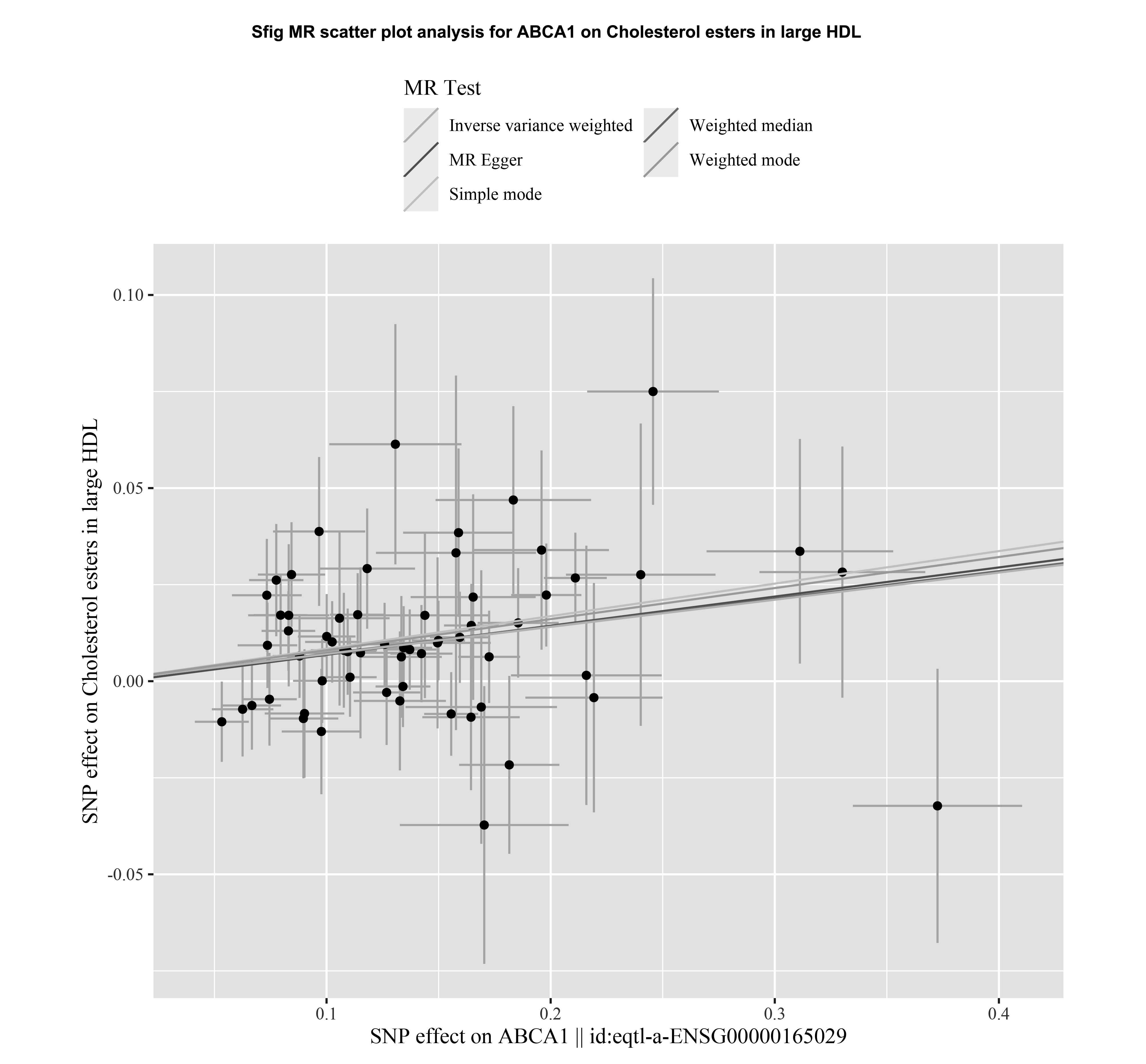

Supplement: Supplementary file 3 — Supplementary Information 3. [file 41598_2025_93644_MOESM3_ESM.zip › the scatter plot/Sfig MR scatter plot analysis for ABCA1 on Cholesterol esters in large HDL.tif]

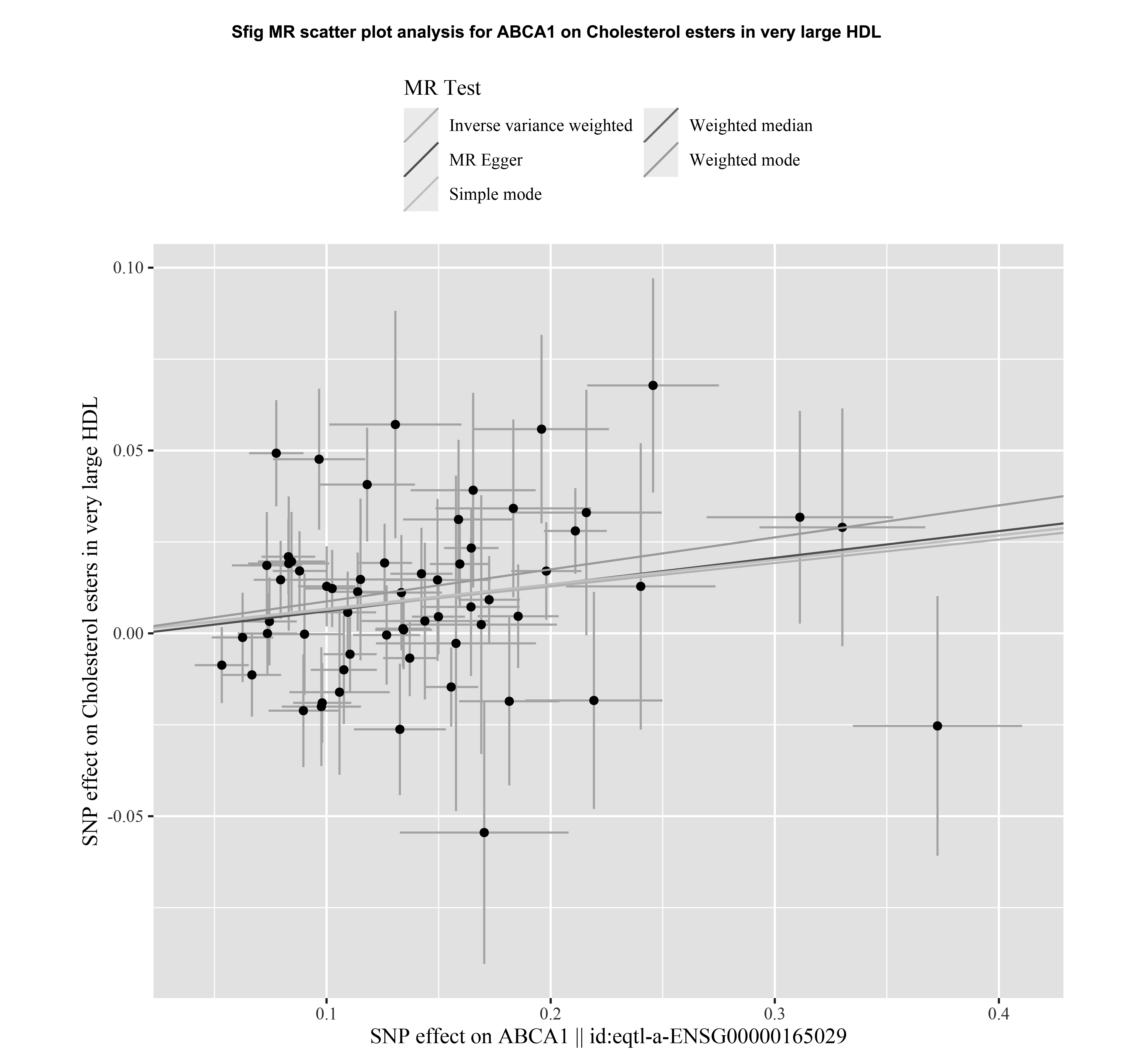

Supplement: Supplementary file 3 — Supplementary Information 3. [file 41598_2025_93644_MOESM3_ESM.zip › the scatter plot/Sfig MR scatter plot analysis for ABCA1 on Cholesterol esters in very large HDL.tif]

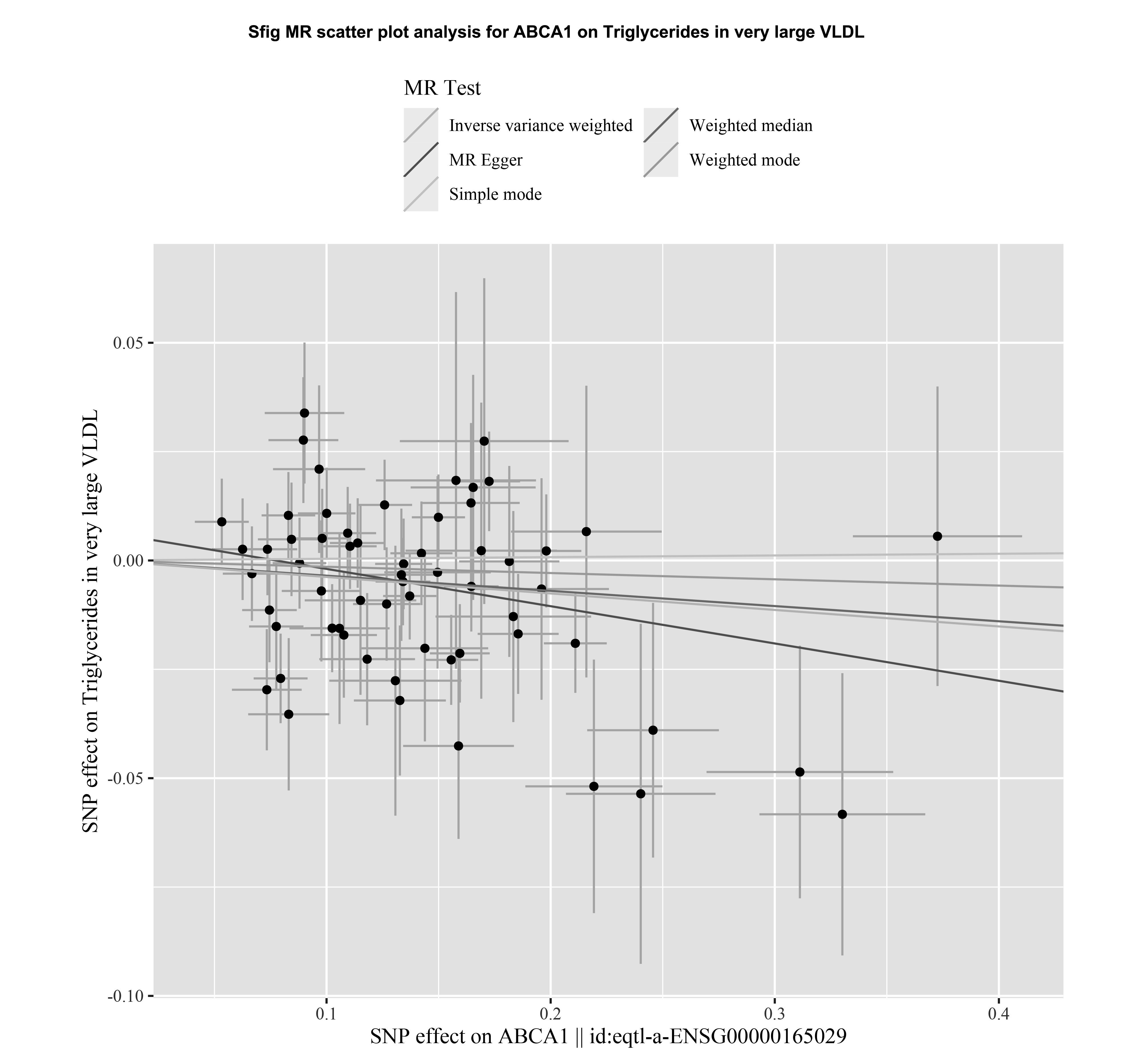

Supplement: Supplementary file 3 — Supplementary Information 3. [file 41598_2025_93644_MOESM3_ESM.zip › the scatter plot/Sfig MR scatter plot analysis for ABCA1 on Concentration of large HDL particles.tif]

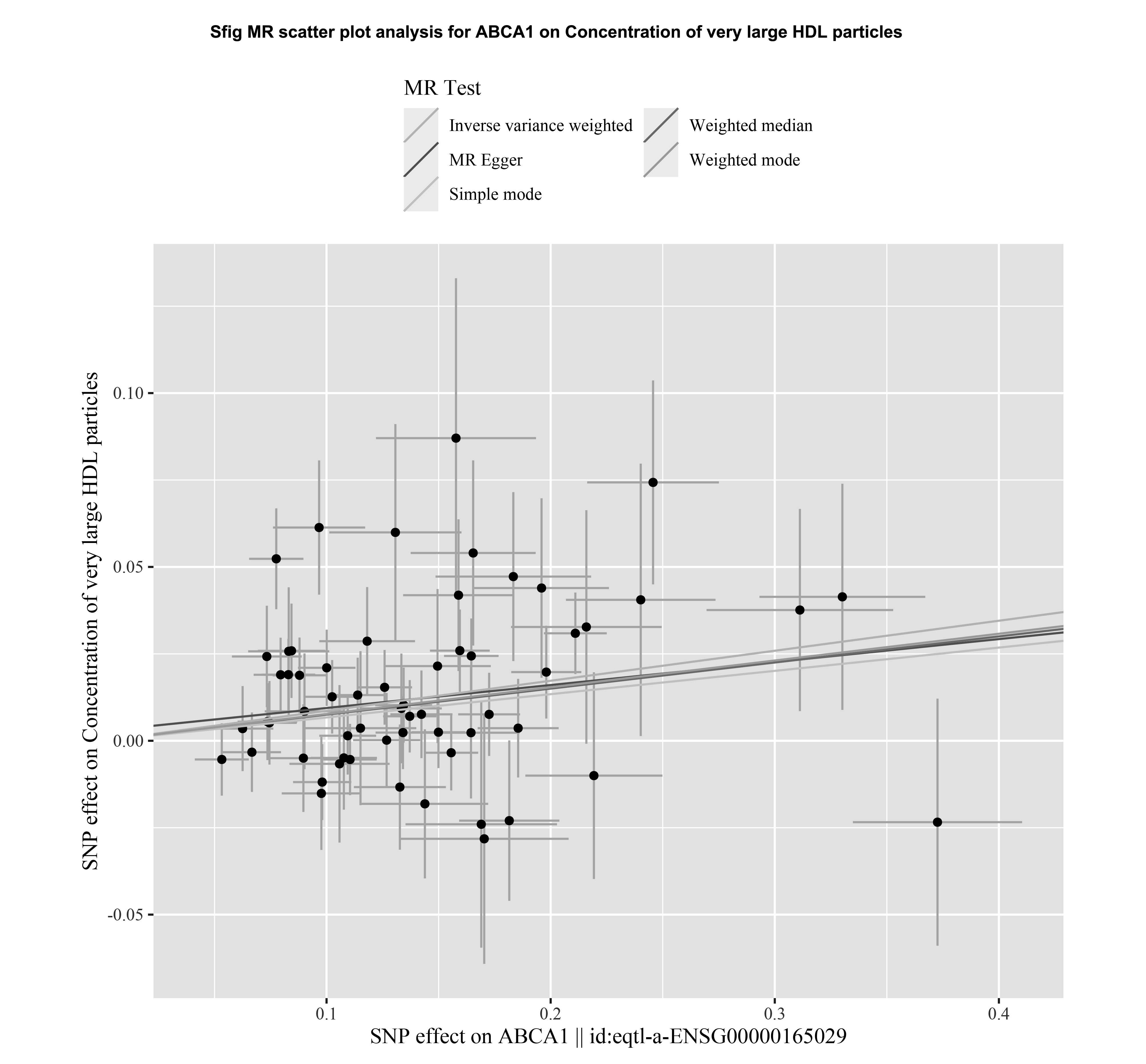

Supplement: Supplementary file 3 — Supplementary Information 3. [file 41598_2025_93644_MOESM3_ESM.zip › the scatter plot/Sfig MR scatter plot analysis for ABCA1 on Concentration of very large HDL particles.tif]

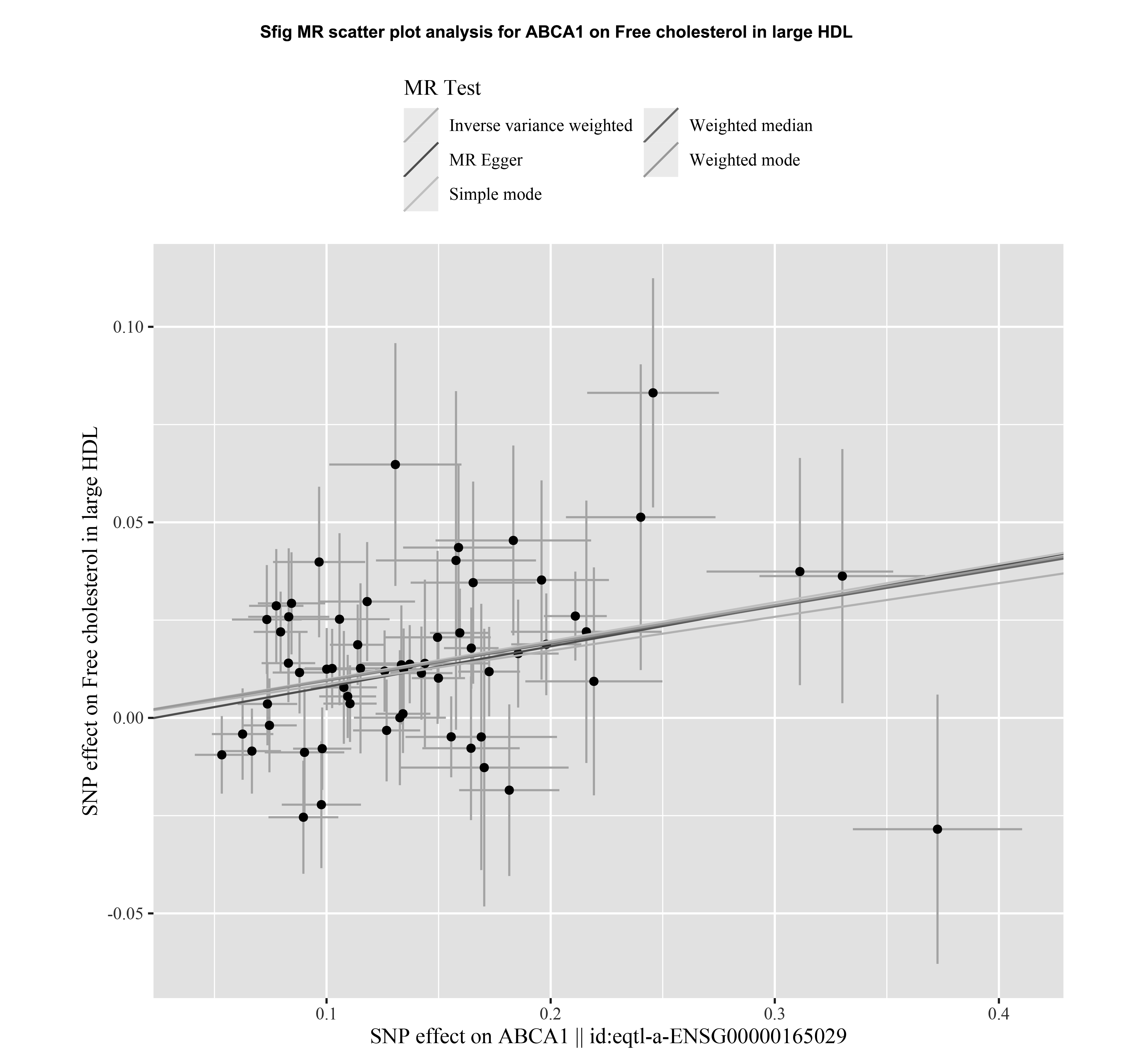

Supplement: Supplementary file 3 — Supplementary Information 3. [file 41598_2025_93644_MOESM3_ESM.zip › the scatter plot/Sfig MR scatter plot analysis for ABCA1 on Free cholesterol in large HDL.tif]

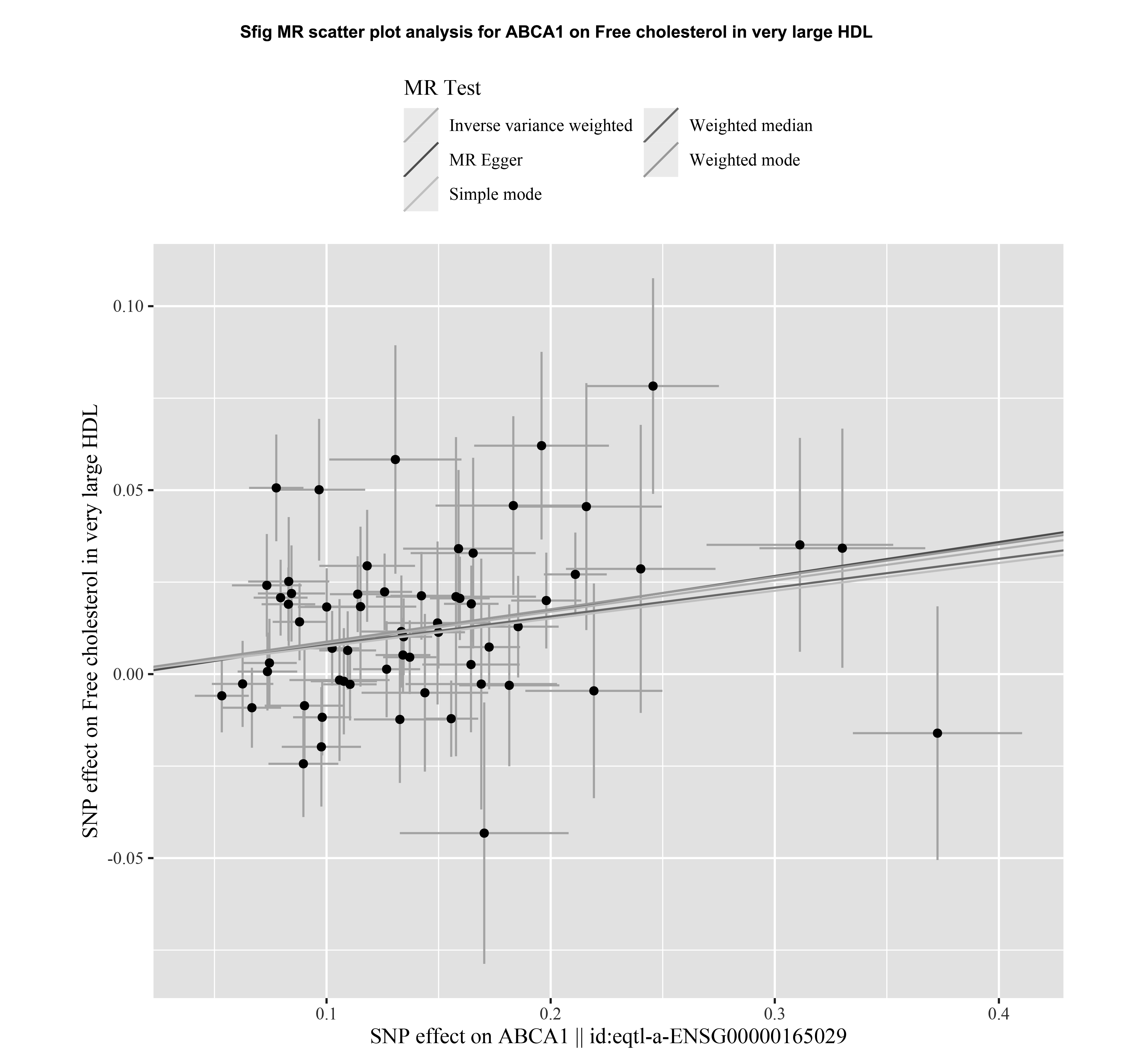

Supplement: Supplementary file 3 — Supplementary Information 3. [file 41598_2025_93644_MOESM3_ESM.zip › the scatter plot/Sfig MR scatter plot analysis for ABCA1 on Free cholesterol in very large HDL.tif]

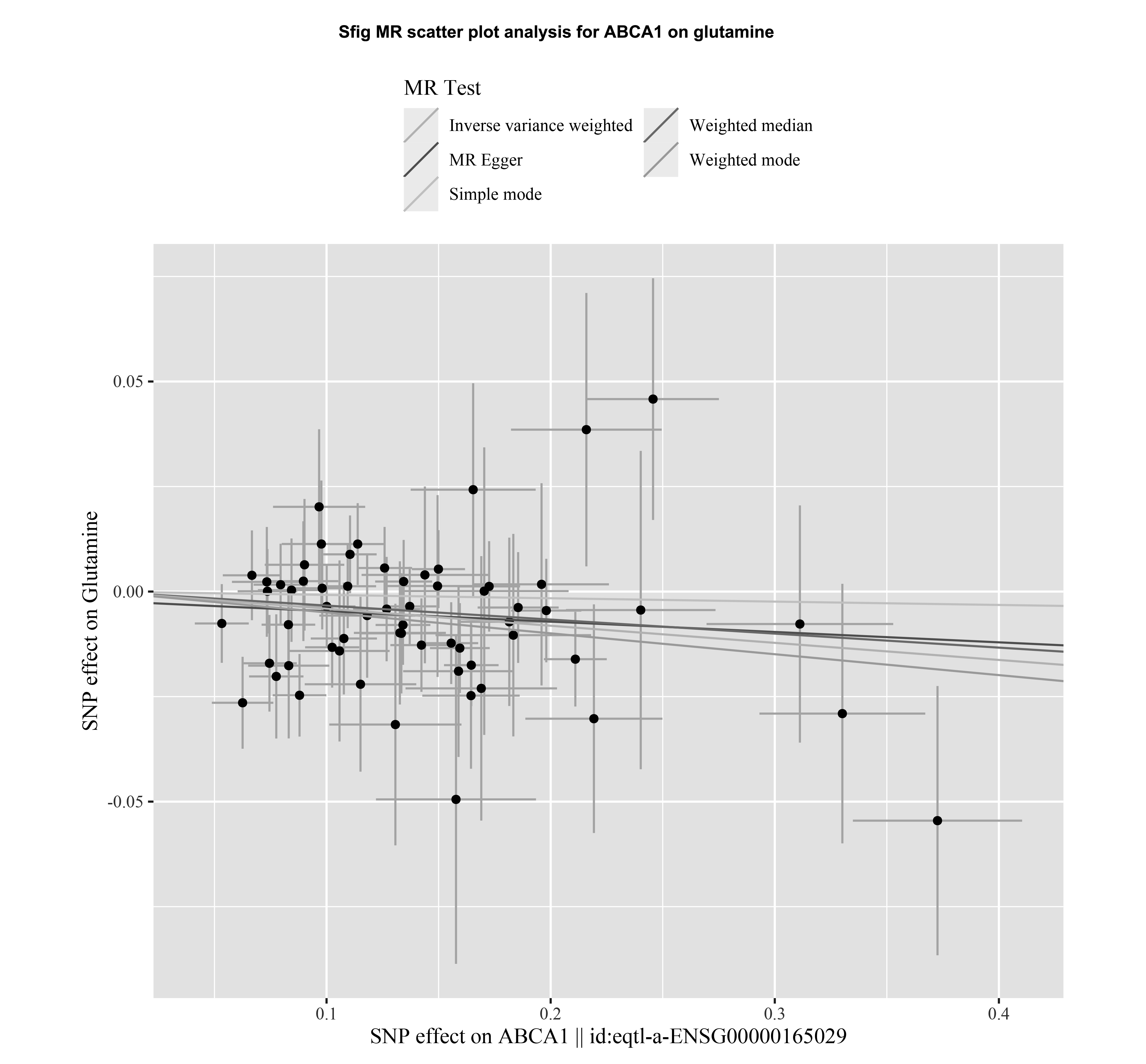

Supplement: Supplementary file 3 — Supplementary Information 3. [file 41598_2025_93644_MOESM3_ESM.zip › the scatter plot/Sfig MR scatter plot analysis for ABCA1 on glutamine.tif]

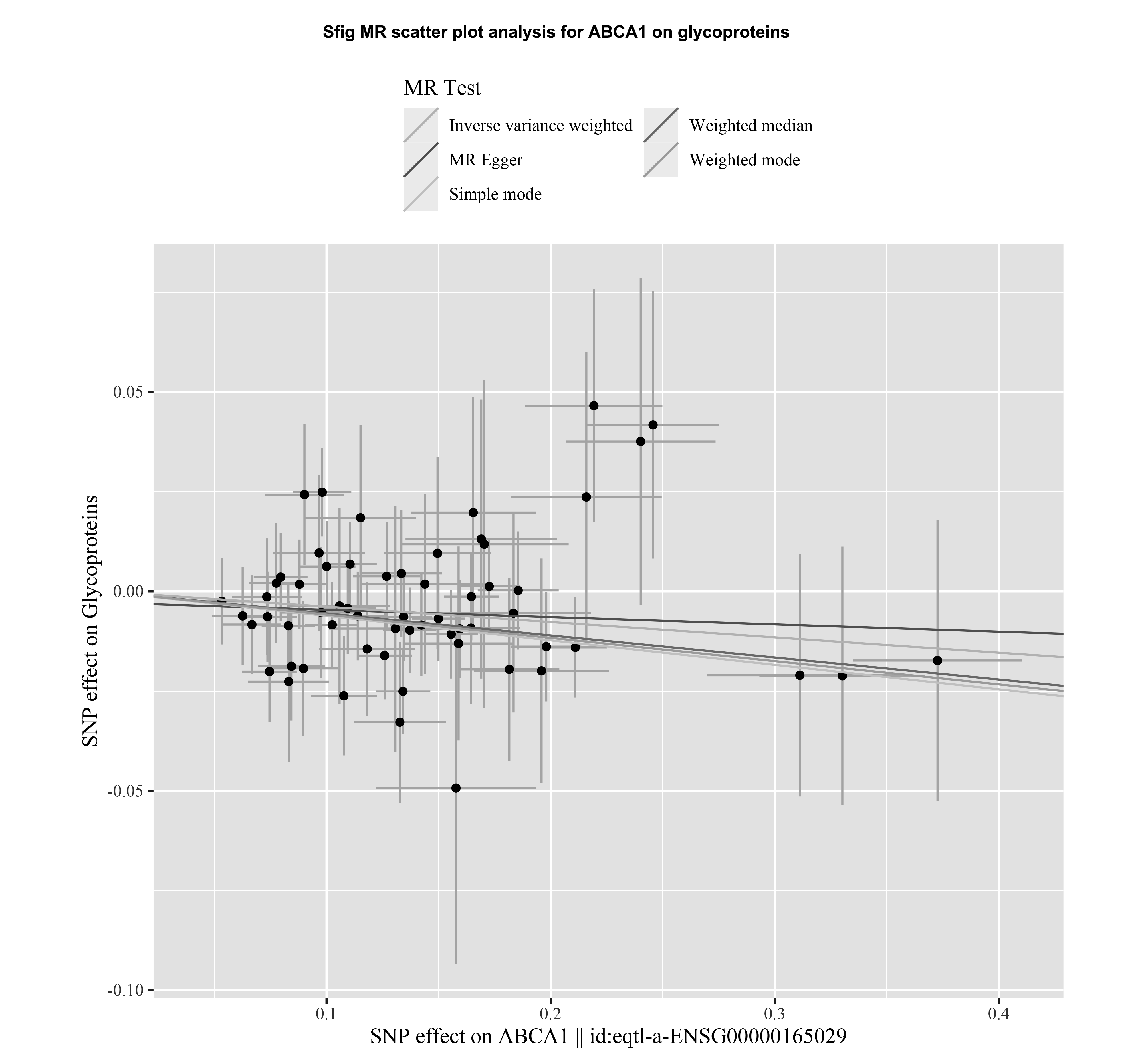

Supplement: Supplementary file 3 — Supplementary Information 3. [file 41598_2025_93644_MOESM3_ESM.zip › the scatter plot/Sfig MR scatter plot analysis for ABCA1 on glycoproteins.tif]

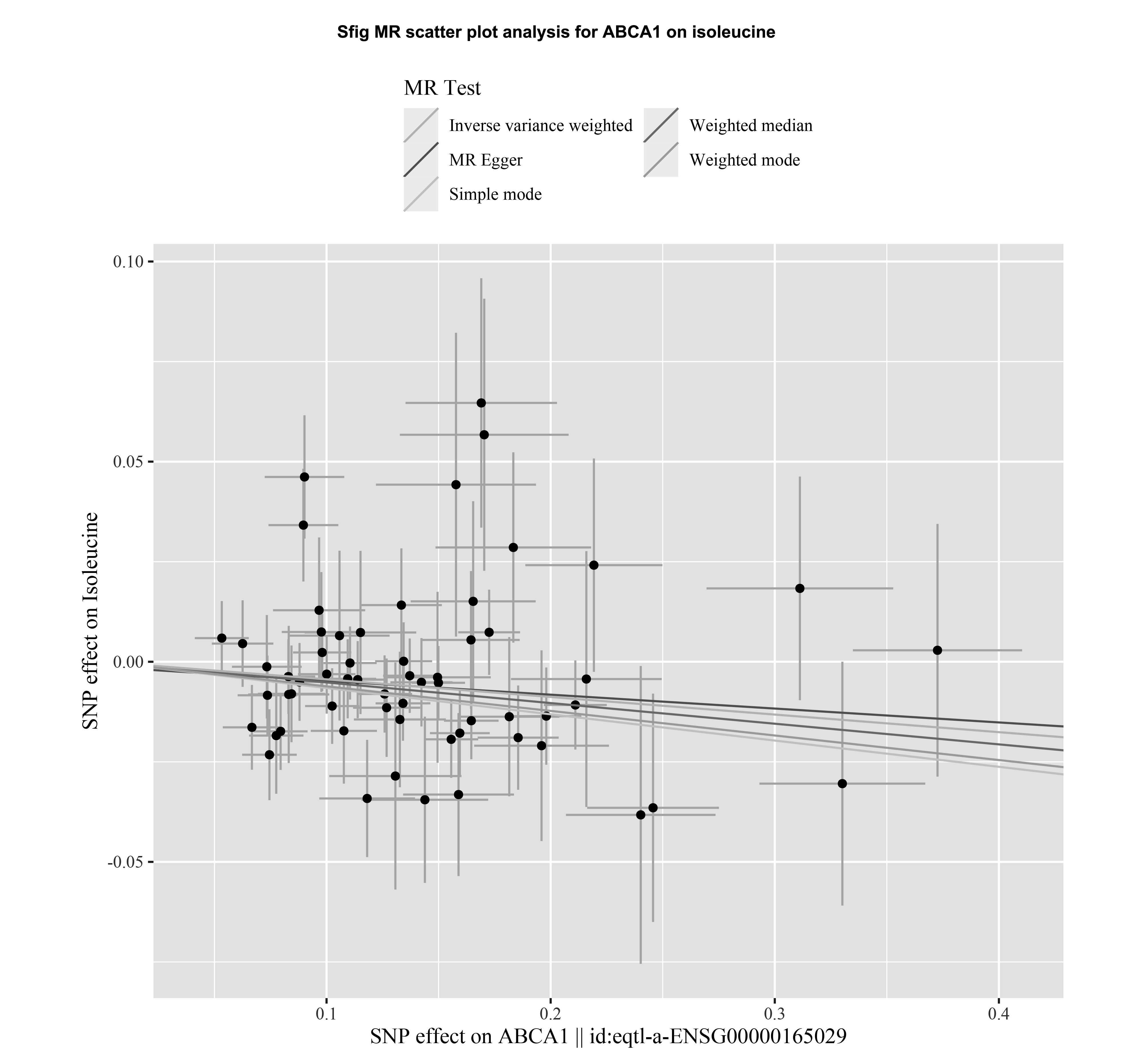

Supplement: Supplementary file 3 — Supplementary Information 3. [file 41598_2025_93644_MOESM3_ESM.zip › the scatter plot/Sfig MR scatter plot analysis for ABCA1 on isoleucine.tif]

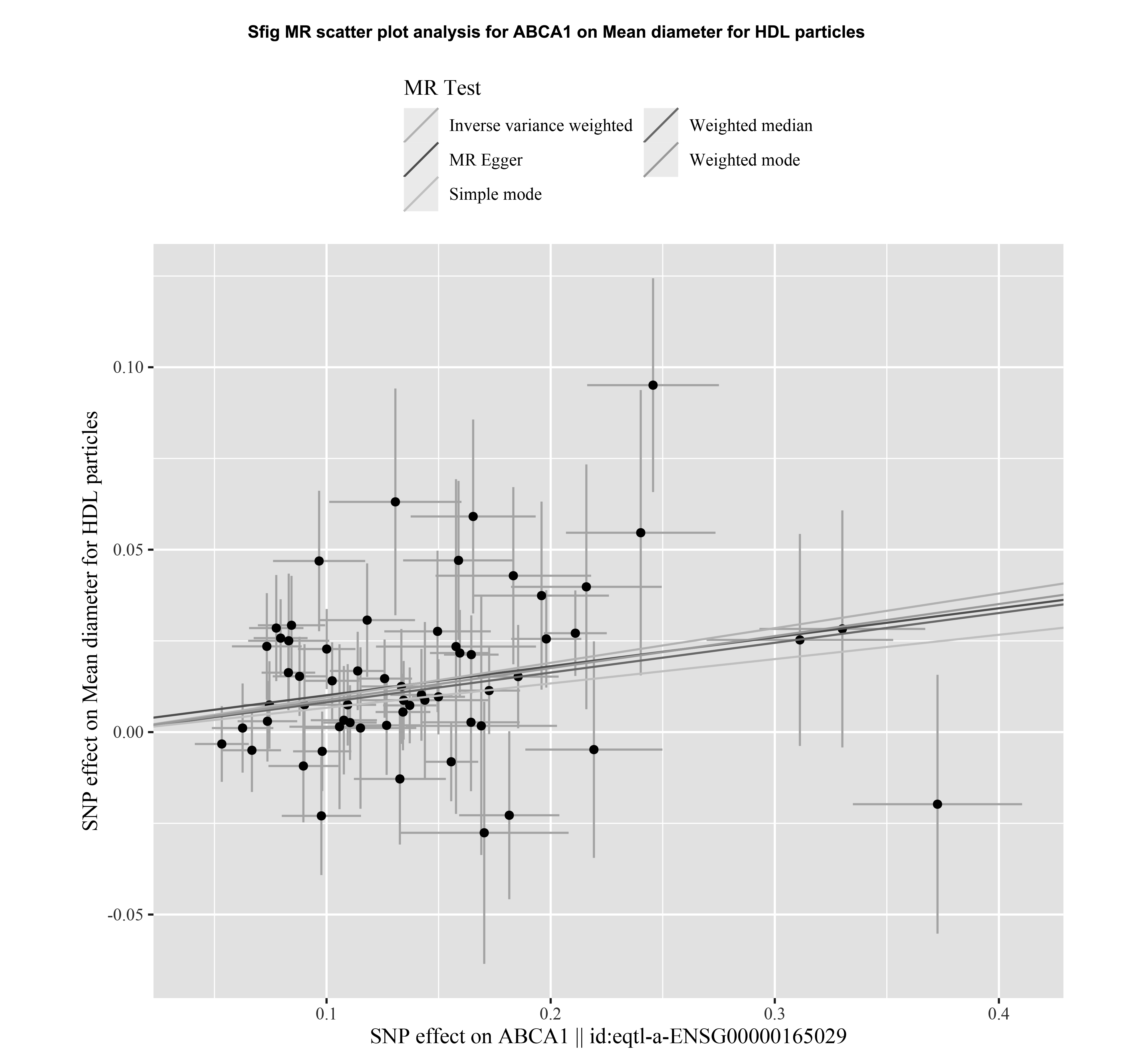

Supplement: Supplementary file 3 — Supplementary Information 3. [file 41598_2025_93644_MOESM3_ESM.zip › the scatter plot/Sfig MR scatter plot analysis for ABCA1 on Mean diameter for HDL particles.tif]

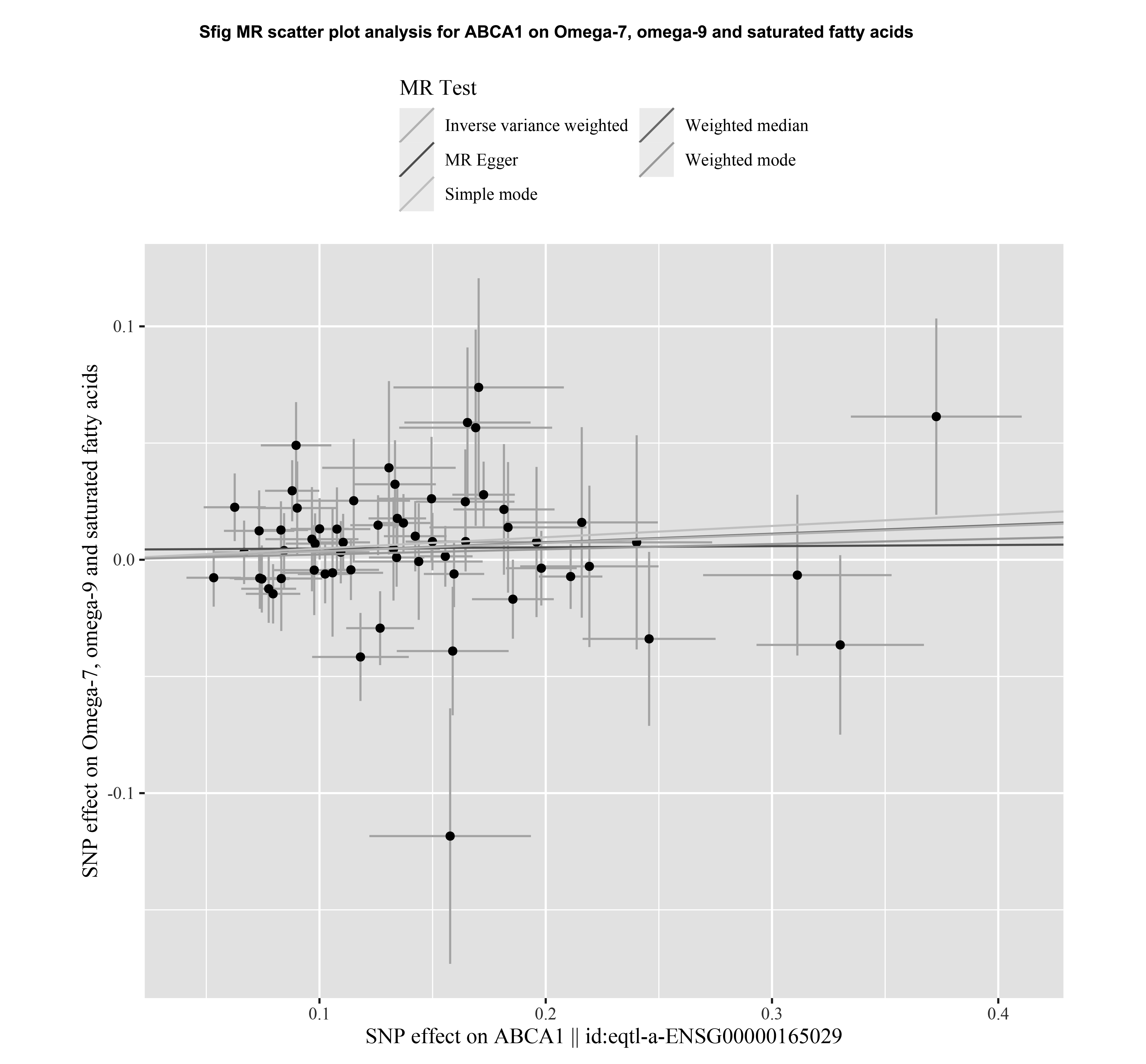

Supplement: Supplementary file 3 — Supplementary Information 3. [file 41598_2025_93644_MOESM3_ESM.zip › the scatter plot/Sfig MR scatter plot analysis for ABCA1 on Omega-7, omega-9 and saturated fatty acids.tif]

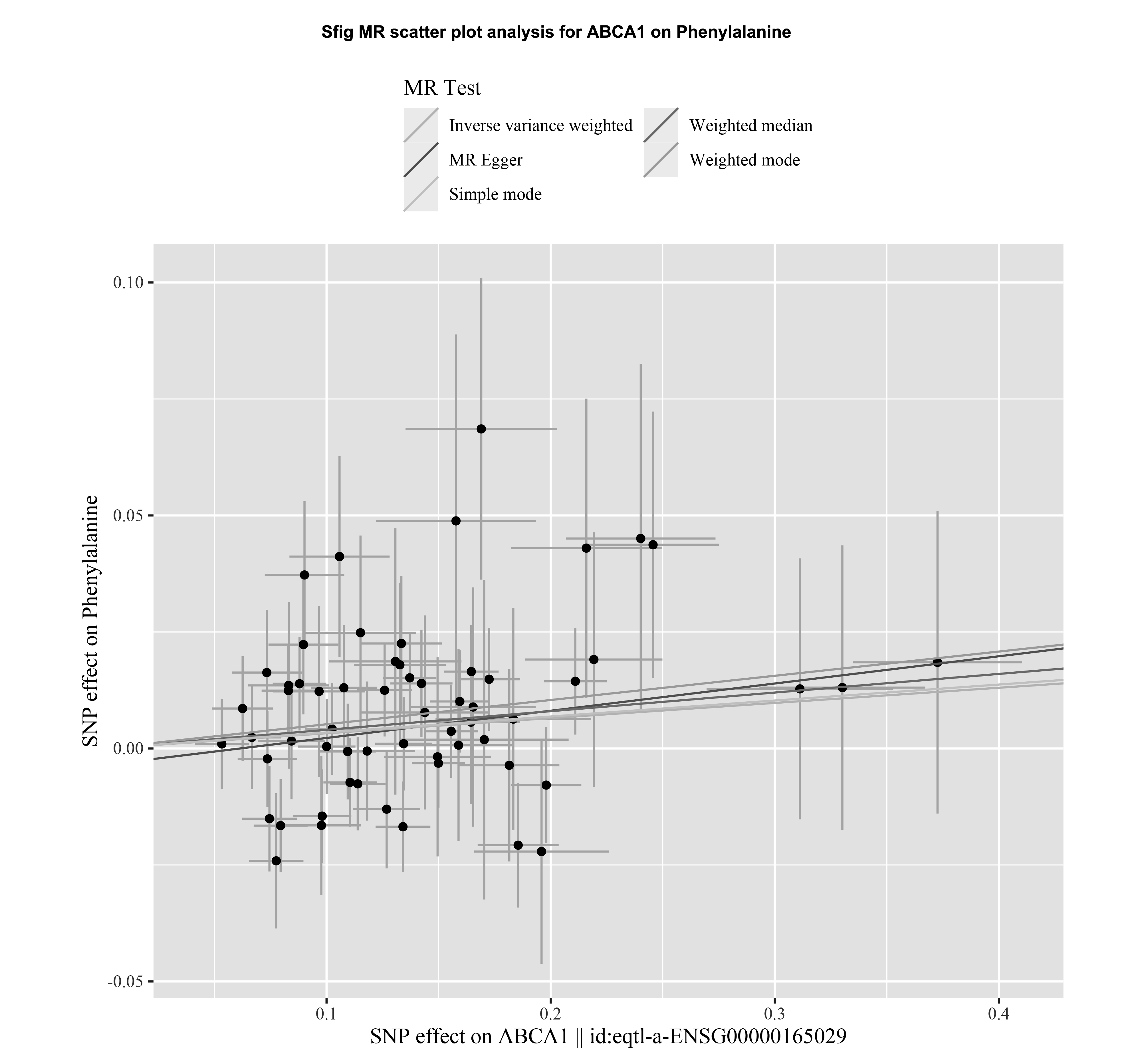

Supplement: Supplementary file 3 — Supplementary Information 3. [file 41598_2025_93644_MOESM3_ESM.zip › the scatter plot/Sfig MR scatter plot analysis for ABCA1 on Phenylalanine.tif]

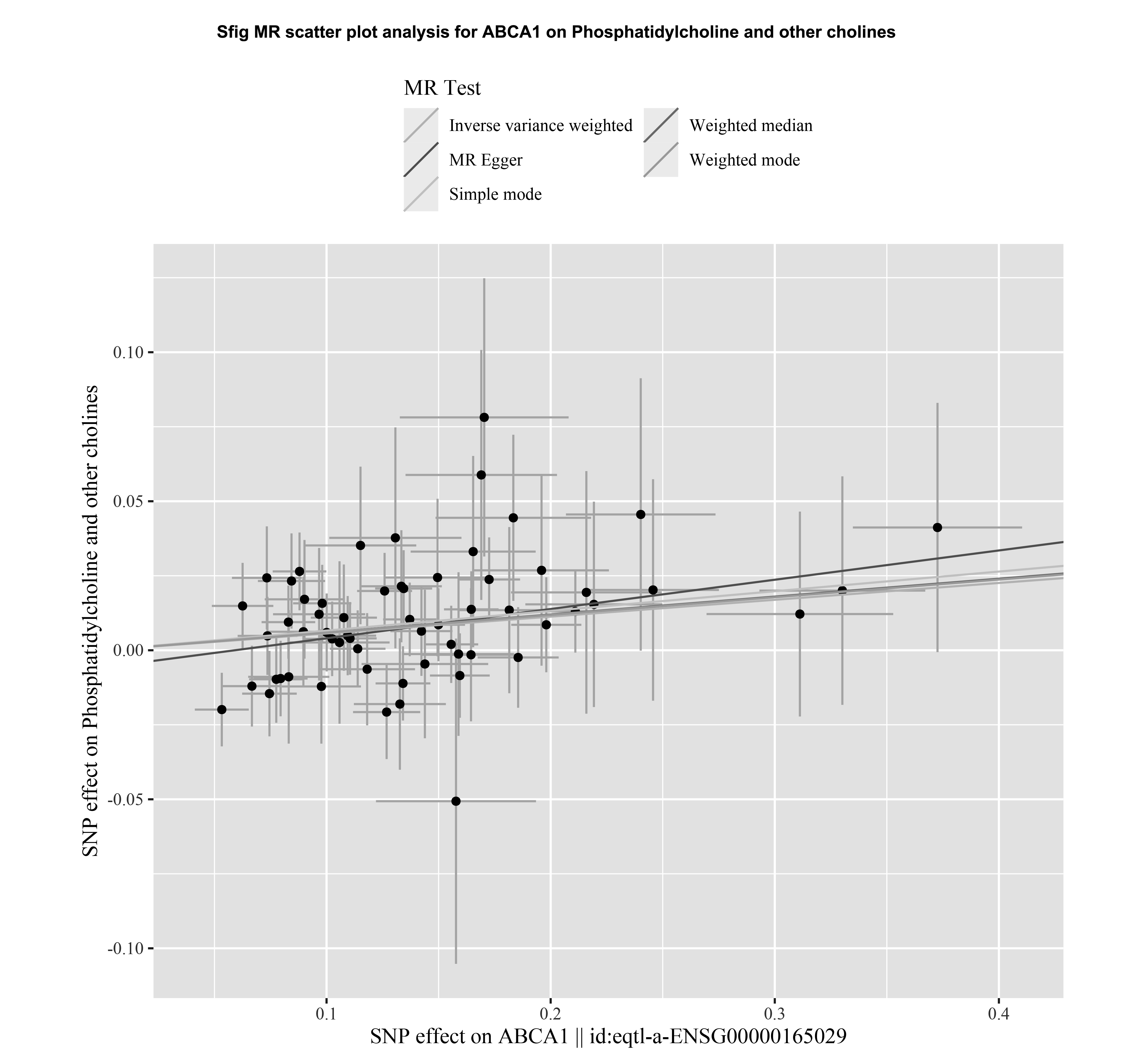

Supplement: Supplementary file 3 — Supplementary Information 3. [file 41598_2025_93644_MOESM3_ESM.zip › the scatter plot/Sfig MR scatter plot analysis for ABCA1 on Phosphatidylcholine and other cholines.tif]

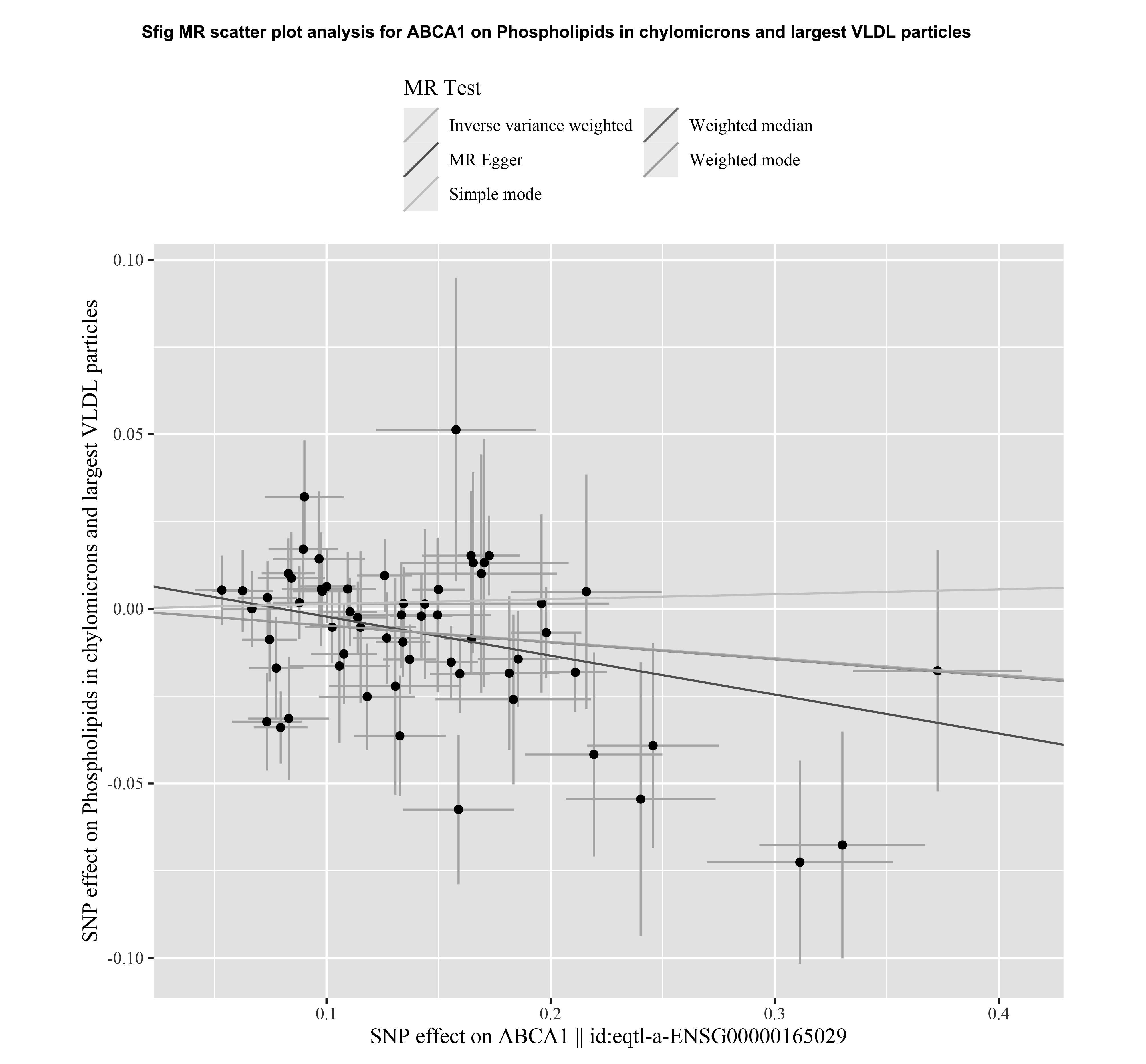

Supplement: Supplementary file 3 — Supplementary Information 3. [file 41598_2025_93644_MOESM3_ESM.zip › the scatter plot/Sfig MR scatter plot analysis for ABCA1 on Phospholipids in chylomicrons and largest VLDL particles.tif]

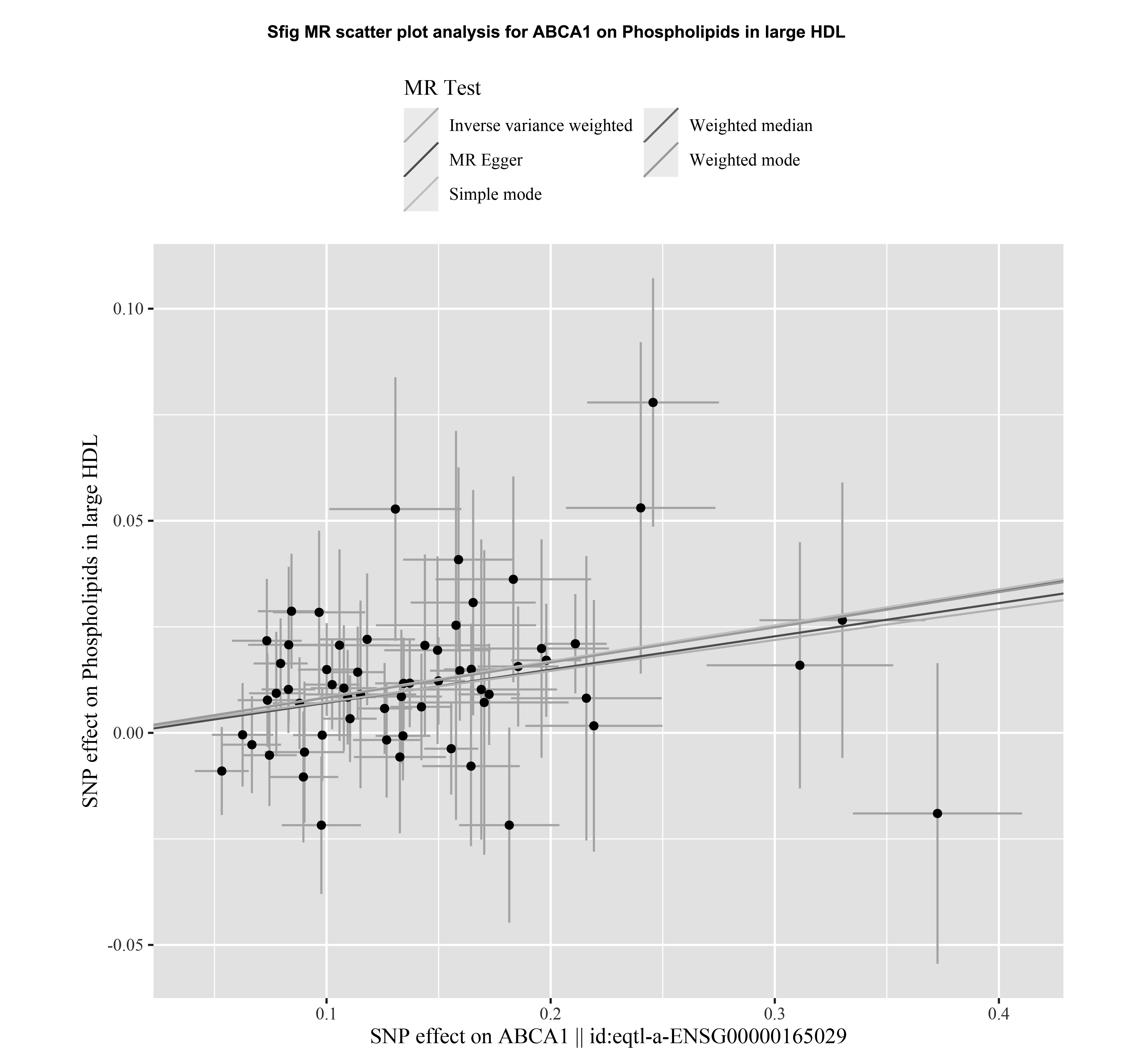

Supplement: Supplementary file 3 — Supplementary Information 3. [file 41598_2025_93644_MOESM3_ESM.zip › the scatter plot/Sfig MR scatter plot analysis for ABCA1 on Phospholipids in large HDL.tif]

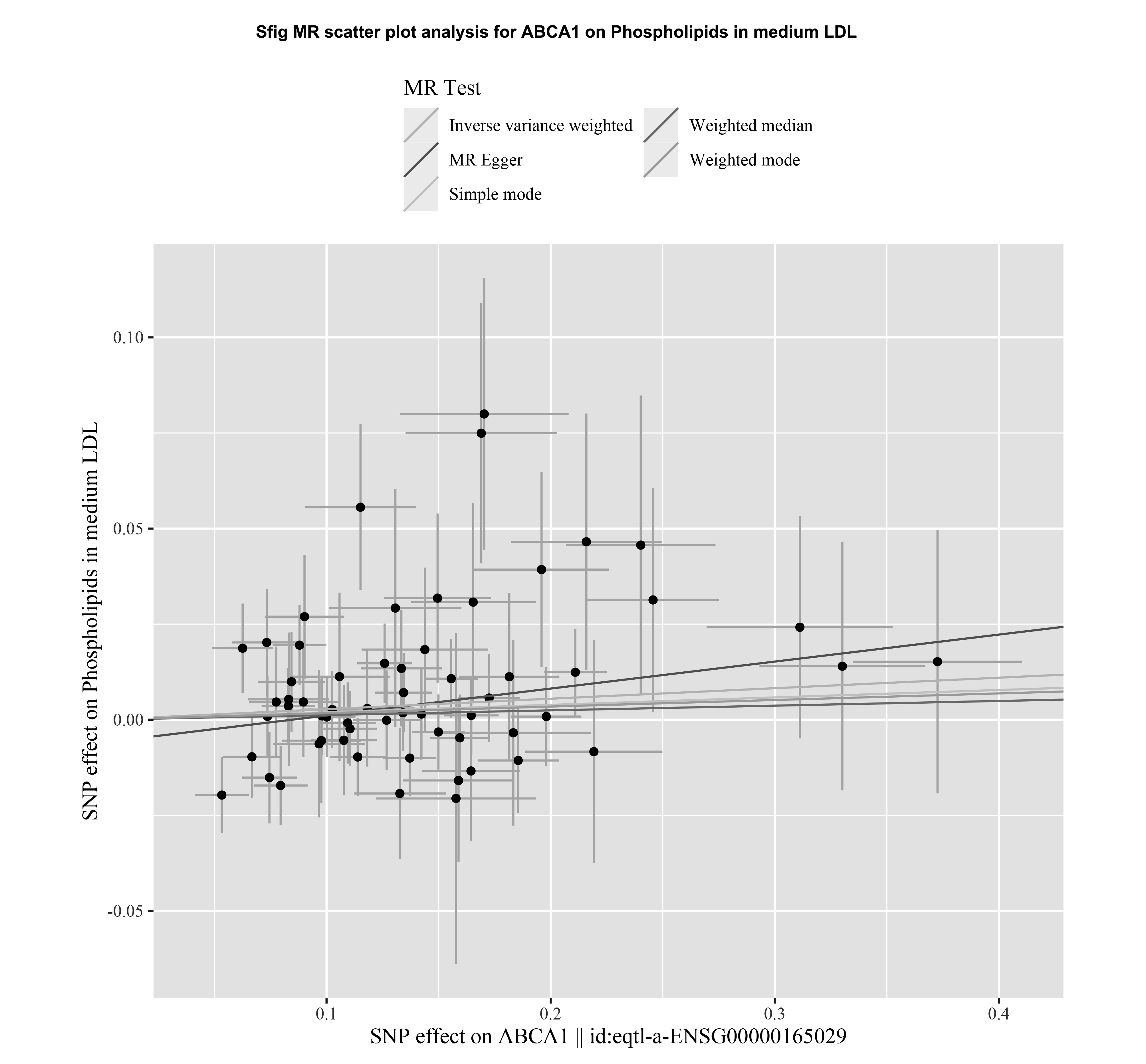

Supplement: Supplementary file 3 — Supplementary Information 3. [file 41598_2025_93644_MOESM3_ESM.zip › the scatter plot/Sfig MR scatter plot analysis for ABCA1 on Phospholipids in medium LDL.tif]

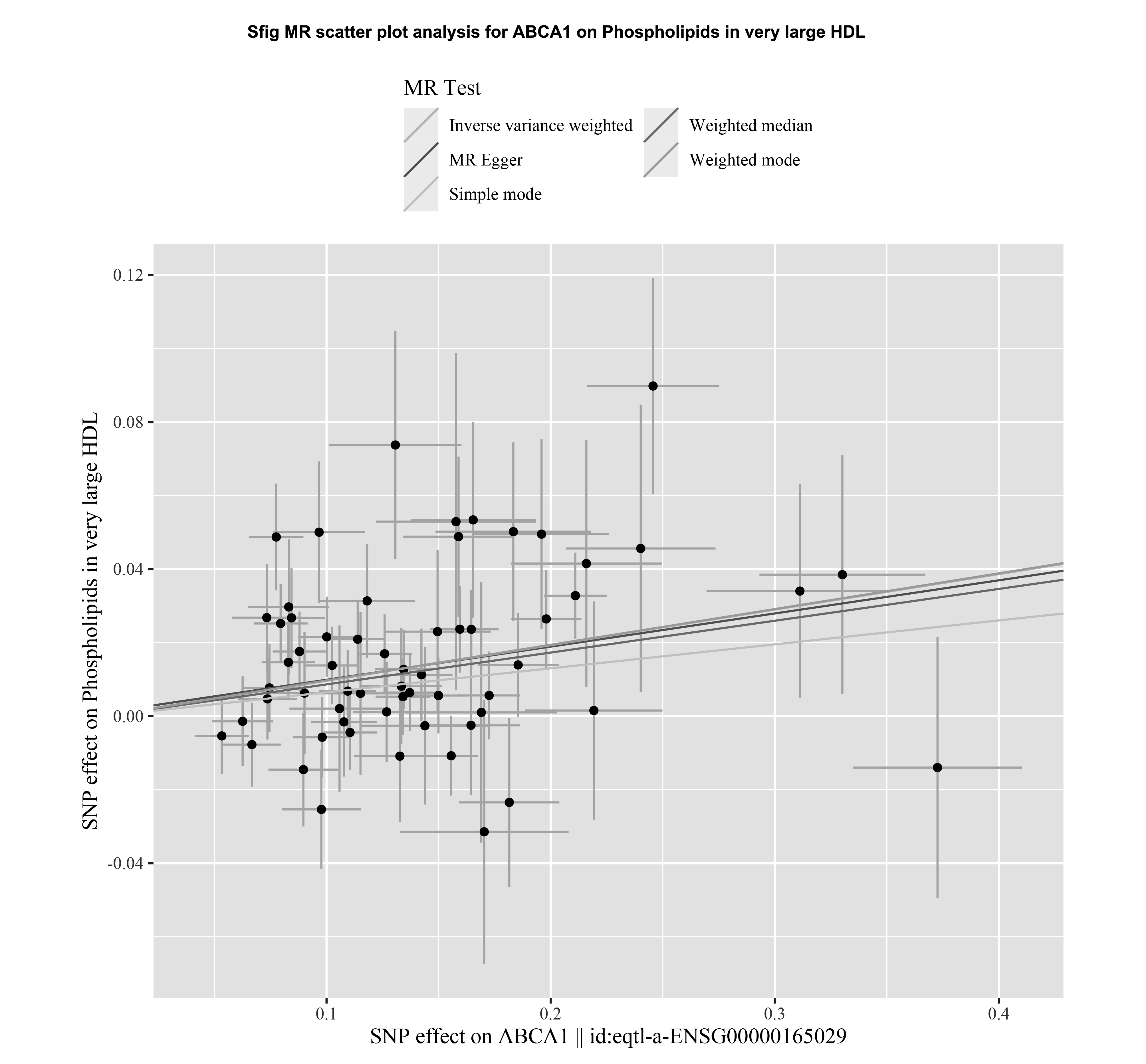

Supplement: Supplementary file 3 — Supplementary Information 3. [file 41598_2025_93644_MOESM3_ESM.zip › the scatter plot/Sfig MR scatter plot analysis for ABCA1 on Phospholipids in very large HDL.tif]

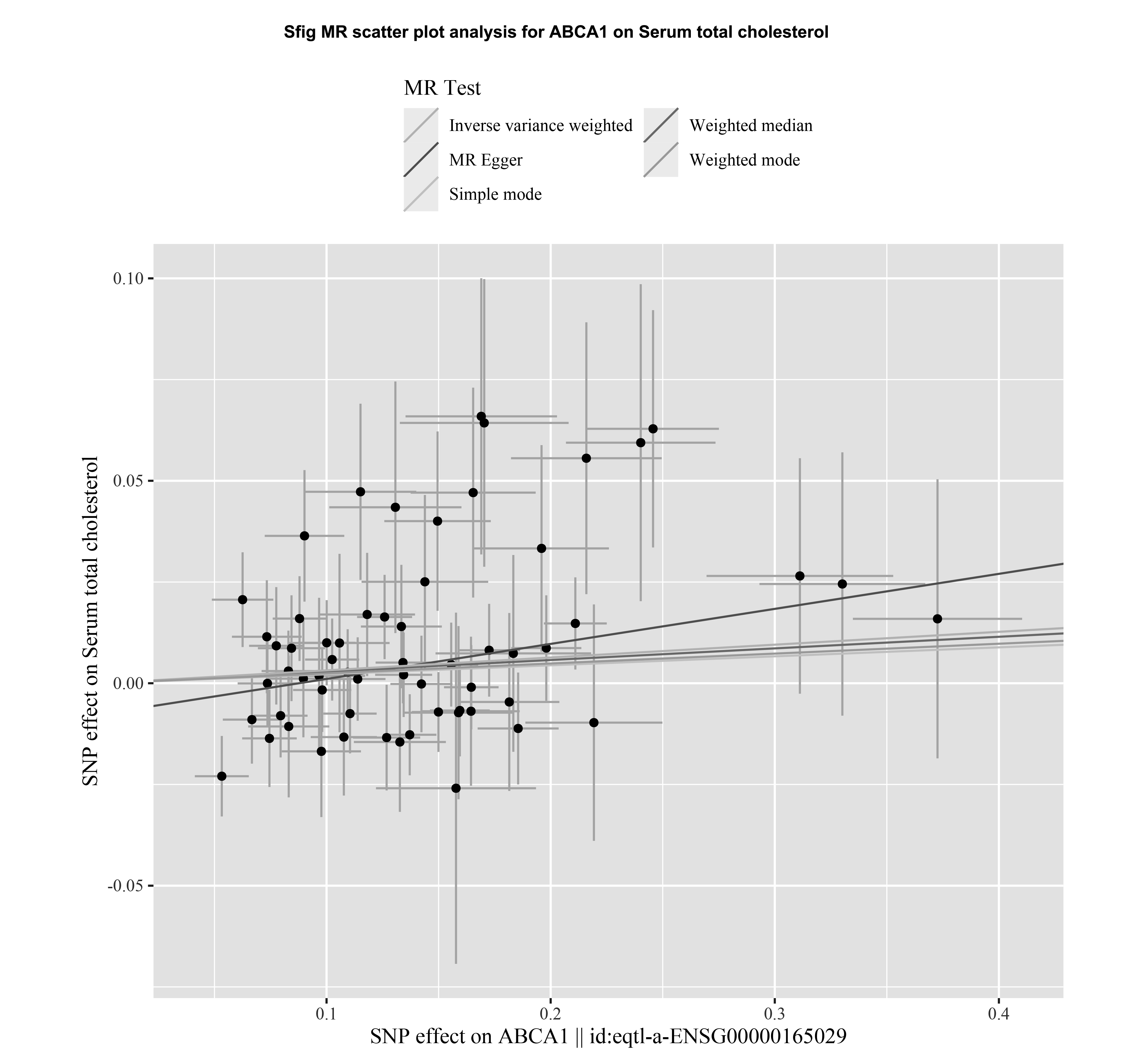

Supplement: Supplementary file 3 — Supplementary Information 3. [file 41598_2025_93644_MOESM3_ESM.zip › the scatter plot/Sfig MR scatter plot analysis for ABCA1 on Serum total cholesterol.tif]

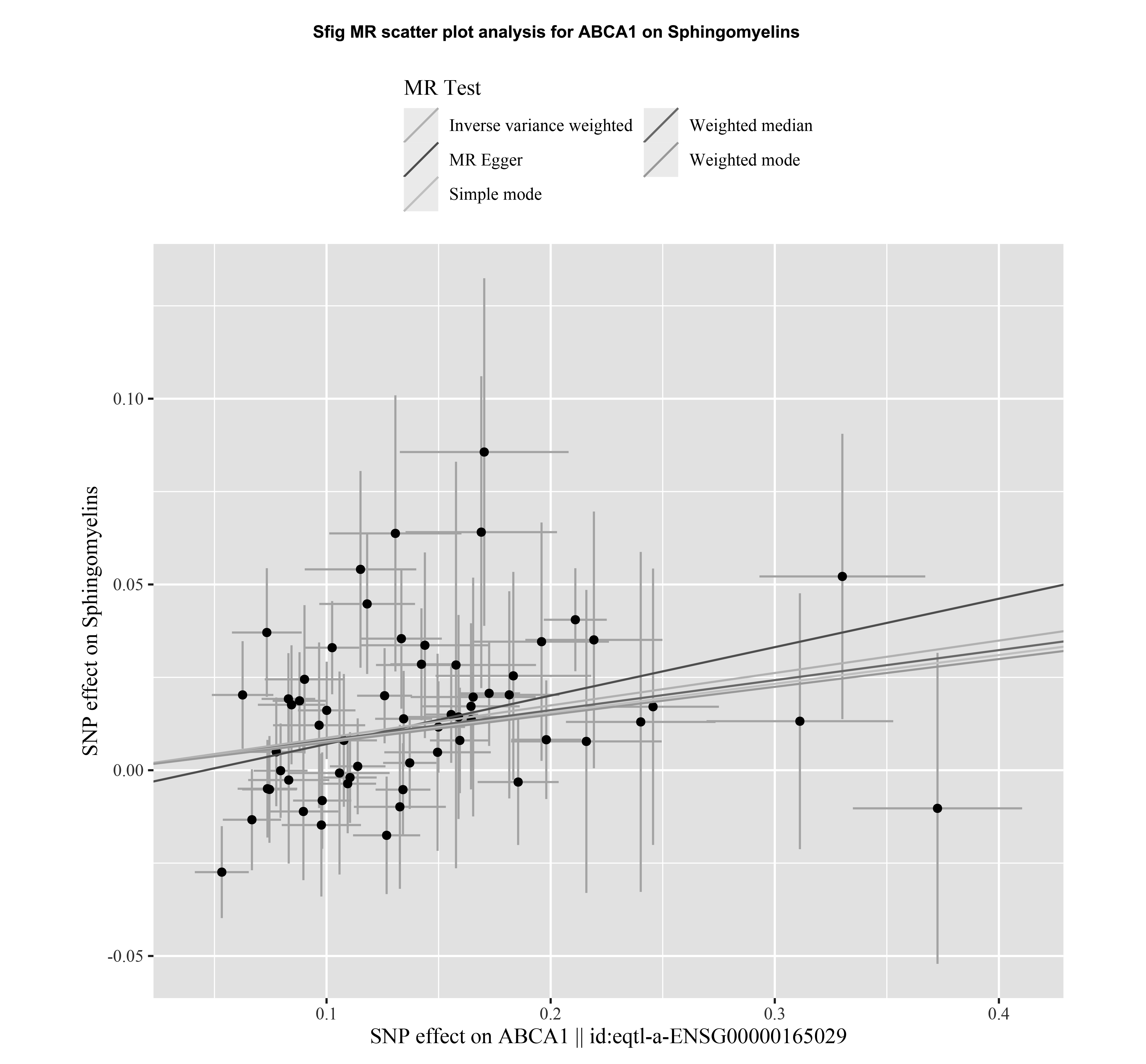

Supplement: Supplementary file 3 — Supplementary Information 3. [file 41598_2025_93644_MOESM3_ESM.zip › the scatter plot/Sfig MR scatter plot analysis for ABCA1 on Sphingomyelins.tif]

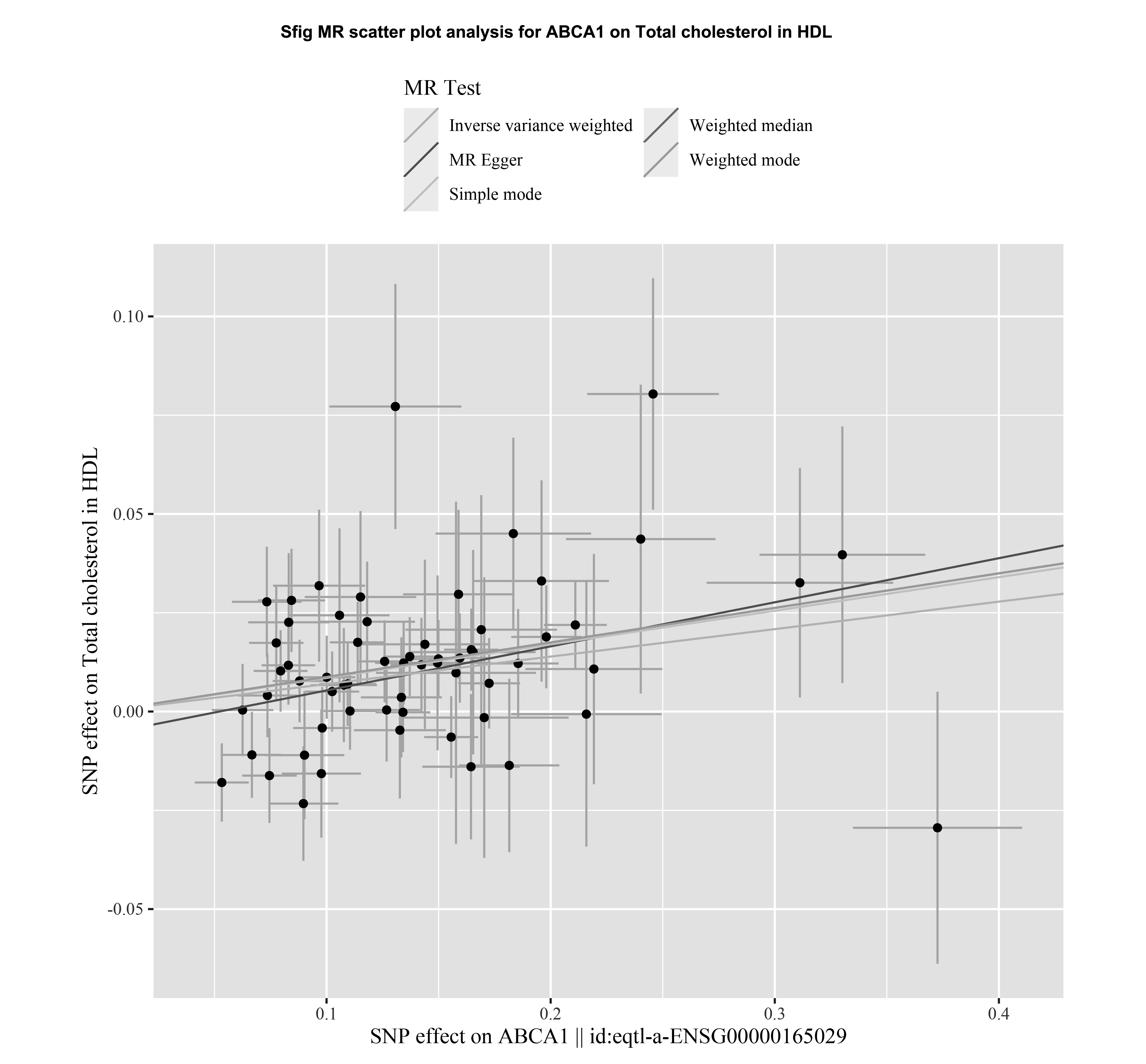

Supplement: Supplementary file 3 — Supplementary Information 3. [file 41598_2025_93644_MOESM3_ESM.zip › the scatter plot/Sfig MR scatter plot analysis for ABCA1 on Total cholesterol in HDL.tif]

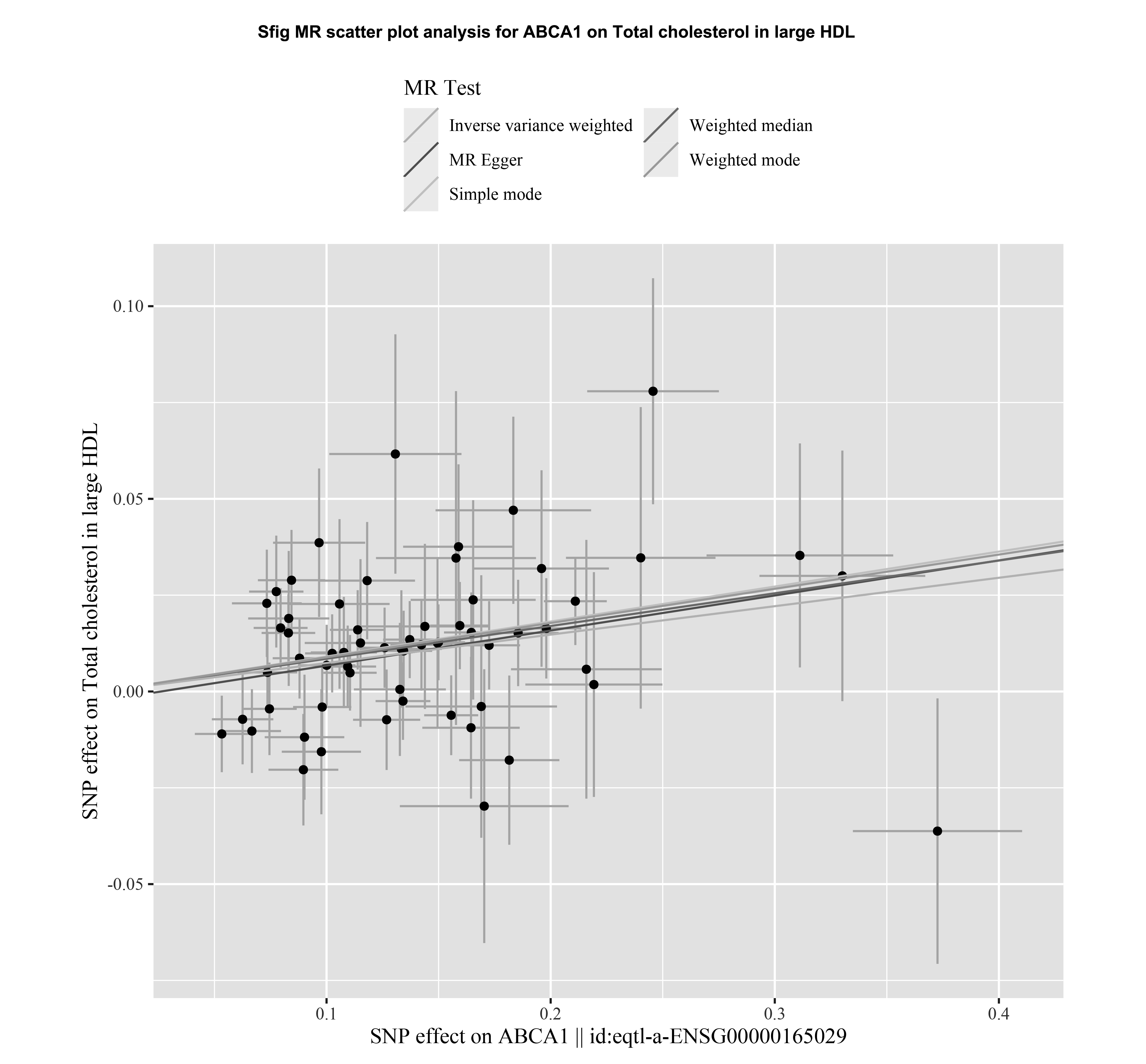

Supplement: Supplementary file 3 — Supplementary Information 3. [file 41598_2025_93644_MOESM3_ESM.zip › the scatter plot/Sfig MR scatter plot analysis for ABCA1 on Total cholesterol in large HDL.tif]

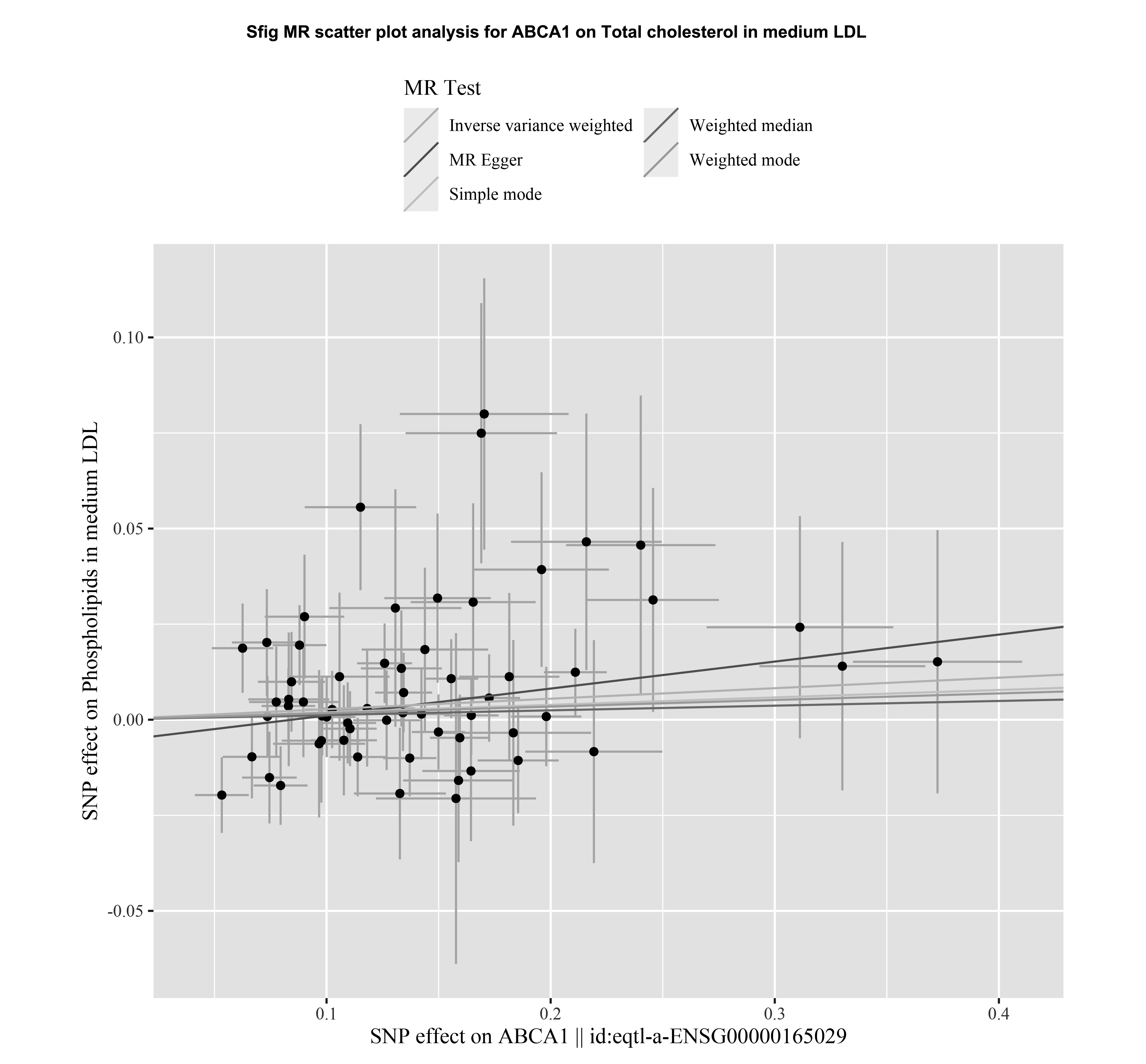

Supplement: Supplementary file 3 — Supplementary Information 3. [file 41598_2025_93644_MOESM3_ESM.zip › the scatter plot/Sfig MR scatter plot analysis for ABCA1 on Total cholesterol in medium LDL.tif]

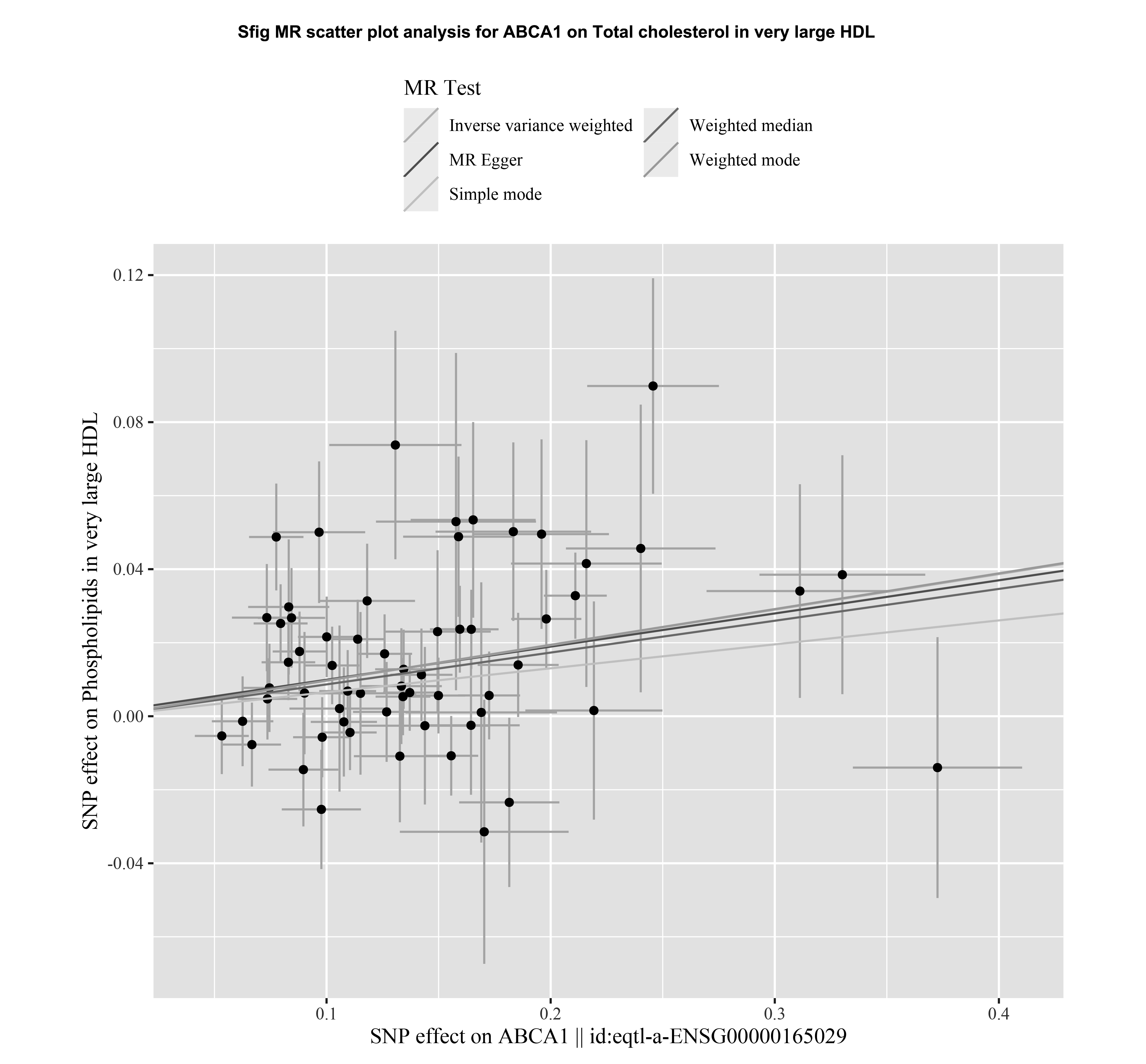

Supplement: Supplementary file 3 — Supplementary Information 3. [file 41598_2025_93644_MOESM3_ESM.zip › the scatter plot/Sfig MR scatter plot analysis for ABCA1 on Total cholesterol in very large HDL.tif]

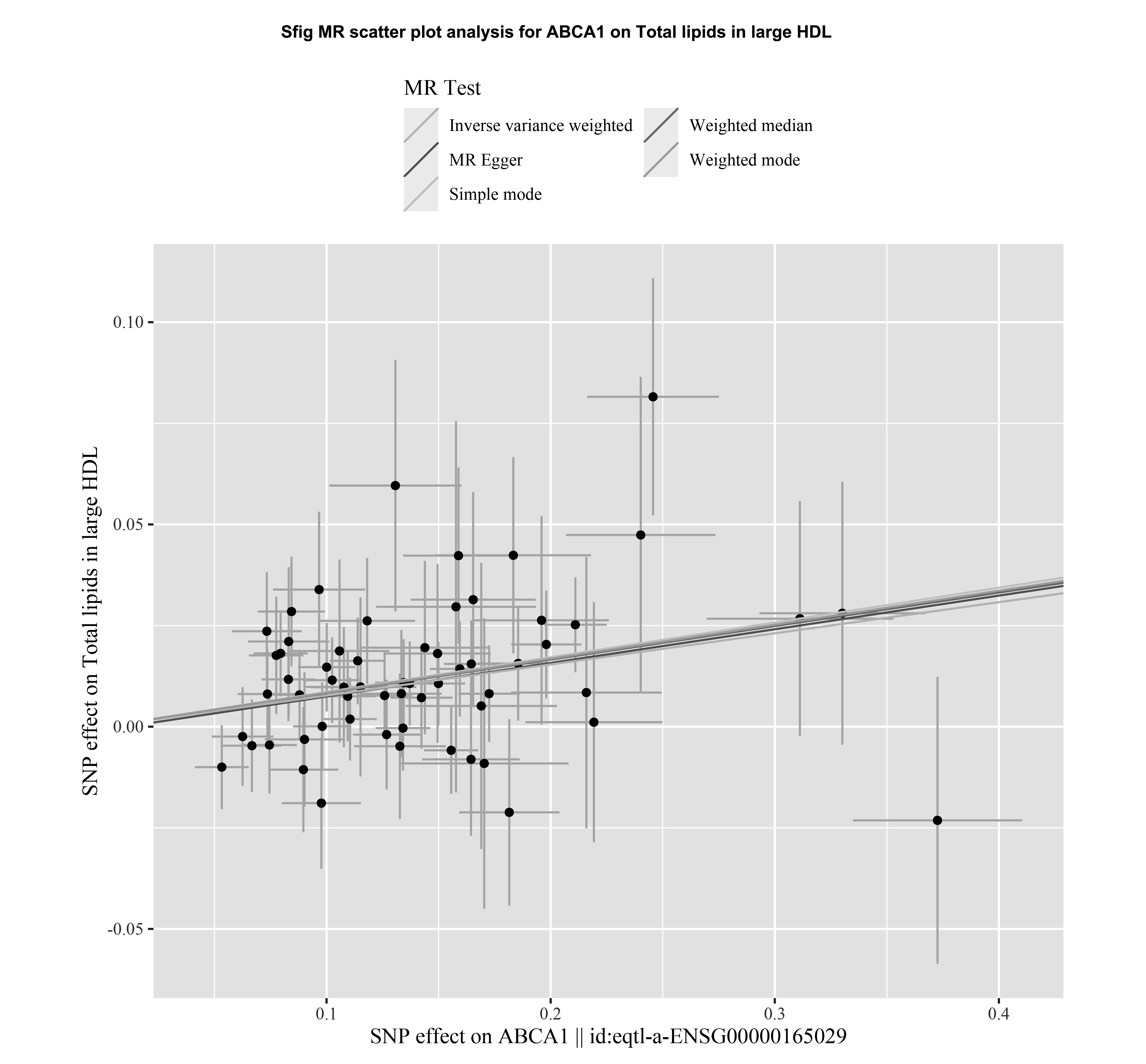

Supplement: Supplementary file 3 — Supplementary Information 3. [file 41598_2025_93644_MOESM3_ESM.zip › the scatter plot/Sfig MR scatter plot analysis for ABCA1 on Total lipids in large HDL.tif]

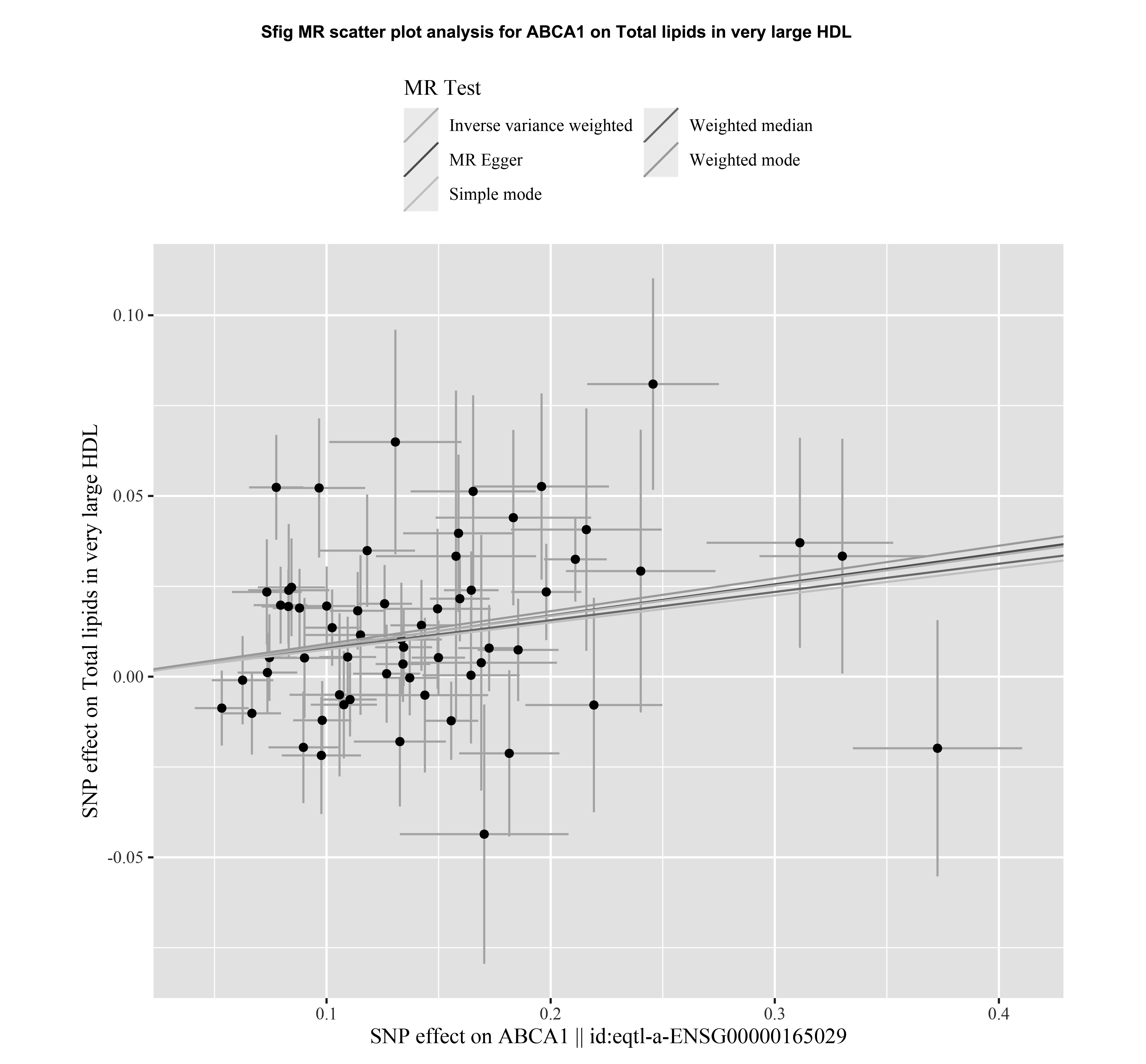

Supplement: Supplementary file 3 — Supplementary Information 3. [file 41598_2025_93644_MOESM3_ESM.zip › the scatter plot/Sfig MR scatter plot analysis for ABCA1 on Total lipids in very large HDL.tif]

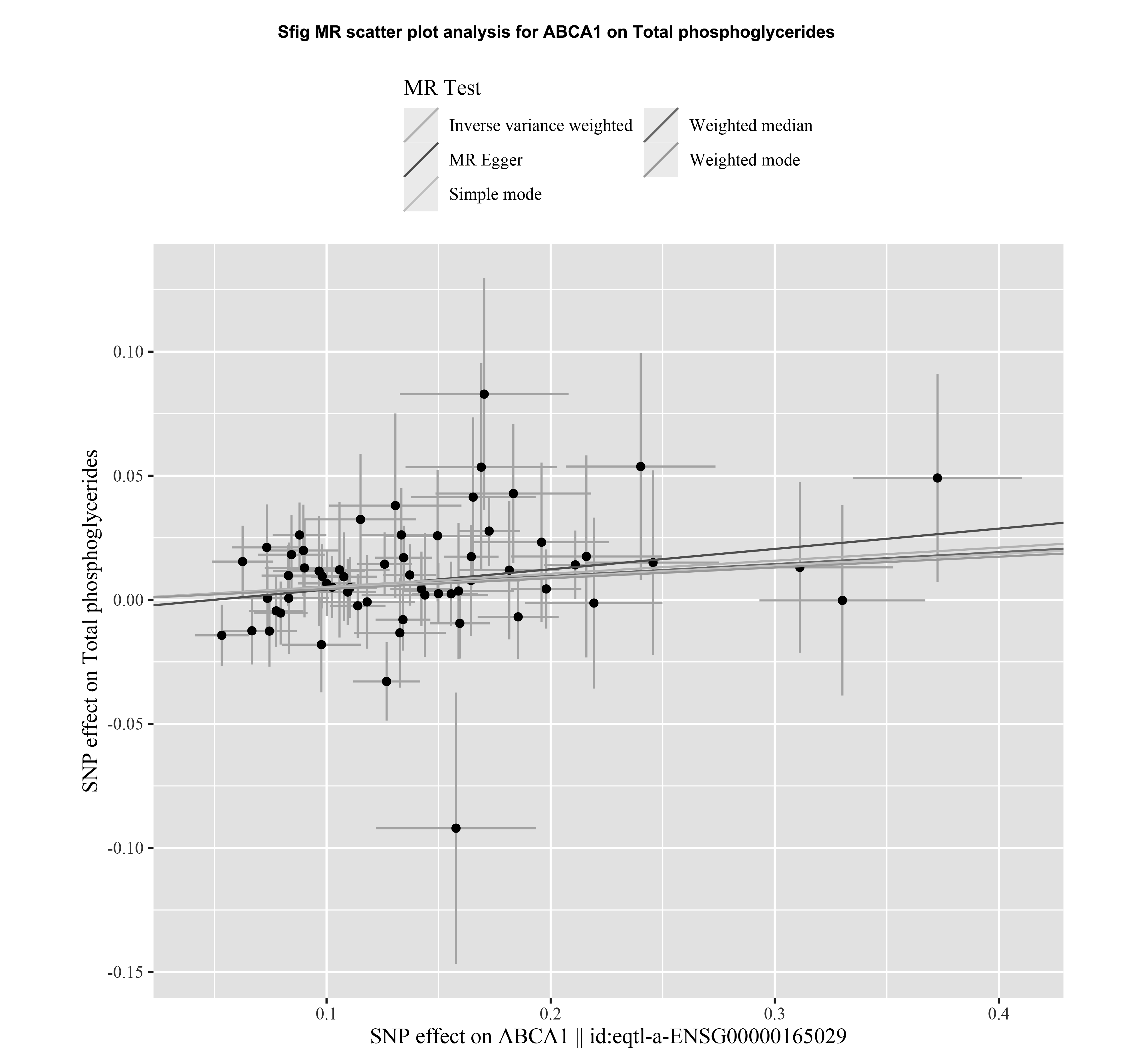

Supplement: Supplementary file 3 — Supplementary Information 3. [file 41598_2025_93644_MOESM3_ESM.zip › the scatter plot/Sfig MR scatter plot analysis for ABCA1 on Total phosphoglycerides.tif]

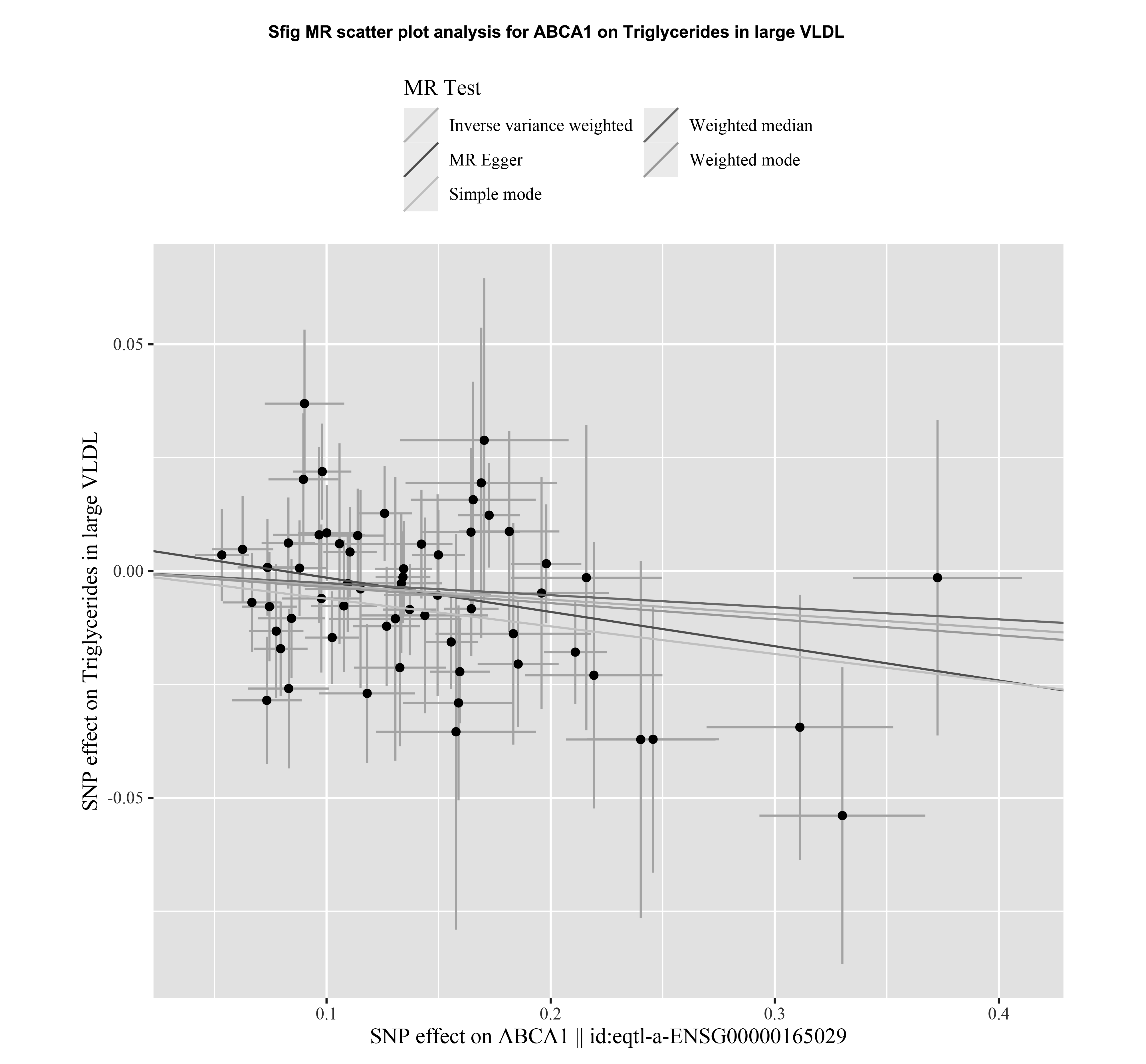

Supplement: Supplementary file 3 — Supplementary Information 3. [file 41598_2025_93644_MOESM3_ESM.zip › the scatter plot/Sfig MR scatter plot analysis for ABCA1 on Triglycerides in large VLDL.tif]

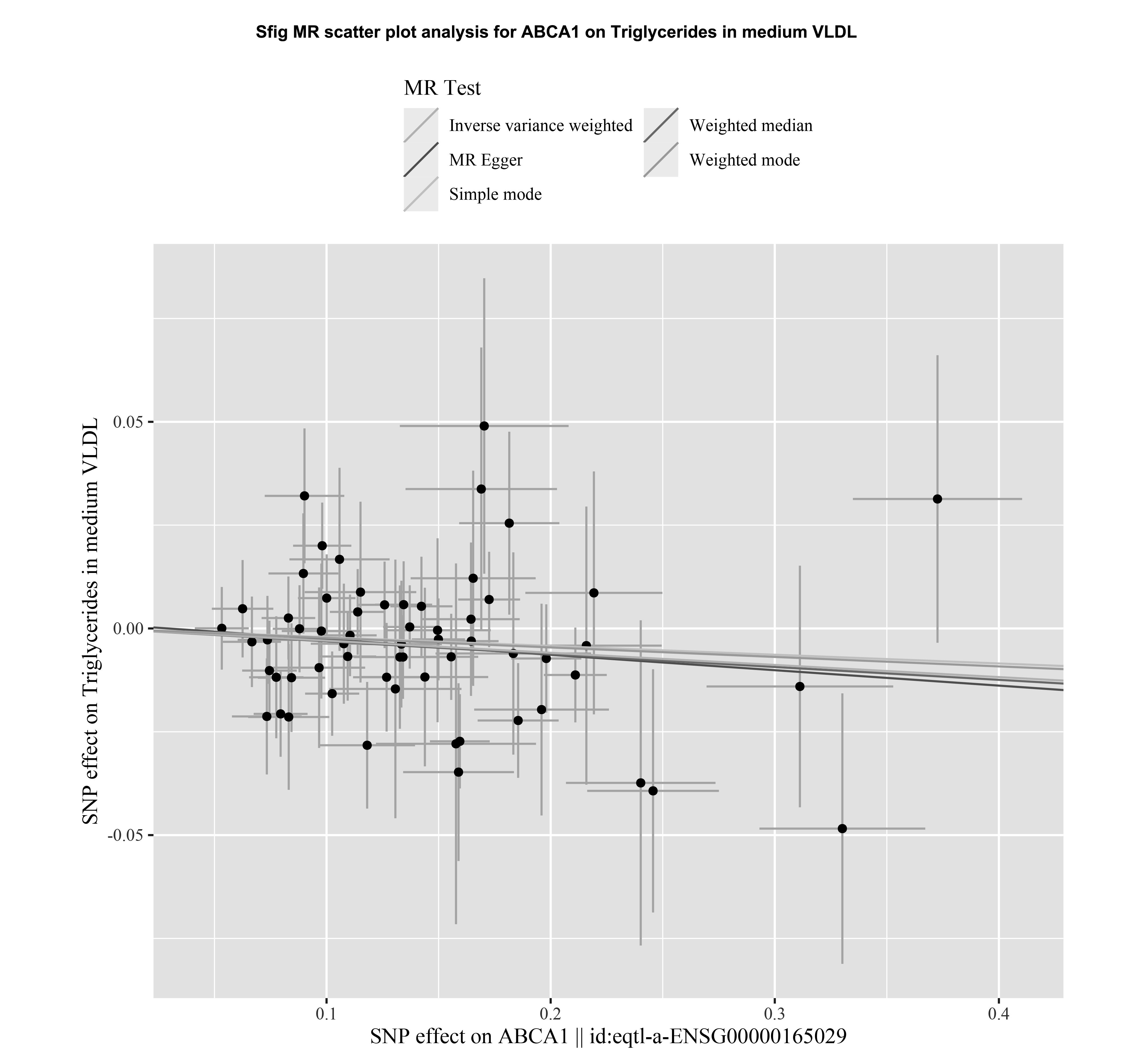

Supplement: Supplementary file 3 — Supplementary Information 3. [file 41598_2025_93644_MOESM3_ESM.zip › the scatter plot/Sfig MR scatter plot analysis for ABCA1 on Triglycerides in medium VLDL.tif]

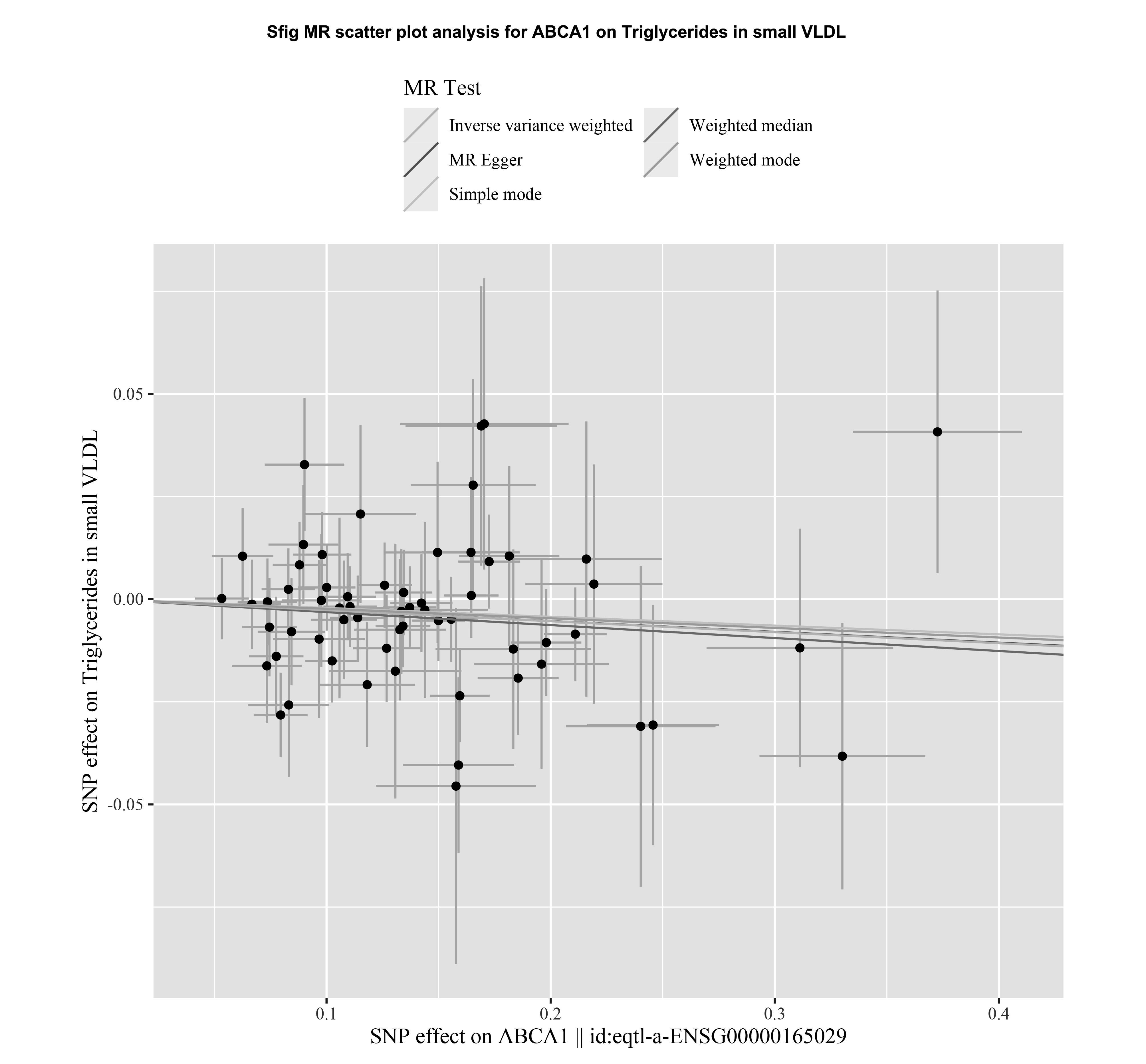

Supplement: Supplementary file 3 — Supplementary Information 3. [file 41598_2025_93644_MOESM3_ESM.zip › the scatter plot/Sfig MR scatter plot analysis for ABCA1 on Triglycerides in small VLDL.tif]

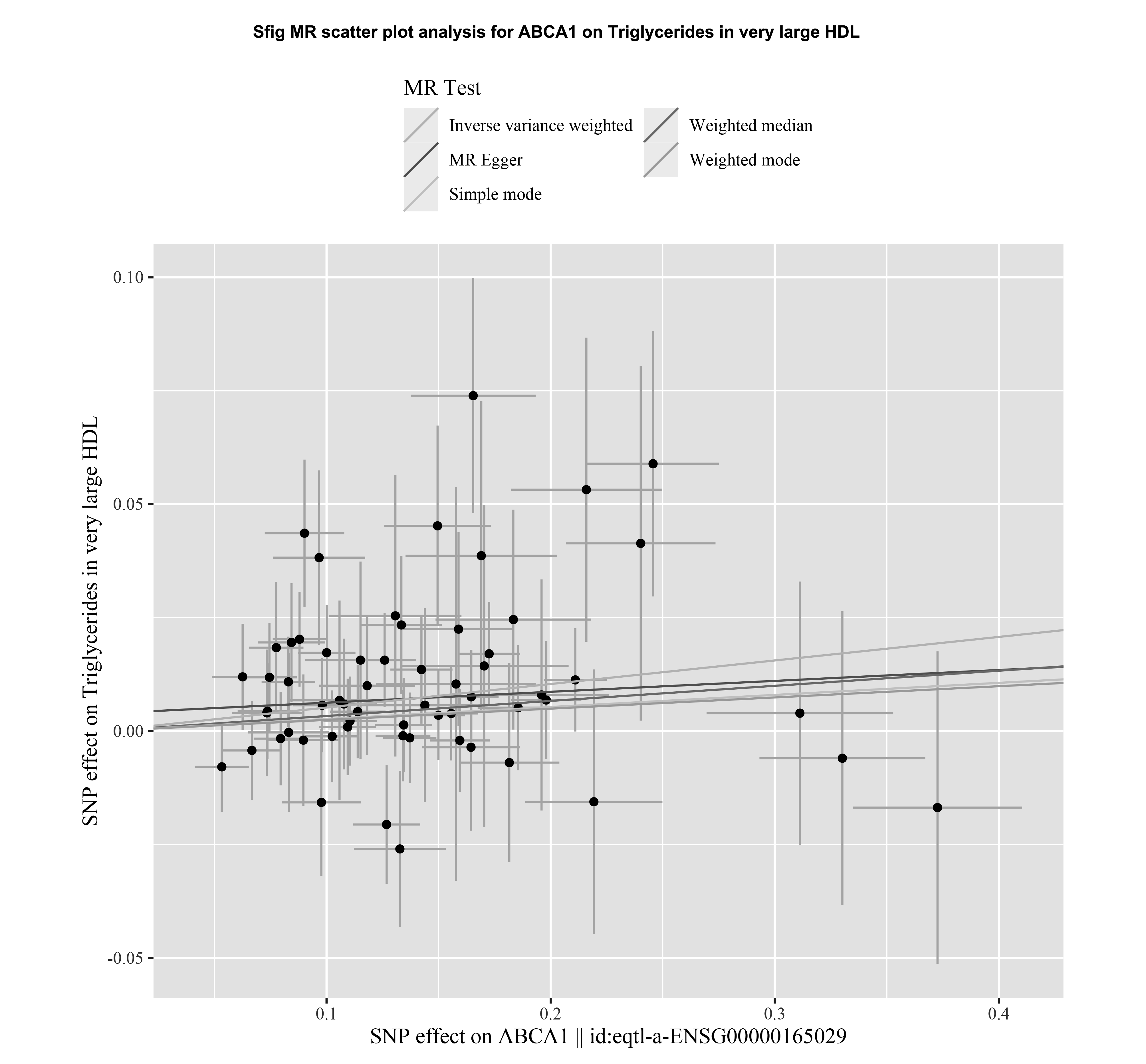

Supplement: Supplementary file 3 — Supplementary Information 3. [file 41598_2025_93644_MOESM3_ESM.zip › the scatter plot/Sfig MR scatter plot analysis for ABCA1 on Triglycerides in very large HDL.tif]

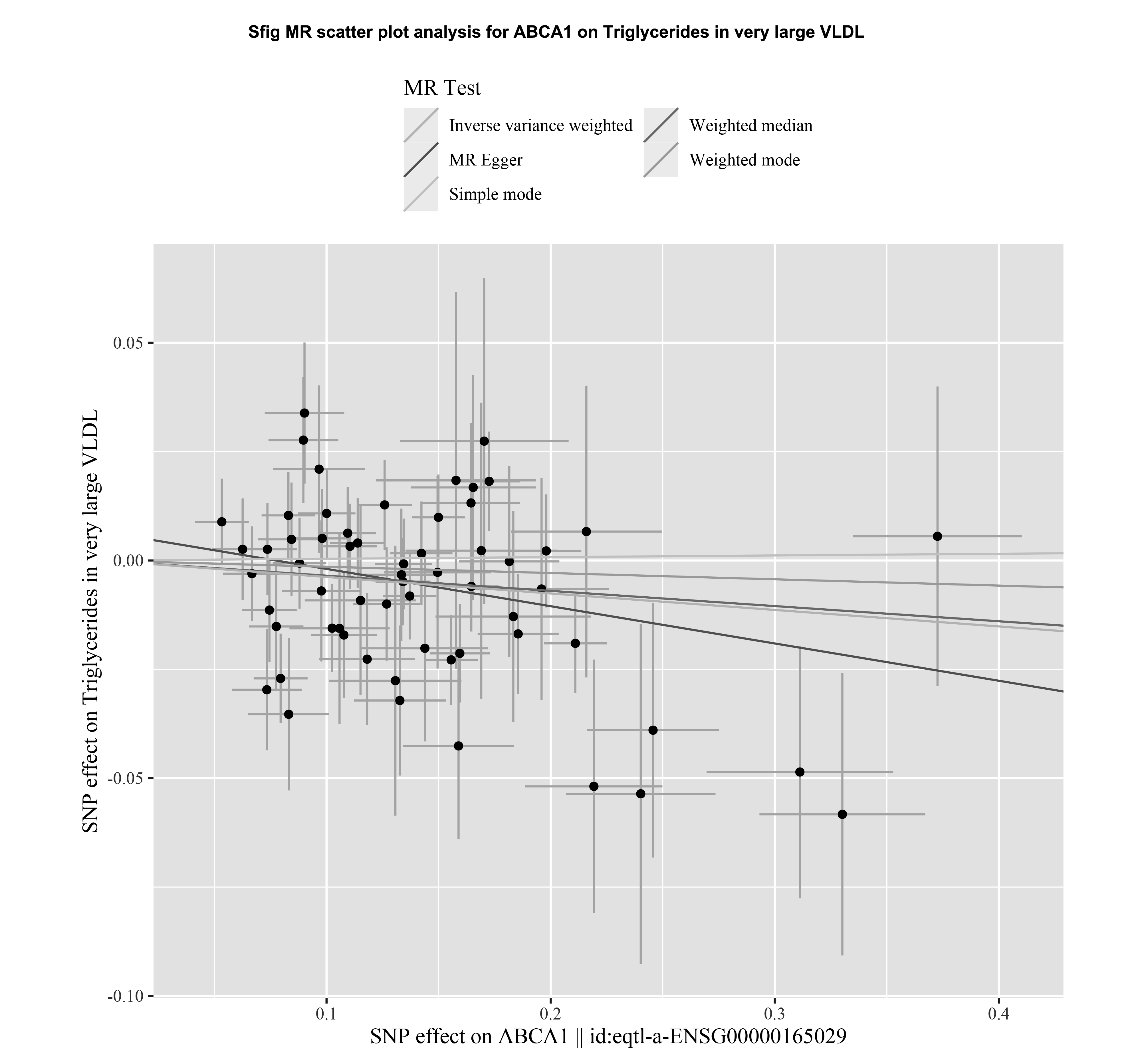

Supplement: Supplementary file 3 — Supplementary Information 3. [file 41598_2025_93644_MOESM3_ESM.zip › the scatter plot/Sfig MR scatter plot analysis for ABCA1 on Triglycerides in very large VLDL.tif]

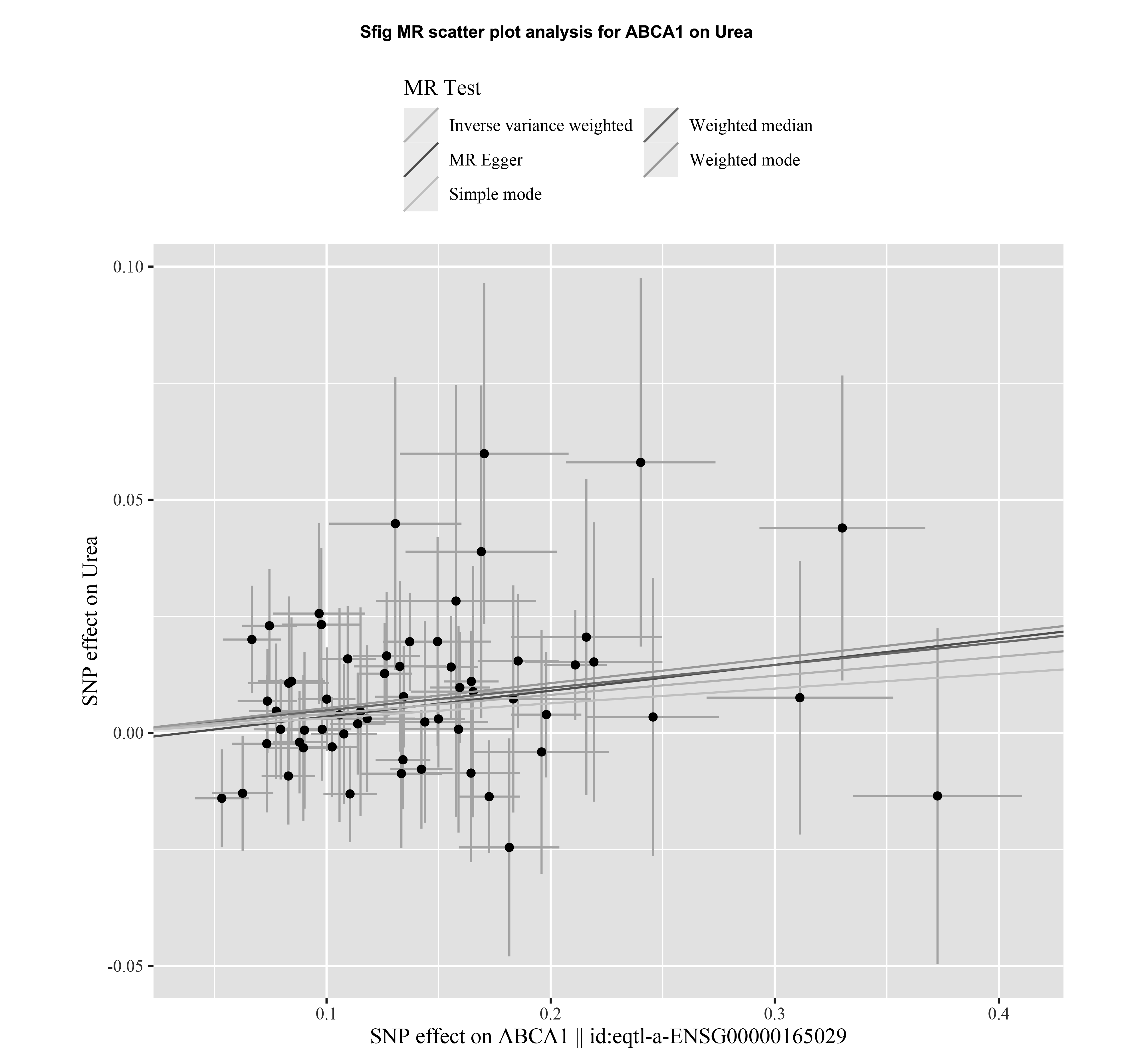

Supplement: Supplementary file 3 — Supplementary Information 3. [file 41598_2025_93644_MOESM3_ESM.zip › the scatter plot/Sfig MR scatter plot analysis for ABCA1 on Urea.tif]

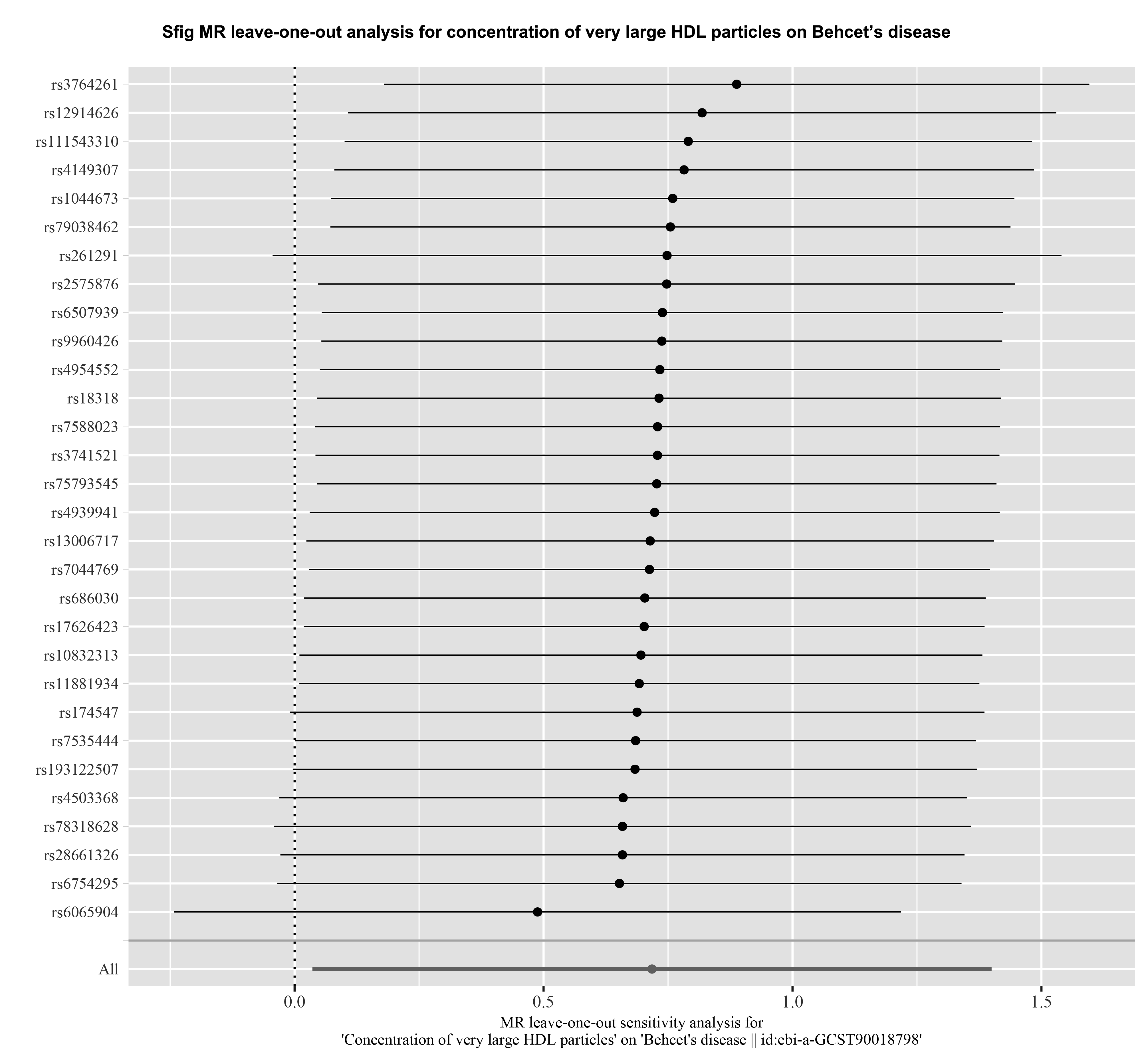

Supplement: Supplementary file 4 — Supplementary Information 4. [file 41598_2025_93644_MOESM4_ESM.zip › leave-one-out analysis/Sfig MR leave-one-out analysis for concentration of very large HDL particles on Behcet’s disease.tif]

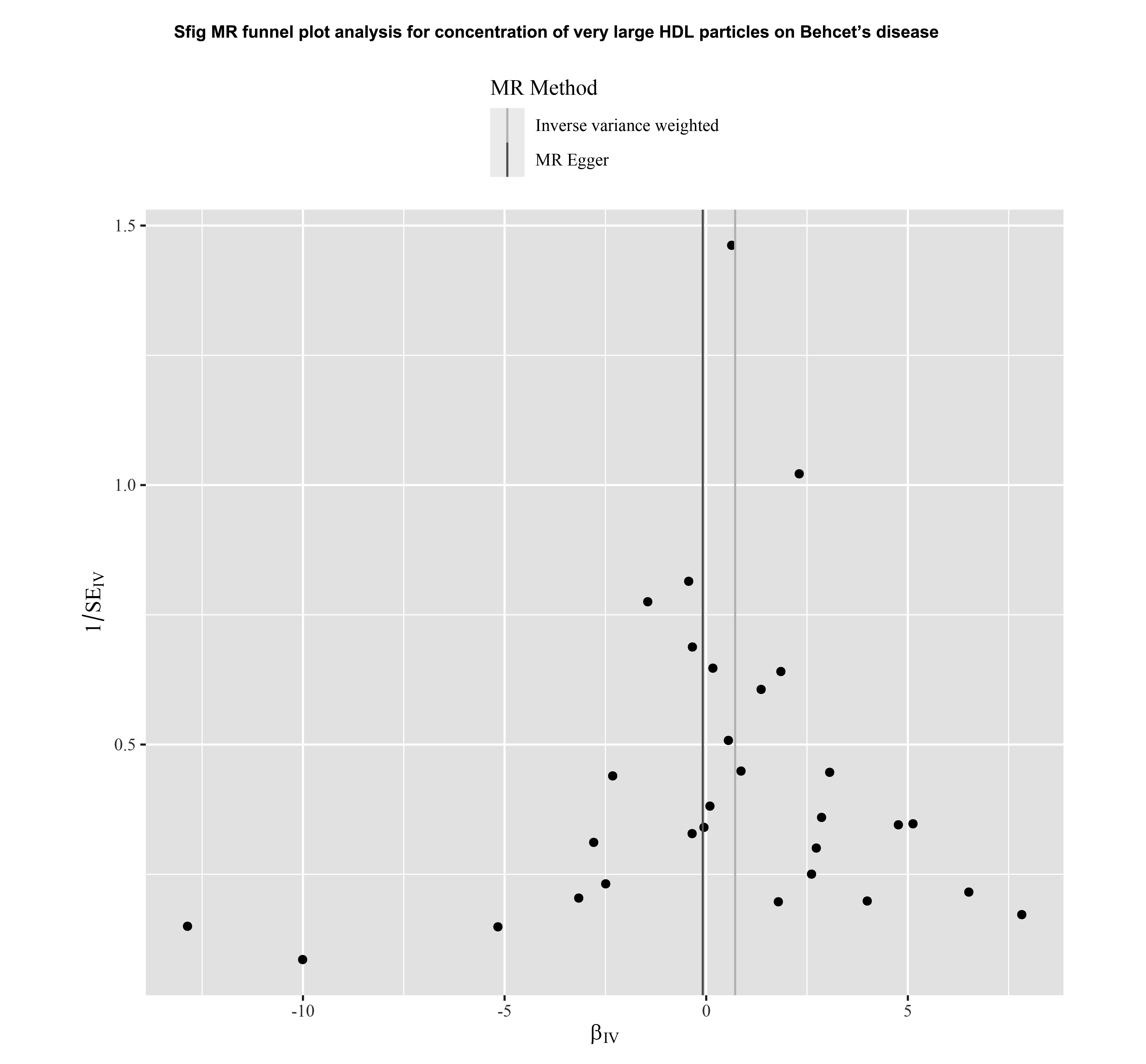

Supplement: Supplementary file 4 — Supplementary Information 4. [file 41598_2025_93644_MOESM4_ESM.zip › the funnel plot/Sfig MR scatter funnel plot analysis for concentration of very large HDL particles on Behcet’s disease.tif]

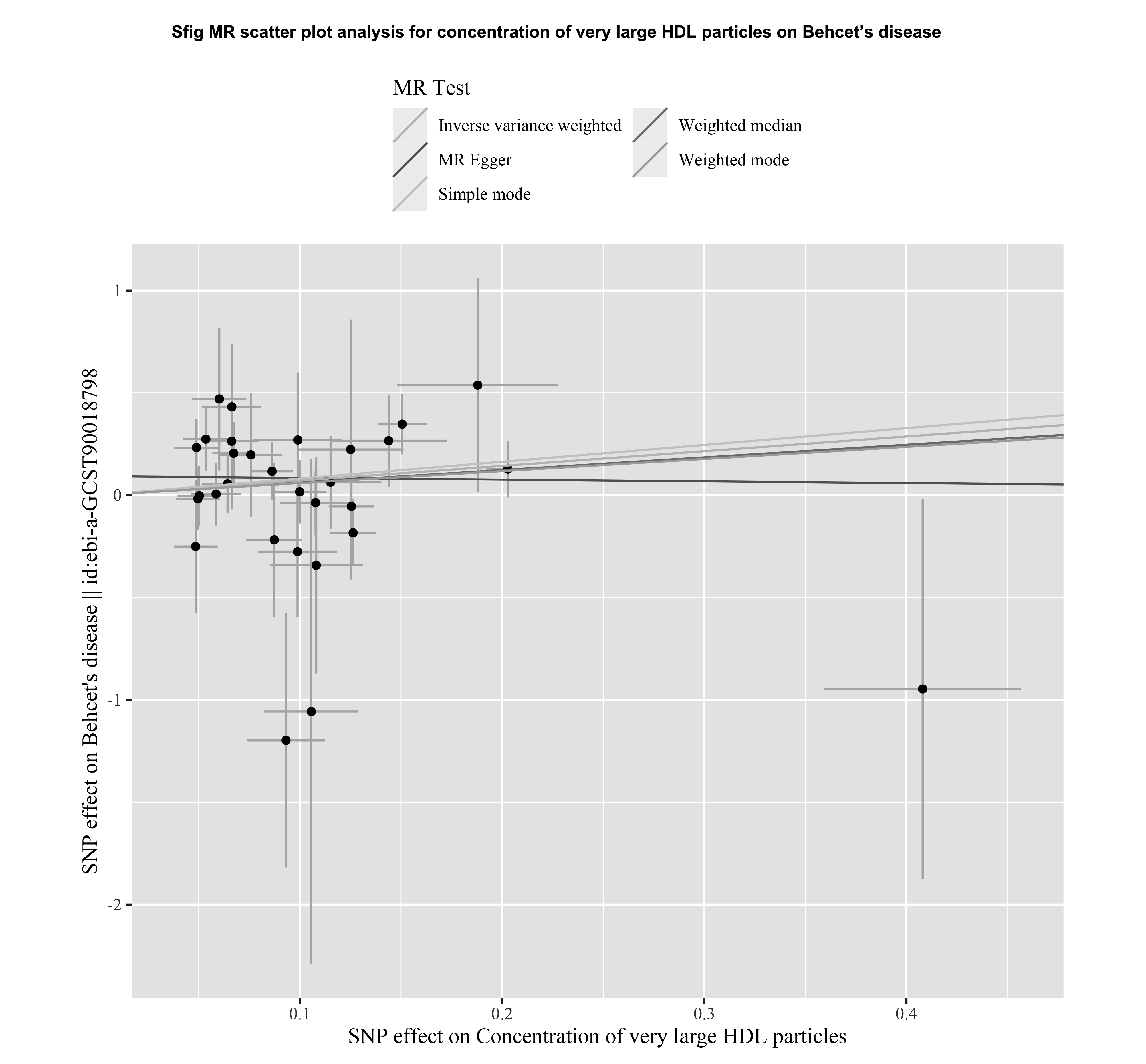

Supplement: Supplementary file 4 — Supplementary Information 4. [file 41598_2025_93644_MOESM4_ESM.zip › the scatter plot/Sfig MR scatter plot analysis for concentration of very large HDL particles on Behcet’s disease.tif]
